# Supplementary material for: Mixed-methods feasibility outcomes for a novel ACT-based video game ‘ACTing Minds’ to support mental health
Source: BMJ Open. 2024 Mar 29;14(3):e080972. doi: 10.1136/bmjopen-2023-080972 (PMC10982759; doi:10.1136/bmjopen-2023-080972)
Supplement: Supplementary data [file bmjopen-2023-080972supp002.pdf]

Swansea University
Prifysgol Abertawe

PARTICIPANT INFORMATION SHEET

A novel ACT-based video game to support mental health through embedded learning

You are being invited to take part in some research. Before you decide whether or not to participate, it is important for you to understand why the research is being conducted and what it will involve. Please read the following information carefully.

What is the purpose of the research?

The aim of the research project is to gain insight into whether exposure to a novel-acceptance and commitment therapy (ACT) video game can improve various outcome measures of depression, anxiety, stress, and other measures relating to wellbeing.

If you are above the age of 18 and are currently experiencing ongoing mild to moderate levels of depression, anxiety, or stress you are welcome to participate in this study. For those individuals who present with high levels of depression and anxiety, we will advise the participant to consult either with a GP or with a therapist if they have one, in relation to participating in this experiment.

Participation in this study will take approximately 3 hours over three weeks. Which is broken down into 1 hour for the intervention (the video game itself); 1 hour for online one-on-one qualitative audio interviews via Zoom after completing the game, and after a 3-week follow-up; and 1 hour for the three-questionnaire assessments at baseline (pre-intervention), immediate post-intervention, and after a 3-week follow-up.

Who is carrying out the research?

The data are being collected by Prof. Andrew Kemp and research assistant Tom Gordon of the Psychology Department within the College of Human and Health Sciences at Swansea University, as well as Dr. Darren Edwards of The Department of Public Health, Policy, and Social Sciences. The research has been approved by the Department of Psychology's Research Ethics Committee.

What happens if I agree to take part?

If you are happy to take part in this research project, please click "accept" at the bottom of the consent section below, which will then allow you to complete a baseline questionnaire assessment. The questionnaire assessment includes a range of multiple-choice questions. These questions cover a range of factors including depression, anxiety, stress, and wellbeing. There are three questionnaire assessments to complete, the first of which is a baseline assessment prior to the intervention commencing, the second will be completed following the intervention, and a follow-up assessment 3 weeks following the baseline assessment. All of the questionnaires ask the same questions with the addition of demographic questions at baseline assessment, and the second also asking about adherence to the intervention.

The intervention will consist of completing a one-hour session of playing an ACT-based video game, followed by completion of a short reflection workbook. The game itself will be completed by the participant at home, using a mobile phone or any iOS device. This is an adventure game that will teach you about psychological resilience. In the game, you will control the character Steve as he embarks on a journey into his own mind, you will help as he confronts painful past memories and learns how to cope with his full potential.

Version 4 4 November 2022

Swansea University
Prifysgol Abertawe

You will also be interviewed, which will be conducted online via zoom, regarding your experiences of the game, focusing on what you felt did and did not work. The interviews will be conducted at two points during the study, post-intervention, and after a 3-week follow-up. Interview audio will be recorded and transcribed, following which the recordings and any personal information will be immediately deleted.

Are there any risks associated with taking part?

We are not aware of any significant risks associated with participation. While some of the questions will ask about existing states of mental health (e.g., levels of current anxiety and depression) – which some people may find uncomfortable – the video game has developed in accordance with recent developments in psychological science to help you manage these, and we expect that participation in this study will help to ameliorate these feelings.

If you feel affected by any issues raised by this research and would like to discuss any concerns, please contact the principal investigators of this study as indicated in the contact details at the bottom of this information sheet. If you feel that you would benefit from further psychological or psychiatric support, we advise you to contact your GP (family doctor) in the first instance. Further information on mental wellbeing can be found at https://www.mind.org.uk/. Additional mental health information can be found at https://www.nhs.uk/mental-health/.

This research has been approved by the Department of Psychology's Research Ethics Committee.

Data Protection and Confidentiality

Your data will be processed in accordance with the Data Protection Act 2018 and the General Data Protection Regulation 2016 (GDPR). All information collected about you will be kept strictly confidential. Your data will only be viewed by the researcher/research team.

Standard ethical procedures will involve you providing your consent to participate in this study by ticking the consent box in the consent section

All electronic data will be stored on password-protected computers.

Please note that the data to be collected for our study will be made anonymous once you have completed all stages of the research, and your response data will not hold any personally identifiable information. We will allocate you with an identifier code, and keep your email information for the duration of the study, so that we can communicate with you at the time points. We will delete contact emails at the end of the study.

All data deposited in Swansea University's OneDrive for Business service is stored within Microsoft's data centres located in the EU. Swansea University retains full ownership and control over the data and is satisfied that the data is properly secured and protected.

The contractual agreements between Microsoft and Swansea University have been negotiated by the JISC on behalf of the UK HE sector and abide by all relevant UK and European legislation. In addition, the UK Government has granted Microsoft Azure and Microsoft Office 365 "OFFICIAL" accreditation. This means that they are accredited to hold or transact public sector data for business conducted at the OFFICIAL level of Security Classification.

What will happen to the information I provide?

Gordon TC, *et al. BMJ Open* 2024; 14:e080972. doi: 10.1136/bmjopen-2023-080972

Swansea University
Prifysgol Abertawe

An analysis of the information will form part of our report at the end of the study and may be presented to interested parties and published in scientific journals and related media. *Note that all information presented in any reports or publications will be anonymous and unidentifiable*.

Is participation voluntary and what if I wish to later withdraw?
Your participation is entirely voluntary – you do not have to participate if you do not want to. If you decide to participate, but later wish to withdraw from the study, then you are free to withdraw at any time, without giving a reason and without penalty (simply close your web browser). All identifying information will be stripped from collected data once you have completed all phases of the study. If you wish to withdraw at any time during the study, all personal information will be immediately deleted.

Data Protection Privacy Notice
The data controller for this project will be Swansea University. The University Data Protection Officer provides oversight of university activities involving the processing of personal data, and can be contacted at the Vice Chancellors Office.

Your personal data will be processed for the purposes outlined in this information sheet. Standard ethical procedures will involve you providing your consent to participate in this study by ticking the consent box on the consent page.

The legal basis that we will rely on to process your personal data will be processing in line with public interest, scientific and statistical purposes.

How long will your information be held?
Anonymised data will be preserved and accessible online, as is encouraged by developments in open science.

What are your rights?
You have a right to access your personal information, to object to the processing of your personal information, to rectify, to erase, to restrict and to port your personal information. Please visit the University Data Protection webpages for further information in relation to your rights.

Any requests or objections should be made in writing to the University Data Protection Officer:-

University Compliance Officer (FOI/DP)
Vice-Chancellor's Office
Swansea University
Singleton Park
Swansea
SA2 8PP
Email: dataprotection@swansea.ac.uk

How to make a complaint
If you are unhappy with the way in which your personal data has been processed you may, in the first instance contact the University Data Protection Officer using the contact details above.

If you remain dissatisfied then you have the right to apply directly to the Information Commissioner for a decision. The Information Commissioner can be contacted at: -

Information Commissioner's Office,
Wycliffe House,

Version 4 4 November 2022

Swansea University
Prifysgol Abertawe

Water Lane,
Wilmslow,
Cheshire,
SK9 5AF
www.ico.org.uk

What if I have other questions?
If you have further questions about this study, please do not hesitate to contact us:

Tom Gordon
Research Assistant
Swansea University
tom.gordon@swansea.ac.uk

Dr. Darren Edwards
Department of Public Health, Policy, and Social Sciences.
Swansea University
d.j.edwards@swansea.ac.uk

Prof. Andrew Kemp
Department of Psychology
Swansea University
a.h.kemp@swansea.ac.uk

Version 4 4 November 2022
