# Supplementary material for: Mixed-methods feasibility outcomes for a novel ACT-based video game ‘ACTing Minds’ to support mental health
Source: BMJ Open. 2024 Mar 29;14(3):e080972. doi: 10.1136/bmjopen-2023-080972 (PMC10982759; doi:10.1136/bmjopen-2023-080972)
Supplement: Supplementary data [file bmjopen-2023-080972supp003.pdf]

Participant Consent Form

Swansea University
Prifysgol Abertawe

Project title: **A novel ACT-based video game to support mental health through embedded learning**

You must be age 18 or over to complete this online survey.

Name and Contact details of the principal researchers: Tom Gordon tom.gordon@swansea.ac.uk, Prof. Andrew Kemp a.h.kemp@swansea.ac.uk, Dr. Darren Edwards d.j.edwards@swansea.ac.uk.

This study is being conducted by Swansea University, Faculty and life sciences.

- I (the participant) consent to participate in the study
- I confirm that I have read and understand the information provided in relation to this study.
- I understand that this study will involve three phases, taking place over a period of three weeks. Within which I will complete a 1-hour therapy-based mobile phone videogame at home, that includes potentially upsetting themes relating to mental health.
- I understand that partaking in this study involves one-on-one online interviews using Zoom audio, and that any identifiable personal information will be immediately deleted following transcription.
- I understand that I have the option of undergoing electrocardiograph recording at three stages during the study, which will take place at Swansea University.
- I understand that my participation is voluntary. I understand that I am free to withdraw at any time during the study but once I have completed all phases of the study, withdrawal will not be possible because data will be completely anonymised.
- I understand what my role will be in this research, and all my questions have been answered to my satisfaction.
- I have been informed that the information I provide will be safeguarded.
- I am happy for the information I provide to be used (anonymously) in academic papers and other formal research outputs, however my name will not be published so anonymity is ensured.

- I agree to the researchers processing my personal data in accordance with the aims of the study described in the participant information.

- I am age 18 years or above.

If you agree with all statements listed above, click **YES** (I consent).

If you disagree with any of the statements above, click **NO** (I do not consent).
