# Supplementary material for: Mixed-methods feasibility outcomes for a novel ACT-based video game ‘ACTing Minds’ to support mental health
Source: BMJ Open. 2024 Mar 29;14(3):e080972. doi: 10.1136/bmjopen-2023-080972 (PMC10982759; doi:10.1136/bmjopen-2023-080972)
Supplement: Supplementary data [file bmjopen-2023-080972supp004.pdf]

DEBRIEF FORM

Title of project: **A novel ACT-based video game to support mental health through embedded learning**

Thank you for taking part in our research. Now that your contribution has finished, let us explain the rationale behind this work.

We are interested in how a video game based on recent developments in clinical psychology might enable learning of psychological resilience skills through play. Typically, psychotherapeutic interventions are delivered through face to face sessions, but there is an increasing need for psychological support that is delivered through an online medium such as the video you have been playing in our study.

This work therefore builds on previous efforts to teach people important skills in psychological resilience through psychoeducation and embedded learning. Specifically, the game is designed to build psychological flexibility, which is a key outcome of an ACT intervention. Psychological flexibility within ACT refers to the promotion of positive mental health, contact with present emotions, wellbeing, and positive emotions.
We hope that information gained from this study will be useful for further developing our ACT-based video game and expanding further research in this area.

If you feel affected by any issues raised by this research and would like to discuss any concerns, please contact the principal investigators of this study as indicated in the details provided below. If you feel that you would benefit from further psychological or psychiatric support, we advise you to contact your GP (family doctor) in the first instance. Further information on mental wellbeing can be found at https://www.mind.org.uk/. Further information regarding mental health can be found at https://www.nhs.uk/mental-health/.

Tom Gordon
Research Assistant
Swansea University
tom.gordon@swansea.ac.uk

Dr. Darren Edwards
Department of Public Health, Policy, and Social Sciences.
Swansea University
d.j.edwards@swansea.ac.uk

Prof Andrew Kemp
Department of Psychology
Swansea University
a.h.kemp@swansea.ac.uk
