# Supplementary figures and images for: Mixed-methods feasibility outcomes for a novel ACT-based video game ‘ACTing Minds’ to support mental health
Source: BMJ Open. 2024 Mar 29;14(3):e080972. doi: 10.1136/bmjopen-2023-080972 (PMC10982759; doi:10.1136/bmjopen-2023-080972)

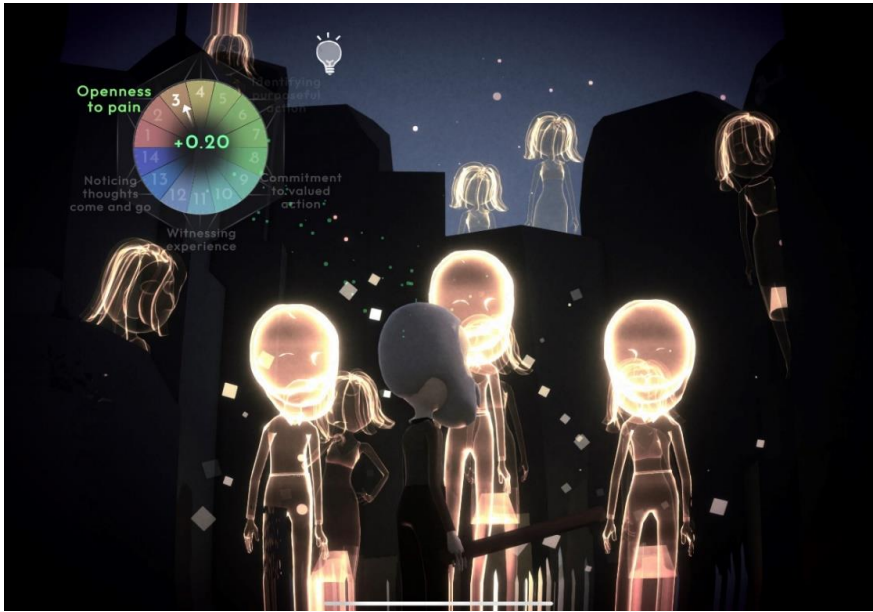

Supplement: Supplementary data [file bmjopen-2023-080972supp006.pdf]

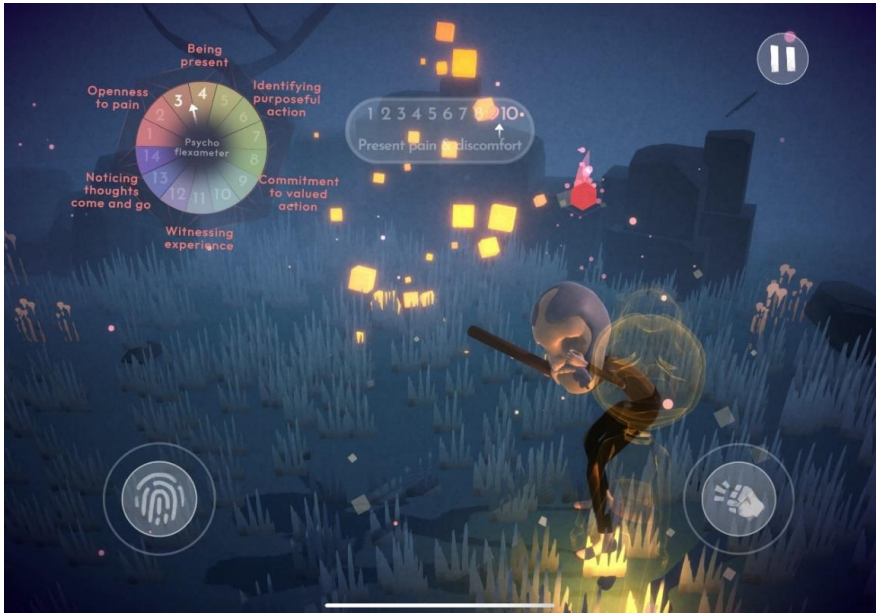

Supplement: Supplementary data [file bmjopen-2023-080972supp007.pdf]

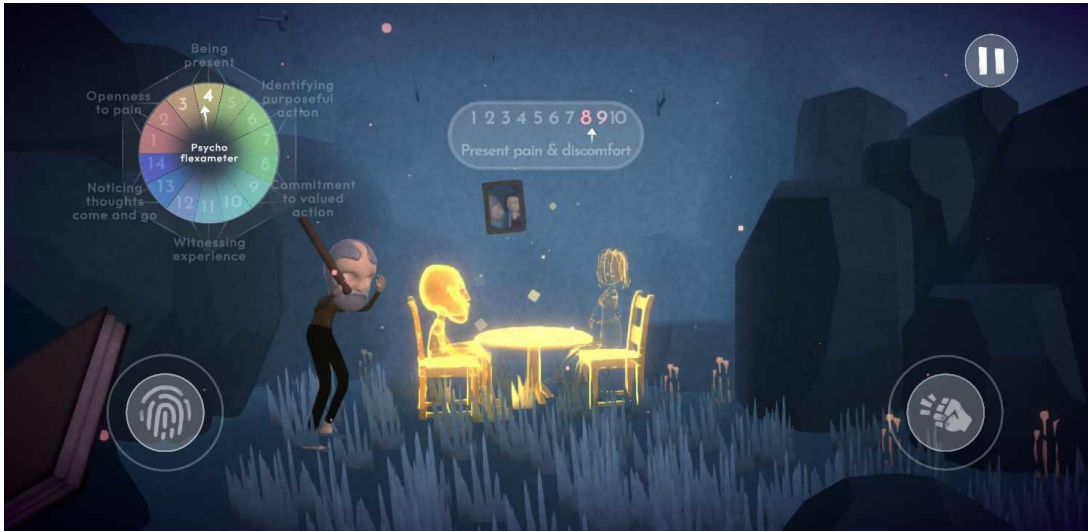

Supplement: Supplementary data [file bmjopen-2023-080972supp008.pdf]

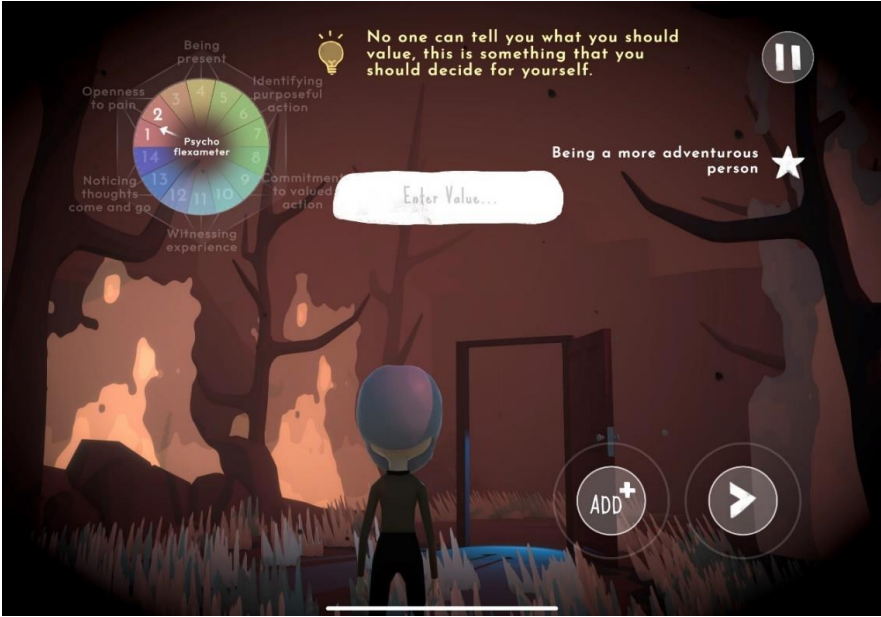

Supplement: Supplementary data [file bmjopen-2023-080972supp009.pdf]
