# Supplementary material for: Mixed-methods feasibility outcomes for a novel ACT-based video game ‘ACTing Minds’ to support mental health
Source: BMJ Open. 2024 Mar 29;14(3):e080972. doi: 10.1136/bmjopen-2023-080972 (PMC10982759; doi:10.1136/bmjopen-2023-080972)
Supplement: Author's manuscript [file bmjopen-2023-080972.draft_revisions.pdf]

BMJ Open

BMJ Open is committed to open peer review. As part of this commitment we make the peer review history of every article we publish publicly available.

When an article is published we post the peer reviewers' comments and the authors' responses online. We also post the versions of the paper that were used during peer review. These are the versions that the peer review comments apply to.

The versions of the paper that follow are the versions that were submitted during the peer review process. They are not the versions of record or the final published versions. They should not be cited or distributed as the published version of this manuscript.

BMJ Open is an open access journal and the full, final, typeset and author-corrected version of record of the manuscript is available on our site with no access controls, subscription charges or pay-per-view fees (http://bmjopen.bmj.com).

If you have any questions on BMJ Open's open peer review process please email info.bmjopen@bmj.com

BMJ Open

Mixed-method feasibility outcomes for a novel ACT-based videogame 'ACTing Minds' to support mental health

Journal:	*BMJ Open*
Manuscript ID	bmjopen-2023-080972
Article Type:	Original research
Date Submitted by the Author:	16-Oct-2023
Complete List of Authors:	Gordon, Tom; Swansea University, School of Psychology ; Swansea University<br>Kemp, Andrew; Swansea University, Psychology<br>Edwards, Darren; Swansea University, Department of Public Health
Keywords:	MENTAL HEALTH, Health Education, Depression & mood disorders < PSYCHIATRY

SCHOLARONE™
Manuscripts

I, the Submitting Author has the right to grant and does grant on behalf of all authors of the Work (as defined in the below author licence), an exclusive licence and/or a non-exclusive licence for contributions from authors who are: i) UK Crown employees; ii) where BMJ has agreed a CC-BY licence shall apply, and/or iii) in accordance with the terms applicable for US Federal Government officers or employees acting as part of their official duties; on a worldwide, perpetual, irrevocable, royalty-free basis to BMJ Publishing Group Ltd ("BMJ") its licensees and where the relevant Journal is co-owned by BMJ to the co-owners of the Journal, to publish the Work in this journal and any other BMJ products and to exploit all rights, as set out in our licence.

The Submitting Author accepts and understands that any supply made under these terms is made by BMJ to the Submitting Author unless you are acting as an employee on behalf of your employer or a postgraduate student of an affiliated institution which is paying any applicable article publishing charge ("APC") for Open Access articles. Where the Submitting Author wishes to make the Work available on an Open Access basis (and intends to pay the relevant APC), the terms of reuse of such Open Access shall be governed by a Creative Commons licence – details of these licences and which Creative Commons licence will apply to this Work are set out in our licence referred to above.

Other than as permitted in any relevant BMJ Author's Self Archiving Policies, I confirm this Work has not been accepted for publication elsewhere, is not being considered for publication elsewhere and does not duplicate material already published. I confirm all authors consent to publication of this Work and authorise the granting of this licence.

For peer review only

Mixed-method feasibility outcomes for a novel ACT-based videogame ‘ACTing Minds’ to support mental health

Tom C. Gordon[1,2], Andrew H. Kemp[2], & Darren J. Edwards[1]

[1]Department of Public Health, Swansea University, UK

[2]School of Psychology, Swansea University, UK

Darren J. Edwards,

Department of Public Health,

Swansea University,

Swansea,

SA2 8PP,

UK

Email: d.j.edwards@swansea.ac.uk

Short title: Feasibility outcomes ACTing Minds

For peer review only

Abstract

Objectives: To determine the feasibility and acceptability of 'ACTing Minds', a novel single-player adventure videogame based on acceptance and commitment therapy (ACT).

Design: A single arm, mixed-methods repeated measures feasibility study.

Setting: Intervention and questionnaires were completed at home by participants. Semi-structured interviews were also conducted at home via the Zoom platform.

Participants: Thirty-six participants were recruited into the study, 29 completed all phases of the feasibility design. Eligibility criteria required participants to be over the age of 18 and self-reporting experiencing ongoing depression, anxiety, or stress.

Intervention: Participants completed a single session of the 'ACTing Minds' videogame, lasting approximately 1-hour, designed to educate users on key principles from acceptance and commitment therapy (ACT).

Primary outcome measures: Participant recruitment and retention, questionnaire completion, long-term intervention adherence, and acceptability of the intervention. Reflexive thematic analysis was conducted on semi-structured interviews run immediately postintervention and 3-weeks later.

Secondary outcome measures: Measures of depression, anxiety, stress, psychological flexibility, social connectedness, and wellbeing were assessed at baseline, immediately following intervention completion, and at a 3-week follow-up period. Using a standardised battery of questionnaires.

Primary results: Twenty-nine participants completed the study. A reflexive thematic analysis indicated that participants responded positively to the intervention and the study at all stages. Themes reflect participants' desire for an engaging therapeutic experience, use of

For peer review only

game for exploring emotions, as well as their perspectives on how they had applied their learning to the real-world.

Secondary results: Quantitative results indicated small to large effect sizes associated with decreases in depression ($\eta p2$ = .011), anxiety ($\eta p2$ = .096), stress ($\eta p2$ = .108), and increases in psychological flexibility ($\eta p2$ = .060), social connectedness ($\eta p2$ =.021), well-being ($\eta p2$ =.011), and participation in usual activities ($\eta p2$ = .307).

Conclusions: Implementation of the 'ACTing Minds' intervention is warranted, based on both qualitative and quantitative outcomes.

Trial Registration number NCT04566042

For peer review only

Strengths and limitations off this study

- Mixed methods approach, combining thematic analysis of interviews and quantitative questionnaires.
- Collection of quantitative data at three time points and qualitative at two time points, allowing the process of change and identification of patterns to be examined.
- Remote data collection due to COVID restrictions meant that participants could not be directly observed while completing the intervention. We were also unable to record planned psychophysiological measurements of wellbeing such as heart rate variability.
- Reliance on self-report measures introduces the potential for bias.

Introduction

Global prevalence of common mental disorders and a lack of available resources for identification and treatment of those with such conditions underpins an increasing burden on

For peer review only - http://bmjopen.bmj.com/site/about/guidelines.xhtml

society (1). The Global Burden of Disease study conducted in 2017, reported a UK prevalence rate of 4.12% for depression, and 4.65% for anxiety disorders (2). Since this estimation, events such as the Covid-19 pandemic and the increasing threat of climate crises have had substantial impact on societal wellbeing, a meta-analysis including 14 studies (n=46,158) found that 32% of adults in the UK experience moderate to severe depressive symptoms in 2022, and 31% of adults reported high levels of anxiety (3), indicating a societal increase of 27.88% for depression, and 26.35% increase for anxiety. These findings require a shift toward population wide strategies focused on building resilience at scale.

In order to positively impact on societal wellbeing, it is imperative that contemporary interventions are affordable and widely accessible. Presently, the demand for mental health services exceeds the available human resources required to meet this need. A study conducted for the Centre for Mental Health estimated that services cost the UK economy approximately £105 billion per year in 2020, 4.8% of the UK's annual GDP (4). Despite significant funding of £34 billion to public mental health support and services, prevalence of psychological disorders is high and only 33% of adults with depression and anxiety receive treatment in England (5), highlighting an urgent need for innovation.

There are numerous barriers to accessing psychological interventions, including shortage of therapists, long waiting times, and societal stigma of accessing psychological treatment (6). A potential solution to these issues might be found in digital health interventions (DHI's). We live in an age of heavy digital media consumption, especially in the West in which at least 90% of UK adults use the internet regularly (7). We also know that during the COVID-19 pandemic, there were significant increases in online video consumption, social media usage, remote work, online news consumption, and video-gaming (8). COVID-19 contributed to significant social isolation and further disconnection from nature further contributing to increases in mental health conditions (9). We argue that that there is an opportunity to utilise

For peer review only

the advancements in and to harness increased use of technology to develop psycho-educational tools necessary to support mental health at scale.

DHI's have already been utilised in a variety of contexts for promoting wellbeing, from delivering healthcare and education to personalised diet and fitness plans. Mobile apps and online platforms offer guided meditation, breathing exercises, sleep tracking, and relaxation programs. Such applications aim to enhance overall wellbeing, reduce stress, improve sleep quality, and cultivate mindfulness practices (10). However, we suggest that effectively addressing wellbeing at a population level will require development of DHIs that considers acceptability, feasibility and widespread appeal.

Compared with alternative forms of media, videogame DHI's offer a number of advantages. By design they are interactive, applying behavioural principles for controlling and modifying behaviour (11), and as a result they are uniquely engaging. In the UK, the COVID-19 pandemic led to a substantial increase in the number of people playing videogames, with males increasing use from 46% to 63% and females increase their use from 32% to 56% in 2022 (12). Innovations in the use of video games for treating mental health issues have wide potential application, potentially offering a platform for individuals to explore and regulate their emotions in a controlled and supportive environment. In theory, by practicing emotional control within the game context, individuals can transfer these skills to real-life situations to better manage stress, anxiety, and mood disorders. Certain games specifically designed for therapeutic purposes, such as 'SPARX' for depression (13) or 'Elude' for anxiety (14), guide players through interactive challenges and cognitive exercises for developing emotional regulation skills. Videogames are also being used in clinical settings to promote wellbeing outcomes. One game designed for this purpose, 'Dojo' (15), aims to treat anxiety by training users in breathing techniques, muscle relaxation, positive thinking, and guided imagery, utilising heart-rate variability (HRV) biofeedback. However, when compared against a

For peer review only

standard commercial game 'Rayman 2' (control condition), a full pre-post RCT (n=1347) found that playing either game significantly reduced participant anxiety, but no differences were observed between 'Dojo' and the control condition in their ability to reduce anxiety. Reasoning for this might be that 'Dojo' failed to develop the psychoeducational skills in the participants of which it aimed to impart, or that both games only reduced anxiety by means of distracting participants from anxiety provoking thoughts (16). The researchers concluded that 'Dojo' had crucial design issues that need to be addressed, including a clear theoretical and therapeutic framework, and that research assessing the real-world effectiveness of videogames in the treatment of mental health issues requires appropriate methodology for understanding the causes of improvement.

A study aiming to explore the well-being effects of playing video games on gamers during the Covid-19 pandemic (n=781) found that time spent playing had significantly increased in 71% of participants, and 58% of participants reported that playing games had positively impacted on their wellbeing (17). The researchers conducted an online survey including closed and open-ended questions, then conducted a thematic analysis in order to identify the causes of positive improvement. Themes of escape, cognitive stimulation, stress relief, agency, and socialisation were most associated with feelings that playing videogames had increased wellbeing. The development of an effective DHI videogame should take such factors into consideration whilst also building on strong theoretical and therapeutic foundations that facilitate the uptake of such tools.

The 'ACTing Minds' videogame, developed in line with our protocol intervention (16), was designed to be a comprehensive transdiagnostic intervention that will integrate lessons from acceptance and commitment therapy (ACT) (18). ACT is a third wave behavioural therapy, which prioritise the holistic promotion of psychological and behavioural processes associated with health and wellbeing, rather than focusing on the elimination of negative mental health

For peer review only - http://bmjopen.bmj.com/site/about/guidelines.xhtml

symptoms (19). More specifically, an ACT based DHI videogame will be a comprehensive approach for managing common mental health issues such as depression and anxiety by developing explicit psychoeducational skills that promote psychological flexibility, wellbeing, and resilience. As a consequence, our videogame may have greater reach and impact than other videogame DHI's not based on third wave psychotherapy, such as 'Dojo', which primarily aims to teach skills for emotional regulation and symptom reduction.

The ACT framework is well-suited for integration into videogame development because it does not necessitate formal clinical training or accreditation in order to be applied effectively (20), and resources for education and practice are freely available through the Association of Contextual Behavioural Science website[1]. A review of 20 meta-analyses, involving 133 studies (n=12,477) found that ACT was efficacious for treating anxiety, depression, chronic pain, substance use, eating disorders, and for improving quality of life. The results also showed that ACT was generally superior to most active intervention conditions (excluding CBT), treatment as usual, and inactive controls (21). ACT has also been effectively implemented in DHI's in the form of mobile phone applications, which are shown to be effective in promoting psychological flexibility (22) and reducing smoking intake(23). Considering this, we believe that choosing ACT as the basis for our game will allow us to harness the advantages of third-wave therapies as transdiagnostic therapeutic tools and integrate these with those of videogames, and if made well, will be engaging, educational, and capable of promoting wellbeing at scale.

ACT aims to decrease suffering and increase wellbeing through six core processes of change (24). These are: (1) present moment awareness: the practice of being in the here and now; (2) acceptance: the practice of being open to the range of human emotional experience, as

[1] See Association of Contextual Behavioural Science Website: https://contectualscience.org/

For peer review only - http://bmjopen.bmj.com/site/about/guidelines.xhtml

opposed to experiential avoidance; (3) Defusion: the act of recognising the self as separate from thoughts, and not interpreting them literally; (4) values: identifying ones' personal values in contrast to perceived expectations, of which drive us toward self-direction and purpose; (5) action: a commitment to ones' personal values, facilitating the development of competence through the act of continual practice of alignment with values (25); (6) self as context: developing an awareness of self that is more than a conceptualised sense of self, one that is flexible and facilitates a sense of connection with others.

Based on our initial protocol (16)[2], the ACT-based videogame called 'ACTing Mind', has been developed as a psychoeducational tool that teaches users the core processes of ACT through embedded learning. The goal of this research will be to determine the acceptability and feasibility of 'ACTing Minds' for reducing mental distress. The game teaches skills based around the ACT principles of acceptance, defusion, and commitment to values. This will be a feasibility study, following the Medical Research Council (MRC) framework (26), laying the foundation for a full-scale RCT on which clinical effectiveness will be determined.

There have been several changes made to our originally published protocol (16), it was initially stated that participants would complete five weekly 1-hour sessions where they would play through six parts of the 'ACTing Minds' videogame, each one focusing on a different process of ACT. However, because of funding restrictions, 'ACTing Minds' has been compressed into a single game focusing on the ACT principles of acceptance, commitment to values, and defusion. Therefore, in this feasibility study participants will be required to complete a single 1-hour session of 'ACTing Minds'. This meant a significant change to the overall time to complete the study protocol. Originally it was expected to take 3 months between baseline measurements and the final follow-up. Now, one-on-one semi-

[2] See Supplementary 1 for Protocol Paper

structured interviews will be conducted immediately postintervention, and after a 3-week follow-up. The research questions are as follows: Is the intervention acceptable and feasible for a full-scale RCT? Is there early evidence for effectiveness in reducing mental distress? Are there any changes in self-reported wellbeing measures following completion of the game? Are participants able to learn ACT principles and apply them in day-to-day life?

Methodology

Design:

This is a single arm, mixed-method repeated measures study, designed to determine the feasibility and acceptability of an ACT-based video game called 'ACTing Minds' that has been designed for individuals reporting mild to moderate anxiety, depression and stress. Data was collected at baseline, immediately post-intervention, and 3-weeks post-intervention Data was collected between November 1st and December 31st, 2022.

Study Setting:

The study was conducted entirely online by participants, including intervention (via a working prototype of the app; https://shorturl.at/iqFGI), quantitative assessment (via the Qualtrics platform), and qualitative interviews (via the Zoom platform). Strict recommendations were given to participants to ensure they are in a quiet room and without disruption for all study components.

Participants:

Thirty-six participants were recruited, 29 of which completed all phases of the study. Participants were recruited using purposive sampling methods, focused on recruiting participants at least 18 years of age, those who self-reported ongoing experience of mild to moderate depression, anxiety, or stress within their day-to-day life, as well as those able to

For peer review only - http://bmjopen.bmj.com/site/about/guidelines.xhtml

read, write and speak English. Sample size was justified on the basis of past research reporting the median numbers of participants recruited for similar feasibility studies, incorporating both quantitative and qualitative elements (27). Advertisements were posted at Swansea university and on social media pages. Participants were recruited between October 1st and December 1st, 2022, they completed a consent form[3] after reading the study information sheet[4], and were given a debrief sheet[5] following completion of the study, each of which are included as supplementary materials.

Primary outcome measures:

Feasibility outcomes were determined using the MRC framework (26) and reported in line with CONSORT guidelines (28)[6]. Feasibility measures included data relating to participant recruitment and retention including number of participants willing to take part, and completing each stage of the study (i.e., intervention, questionnaires, interviews, and follow-up), as well as acceptability, assessed through thematic analysis of semi-structured interviews conducted immediately post-intervention and 3-weeks post-intervention, with interview question topics including but not limited to: barriers for adoption of the intervention; ACT adherence in daily life; participants experience with the intervention; and whether they would be willing to complete further acts of 'ACTing Minds' once they have been developed and released.

Secondary outcome measures:

Questionnaires were distributed at three points in time (baseline, immediate postintervention and 3-month follow-up). A rule was created in Qualtrics requiring participants to complete

[3] See Supplementary 2 for Participant Information Sheet
[4] See Supplementary 3 for Consent Form
[5] See Supplementary 4 for Debrief Form
[6] See Supplementary 5 for CONSORT Checklist

For peer review only - http://bmjopen.bmj.com/site/about/guidelines.xhtml

every questionnaire item in order to finish the survey which included the following questionnaires:

Depression Anxiety Stress Scales (DASS-21): a measure of general psychological distress with good construct validity (confirmatory factor analysis of 0.94), that can be broken down into subscales relating to stress, anxiety, and depression. It has good internal reliability as measured through Cronbach's alpha coefficients, which are 0.88 for depression, 0.82 for anxiety, 0.90 for stress, and 0.93 for the total scale (29).

Acceptance and Action Questionnaire-second version (AAQ-II): a questionnaire of seven items, assessing psychological inflexibility by gauging one's capacity to embrace and remain receptive to challenging thoughts and emotions, while also actively participating in meaningful actions despite their presence. A higher score indicates higher psychological inflexibility. The measure has good construct validity with a Cronbach's alpha coefficient of 0.84 (30).

Social connectedness (adapted from Russell's (1996) UCLA Loneliness Scale (31): this measure involves two questions: (1) "During social interactions, I feel 'in tune' with the person/s around me", and (2) "During social interactions, I feel close to the person/s". The Cronbach's alpha coefficients for these two items ranged from 0.80 to 0.98 (M=0.94, SD=0.03) (32).

Warwick-Edinburgh Mental Well-Being Scale (WEMWBS): a metric that emphasises the positive facets of mental health, aiming to evaluate overall psychological well-being. This measure has good internal consistency with a Cronbach's alpha coefficient of 0.89 (student sample) and 0.91 (general population sample) (33).

For peer review only

EuroQol five dimensions (EQ5D) (34): a measure for health-related quality of life. There are five components within this measure which assess mobility, self-care, usual activities, pain, discomfort, anxiety, and a visual analogue scale for measuring current health status.

Intervention:

The intervention comprised of an ACT-based video game intervention called 'ACTing Minds', developed and designed by author DJE. Participants attended a single session lasting approximately 1-hour, during which they completed four in-game chapters.

The intervention teaches three core principles pertaining to ACT through embedded learning, meaning that the player will gain ACT-based skills while completing in-game objectives, without being directly teaching those skills. These skills include 'Acceptance', 'Cognitive Defusion', and 'Commitment to Values'. Embedded learning refers to the incorporation of educational elements into the gameplay itself, in contrast to explicit lessons. In this context, it involves designing the game in a way that promotes positive health-related behaviours derived from ACT, such that adoption of ACT processes by participants results in positive long-term quality of life changes. In 'ACTing Minds,' an example of embedded learning is the 'Psychoflexameter' (see Figure 1A), which serves as a gamified version of the Hexaflex (see Figure 1B), a model used in ACT to illustrate both the theory and goals for clinical change (36). Similar to the Hexaflex, the 'Psychoflexameter' showcases the 6 core processes of ACT. Initially introduced to players during the first ACT-oriented activity in the game, which emphasises acceptance, the 'Psychoflexameter' remains visible in the corner of the screen throughout the game while players engage in other ACT-related activities. As players exhibit ACT-consistent behaviours, they earn points and gradually increase the dial on the centre of the 'Psychoflexameter.', and the text reflecting the ACT process improved lights up green[7]. If

[7] See Supplementary 6 for in-game screenshot illustration

For peer review only - http://bmjopen.bmj.com/site/about/guidelines.xhtml

players exhibit ACT-inconsistent behaviours, they lose points and the 'Psychoflexameter' lights up red[8].

--**Figure 1 Here**--

The game starts with a text-based chapter, telling the story of a depressed individual, 'Steve', who has recently lost his wife in an accident, he is feeling depressed, isolated, and lonely. The character has built a 'mind escape machine' with the intention of entering his own mind in order to destroy and suppress his unwanted painful thoughts and memories. The player takes control of 'Steve' in chapter two, where they see him in a state of mental distress in his home, surrounded by items that are reminders of his lost wife. At this stage participants will learn how to control the character using an onscreen directional stick and interact with the environment by touching key elements with their finger.

Participants engaged with ACT content within chapters three and four, which begin with the character entering his mind, walking around and viewing representations of his memories. Chapter three focuses on 'Acceptance', introducing players to a bar in the centre of the screen indicating the characters' present level of distress, as well as the 'Psychoflexameter' dial in the corner of the screen, indicating the characters' psychological flexibility. While in the mind of the character, the player is able to approach memories of 'Steve' and his wife, which leads to an increase in present distress and allows the option to destroy the memories. Destroying memories decreases present distress, but also removes points from the 'Psychoflexameter'. If the player chooses to destroy the memories (avoidance-based strategies), the world becomes increasingly distorted, and barriers form making the chapter

[8] See Supplementary 7 for in-game screenshot illustration

For peer review only - http://bmjopen.bmj.com/site/about/guidelines.xhtml

impossible to complete. Alternatively, if the player chooses acceptance-based strategies they can continue the game and learn that acceptance is functionally better than avoidance[9].

Chapter four focuses on rewarding ‘Commitment to Values’ and ‘Cognitive Defusion’. The player is still in the mind of the character, where they are then asked to reflect on their values, to type them out and make them explicit[10]. Following this, they complete a ‘leaves on a stream’ task, requiring them to type out any negative thoughts that they might have and place them on a leaf, watching them as they float downstream (see Figure 1C). Both tasks reward the player by decreasing present distress and increasing their score on the ‘Psychoflexameter’.

Qualitative analysis:

Here we employed a critical realist ontological framework to interrogate the underlying nature of the phenomena under study. Concurrently, a contextualist epistemological stance was adopted to facilitate a nuanced understanding of knowledge construction. These philosophical underpinnings guided the application of reflexive thematic analysis, serving as a methodological lens for identifying and interpreting emergent themes within the data. The analytical process commenced with an inductive mode of inquiry, allowing for data-driven insights to emerge organically. As the analysis progressed, there was a methodological pivot towards a more deductive approach, guided by pre-existing theoretical frameworks. The coding schema evolved correspondingly, transitioning from a focus on explicit, semantic content to an exploration of underlying, latent themes. Two semi-structured interviews (post-intervention, and 3-week follow-up) formed the foundation for two sequential thematic analyses, following the guidelines outlined by Braun and Clarke (35). Both interviews were analysed separately to gain an understanding of differences in participant perceptions of the

[9] See Supplementary 8 for in-game screenshot illustration
[10] See Supplementary 9 for in-game screenshot illustration

For peer review only

intervention and relevant outcomes over time. For reporting on the acceptability of the 'ACTing Minds' intervention, findings and themes from both interviews are summarised in the primary outcomes section of the paper (see Table 1).

Interview data were transcribed using Microsoft's automated audio-to-text software, which was then double checked and edited to correct for major spelling or grammatical errors. Throughout the data familiarisation phase, multiple points of potential analytical interest were identified within both interview sets. In the coding phase, several hundred codes were initially produced, which were then clustered to make them more manageable and categorised into potential broad patterns of meaning. For the analysis of the first interview set these included: emotional experience; wellbeing needs, perceptions on mental-health education within the game; and participant engagement. For the second set of interviews these were: application of the games' lessons, perceptions on what was learned, desire for growth, and sense of development.

Themes were then refined in the context of our research questions relating to how the participants experienced 'ACTing Minds', which involved a review of preliminary themes in relation to the codes, the coded data, and the full dataset. We became most interested in the latent ideas underpinning statements relating to how participants utilised the game as a psychoeducational tool. The preliminary theme 'emotional experience' was developed, as it was interpreted from the codes that participants were using the game as a 'base for exploring emotions.' From there many of the remaining codes fell easier into place, allowing for further development of themes that emphasise the processes involved in participant engagement, personal therapeutic goals, and feelings regarding the games' embedded learning features. Themes developed from the second interview codes tended to reflect the participant outcomes since playing 'ACTing Minds', allowing for exploration of specific vectors of growth, how

For peer review only

For peer review only - http://bmjopen.bmj.com/site/about/guidelines.xhtml

the participants applied their takeaways from the game in the weeks following playing, and what they felt they had learned as a consequence of applying those takeaways in real-life.

Table 1.
Themes and subthemes taken from thematic analysis for interviews 1 and 2.

Interview 1 (Immediate postintervention)	Interview 2 (3-weeks postintervention)
Theme 1: Desire for an engaging therapeutic experience<br>Theme 2: Personal process of immersion<br>Theme 3: Game as a base for exploring emotions<br>Theme 4: Embedded learning pros and cons<br>Theme 5: Necessary learning for anyone	Theme 6: Utility in the real world<br>Theme 7: Practice facilitates wellbeing<br>Theme 8: Closer to the self

Quantitative analysis

A repeated measures ANOVA was performed using IBM SPSS Statistics 29 (the most up-to-date version at the time of analysis), to compare the effects of playing the 'ACTing Minds' videogame on scores taken from the questionnaires DASS-21, AAQ-II Psychological flexibility questionnaire, Warwick-Edinburgh Mental Well-Being Scale, Social connectedness score, and EuroQol five dimensions.

Descriptive statistics and corresponding effect sizes were used to summarise secondary outcome measures (see Table 2). Changes in scores from baseline are reported for each of the measurement timepoints. Partial eta squared ($\eta p2$) effect sizes were calculated for each independent variable and interpretation was informed by prior literature on the topic (36). Values 0.14 or higher were interpreted to be a large effect, 0.06 – 0.14 were interpreted to be a moderate effect, and 0.01 – 0.06 were interpreted to be a small effect.

For peer review only

Results

Primary outcome measures

Participant recruitment and retention (feasibility)

Thirty-six participants were recruited through the initial study advertisement between October 1st and December 1st, all of which met eligibility criteria. Six participants did not show up for initial baseline measures, while 1 participant did not follow through with the intervention (see Figure 2).

---**Figure 2 Here**---

Participant feedback (acceptability)

Acceptability measures were assessed through thematic analysis of semi-structured interviews, taken place immediately postintervention and at a 3-week follow-up. The interviews were analysed separately in order to understand participant perceptions before and after applying lessons learned from the game to their everyday lives. The results from both have been summarised and reported together as key themes.

Theme 1: Desire for an engaging therapeutic experience

Participants regularly expressed interest in the novelty and potential utility of digital mental health applications, more specifically in a gamified context. Overall, they felt that a videogame platform provided a uniquely stimulating means of engaging with mental health learning. This contrasts with feelings expressed towards more typically used wellbeing applications, which were considered to have potential benefit but are regularly seen as a chore. Participants suggested that psychoeducational tools like 'ACTing minds' may be able to function as an alternative, or at least as an adjunct to therapy.

For peer review only - http://bmjopen.bmj.com/site/about/guidelines.xhtml

"I don't really think I've ever played a game that's based on emotions [...] as a gamer I don't really do all these other things, like writing in books or doing all this other stuff, but like playing a game that's based on that for your mental health thing is really good because you can actually like do something therapeutical in the things that I like doing." -P06.

This quote comes from a participant who identifies as a gamer, with little experience or prior interest in mental-health learning. There is a sense that this relates to the participant having no exposure to such education within their real-life, and that there is an underlying desire for personal growth if it were to be made more comfortably accessible.

"I'm not one to sit down and just watch a video and then put that in practise. I'm someone who actually needs to be involved in something and interact with it and course work just reminds me too much of school [...] with games it makes me sit, actually interact because I'm enjoying the game at the same time. It's not just that I need to focus on my mental health. I get to play a cool game while learning about my mental health. So yeah, I like I like video games with mental health stuff." -P08

This quotation contrasts with the first in that the participant is not a gamer but has substantial experience with mental health learning and therapy. Despite this, they express the same desire for more engaging therapeutic experiences. The participant expresses an aversion to learning in a way that feels like school, which may reflect their poor experiences with traditional therapies like CBT. Another idea expressed is that playing a mental health game removes feelings of seriousness, or that it must be done out of necessity, facilitating a more relaxed approach to learning.

Theme 2: Personal process of immersion

Responses from the semi-structured interviews painted a picture of the individual differences between participants in their ability to immerse themselves within the content of 'ACTing

For peer review only - http://bmjopen.bmj.com/site/about/guidelines.xhtml

Minds'. They generally expressed a prior interest in mindfulness or growth, which appeared to be a driving motivational factor for engaging with the games' activities and story. Along with this, participants had a vague awareness of the therapeutic intent behind the videogame, which was explicitly stated to motivate their engagement.

"It plays a very good way of dealing with visual metaphor, but also the fact that you're given the agency to do it. When you were smashing the memories and I wish I didn't have to, but I have to carry on Steve's journey and I think it's a really good way of showing like yeah, these memories do hurt and sometimes you just have to accept the fact that it's going to hurt. Move on like it's explained through the visual and like narrative storytelling, and I think it's a really good way to do it." -P18

This participant statement provides a window into their own process of immersion, in which from early into the game, the participant perceives a conflict between what they think is the right action (choosing not to destroy memories) versus the action they think they need to complete (destroying memories) in order for the story to progress. The participant chooses to understand this metaphorically, framing it as supporting the growth of 'Steve', but vicariously growing in the process. The requirement for participant agency in making emotionally difficult decisions for the main character 'Steve' appears to have facilitated immersion into the games' story and engagement with the lessons embedded. The participant relates to 'Steve', saying "yeah, these memories do hurt…", which likely reflects perspective taking, a cognitive process central to ACT practice, involving the ability to understand and consider another persons' experience, which is not a core focus within 'ACTing Minds' but appears to relate to the uptake of the other ACT oriented lessons.

"It sort of grounds it back to you because it's all very well playing the game as Steve and sort of doing Steve's memories and things, you sort of might forget that. So it's been like a

For peer review only - http://bmjopen.bmj.com/site/about/guidelines.xhtml

learning experience, and so the sort of personalised bit at the end with like the lily pads and the values. I think it was quite good because then you actually were able to think about like the purpose of the game within your own situation." -P10

This participants' statement puts significance on the more personalised sections of the game for cementing the lessons that they might have picked up during the earlier sections. This contrasts with the prior quotation, as the participants' process of immersion is less reliant on relating to the main character of the story, but instead centres on the tasks within the game that required them to input their own values, and their own negative thoughts.

Theme 3: Game as a base for exploring emotions

Participants regularly invoked the idea that the game provided a platform that allowed them to explore their own emotions. They noted the importance of the games' art-style and music in setting a calm environment, from which they were able to engage with the more emotionally intensive elements of the game and story. Participants reported experiencing a variety of negative emotions throughout the game, including sadness, anxiety, and grief, but also felt after playing that there would be long-term benefit to them observing and accepting those feelings.

"I would download it if I was feeling like I really needed it at the time. Although it was engaging, it's not necessarily something I would just download and play for fun, like if I was feeling particularly depressed or unhappy or anxious or whatever. I would genuinely use it as a tool to sort of try and organise my thoughts and do what the game is trying to sort of simulate doing of getting over something and helping yourself to understand something. But yes, I would sort of use it as a reactionary tool, but I think that's just my own fecklessness." - P07

For peer review only - http://bmjopen.bmj.com/site/about/guidelines.xhtml

The participants' statement that they would not replay the game just for enjoyment puts emphasis on the distinction between this game as a psychoeducational tool and recreational videogames. This is further highlighted in their perception of the game as simulating an experience of emotional growth and understanding. Interestingly, this participant does not suggest that they learned anything new through the program, but instead expresses a sense of value for the utility of the game as a tool for mental organisation.

"I think it was sad thinking about how the relationship crumbled within the story. I mean it's quite intense you know. As someone who is feeling those emotions crying out at the moment. But yeah, I think lots of those things could trigger stuff for different people. But you know, there's a lot to be said about if that's a bad thing." -P02

This quotation illustrates the intensity of negative emotion experienced by the participant during their playthrough of 'ACTing Minds'. They acknowledge that their reaction is to specific aspects of the games' story that resonate with them personally, and that others might have stronger reactions to different aspects of the game. The participant states "There's a lot to be said about if that's a bad thing", which whilst being ambiguous to whether or not the game is beneficial, seems to put responsibility on the player to engage with the games content in order to get the benefit. The game may be able to function as a base for emotional exploration, but it cannot do the exploration for the player.

Theme 4: Embedded learning pros and cons

Data from the immediate post-intervention interviews suggest a mixed response to embedded learning in 'ACTing Minds'. Many participants reported learning a variety of lessons through playing the game, most being intended in the games' design, as well as some unintended but positive takeaways. Participants generally felt positively about the embedded learning approach, expressing that allowing them to work things out for themselves provided a deeper

For peer review only - http://bmjopen.bmj.com/site/about/guidelines.xhtml

level of learning than being directly taught. A few however, felt that they did not learn anything through playing, or that they did not feel that they could apply the lessons from the game to real-life. It was noted that some participants did feel that there was vague learning, but that it would be ineffective without more explicit information on the core ACT principles present in the game, or how to actively apply them.

"So when you hit them [memories], the like walls go up right, yeah? Honestly, like I think that at this part of the game I was just confused. I didn't really know what I was or wasn't supposed to do. I think after wondering a bit, I was a bit confused, like what do I do next in the game? But I guess it was like kind of showing you that if you hit the memories, you kind of close yourself off and that's what the walls were." -P14

Participants often reported some confusion with completing the games' tasks. This is likely a consequence of the embedded learning approach, as by design players are expected to learn the ACT oriented lessons by figuring out how to progress through the game. The prior quotation illustrates how despite confusion, the participant in hindsight had the ACT-consistent takeaway that choosing to not accept memories closes you off to progress.

"Obviously, like for the character Steve, maybe that wasn't immediately clear when he went in [that destroying memories made progression more difficult], so even the fact that I didn't get that straight away and I went in destroying things, I didn't feel like I was at odds with the game. Like after playing it for a couple of minutes like I realised. Yeah, I suppose he did too then." -P29

Some participants understood their confusion with the games objectives as being reflective of the main characters mental state. This participant notes that whilst they experienced uncertainty with whether or not to destroy memories in the acceptance section of the game due to already feeling that it was a poor choice, they did still destroy memories with the belief

For peer review only - http://bmjopen.bmj.com/site/about/guidelines.xhtml

that the behaviour was important for the main characters journey. Providing further evidence of the effectiveness of perspective taking in helping participants to properly engage with the ACT lessons embedded within the game. Where other participants might have become frustrated due to being confused with the games' objectives, this participant was seemingly able to extend their empathy for Steve to their self.

"I just think that like. That's not enough for someone. I mean, they [the game designers] might get someone thinking about it, like it might make someone aware, but to actually like, maybe solidify into someone's life like you have to have kind of a discussion about it. So I wouldn't say they [the lessons in the game] were too abstract. I just think again for it to like fully land that like discussion afterward might be necessary." -P09

This quotation is representative of a small number of the participants in this study, who felt that the game was successful in priming them to consider their own wellbeing and how they might act to improve it. But also felt that the information in the game was not thorough enough to provide them with a sense of explicit learning. This information is valuable to future development of similar interventions that might benefit from including links to further learning.

Theme 5: Necessary learning for anyone

Participants widely expressed that they were able to gain skills that they felt would be applicable to their own lives after playing 'ACTing Minds'. The ACT processes reported as being most personally relevant varied between participants. However, acceptance, defusion, and commitment to values were brought up in fairly equal proportions. Participants felt that even if they already knew the lessons being taught, that the game was effective in reaffirming those beliefs, making the wellbeing concepts more tangible, and priming them to consider wellbeing concepts more in their day-to-day lives. There was a notion across participants that

For peer review only - http://bmjopen.bmj.com/site/about/guidelines.xhtml

the lessons taught in the game are essential for everyone, and that further development of the technology will have large societal benefit.

"I actually learned that it's good to let your feelings go. You can't just hide from things that have happened or just forget they've happened. You've gotta deal with them. You've gotta find a way that works for you to let things go." -P16

This participant felt that they struggled with holding on to negative feelings prior to playing 'ACTting Minds'. Their statement implies that they would typically respond to this problem with self-distraction, but that they have realised it to be an ineffective strategy in comparison to acceptance. Whilst it is uncertain whether this participant had learned how to integrate the lesson of acceptance into their own life, their statement "You've gotta find a way that works for you to let things go" illustrates their belief that there is value in such lessons for everyone.

"Some people I know struggle a lot. Because I don't really find it difficult to not hold on [to negative thoughts and feelings], but I know a lot of people really do fixate on something, and you know, they just can't get over it, but I can see it [ACTing Minds] being very useful for other people's day-to-day." -P04

Interestingly, despite this participant feeling that they do not find it difficult to avoid fixating on negative emotions, repeats the notion that the lessons taught in 'ACTing Minds' are of significant value to others. There is a sense that psychological wellbeing skills like acceptance and defusion should be developed in everyone at some stage in their lives.

Theme 6: Utility in the real world

In the interview occurring 3-weeks postintervention, participants discussed how they were able to apply the lessons that they took from 'ACTing Minds' into the real-world. Whilst a few participants had not considered the game since playing or tried to actively apply lessons, many found that they made a conscious effort to apply ACT principles and techniques from

For peer review only - http://bmjopen.bmj.com/site/about/guidelines.xhtml

the game, or that situations in real life prompted them to consider and apply the core concepts, each of which were mentioned by participants (i.e. Acceptance; Defusion; and Commitment to Values). Some also found that they had an easier time helping others through emotional challenges by sharing what they had learned from the game.

"I have noticed I've actively now, if I get like a negative thought in my brain. I try and register it and I don't hold on. But like, because sometimes before, even though I kind of subconsciously did it with some things, I just didn't [always], you know. But now if it's even something stupid like I've been lazy. I was like alright don't think about it all day. Actually go out for a walk. Go to the gym you know. Don't just keep in your head, I'm so lazy and miserable and fat. Get out to do something about it." -P04

This statement was given by a participant who in the immediate-postintervention interview felt that they did not have a problem with holding on to negative thoughts. Despite this, in the weeks since playing 'ACTing Minds', the participant found that they were more conscious of how their thoughts have impacted on their real life, and as a result has been able to apply the core ACT concepts in tandem. Their statement indicates that they were able to apply defusion lessons to negative thoughts about their self ("I'm so lazy and miserable and fat"), acceptance of their own behaviours ("I've been lazy"), and commitment to values in taking part in positive wellbeing activities regardless of their negative thoughts.

"I wouldn't necessarily say I've sat there and primed those thoughts [regarding ACT concepts], but in like unconscious thinking, if you know like passive thought and stuff like that in my day-to-day, I've definitely had hints of some of those topics. Do you know what I mean? Like even today, I was going about my day doing my thing and you know you'll have a thought that'll throw you back to the past, and then you learn like I came to accept it." -P11

For peer review only - http://bmjopen.bmj.com/site/about/guidelines.xhtml

Many participants noted that even if they had not explicitly tried to apply the ACT principles learned from the game to real life, that they felt the game had influenced them subconsciously, and that they considered those topics more readily when real-life situations demanded them. In these cases participants most commonly felt that they were passively more accepting of difficult emotions and situations.

Theme 7: Practice facilitates wellbeing

A core theme present across follow-up interviews was that the real learning occurred during application of ACT concepts in the real-world or during discussions regarding the game, as opposed to during their playthrough. Participants felt that they gained a deeper understanding of why the ACT concepts are useful since playing, which facilitated a shift in thinking and a sense that learning them had a subconscious influence over their actions. Several positive outcomes were reported as a result of practice, these being: a more positive mindset; a new interest in personal mental health; better able to take a step back; and reduced reactivity.

"Well, it's like actually testing on a real situation as to just generally learning it. But then when you come into a situation, you start to understand a bit better why you do those things [referring to ACT skills] and what benefit those things have, because the situation is actually impacting on your emotions or your feelings and stuff like that, and so then you're like ohh, this is why this is a good technique." -P05

In practicing real life application of ACT principles, participants were able to get a deeper sense of how and why the techniques worked for them personally. The prior statement implies that these increases in understanding occur as moments of insight during emotionally challenging situations, providing a general sense of relief and growth.

"Yeah, I'd say, uh. I've learned ways to engage with my thoughts, and like I've always tried to practise letting go of things that aren't like too meaningful, like things that won't matter in a

For peer review only

day and all that sort of stuff. But I feel like the game has helped me also realise the opposite, like a way to really put my values down in a more straightforward manner." -P13

This statement gives an outlook on how practice of some ACT principles, might lead to insights that involve others. By actively observing thoughts and through practice of disregarding thoughts that did not strike them as meaningful, they were able to get a clearer sense of what is meaningful to them (their values), which facilitated living in a way that prioritises those values.

Theme 8: Closer to the self

One of the most consistent patterns across interview responses was that participants felt they had learned more about themselves, or that practicing the lessons taught in the game brought them closer to their selves. For some, this was in becoming more aware of their personal values, or that playing the game had revitalised a sense of mindfulness and awareness of their feelings. Participants also discussed becoming closer to their self through practice of acceptance, which was seemingly more the case for those who applied acceptance internally to their own thoughts and emotions, compared to those that focussed on accepting external situations.

"Since playing the game I've downloaded a few different things with visibility and mental ability and stuff. And taking a few online tests and stuff and just to know, just down to interest. But I wouldn't have done that if I hadn't played the game." -P16

Many of the participants since playing the game felt that they had a renewed interest in personal wellbeing, which from interview data appears to have a circular relationship with a desire to learn about the self. Such that increase in one appears to correspond to an increase of the other. The prior quotation illustrates that since playing the game, this participant has

For peer review only - http://bmjopen.bmj.com/site/about/guidelines.xhtml

explored various other avenues of self-learning, believing it to be a direct consequence of playing 'ACTing Minds'.

"I just feel like it actually brought something into my life that is happening, if you're saying that about the practises, then of course I cannot be like, [these are the] step-by-step stages, but I actually learned a lot from this game about like these inner emotions or bad memories are not sinful. They're a part of you, and they contribute what you're going to be or the current you. So yeah, that's the core lessons I guess I learned from the game." -P23

In discussing their feelings on what they learned, this participant articulates a point that is expressed across many of the other participants, that the growth experienced from playing 'ACTing Minds' is intangible. Understanding the growth as a collection of processes in motion that cannot be broken down into steps and easily captured in language. Expanding on this, the participant states they have learned that internal feelings and bad memories are not sinful, implying that before playing the game they held the belief that some parts of their internal experience could be inherently wrong. This contrasts substantially with the belief they now hold, that those negative internal experiences should be accepted as part of the self.

Secondary outcome measures

A large effect size was found for the EQ5D usual activities score ($\eta p2 = .307$), while medium effect sizes were found for DASS-21 Stress scores ($\eta p2 = .108$), DASS-21 Anxiety scores ($\eta p2 = .096$), and AAQII Psychological Flexibility scores ($\eta p2 = .060$). Small effect sizes were obtained for the DASS-21 Depression scores ($\eta p2 = .011$), the WEMWBS ($\eta p2 =.011$), UCLA Social Connectedness scale ($\eta p2 =.021$), EQ5D Pain/Discomfort ($\eta p2 =.010$), and the EQ5D Anxiety/Depression ($\eta p2 = .018$). While no significant effects were observed, the observed large and moderate effect sizes provide preliminary evidence of beneficial impacts (see Table 2).

For peer review only - http://bmjopen.bmj.com/site/about/guidelines.xhtml

Convergent outcomes

Integration of results is considered a defining feature of mixed-methods research (37). In the interest of inferential transparency, we have included a visualisation of the research outcomes taken from our qualitative and quantitative approaches, illustrating how each outcome links back to feasibility, as well as the conclusions made following each outcome (see Figure 3).

For peer review only

For peer review only - http://bmjopen.bmj.com/site/about/guidelines.xhtml

Table 2.
Illustrating change in intervention outcomes over time (n=29)

	**Preintervention Baseline, mean (SD)**	**Postintervention, mean (SD)**	**Follow-up (3 weeks), mean (SD)**	**ηp2**	**Effect size**
DASS-21 Depression	14.34 (5.97)	14.21 (5.54)	13.90 (4.75)	.011	Small
DASS-21 Stress	15.34 (4.55)	14.52 (4.40)	14.28 (4.65)	.108	Medium
DASS-21 Anxiety	12.79 (5.19)	12.10 (4.42)	11.66 (4.75)	.096	Medium
AAQII (Psychological Flexibility)	27.34 (10.47)	28.38 (9.86)	28.86 (8.65)	.060	Medium
WEMWBS	42.07 (7.31)	42.62 (7.91)	42.07 (7.00)	.011	Small
UCLA Social-Connectedness	64.72 (7.52)	64.24 (8.85)	63.90 (8.33)	.021	Small
EQ5D Mobility	4.83 (.38)	4.83 (.38)	4.83 (.38)	.000	None
EQ5D Self-Care	4.78 (.58)	4.79 (.49)	4.76 (.51)	.000	None
EQ5D Usual Activities	4.14 (.79)	4.38 (.68)	4.62 (.68)	.307	Large
EQ5D Pain/Discomfort	4.24 (.64)	4.45 (.63)	4.31 (.81)	.010	Small
EQ5D Anxiety/ Depression	3.86 (.99)	4.03 (.87)	3.97 (.94)	.018	Small
EQ5D Self-rated health score	71.14 (19.01)	72.76 (19.57)	70.93 (20.71)	.000	None

--**Figure 3 Here**--

For peer review only - http://bmjopen.bmj.com/site/about/guidelines.xhtml

Discussion

Statement of principle findings

The overarching aim of this research was to test the feasibility and acceptability of the 'ACTing Minds' video game as a DHI for treating depression, anxiety, and stress. Results demonstrate that the study design and videogame intervention are feasible for testing in a full-scale RCT. Thematic analysis on qualitative data revealed several key findings. Firstly, participants were successfully able to learn about core ACT principles through embedded learning, specifically acceptance, defusion from thoughts, and commitment to personal values. Participants felt that the lessons taught within the game could be applied to their daily lives, and that the game was effective in priming them to consider the core principles throughout the weeks following completion of the intervention. Participants also felt that they would recommend the game to someone that they care about, and that they would be interested in downloading and completing future releases of 'ACTing Minds'.

Summary of secondary outcomes

Quantitative analysis of measures taken at baseline, immediately post-intervention, and after a 3-week follow up, revealed one large effect size indicating increased partaking in usual activities ($\eta p2 = .307$). As well as three medium effect sizes relating to reduced stress ($\eta p2 = .108$), anxiety ($\eta p2 = .096$), increased psychological flexibility ($\eta p2 = .060$). Small effect sizes were found for depression ($\eta p2 = .011$), social connectedness ($\eta p2 = .021$), general wellbeing ($\eta p2 = .011$), reduced EQ5D pain/discomfort ($\eta p2 = .010$), and EQ5D anxiety/depression ($\eta p2 = .018$).

Comparison to existing literature

For peer review only - http://bmjopen.bmj.com/site/about/guidelines.xhtml

This is the first study to utilise a videogame Digital Health Intervention (DHI) rooted in third-wave behavioural therapy to address mental distress. 'ACTing Minds' imparts psychological skills based on Acceptance and Commitment Therapy (ACT) to promote wellbeing. Prior research has primarily focused on videogame DHIs targeting 'illbeing', that aim to reduce symptoms of mental illness, two examples are games' 'REThink' and 'Dojo'. 'REThink,' designed for a younger audience, developed the players' ability to discern functional emotions from maladaptive ones, and was shown to effectively improve emotional symptoms and reduce depressive mood (38). 'Dojo,' utilising biofeedback and relaxation techniques to promote emotional regulation, was shown to significantly decrease participants' anxiety and aggressive behaviour scores post-intervention but exhibited no long-term effects at a 4-month follow-up (39). Our DHI differs fundamentally from these other games due to strong theoretical underpinnings based on ACT. Instead of a focus on regulating or reducing unwanted emotions, ACT teaches acceptance of unpleasant emotions, which are an inherent part of being human. Practitioners are taught to observe their emotions without judgement or attempt to change them, a key focus within the 'ACTing Minds' intervention.

Several meta-analyses have been conducted to explore the potential efficacy of DHI's, one found that the majority of DHI's were based on Cognitive Behavioural Therapy (CBT), and that the effect size for such interventions were small for reducing depressive symptoms compared to non-treatment controls (40). CBT-based DHI's usually do not take the form of full videogames, but may include elements of gamification such as rewards, badges, and progress tracking. They are typically structured programs including online education tools, interactive exercises, and self-assessment tools, which focus on challenging and modifying negative thoughts and behaviours. Another meta-analysis including 34 RCT's (17 of which were CBT-based) found that CBT-based DHI's yielded a medium effect size for reducing symptoms of depression and anxiety (41). However, a meta-analysis of 117 CBT-based

For peer review only

applications revealed that only 12 of them provided support aligned with the evidence-based tenets of CBT (42). This finding suggests that the observed effectiveness in earlier studies could potentially stem from participants' interaction with a DHI rather than their proficient implementation of CBT principles. CBT-DHI programs often require consistent use, and high attrition rates have limited their efficacy in research (43). One meta-analysis author suggested that DHI's may need to be complemented by existing mental health support (40). However, our study challenges this notion. In just a 40-minute playthrough of 'ACTing Minds', participants fully explored the game, retained ACT knowledge, and discussed its application in interviews conducted 3 weeks post-intervention.

Strengths and limitations

Our study is the first to explore the feasibility and acceptability of a novel videogame DHI based on ACT. A core strength of this research was the utilisation of a mixed methods approach. By incorporating thematic analysis of semi-structured interviews as well as quantitative analysis of questionnaire data, we were able to gain a comprehensive understanding of participant experience using 'ACTing Minds' DHI. Collecting quantitative data at three separate time points, and interviews conducted at two separate time points meant that we were able to examine the process of change and identify patterns of improvement. We gained valuable input from participants in terms of suggestions for improving the intervention which will aid in the further development of 'ACTing Minds' as to optimise effectiveness and user engagement. The results from the interviews also indicated that 'ACTing Minds' has broad appeal as a videogame even to those outside of clinical populations. However, it is important to acknowledge the limitations of our study. Firstly, there was no control group, meaning that no causal links can be made between the intervention and our findings. There was a lack of objective data collection, including user activity, adherence to in-game tasks, and physiological measurements, limiting our ability to draw conclusions about engagement

For peer review only - http://bmjopen.bmj.com/site/about/guidelines.xhtml

with the intervention and changes in physiological wellbeing outcomes such as heart rate variability. The reliance on self-report measures meant that there was potential for biased responses, as participants knowing that they are taking part in a study may have answered interview questions in line with their perceptions of what the study was looking for. Participants in the study were also confused by a section of the WEMWBS questionnaire, which asked about their wellbeing in the two weeks before they completed it. This posed an issue as participants had to complete the questionnaire twice in one day, both before and after the intervention. Lastly, it is possible that some of the questionnaires used in this study were insufficient for capturing the target measurements. The AAQ-II psychological flexibility questionnaire has been criticised as being too simple a measure for psychological flexibility (44) and is likely also the case for our social connectedness measurement which included only two questions adapted from the UCLA loneliness scale.

Clinical implications and directions for future research

The ACT based videogame DHI used in this study is a low-cost, engaging, and easy to disseminate means of supporting those who suffer mental distress. The present study highlights the clinical implications of 'ACTing Minds', including its' potential therapeutic value, user engagement, and accessibility. However, further research is warranted to establish long-term effects, explore specific populations, conduct comparative studies, investigate underlying mechanisms, and address ethical considerations. Critically, a full randomised control trial is now needed, in which participants are compared quantitatively with a control group, incorporating physiological wellbeing measures such as heart rate variability, as well as research validated questionnaires regarding mental health (i.e., depression, stress, anxiety, psychological flexibility, social connectedness, and wellbeing). By pursuing these future research directions, we can leverage the potential of videogames to enhance patient care, improve outcomes, and expand the reach of interventions in an increasingly digital era.

For peer review only

Conclusion

The results of this study demonstrate that 'ACTing Minds' is feasible to implement in a full-scale randomised control trial. Both the intervention and study were well-received by participants, thematic analysis of semi-structured interviews indicated that a single playthrough of the game was sufficient for teaching several core principles of ACT, namely 'Acceptance', 'Commitment to Values', and 'Cognitive Defusion' to participants, and priming them to implement the lessons in their day-to-day lives. Quantitative results indicate that playing 'ACTing Minds' is associated with decreases in depression, anxiety, and stress, as well as increases in psychological flexibility, social connectedness, and wellbeing. However, these effects will need to be further explored in an adequately powered RCT in order to understand the potential clinical implications, therapeutic value, user engagement, and accessibility of an ACT-based video game DHI.

Data availability statement

The datasets supporting the conclusions of this article are available in the Open Science Framework: https://osf.io/3wuh5/?view_only=7998f1d0ae4b473ab7089a71be710270.

Original published protocol

https://bmjopen.bmj.com/content/10/11/e041667

Contribution statement

DJE and AHK designed the original protocol, whilst TCG updated and revised the protocol design. TCG wrote the first draft of the paper and conducted all of the quantitative and qualitative results. DJE and AHK provided substantial revisions on all drafts and advised TCG throughout the development of this manuscript. DJE designed and developed the game development.

Public and patient involvement

Key stakeholders were consulted and involved in the development of this protocol. The Patient Experience and Evaluation in Research (Patient Experience and Evaluation in Research (PEER): https://www.swansea.ac.uk/humanandhealthsciences/research-at-the-college-of-human-and-health/patientexperienceandevaluationinresearchpeergroup/) group in the College of Human and Health Sciences at Swansea University were consulted. This group represented members of the public, students and staff members, several of whom reported that they had experienced depression, anxiety or stress at some point in their lives and emphasised the need for innovative approaches of the delivery of mental health support. The feasibility design was explained to them, and they gave positive feedback about the nature of the design, intervention and outcome measures.

Competing interests

The game ACTing Minds was developed using European development funds via the commercial entity of Swansea University (AgorIP) and awarded to DJE with the intention to develop this game with for commercial purposes (as a game app for the Apple and Google Play stores). DJE was involved in the design of the protocol but did not recruit participants, collect any data, and did not conduct the analysis on the data. TCG and AHK have no involvement in any commercial aspects of the game.

Funding

Funding for the development of this game came from European development funds via the commercial entity of Swansea University called AgorIP (Reference: 229-0256-0046) awarded to DJE.

Acknowledgements

We would like to thank Miricle Tea Studios Ltd and Mikoshi Ltd for the production and release of the ACTing Minds videogame.

Ethics statement

For peer review only

This study has received ethical approval from Swansea University School of Psychology ethics sub-committee (2022-5630-4834).

Figure legends

Figure 1.

(A) Screenshot from 'ACTing Minds' showing the 'Psychoflexameter'. (B) The Acceptance and Commitment Therapy Hexaflex and core processes. (C) Screenshot from 'ACTing Minds', example of 'Cognitive defusion' task. The player is required to type in their own negative thoughts, before dragging them on to a leaf that floats downstream.

Figure 2.
Flow diagram of participants through the study.

Figure 3.
Visualisation diagram illustrating integrated outcomes of 'ACTing Minds' feasibility and acceptability study.

References

1. Fineberg NA, Haddad PM, Carpenter L, Gannon B, Sharpe R, Young AH, et al. The size, burden and cost of disorders of the brain in the UK. Journal of Psychopharmacology. 2013;27(9):761-70.

2. GBoDC N. Global Burden of Disease Study 2017 Results. Seattle, United States: Global Burden of Disease Collaborative Network. 2018.

3. Dettmann LM, Adams S, Taylor G. Investigating the prevalence of anxiety and depression during the first COVID-19 lockdown in the United Kingdom: Systematic review and meta-analyses. British Journal of Clinical Psychology. 2022;61(3):757-80.

4. Flynn D, Moloney E, Bhattarai N, Scott J, Breckons M, Avery L, et al. COVID-19 pandemic in the United Kingdom. Health Policy and Technology. 2020;9(4):673-91.

5. McManus S, Bebbington PE, Jenkins R, Brugha T. Mental health and wellbeing in England: the adult psychiatric morbidity survey 2014: NHS digital; 2016.

For peer review only - http://bmjopen.bmj.com/site/about/guidelines.xhtml

6. Salaheddin K, Mason B. Identifying barriers to mental health help-seeking among young adults in the UK: a cross-sectional survey. British Journal of General Practice. 2016;66(651):e686-e92.

7. Serafino P. Exploring the UK's digital divide. Office for National Statistics. 2019.

8. Lemenager T, Neissner M, Koopmann A, Reinhard I, Georgiadou E, Müller A, et al. COVID-19 lockdown restrictions and online media consumption in Germany. International journal of environmental research and public health. 2021;18(1):14.

9. Talevi D, Socci V, Carai M, Carnaghi G, Faleri S, Trebbi E, et al. Mental health outcomes of the CoViD-19 pandemic. Rivista di psichiatria. 2020;55(3):137-44.

10. Murray E, Hekler EB, Andersson G, Collins LM, Doherty A, Hollis C, et al. Evaluating digital health interventions: key questions and approaches. Elsevier; 2016. p. 843-51.

11. Linehan C, Kirman B, Roche B, editors. Gamification as behavioral psychology. The gameful world: Approaches, issues, applications; 2015: MIT Press.

12. Ofcom. Gaming penetration in the United Kingdom (UK) from 2013 to 2022, by age group and gender 2023 [cited 2023 June 19]. Available from: https://www.statista.com/statistics/300513/gaming-by-demographic-group-uk/.

13. Kuosmanen T, Fleming T, Newell J, Barry M. A pilot evaluation of the SPARX-R gaming intervention for preventing depression and improving wellbeing among adolescents in alternative education. Internet Interventions. 2017;8:40-7.

14. Rusch DC, editor " Elude" designing depression. Proceedings of the International Conference on the Foundations of Digital Games; 2012.

15. Scholten H, Malmberg M, Lobel A, Engels RC, Granic I. A randomized controlled trial to test the effectiveness of an immersive 3D video game for anxiety prevention among adolescents. PloS one. 2016;11(1):e0147763.

For peer review only

16. Edwards DJ, Kemp AH. A novel ACT-based video game to support mental health through embedded learning: a mixed-methods feasibility study protocol. BMJ open. 2020;10(11):e041667.

17. Barr M, Copeland-Stewart A. Playing video games during the COVID-19 pandemic and effects on players' well-being. Games and Culture. 2022;17(1):122-39.

18. Hayes S, Strosahl K, Wilson K. Acceptance and commitment therapy: American Psychological Association. Washington, DC[Google Scholar]. 2009.

19. Hayes SC, Hofmann SG. The third wave of cognitive behavioral therapy and the rise of process-based care. World psychiatry. 2017;16(3):245.

20. Harris R. ACT made simple: An easy-to-read primer on acceptance and commitment therapy: New Harbinger Publications; 2019.

21. Gloster AT, Walder N, Levin ME, Twohig MP, Karekla M. The empirical status of acceptance and commitment therapy: A review of meta-analyses. Journal of Contextual Behavioral Science. 2020;18:181-92.

22. Mattila E, Lappalainen R, Välkkynen P, Sairanen E, Lappalainen P, Karhunen L, et al. Usage and dose response of a mobile acceptance and commitment therapy app: secondary analysis of the intervention arm of a randomized controlled trial. JMIR mHealth and uHealth. 2016;4(3):e5241.

23. Singh S, Starkey NJ, Sargisson RJ. Using SmartQuit®, an acceptance and commitment therapy smartphone application, to reduce smoking intake. Digital health. 2017;3:2055207617729535.

24. Hayes SC, Strosahl KD, Wilson KG. Acceptance and commitment therapy: The process and practice of mindful change: Guilford press; 2011.

25. Hayes S. A Liberated Mind: The essential guide to ACT: Random House; 2019.

For peer review only

26. Craig P, Dieppe P, Macintyre S, Michie S, Nazareth I, Petticrew M. Developing and evaluating complex interventions: the new Medical Research Council guidance. Bmj. 2008;337.

27. Billingham SA, Whitehead AL, Julious SA. An audit of sample sizes for pilot and feasibility trials being undertaken in the United Kingdom registered in the United Kingdom Clinical Research Network database. BMC medical research methodology. 2013;13:1-6.

28. Eldridge SM, Chan CL, Campbell MJ, Bond CM, Hopewell S, Thabane L, et al. CONSORT 2010 statement: extension to randomised pilot and feasibility trials. bmj. 2016;355.

29. Henry JD, Crawford JR. The short-form version of the Depression Anxiety Stress Scales (DASS-21): Construct validity and normative data in a large non-clinical sample. British journal of clinical psychology. 2005;44(2):227-39.

30. Bond FW, Hayes SC, Baer RA, Carpenter KM, Guenole N, Orcutt HK, et al. Preliminary psychometric properties of the Acceptance and Action Questionnaire–II: A revised measure of psychological inflexibility and experiential avoidance. Behavior therapy. 2011;42(4):676-88.

31. Russell DW. UCLA Loneliness Scale (Version 3): Reliability, validity, and factor structure. Journal of personality assessment. 1996;66(1):20-40.

32. Kok BE, Coffey KA, Cohn MA, Catalino LI, Vacharkulksemsuk T, Algoe SB, et al. How positive emotions build physical health: Perceived positive social connections account for the upward spiral between positive emotions and vagal tone. Psychological science. 2013;24(7):1123-32.

33. Tennant R, Hiller L, Fishwick R, Platt S, Joseph S, Weich S, et al. The Warwick-Edinburgh mental well-being scale (WEMWBS): development and UK validation. Health and Quality of life Outcomes. 2007;5(1):1-13.

For peer review only - http://bmjopen.bmj.com/site/about/guidelines.xhtml

34. Balestroni G, Bertolotti G. EuroQol-5D (EQ-5D): an instrument for measuring quality of life. Monaldi Archives for Chest Disease. 2012;78(3).

35. Clarke V, Braun V. Thematic analysis: a practical guide. Thematic Analysis. 2021:1-100.

36. Grissom RJ, Kim JJ. Effect sizes for research: Univariate and multivariate applications: Routledge; 2012.

37. McCrudden MT, Marchand G, Schutz PA. Joint displays for mixed methods research in psychology. Methods in Psychology. 2021;5:100067.

38. David OA, Cardoș RA, Matu S. Is RET hink therapeutic game effective in preventing emotional disorders in children and adolescents? Outcomes of a randomized clinical trial. European child & adolescent psychiatry. 2019;28:111-22.

39. Schuurmans AA, Nijhof KS, Engels RC, Granic I. Using a videogame intervention to reduce anxiety and externalizing problems among youths in residential care: an initial randomized controlled trial. Journal of psychopathology and behavioral assessment. 2018;40:344-54.

40. Garrido S, Millington C, Cheers D, Boydell K, Schubert E, Meade T, et al. What works and what doesn't work? A systematic review of digital mental health interventions for depression and anxiety in young people. Frontiers in psychiatry. 2019;10:759.

41. Grist R, Croker A, Denne M, Stallard P. Technology delivered interventions for depression and anxiety in children and adolescents: a systematic review and meta-analysis. Clinical Child and Family Psychology Review. 2019;22:147-71.

42. Huguet A, Rao S, McGrath PJ, Wozney L, Wheaton M, Conrod J, et al. A systematic review of cognitive behavioral therapy and behavioral activation apps for depression. PloS one. 2016;11(5):e0154248.

For peer review only

43. Venkatesan A, Rahimi L, Kaur M, Mosunic C. Digital cognitive behavior therapy intervention for depression and anxiety: retrospective study. JMIR Mental Health. 2020;7(8):e21304.

44. Doorley JD, Goodman FR, Kelso KC, Kashdan TB. Psychological flexibility: What we know, what we do not know, and what we think we know. Social and Personality Psychology Compass. 2020;14(12):1-11.

For peer review only

For peer review only - http://bmjopen.bmj.com/site/about/guidelines.xhtml

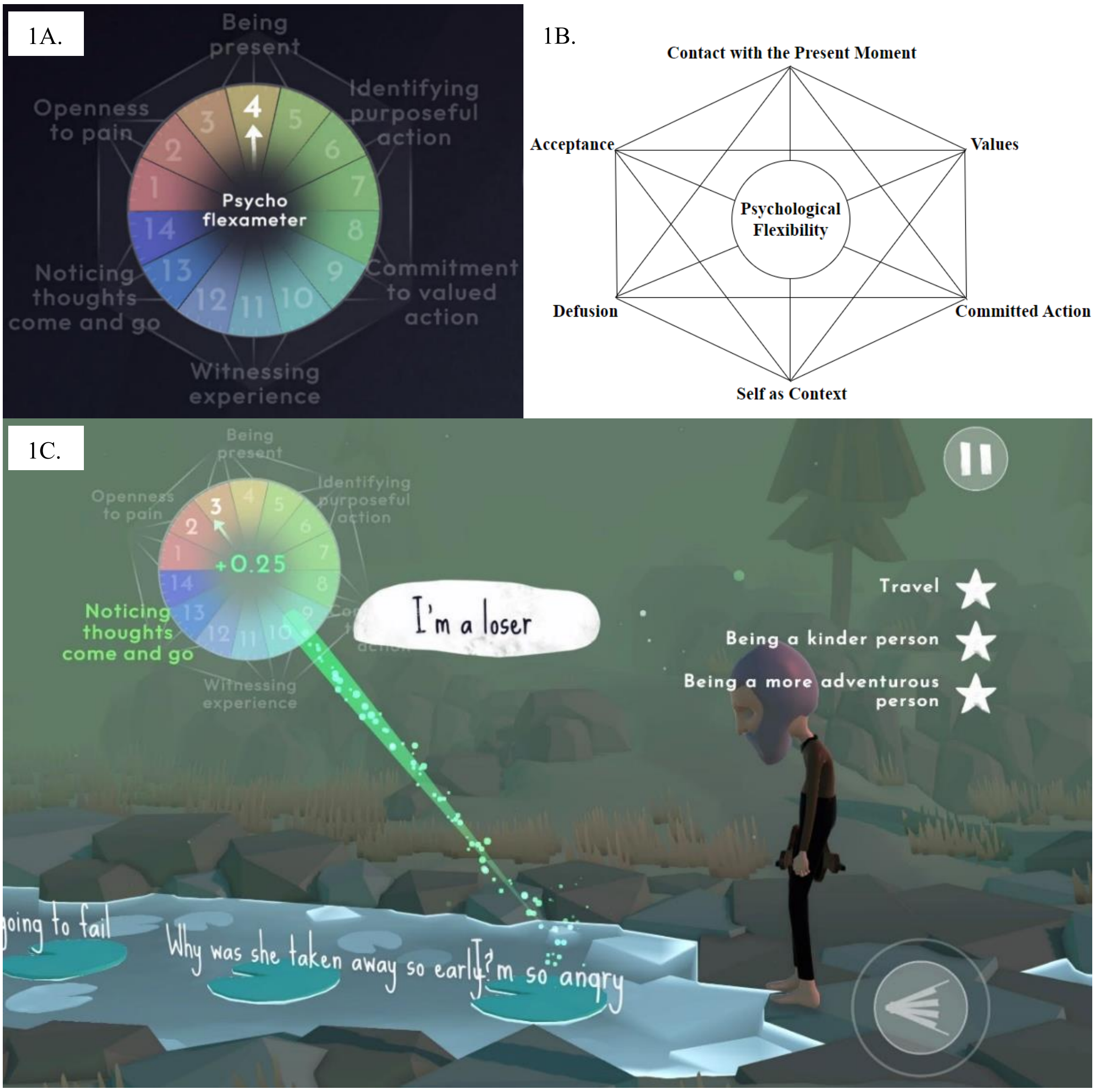
1A.
Being present
Identifying purposeful action
Commitment to valued action
Witnessing experience
Noticing thoughts come and go
Openness to pain
Psycho flexameter
1B.
Contact with the Present Moment
Acceptance
Values
Psychological Flexibility
Defusion
Committed Action
Self as Context
1C.
+0.25
I'm a loser
Travel
Being a kinder person
Being a more adventurous person
Why was she taken away so early
I'm so angry

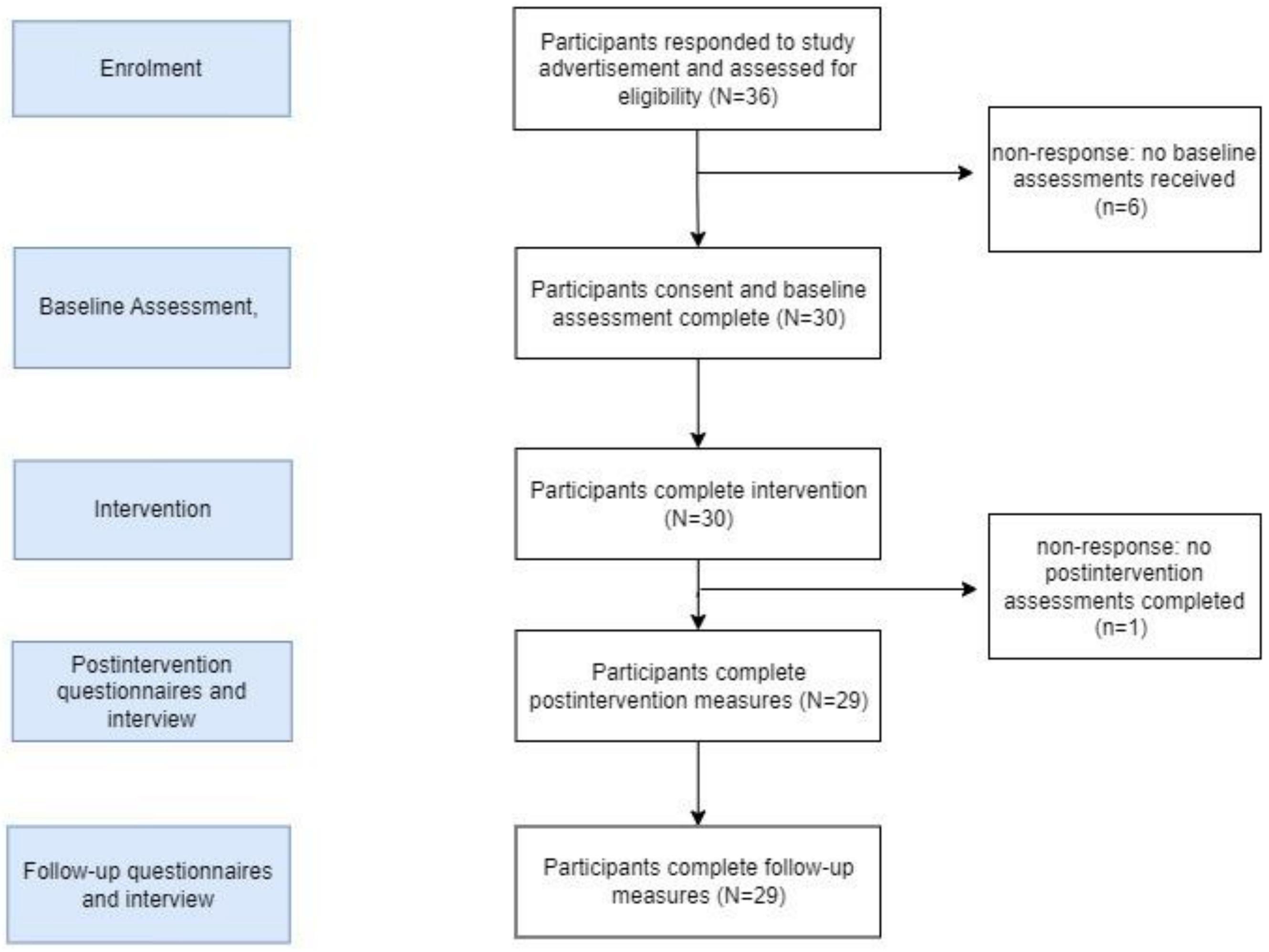
Enrolment
Participants responded to study advertisement and assessed for eligibility (N=36)
non-response: no baseline assessments received (n=6)
Baseline Assessment,
Participants consent and baseline assessment complete (N=30)
Intervention
Participants complete intervention (N=30)
non-response: no postintervention assessments completed (n=1)
Postintervention questionnaires and interview
Participants complete postintervention measures (N=29)
Follow-up questionnaires and interview
Participants complete follow-up measures (N=29)

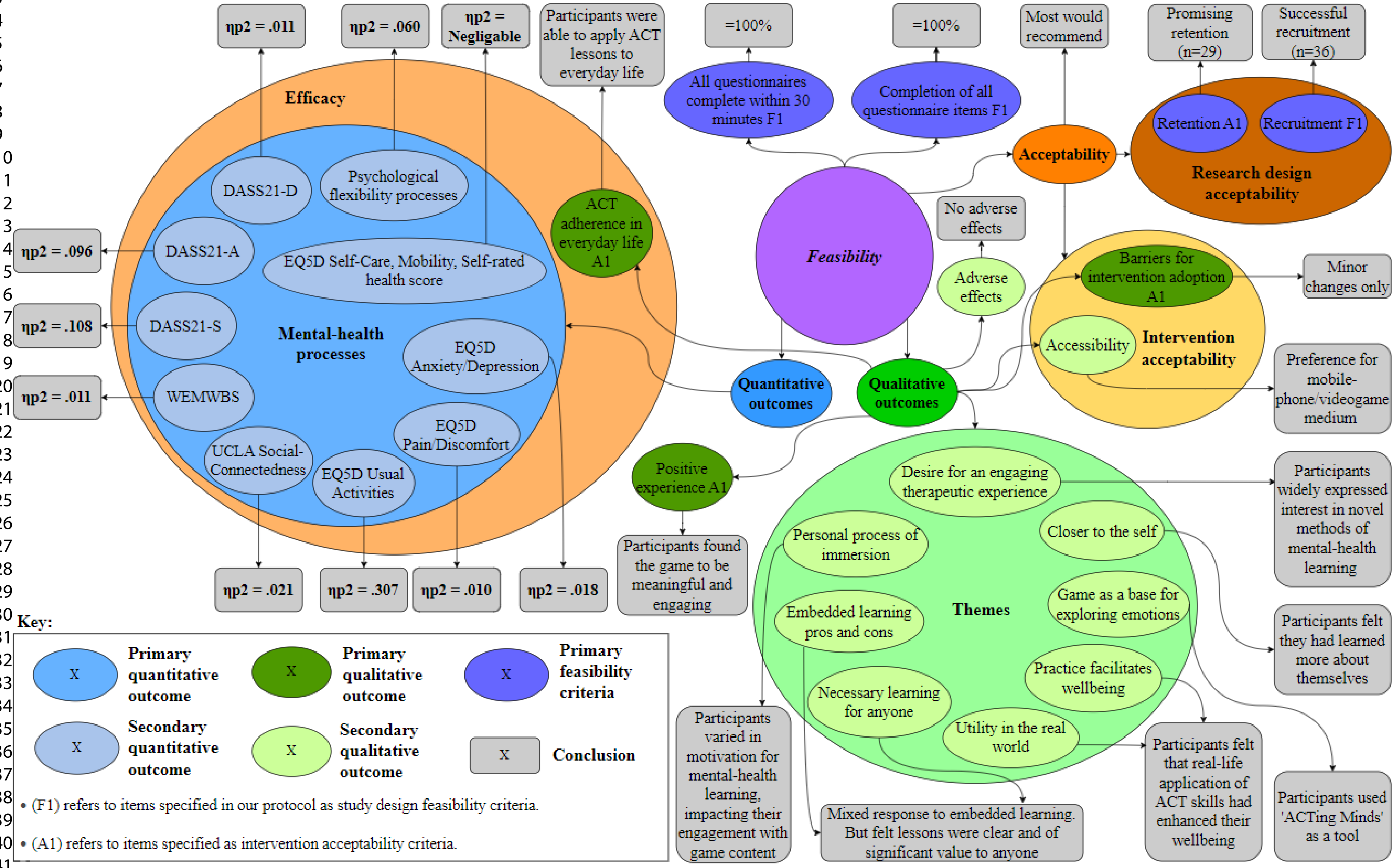
ηp2 = .011
ηp2 = .060
ηp2 = Negligable
Participants were able to apply ACT lessons to everyday life
=100%
=100%
Most would recommend
Promising retention (n=29)
Successful recruitment (n=36)
Efficacy
All questionnaires complete within 30 minutes F1
Completion of all questionnaire items F1
Retention A1
Recruitment F1
Acceptability
Research design acceptability
DASS21-D
Psychological flexibility processes
ACT adherence in everyday life A1
Feasibility
No adverse effects
ηp2 = .096
DASS21-A
EQ5D Self-Care, Mobility, Self-rated health score
Adverse effects
Barriers for intervention adoption A1
Minor changes only
ηp2 = .108
DASS21-S
Mental-health processes
EQ5D Anxiety/Depression
Accessibility
Intervention acceptability
Preference for mobile-phone/videogame medium
ηp2 = .011
WEMWBS
Quantitative outcomes
Qualitative outcomes
UCLA Social-Connectedness
EQ5D Pain/Discomfort
EQ5D Usual Activities
Positive experience A1
Desire for an engaging therapeutic experience
Participants widely expressed interest in novel methods of mental-health learning
Personal process of immersion
Closer to the self
Participants found the game to be meaningful and engaging
ηp2 = .021
ηp2 = .307
ηp2 = .010
ηp2 = .018
Embedded learning pros and cons
Themes
Game as a base for exploring emotions
Participants felt they had learned more about themselves
Practice facilitates wellbeing
Necessary learning for anyone
Utility in the real world
Participants varied in motivation for mental-health learning, impacting their engagement with game content
Mixed response to embedded learning. But felt lessons were clear and of significant value to anyone
Participants felt that real-life application of ACT skills had enhanced their wellbeing
Participants used 'ACTing Minds' as a tool
Key:
X Primary quantitative outcome
X Primary qualitative outcome
X Primary feasibility criteria
X Secondary quantitative outcome
X Secondary qualitative outcome
X Conclusion
• (F1) refers to items specified in our protocol as study design feasibility criteria.
• (A1) refers to items specified as intervention acceptability criteria.

Open access Protocol

BMJ Open

A novel ACT-based video game to support mental health through embedded learning: a mixed-methods feasibility study protocol

Darren J Edwards [1], Andrew H Kemp [2]

To cite: Edwards DJ, Kemp AH. A novel ACT-based video game to support mental health through embedded learning: a mixed-methods feasibility study protocol. *BMJ Open* 2020;**10**:e041667. doi:10.1136/bmjopen-2020-041667

► Prepublication history and additional material for this paper is available online. To view these files, please visit the journal online (http://dx.doi.org/10.1136/bmjopen-2020-041667).

Received 15 June 2020
Revised 06 October 2020
Accepted 30 October 2020

Check for updates

© Author(s) (or their employer(s)) 2020. Re-use permitted under CC BY-NC. No commercial re-use. See rights and permissions. Published by BMJ.

[1]Department of Public Health, Policy, and Social Sciences, Swansea University, Swansea, UK
[2]Department of Psychology, Swansea University, Swansea, UK

Correspondence to
Dr Darren J Edwards;
D.J.Edwards@swansea.ac.uk

ABSTRACT

Introduction In recent years, serious video games have been used to promote emotional regulation in individuals with mental health issues. Although these therapeutic strategies are innovative, they are limited with respect to scope of treatment, often focusing on specific cognitive skills, to help remediate a specific mental health disorder.
Objective Here, we propose a protocol for assessing the feasibility of a novel acceptance and commitment therapy (ACT)-based video game for young adults.
Methods and analysis The Medical Research Council (MRC) framework will be used for developing a complex intervention to design and test the feasibility of an ACT-based video game intervention using a mixed-methods approach involving qualitative and quantitative data. The primary outcomes will include feasibility testing of recruitment processes and the acceptability of the intervention through qualitative interviews, attendance and rates of attrition. Secondary outcomes will involve a series of quantitative questionnaires to obtain effect sizes for power analysis, allowing for the ideal sample size for an appropriately powered, randomised controlled trial to be determined.
Ethics and dissemination This study has been approved by the Psychology Department Research Ethics Committee (2020-4929-3923) at Swansea University in the UK. Dissemination activities will involve publications in peer-reviewed journals, presentations at local and national conferences and promotion through social media.
Trial registration number NCT04566042.

Strengths and limitations of this study

- Mixed-methods approach to build a rich dataset on which conclusions will be drawn.
- Protocol follows established Medical Research Council (MRC) guidelines.
- In line with MRC guidelines and stage of game development, randomisation is not a component of this study.
- Aims are to assess feasibility, an important step in the development of complex interventions, although limiting conclusions able to be drawn.

INTRODUCTION

Mental health issues such as anxiety and depression are a global problem of increasing concern, imposing considerable burden on society. The Global Burden of Disease project[1] has identified mental health disorders as a leading cause of disability globally, and suggest that there are 266 million cases of anxiety, and 253 million cases of major depressive disorder globally.

Unfortunately, the demand for mental health services far exceeds the available human resources able to meet this need in high-income and low-income andmiddle-income countries. This includes ever-growing treatment gaps[2] and lags.[3] These alarming increases have prompted the 2018 Lancet commission on global mental health to suggest that universal health coverage should include efforts to ensure the sustainable development of mental health.[4] Innovations to promote accessibility to mental health treatments include technology such as telephone, internet and smartphone devices, augmenting the psychotherapeutic toolkit.[5]

Innovations in video gaming for remediating mental health issues have wide potential application. In the USA, over 164 million adults play video games, and at least three-quarters of all American families have at least one person who video games regularly.[6] In the EU, 54% of the population play video games between the ages of 6 and 64, where the average age of video gamers is 31, and with a distribution of 46% female and 54% male. Of these, 77% play at least 1 hour per week, 16% play 1 hour per month, while only 7% play 1 hour per year.[7] Given that such a large proportion of the Western population play video games, developing mental health training in the form of psychoeducation may have great potential for building psychological resilience and

BMJ Open: first published as 10.1136/bmjopen-2020-041667 on 16 November 2020. Downloaded from http://bmjopen.bmj.com/ on October 15, 2023 by guest. Protected by copyright.

For peer review only

For peer review only - http://bmjopen.bmj.com/site/about/guidelines.xhtml

helping to better manage depression, anxiety and other forms of distress.

Technological developments for tackling such challenges include the exploitation of gamification.[8] This involves the application of behavioural principles for controlling and modifying human behaviour, in which game design elements are used to increase human interaction with or without technology.[9] Some examples of gamification include gamifying the development of cognitive skills and emotional regulation by rewarding the completion of relevant tasks within complex video games[10–13] in order to promote mental health.

When mental health-related video games are designed well, they have been shown to elevate self-esteem, self-efficacy, knowledge and awareness of illness, adherence to treatment and problem-solving skills, while lowering aggression.[14] One of the most successful in the facilitation of mental health improvement is a serious video game—a complex game with multiple levels and settings—called PlayMancer (PM), which targets emotional regulation and was specifically designed to help manage impulse control disorders.[11 13] The objective of the PM game is to develop emotional and cognitive skills, while reducing impulsivity. The game has been shown to help treat bulimia nervosa by improving emotional regulation.[15 16]

PM also uses biofeedback (heart rate and heart rate variability) to model physiological and emotional reactions, feeding this information back to the participant. Some research has shown that facilitating awareness of one's own physiology (such as brain activity or cardiac function) enhances the treatment effects of mental health disorders (such as anxiety disorder, depression, obsessive-compulsive disorder (OCD) and schizophrenia) via self-regulation.[17] Biofeedback has also been shown to improve impulse control difficulties, and attentional difficulties in bulimia nervosa and attention deficit hyperactivity disorder,[15 16 18] as well as symptoms of stress, anxiety and anger.[19] The focus on physiological data in the psychotherapeutic context is gaining traction[20–22] and has strong theoretical underpinnings.[23–25]

Within PM, there are three mini-games: 'The face of Cronos'; 'Treasures of the sea' and 'Sign of the Magupta'. Each of these mini-games were designed to train different skills, for example, 'The face of Cronos' and 'Treasures of the sea' develops planning skills, impulse control, coping skills, stress management and emotional self-regulation, while 'Sign of the Magupta' was designed to train relaxation, breathing techniques and improve physiological and emotional awareness. However, in the study[15] PM was combined with sessions of cognitive behavioural therapy (CBT) and without a control measure (eg, CBT only) so the game was developed as an adjunct to traditional mental health training, and there is no real way of knowing the direct benefits of the game as opposed to training in CBT. In another study—a case study of a single participant playing PM—anxiety and impulsivity decreased prior to CBT.[16] However, as this study was based on a single case, further studies using a randomised controlled trial (RCT) approach are needed to support and provide confidence to these findings.

Another game, Dojo,[26] develops emotional regulation in adolescents with anxiety. It uses biofeedback (heart rate variability) and trains breathing techniques, muscle relaxation, positive thinking and guided imagery to attempt to reduce anxiety in adolescence. It also uses instructional videos and then engages players through immersive and emotionally evocative puzzles that challenge players to use newly acquired emotion regulation skills. However, a pre-post RCT with 1347 participants, compared with a standard 'off the shelf' commercial game 'Rayman 2' (whereby Rayman 2 was the control), reported no difference between Dojo and the control condition at reducing anxiety. As both of these games significantly reduced anxiety, it is possible that the reduction in anxiety was due distraction from anxiety-provoking thoughts, rather than developing psychoeducational skills per se. The authors concluded that crucial design issues need to be carefully thought through, which include a clear theoretical and therapeutic foundation. This includes appropriate methodology that can assess the causes of improvement, before developing and testing a serious video game for the treatment of mental health issues such as anxiety.

Commercial games (such as Rayman 2) have been explored in their unmodified forms for their effectiveness in helping with social skills training for autism, and cognitive distraction for anxiety and nausea for patients undergoing chemotherapy,[27] with limited success. Evidence of generalisability of these games beyond game-playing is limited,[28] and this may be because they act as simple distractions rather than therapeutic psychoeducation applicable to participant's everyday lives. Another issue with many of these studies is that they often lack appropriate and rigorous methodology such as longitudinal follow-up,[29] and a mixed-methodological approach that can assess the feasibility and acceptability of such interventions.

Given these issues, it is important to emphasise that the underlying theoretical basis for PM and Dojo relates to the development of emotional regulation skills. While emotional regulation has transdiagnostic application[30] (ie, an intervention designed to treat multiple mental health conditions), these applications are not underpinned by theoretical frameworks that relate to formal psychotherapeutic interventions. Our proposed game is designed to be a comprehensive transdiagnostic intervention that will integrate a third wave behavioural therapy—as opposed to an adjunct to—acceptance and commitment therapy (ACT).[31 32] It will therefore be a comprehensive strategy for managing many common mental health issues such as depression and anxiety and focus on developing clear psychoeducational skills in the form of psychological flexibility, well-being and resilience more generally.[33]

Given this comprehensive transdiagnostic focus on psychological flexibility through ACT—a fundamental component of general health and well-being[34]—our online video game may have much greater reach and

For peer review only

BMJ Open: first published as 10.1136/bmjopen-2020-041667 on 16 November 2020. Downloaded from http://bmjopen.bmj.com/ on October 15, 2023 by guest. Protected by copyright.

impact than other serious video games such as PM, Dojo and many of the commercial games which are not based on third wave psychotherapy. Greater accessibility and impact have important implications for reducing treatment gaps and lags by making more mental health services available to those who need them.

One reason for choosing ACT in the game development process was pragmatism. For instance, researchers and clinicians may access freely available materials through the Association of Contextual Behavioural Science website (https://contextualscience.org/), and it does not require formal clinical training or accreditation to practice[35] which has important implications for translation to video game platforms. Another reason for choosing ACT as the basis for the game, is that it has a strong evidence base, and meta-analysis has found it to be efficacious for improving chronic pain, depression, psychotic symptoms, mixed anxiety, OCD, drug abuse and stress at work.[36] This means it is an ideal general purpose therapeutic tool as opposed to restricted focus on for example impulsivity control such as the PM application[11 13] or simple relaxation skills for adolescence with anxiety, as is the focus of the Dojo game.[26]

ACT principles are designed to undermine the trappings of language in the form of difficult thoughts and associated feelings, and promotion of psychological flexibility.[37] Language trappings can get individuals entangled and can prevent them from engaging with what is truly meaningful to them. The development of psychological flexibility through ACT is important because it is considered to be a fundamental component of well-being.[34]

The six ACT processes are: (1) the act of being in the here and now, present and mindful[32 38]; (2) acceptance, the act of being aware and open to painful thoughts; (3) cognitive fusion, the act of recognising that thoughts are just thoughts and not to buy into them (the process of cognitive defusion)[39]; (4) identifying values, values act as a life compass and direct us towards a life filled with purpose; (5) commitment to values orientation, which is the act of continually working towards a values orientation, even when an individual goes off track; (6) self as context (also called the transcendental self), is flexible and transcendent form of self. This involves the awareness of thoughts and feelings but the complete detachment from the literal meaning of thoughts.[34]

ACT has been usefully applied to many forms of mental health issues and has been applied in many different forms of delivery. This includes web-based interventions,[40–42] teleconference[43] and a downloadable app for smartphones.[44 45] So, given the fact that video games can have positive well-being benefits,[46 47] and are applicable for therapeutic purposes,[28 48] a transdiagnostic ACT serious video-game may have great potential for similar reasons.

As ACT is a comprehensive transdiagnostic model and formal third wave cognitive behavioural approach, then its reach and impact in the form of a video game may be greater than that of PM or Dojo which were focused on simpler emotional skills development and biofeedback. For these reasons, we are proposing an ACT-based video game called 'ACTing Mind' as an innovative and accessible intervention to help individuals who struggle with anxiety, depression, stress and other forms of distress.

Aims

The research goals of this proposal are to determine the feasibility and acceptability of a novel ACT-based video game intervention for individuals with mental distress, in line with methodology described in the Medical Research Council (MRC) framework.[49 50] This proposal lays the foundation for which a pilot and full-scale RCT will be conducted to determine clinical effectiveness, and ultimately the recommendations of the importance of such innovations in primary care mental health policies and practices.

METHODOLOGY

This protocol has been developed following the Template for Intervention Description and Replication of Studies[51] (see online supplemental appendix 1), as well as the MRC guidelines for the development of complex interventions.[49 50] This includes five stages of development for a complex intervention including: (1) preclinical, involving a theoretical review of the literature (provided here), justifying the need for such an intervention for the proposed population; (2) phase I, modelling, involving the use of evidence to determine the components for underlying mechanisms. For this, we propose a qualitative element involving thematic analysis to enable us to understand what would be most beneficial to a general population with anxiety and depression; (3) phase II, conducting an exploratory pilot study (outlined here) to determine the feasibility of the methodology and design where some initial data can be collected; (4) phase III, an RCT to test the efficacy of the proposed intervention (in subsequent work); (5) phase IV, long-term follow-up to assess replicability.

Public and patient involvement

Key stakeholders were consulted and involved in the development of this protocol. The Patient Experience and Evaluation in Research (Patient Experience and Evaluation in Research (PEER): https://www.swansea.ac.uk/humanandhealthsciences/research-at-the-college-of-human-and-health/patientexperienceandevaluationinresearchpeergroup/) group in the College of Human and Health Sciences at Swansea University were consulted. This group represented members of the public, students and staff members, several of whom reported that they had experienced depression, anxiety or stress at some point in their lives and emphasised the need for innovative approaches of the delivery of mental health support. The feasibility design was explained to them, and they gave positive feedback about the nature of the design, intervention and outcome measures.

For peer review only - http://bmjopen.bmj.com/site/about/guidelines.xhtml

BMJ Open: first published as 10.1136/bmjopen-2020-041667 on 16 November 2020. Downloaded from http://bmjopen.bmj.com/ on October 15, 2023 by guest. Protected by copyright.

Study design

This is a mixed-methods study which is designed to determine the feasibility and acceptability of an ACT-based video game for individuals with anxiety, depression and stress, and to increase psychological flexibility.

Study setting

The study will be conducted entirely online including the game and assessment (via the Qualtrics platform), and qualitative interviews (via the Zoom platform). Thus, potential participants will be able to access this study without restrictions, an important consideration for ongoing local lockdowns associated with the COVID-19 pandemic. Strict recommendations will require participants to ensure they are in a quiet room and without disruption for the duration of the study.

Recruitment and consent

We will recruit participants (n=36) using purposive sampling, focusing on—unlike an opportunity sample—the types of participants needed for a full-scale RCT (ie, individuals with depression, anxiety and stress). The sample size is justified on the basis of past research reporting the median numbers of participants recruited for similar types of feasibility studies,[52] incorporating both quantitative and qualitative elements.

Eligibility criteria

Participants will be recruited through general public mental health forums, social media and student populations. Thirty-six participants will take part in the study and they will be aged 18 years or older, be experiencing ongoing depression, anxiety and stress, and be able to read, write and speak English.

Intervention

This ACT-based video game intervention called 'ACTing Mind', developed and designed solely by DE, will involve students and members of the public attending five 1 hour sessions of an ACT-based video game. Each session will involve a different chapter of the video game, and each chapter will explore a different key component of ACT, with there being six in total (see table 1 for the different chapters and sessions involved).

These various components and principles of ACT[31 32] will be taught within the different chapters of the game and through embedded learning. For example, the player will gain ACT skills while completing objectives within the game and without directly being taught these skills, but rewarded indirectly through points and progress awards. For instance, in one scene (see table 1) the character is confronted by painful memories, and the player has two choices: (1) to destroy the painful memories or (2) to accept these memories. If the player chooses to destroy the memories (avoidant-based strategies), the world becomes distorted and barriers form making the chapter impossible to complete. Alternatively, if the player chooses acceptance-based strategies they will be able to continue the game (hence in this scene they learn that acceptance is functionally better than avoidance).

The game will start with a depressed individual who has recently lost his wife in an accident, and is feeling depressed, isolated and lonely (see figure 1 as an example of this scene). Each chapter will reward ACT consistent behaviour with points on a 'psychoflexameter'. This is a dial on the border of the screen which indicates increased psychological flexibility as the player completes ACT-based tasks such as acceptance (chapter 1), being present (chapter 2), values and commitment (chapter 3), defusion (chapter 4) and self as context (chapter 5). ACT uses metaphors to help clients visualise the key processes of ACT. In the game, these metaphors are real representations, such as the 'sinking sand' game, 'dropping the rope' game, the 'chessboard game', the 'unwanted monster' game, the 'leaves on a stream' game (see table 1).

Within the game, the character will have to enter his own mind through a 'mind escape machine' (see figure 2 of this as an example of the character in his own mind). At the start of the game, it is explained through a brief historical story that he develops this machine to destroy and supress his unwanted painful thoughts and memories about his wife and loss. Once in his mind, he will learn that destroying or supressing thoughts creates barriers in his mind which prevents him from continuing the game. So, learning acceptance is crucial throughout this game and the character is rewarded for this through points and progress awards. Also, within the game, psychoeducation components explain thoughts as trappings of language which can often get people stuck in life, and prevent them from value consistent living, as well as the various emotional regulation strategies such as avoidance and acceptance.

As part of the study, in addition to playing the video game, participants will be asked to record events on a weekly basis, aspects of application of the ACT principles learnt in an everyday life in a journal. It is anticipated that greater adherence to the intervention in everyday life, and engagement with the journal will lead to greater success of the intervention (greater psychological flexibility).

Data collection and management

MSc students will have the opportunity to be involved in this study and will collect and process the data under supervision by project leads, DJE and AK. Questionnaires will be completed online through Qualtrics which will store raw data copies, and also be held on an encrypted university server. Names and other personally identifiable information will not be stored, and consent form information will not be associated with the raw or processed data, instead each participant will be given a unique identifier code. Similarity recorded interviewer transcripts will use identifier codes as opposed to personal information (eg, names). The project leads (DJE, AHK) will frequently audit all processes in data collection and processing to ensure that the procedures stated in this protocol are adhered to.

For peer review only

For peer review only - http://bmjopen.bmj.com/site/about/guidelines.xhtml

BMJ Open: first published as 10.1136/bmjopen-2020-041667 on 16 November 2020. Downloaded from http://bmjopen.bmj.com/ on October 15, 2023 by guest. Protected by copyright.

Table 1 Overview of the 'ACTing Mind' intervention and everyday journal instructions

Session 1 (week 1): acceptance and openness to pain	► Chapter 1—Acceptance<br>► Introducing participants to the video game and ACT in everyday journal.<br>► A brief overview of the purpose of the programme and the content of each session.<br>► Explaining basic ACT tenets through introduction text of journal.<br>► Explaining the nature of painful thoughts and memories and getting caught up in the struggle explained through journal.<br>► Basic story context about the character being depressed and why, at start of video game.<br>► Explaining the objective of the video game, that is, to transcend form psychological inflexibility to psychological flexibility.<br>► Exercise, within the game there are choice, either to suppress, and break thoughts, or to accept and be open to them.<br>► Acceptance and openness are rewarded by psychological flexibility points on the 'psychoflexameter' and game progression, while suppression actions (breaking or suppressing painful memories) are punished with physical barriers, and sinking sand, which prevent the player from progressing in the game.<br>► A monster pulls against the player to prevent progress, but if the player fights with the monster, they get even more stuck (analogous to the drop the rope and sinking sand metaphor). Again, acceptance is important and must be learnt here.<br>► Reflecting in the journal about how this might be applied in life, and when this has occurred throughout the week daily.
Session 2 (week 1): being present (mindfulness)	► Chapter 2—Being present (mindfulness)<br>► Some instructions form the journal about being present and mindful is given, why it is useful and how to go about achieving with breathing exercises.<br>► The character is approached by monsters in the game in the past and future making him worry excessively about imaginary dangers, and reminding him of painful events.<br>► The game (in the form of the character's wife's ghost) instructs the player to be present, to focus on your breathing for 10 min.<br>► As the participant learns and completes relevant psychological flexibility tasks psychological flexibility on the 'psychoflexameter' will increase, which rewards the player for being present.<br>► Reflecting in the journal about how this might be applied in life, and when this has occurred throughout the week daily.
Session 3 (week 2): values identification and commitment	► Chapter 3—Values identification and commitment<br>► Instructions about what are values (a life compass) explained through the journal.<br>► Acceptance and commitment to values orientation as opposed to avoidance behaviour is rewarded.<br>► There are challenges to reach goals which are linked to the character's values, such as scary weather and monsters.<br>► Psychological flexibility on the 'psychoflexameter' and game progress, will increase with values consistent behaviour which rewards the player for committing to values.<br>► Reflecting in the journal about how this might be applied in life, and when this has occurred throughout the week daily.
Session 4 (week 2): defusion	► Chapter 4—Defusion<br>► Instructions about what is cognitive fusion and defusion (holding self-stories lightly) explained through journal.<br>► The character goes back into the 'Mind Escape' machine but this time there is a flowing river with leaves (analogous to leaves on a stream metaphor).<br>► Some of the character's painful memories will beg the player to help them, but if the player interacts, barriers and quicksand appear, punishing the player and preventing them from progressing in the game (analogous to the sinking sand metaphor).<br>► The ghost of the character's wife eventually instructs the player to put the memories and thoughts onto the leaves and watch them flow down the river, without interacting with them, and to simply observe.<br>► Psychological flexibility on the 'psychoflexameter', will increase when all of the memories and thoughts as left to go down the stream, hence the player is rewarded for defusing.<br>► Reflecting in the journal about how this might be applied in life, and when this has occurred throughout the week daily.

Continued

BMJ Open: first published as 10.1136/bmjopen-2020-041667 on 16 November 2020. Downloaded from http://bmjopen.bmj.com/ on October 15, 2023 by guest. Protected by copyright.

For peer review only - http://bmjopen.bmj.com/site/about/guidelines.xhtml

Table 1 Continued

Session 5 (week 3): self as context	▶ Chapter 5—Self as context<br>▶ Instructions about what is self as context (being the observer of your thoughts and not your thought) are explained through journal.<br>▶ The world starts to fall apart and becomes abstract, like a chess board.<br>▶ The player realises that they are the white pieces on the chessboard (analogous to chess board metaphor).<br>▶ The player is compelled by the game to beat the black pieces in the chess game.<br>▶ But the more the players fights against the black pieces, the more they lose points on the 'psychoflexameter' and cannot progress in the game.<br>▶ The player must let the battle play out, once they do, they become aware that they are the chess board (they become it) and realise they do not need to be part of the never-ending battle between the opposing forces.<br>▶ Finally, a bus arrives, memories of the character's wife beg the player to stay, and the monsters pull on player.<br>▶ The player needs to get onto the bus with the monsters to move towards their values, a new beginning (analogues to bus metaphor).<br>▶ Finally, the player has a choice, go back and change the events that led to your wife's death, or stay on the bus with the monsters.<br>▶ Trying to change events leads to a loss in points and prevents game progression.<br>▶ Only staying on the bus, towards values, and accepting the monsters allows the player to complete the game successfully.<br>▶ Reflecting in the journal about how this might be applied in life, and when this has occurred throughout the week daily.

Outcome measures

Questionnaires will be collected at three points in time (baseline, immediate postintervention and 3-month follow-up). Interview data will be collected immediate postintervention only.

Demographic data

Demographic measures will include age, sex, medication use, which will all be recorded through Qualtrics and assessed by DJE and AHK.

Primary outcome measure

The primary outcomes for feasibility are determined using MRC framework measure for developing a complex intervention.[49 50] As this is a feasibility study, the primary outcomes (in accordance with the MRC framework) will include the acceptability of the ACT-based video game intervention, the feasibility of the recruitment, outcome measures and intervention adherence.

Acceptability

- Number of people dropping out.
- Barriers for adoption of intervention as assessed through interviews.
- Number of sessions attended.
- Time dedicated to home journal.
- ACT principles adherence in everyday life setting (as recorded in journal and expressed through interviews).
- Experience, identifying whether participants had positive experience with the intervention and whether they wanted to continue to be part of the intervention.

Feasibility

- Number of participants who are willing to take part.
- Time taken to complete questionnaires.
- Number of complete and incomplete questionnaires.

Secondary outcome measures

Warwick-Edinburgh Mental Well-Being Scale[53]: a measure of mental well-being with a focus on positive aspects of mental health. This measure has good internal

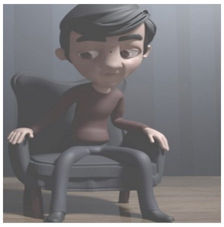

Figure 1 First scene in 'ACTing Mind', the character, Steve, is depressed and alone.

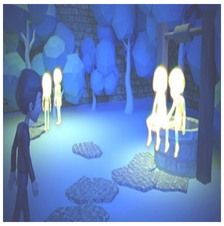

Figure 2 An example scene, where the character 'Steve' is in his own mind, and can see his own memories, through his Mindscape machine.

For peer review only - http://bmjopen.bmj.com/site/about/guidelines.xhtml

BMJ Open: first published as 10.1136/bmjopen-2020-041667 on 16 November 2020. Downloaded from http://bmjopen.bmj.com/ on October 15, 2023 by guest. Protected by copyright.

consistency with a Cronbach's alpha coefficient of 0.89 (student sample) and 0.91 (general population sample).

Depression Anxiety Stress Scales: a short version of this measure and a measure of general psychological distress with good construct validity (confirmatory factor analysis of 0.94). It has good internal reliability as measured through Cronbach's alpha coefficients, which are 0.88 for depression, 0.82 for anxiety, 0.90 for stress and 0.93 for the total scale.[54]

Social connectedness (adapted from Russell's (1996) UCLA Loneliness Scale[55]: this measure involves two questions: (1) "During social interactions, I feel 'in tune' with the person/s around me", and (2) "During social interactions, I feel close to the person/s". The Cronbach's alpha coefficients for these two items ranged from 0.80 to 0.98 (M=0.94, SD=0.03).[55]

EuroQol five dimensions (EQ5D): the EQ5D is a measure for health-related quality of life. There are five components within this measure which assess mobility, self-care, usual activities, pain, discomfort and anxiety. It also has a visual analogue scale (VAS) for measuring current health status. Scores for these will be calculated for each of these five subsections as well as including the VAS and total EQ5D score of all five subsections. The EQD5 correlates well with other health-related questionnaires such as the 36-Item Short Form Survey (r=0.61, p<0.0001) and Parkinson's Disease Questionnaire (PDQ)-39 (r=−0.75, p<0.0001).[56]

Acceptance and Action Questionnaire-second version: this is a seven-item scale developed by Bond *et al*[57] to measure psychological inflexibility, which involves the ability to accept and be open to difficult thoughts and feelings as well as to engage in valued behaviour in the presence of the difficult thoughts and feelings. A higher score indicates higher psychological inflexibility. The measure has good construct validity with a Cronbach's alpha coefficient of 0.84.[57]

Adherence to the intervention measure and trial

Adherence will be measured in a variety of ways such as intervention feedback, treatment adherence through attrition rates as well as meta-data of relating to game log-in and log-out, as well as how long the game was played for and what sessions of the game were completed for each participant. Similar information can be recorded in Qualtrics for ensuring questionnaires are completed carefully. This includes length of times completing the questionnaire, and paying attention to reverse-scored questions.

Sample size and statistical analysis

Sample size recruited will help us determine whether it is possible to recruit sufficient numbers of participants to manage a full-scale RCT at a later date.

Quantitative data analysis: analysis will focus on descriptive statistics and feasibility outcomes of the questionnaires. While clinical effectiveness will not be formally evaluated at this stage, effect sizes will be explored for early evidence that the intervention shows promising signs (including ACT-related process measures). It is predicted that outcomes will improve, and any improvement will be identified using a one-way analysis of covariance with a single within-subjects factor (time). The effect sizes will also allow for a power calculation to be made which will allow for an approximation for a sample size required in a future trial (if indicated).

Qualitative data analysis: transcripts of focus group interview data will be generated from digital audio-recordings of in-depth, face-to-face semi-structured interviews (all online and via a password-protected room in Zoom). In-depth semi-structured interviews will form the core topics to be discussed (see table 2), while leaving space and scope for the identification and exploration of unforeseen information that may emerge. Insights from this will allow for further development and improvement of the intervention, along with the quantitative data in line with the MRC guidelines.[49 50]

Thematic analysis will then be conducted which will explore key overarching themes that may emerge from the focus group interviews following standardised guidelines.[58] The interview questions are based on other novel ACT-based protocols.[40 59] The data will be analysed after the study has been completed. We will follow the inductive and deductive code development as outlined by Fereday and Muir-Cochrane[60] to ensure necessary rigour. Any key overarching themes identified which relate to feasibility of the study design of the acceptability of the intervention, as well as potential adverse effects, will be explored and reported.

The focus groups will comprise 4 to 6 groups with 6 to 10 individuals in each group as has been suggested as optimal in other studies.[61] The interview will take place at the end of the intervention (week 3). It will explore various aspects of the intervention such as perceived process of change, barriers to intervention adherence, trial process and any adverse effects, which help supplement the quantitative approach. Process of change questions indicate whether the participant learnt anything about ACT, and felt any positive change in their life due to participating in the intervention. The question relating to barriers explores any problems and difficulties they had with the intervention. Another question will be asked to elicit suggestions for improvement relating to game or study design. Acceptability questions and process of change in one's life relate to whether the participant accepted the intervention and used skills they learnt through the intervention in daily life. The question relating to the trial process will determine whether there were any difficulties or limitations of the trial itself such as whether the instructions were clear and how it could be improved. Finally, the question on adverse effects explores whether there were any potential unforeseen negative consequences of the intervention.

Limitations of the study

This study protocol has limitations. First, while physiological measures would ideally be collected to measure

For peer review only - http://bmjopen.bmj.com/site/about/guidelines.xhtml

BMJ Open: first published as 10.1136/bmjopen-2020-041667 on 16 November 2020. Downloaded from http://bmjopen.bmj.com/ on October 15, 2023 by guest. Protected by copyright.

Table 2 Qualitative interview protocol for the focus groups

Acceptability and feasibility	How would you describe your experience of taking part in 'ACTing Mind' video game programme?
Accessibility of intervention	If this intervention were rolled out as a video game app, do you think you would download it? Would you appreciate the accessibility?
Process of change	What did you learn from this programme?
Acceptability	What was the aspect of the programme that you liked the most? What was your favourite activity within the game (or applied to your everyday life)?
Suggestions for further improvement	What did you least like about the intervention? What do you think could be improved?
Barriers	Were there any difficulties to taking part?
Implementing change in everday life	Do you practice mindfulness, acceptance, defusion and values? How often? Could you apply what you have learnt through video game intervention to the real world in everyday events? Will you apply this new knowledge to everyday events?
Process of change	Have you noticed any differences in your life as a result of taking part in 'ACTing Mind'? If 'yes', what are these differences?
Acceptability	Would you recommend this intervention to someone you care about? Did you like the theoretical concepts central to the ACT intervention? How did you feel about its delivery? Was any of it too abstract or difficult to understand?
Processes of the trial	Was there anything you liked, or disliked about the study? How could we improve this study? Were all the instructions clear?
Adverse effects	Did you feel that any aspect of the intervention may have made worse any aspect of your anxiety, depression or stress? Were there any adverse effects that you can recognise due to the intervention?

variables such as heart rate variability, the COVID-19 pandemic limits our capacity to do this. However, the present study will provide important data on which such measures could be collected, analysed and interpreted in a future trial. Second, it could be questioned why there is no control condition in this study. Our response to this potential criticism is that the aim of the present study is to assess feasibility and—in line with the MRC guidelines[49 50]—has not been designed to be a full-scale RCT given the current research phase. Once the feasibility component is completed, a control condition will be introduced, which allow for the intervention condition to be compared with control, and as part of a full trial. Finally, although we would like to have ability to monitor the participant more directly, to ensure adherence to the intervention, we are sensitive to privacy issues associated with, for example, capturing participants' identity from the computers video camera. To mitigate this limitation, we have opted for less invasive procedures for measuring intervention adherence that will include logging metadata of the game such as log in and out times, as well as completion of game sections. Several questions in the questionnaire are also reverse scored to ensure participants are paying attention.

Protocol amendments

If the protocol is amended in any way, it will be communicated to relevant parties immediately, such as to participants, journal and ethics committee.

Ethics and dissemination

This study has received ethical approval from Swansea University Psychology Department ethics committee (2020-4920-3923). Participants will be informed of their rights to confidentiality and to leave the study at any time and without penalty. Both qualitative and quantitative data will be held on a password-protected computer accessible only to researchers DJE and AHK. The data will be anonymised with a unique identifier code, and any personally identifiable information will be removed.

Dissemination will involve peer-reviewed journals; leading national and international conferences, social media and public events and through general public health engagement such as talks at schools, the Welsh Government and engagement with annual science festivals including 'a pint of science'.

Impact of intervention

The potential impact of this study is far reaching as it will add to the growing set of online resources which support psychological resilience, flexibility and well-being. These resources are designed to be easy to access and are ideal for situations where travel is limited due to physical (disability) or situational (coronavirus) immobility. Such interventions can help alleviate widely reported mental health treatment gaps[2] and lags,[3] associated with the widely reported scarce human resources needed to provide mental health support for the many individuals who need it. The 2018 Lancet commission on global mental health argued that sustainable development

BMJ Open: first published as 10.1136/bmjopen-2020-041667 on 16 November 2020. Downloaded from http://bmjopen.bmj.com/ on October 15, 2023 by guest. Protected by copyright.
For peer review only - http://bmjopen.bmj.com/site/about/guidelines.xhtml

of mental health should be an essential component of universal health coverage.[4] Technological innovation of mental health support services, in the form of video games, may be one means to achieve this sustainability and a reduction in the treatment gap and lag.

Ancillary and poststudy care

Postintervention care has not been anticipated given this is a low-level (low-risk) intervention. Of course, all participants will be given a debrief form which will signpost individuals to the relevant free well-being services such as the Samaritans.

Acknowledgements The authors would like to thank Professor Louise McHugh (University College Dublin), who provided some very helpful feedback about the gamification of an ACT-based approach through the utilisation of behavioural principles.

Contributors DJE developed the intervention. DJE and AHK agreed on a set of outcomes. DJE wrote the first draft of the protocol and DJE and AHK then revised the subsequent drafts of the protocol. Both authors helped to revise the manuscript for intellectual content and agreed on the final version prior to submission for peer review.

Funding The authors have not declared a specific grant for this research from any funding agency in the public, commercial or not-for-profit sectors.

Competing interests At the time of writing this, DJE is discussing with Agor IP at Swansea University the potential to commercialise the described video game as a mobile application; however, at this time no agreements have been made or signed. AHK has no competing interests.

Patient consent for publication Not required.

Provenance and peer review Not commissioned; externally peer reviewed.

Supplemental material This content has been supplied by the author(s). It has not been vetted by BMJ Publishing Group Limited (BMJ) and may not have been peer-reviewed. Any opinions or recommendations discussed are solely those of the author(s) and are not endorsed by BMJ. BMJ disclaims all liability and responsibility arising from any reliance placed on the content. Where the content includes any translated material, BMJ does not warrant the accuracy and reliability of the translations (including but not limited to local regulations, clinical guidelines, terminology, drug names and drug dosages), and is not responsible for any error and/or omissions arising from translation and adaptation or otherwise.

Open access This is an open access article distributed in accordance with the Creative Commons Attribution Non Commercial (CC BY-NC 4.0) license, which permits others to distribute, remix, adapt, build upon this work non-commercially, and license their derivative works on different terms, provided the original work is properly cited, appropriate credit is given, any changes made indicated, and the use is non-commercial. See: http://creativecommons.org/licenses/by-nc/4.0/.

ORCID iD

Darren J Edwards http://orcid.org/0000-0002-2143-1198

REFERENCES

1 Whiteford H, Ferrari A, Degenhardt L. Global burden of disease studies: implications for mental and substance use disorders. *Health Aff* 2016;35:1114–20.
2 Patel V, Maj M, Flisher AJ, *et al*. Reducing the treatment gap for mental disorders: a WPA survey. *World Psychiatry* 2010;9:169–76.
3 Wang PS, Berglund PA, Olfson M, *et al*. Delays in initial treatment contact after first onset of a mental disorder. *Health Serv Res* 2004;39:393–416.
4 Patel V, Saxena S, Lund C, *et al*. The Lancet commission on global mental health and sustainable development. *The Lancet* 2018;392:1553–98.
5 Kazdin AE, Blase SL. Rebooting psychotherapy research and practice to reduce the burden of mental illness. *Perspect Psychol Sci* 2011;6:21–37.
6 ESA. 2019 essential facts about the computer and video game industry, 2020. Available: https://www.theesa.com/esa-research/2019-essential-facts-about-the-computer-and-video-game-industry/
7 ISFE. ISFE key facts 2019, 2020. Available: https://www.isfe.eu/isfe-key-facts/
8 Linehan C, Kirman B, Roche B. *Gamification as behavioral psychology. In the gameful world: approaches, issues, applications*. Cambridge, Massachusetts: MIT Press, 2015.
9 Deterding S, Dixon D, Khaled R, *et al*. From game design elements to gamefulness: defining "gamification". *Proceedings of the 15th international academic MindTrek conference: Envisioning future media environments*, 2011:9–15.
10 Jon Hobbs L, Yan Z. Cracking the walnut: using a computer game to impact cognition, emotion, and behavior of highly aggressive fifth grade students. *Comput Human Behav* 2008;24:421–38.
11 Jiménez-Murcia S, Fernández-Aranda F, Kalapanidas E, *et al*. Playmancer project: a serious videogame as an additional therapy tool for eating and impulse control disorders. *Stud Health Technol Inform* 2009;144:163–6.
12 Ducharme P, Wharff E, Hutchinson E, *et al*. Videogame assisted emotional regulation training: an act with RAGE-Control case illustration. *Clin Soc Work J* 2012;40:75–84.
13 Fernández-Aranda F, Jiménez-Murcia S, Santamaría JJ, *et al*. Video games as a complementary therapy tool in mental disorders: PlayMancer, a European multicentre study. *J Ment Health* 2012;21:364–74.
14 Santamaria JJ, Antonio S, Fernando F-A, *et al*. Serious games as additional psychological support: a review of the literature. *J Cyber Ther Reh* 2011;4:469–76.
15 Fagundo AB, Santamaría JJ, Forcano L, *et al*. Video game therapy for emotional regulation and impulsivity control in a series of treated cases with Bulimia nervosa. *Eur Eat Disord Rev* 2013;21:493–9.
16 Giner-Bartolomé C, Fagundo AB, Sánchez I, *et al*. Can an intervention based on a serious videogame prior to cognitive behavioral therapy be helpful in Bulimia nervosa? a clinical case study. *Front Psychol* 2015;6:982.
17 Schoenberg PLA, David AS. Biofeedback for psychiatric disorders: a systematic review. *Appl Psychophysiol Biofeedback* 2014;39:109–35.
18 Howard R, Schellhorn K, Lumsden J. A biofeedback intervention to control impulsiveness in a severely personality disordered forensic patient. *Personal Ment Health* 2013;7:168–73.
19 Pawlow LA, O'Neil PM, Malcolm RJ. Night eating syndrome: effects of brief relaxation training on stress, mood, hunger, and eating patterns. *Int J Obes Relat Metab Disord* 2003;27:970–8.
20 Dana DA. *The Polyvagal theory in therapy: engaging the rhythm of regulation (Norton series on interpersonal neurobiology)*. New York: WW Norton & Company, 2018.
21 Lehrer PM. Heart rate variability biofeedback and other psychophysiological procedures as important elements in psychotherapy. *Int J Psychophysiol* 2018;131:89–95.
22 Tulip C, Fisher Z, Bankhead H, *et al*. Building wellbeing in people with chronic conditions: a qualitative evaluation of an 8-week positive psychotherapy intervention for people living with an acquired brain injury. *Front Psychol* 2020;11:66.
23 Kemp AH, Koenig J, Thayer JF. From psychological moments to mortality: a multidisciplinary synthesis on heart rate variability spanning the continuum of time. *Neurosci Biobehav Rev* 2017;83:547–67.
24 Mead J, Wilkie L, Gibbs K, *et al*. Rethinking wellbeing: toward a more ethical science of wellbeing that considers current and future generations, 2019. Available: https://www.authorea.com/doi/full/10.22541/au.156649190.08734276
25 Kemp A. *Fisher, social ties, health and wellbeing: a literature review and model, in neuroscience and social science*. Berlin: Springer, 2017: 397–427.
26 Scholten H, Malmberg M, Lobel A, *et al*. A randomized controlled trial to test the effectiveness of an immersive 3D video game for anxiety prevention among adolescents. *PLoS One* 2016;11:e0147763.
27 Colder Carras M, Van Rooij AJ, Spruijt-Metz D, *et al*. Commercial video games as therapy: a new research agenda to unlock the potential of a global pastime. *Front Psychiatry* 2017;8:300.
28 Griffiths MD, Kuss DJ, de Gortari ABO. Videogames as therapy: an updated selective review of the medical and psychological literature. *JPHIM* 2017;5:71–96.
29 Zayeni D, Raynaud J-P, Revet A. Therapeutic and preventive use of video games in child and adolescent psychiatry: a systematic review. *Front Psychiatry* 2020;11:36.
30 Sloan E, Hall K, Moulding R, *et al*. Emotion regulation as a transdiagnostic treatment construct across anxiety, depression, substance, eating and borderline personality disorders: a systematic review. *Clin Psychol Rev* 2017;57:141–63.
31 Hayes S. *Acceptance and commitment therapy*. Washington, DC: American Psychological Association, 2009.

For peer review only

BMJ Open: first published as 10.1136/bmjopen-2020-041667 on 16 November 2020. Downloaded from http://bmjopen.bmj.com/ on October 15, 2023 by guest. Protected by copyright.

For peer review only - http://bmjopen.bmj.com/site/about/guidelines.xhtml

32 Hayes Steven C, Kirk D. *Acceptance and commitment therapy: the process and practice of mindful change*. New York: Guilford Press, 2011.
33 Dindo L, Van Liew JR, Arch JJ. Acceptance and commitment therapy: a transdiagnostic behavioral intervention for mental health and medical conditions. *Neurotherapeutics* 2017;14:546–53.
34 Kashdan TB, Rottenberg J. Psychological flexibility as a fundamental aspect of health. *Clin Psychol Rev* 2010;30:865–78.
35 Harris R. *Act made simple: an easy-to-read primer on acceptance and commitment therapy*. Oakland, California: New Harbinger Publications, 2009.
36 Öst L-G. The efficacy of acceptance and commitment therapy: an updated systematic review and meta-analysis. *Behav Res Ther* 2014;61:105–21.
37 Hayes SC, Strosahl KD, Bunting K, *et al*. *What is acceptance and commitment therapy?, in a practical guide to acceptance and commitment therapy*. Berlin: Springer, 2004: 3–29.
38 Strosahl K, Wilson K. *Acceptance and commitment therapy: an experiential approach to behavior change*. New York: Guilford Press, 1999.
39 Hayes SC. *Get out of your mind and into your life: the new acceptance and commitment therapy*. Oakland, California: New Harbinger Publications, 2005.
40 Edwards DJ, Rainey E, Boukouvala V, *et al*. Novel ACT-based eHealth psychoeducational intervention for students with mental distress: a study protocol for a mixed-methodology pilot trial. *BMJ Open* 2019;9:e029411.
41 Viskovich S, Pakenham KI. Pilot evaluation of a web-based acceptance and commitment therapy program to promote mental health skills in university students. *J Clin Psychol* 2018;74:2047–69.
42 Levin ME, Haeger JA, Pierce BG, *et al*. Web-based acceptance and commitment therapy for mental health problems in college students: a randomized controlled trial. *Behav Modif* 2017;41:141–62.
43 Herbert MS, Afari N, Liu L, *et al*. Telehealth versus in-person acceptance and commitment therapy for chronic pain: a randomized noninferiority trial. *J Pain* 2017;18:200–11.
44 Bricker JB, Mull KE, Kientz JA, *et al*. Randomized, controlled pilot trial of a smartphone APP for smoking cessation using acceptance and commitment therapy. *Drug Alcohol Depend* 2014;143: :87–94.
45 Levin ME, Haeger J, Pierce B, *et al*. Evaluating an adjunctive mobile APP to enhance psychological flexibility in acceptance and commitment therapy. *Behav Modif* 2017;41:846–67.
46 Johnson D, Jones C, Scholes L, *et al*. Videogames and wellbeing: a comprehensive review. young and well cooperative research centre, Melbourne, 2013. Available: https://www.youngandwellcrc.org.au/wp-content/uploads/2014/03/Videogames_and_Wellbeing.pdf
47 Vella K, Johnson D, Hides L. Positively playful: when videogames lead to player wellbeing. *Proceedings of the First International Conference on Gameful Design, Research, and Applications*, 2013:99–102.
48 Villani D, Carissoli C, Triberti S, *et al*. Videogames for emotion regulation: a systematic review. *Games Health J* 2018;7:85–99.
49 Craig P. Developing and evaluating complex interventions: following considerable development in the field since 2006, MRC and NIHR have jointly commissioned an update of this guidance to be published in 2019, 2019. Available: https://mrc.ukri.org/documents/pdf/complex-interventions-guidance/
50 Craig P, Dieppe P, Macintyre S, *et al*. Developing and evaluating complex interventions: the new medical Research Council guidance. *BMJ* 2008;337:a1655.
51 Hoffmann TC, Glasziou PP, Boutron I, *et al*. Better reporting of interventions: template for intervention description and replication (TIDieR) checklist and guide. *BMJ* 2014;348:g1687.
52 Billingham SAM, Whitehead AL, Julious SA. An audit of sample sizes for pilot and feasibility trials being undertaken in the United Kingdom registered in the United Kingdom clinical research network database. *BMC Med Res Methodol* 2013;13:104.
53 Tennant R, Hiller L, Fishwick R, *et al*. The Warwick-Edinburgh mental well-being scale (WEMWBS): development and UK validation. *Health Qual Life Outcomes* 2007;5:63.
54 Henry JD, Crawford JR. The short-form version of the depression anxiety stress scales (DASS-21): construct validity and normative data in a large non-clinical sample. *Br J Clin Psychol* 2005;44:227–39.
55 Kok BE, Coffey KA, Cohn MA, *et al*. How positive emotions build physical health: perceived positive social connections account for the upward spiral between positive emotions and vagal tone. *Psychol Sci* 2013;24:1123–32.
56 Schrag A, Selai C, Jahanshahi M, *et al*. The EQ-5D--a generic quality of life measure-is a useful instrument to measure quality of life in patients with Parkinson's disease. *J Neurol Neurosurg Psychiatry* 2000;69:67–73.
57 Bond FW, Hayes SC, Baer RA, *et al*. Preliminary psychometric properties of the acceptance and action Questionnaire-II: a revised measure of psychological inflexibility and experiential avoidance. *Behav Ther* 2011;42:676–88.
58 Braun V, Clarke V. Using thematic analysis in psychology. *Qual Res Psychol* 2006;3:77–101.
59 Saracutu M, Edwards DJ, Davies H, *et al*. Protocol for a feasibility and acceptability study using a brief ACT-based intervention for people from Southwest Wales who live with persistent pain. *BMJ Open* 2018;8:e021866.
60 Fereday J, Muir-Cochrane E. Demonstrating rigor using thematic analysis: a hybrid approach of inductive and deductive coding and theme development. *Int J Qual Methods* 2006;5:80–92.
61 Morgan DL. Focus groups. *Annu Rev Sociol* 1996;22:129–52.

For peer review only

BMJ Open: first published as 10.1136/bmjopen-2020-041667 on 16 November 2020. Downloaded from http://bmjopen.bmj.com/ on October 15, 2023 by guest. Protected by copyright.

For peer review only - http://bmjopen.bmj.com/site/about/guidelines.xhtml

Swansea University
Prifysgol Abertawe

PARTICIPANT INFORMATION SHEET

A novel ACT-based video game to support mental health through embedded learning

You are being invited to take part in some research. Before you decide whether or not to participate, it is important for you to understand why the research is being conducted and what it will involve. Please read the following information carefully.

What is the purpose of the research?

The aim of the research project is to gain insight into whether exposure to a novel-acceptance and commitment therapy (ACT) video game can improve various outcome measures of depression, anxiety, stress, and other measures relating to wellbeing.

If you are above the age of 18 and are currently experiencing ongoing mild to moderate levels of depression, anxiety, or stress you are welcome to participate in this study. For those individuals who present with high levels of depression and anxiety, we will advise the participant to consult either with a GP or with a therapist if they have one, in relation to participating in this experiment.

Participation in this study will take approximately 3 hours over three weeks. Which is broken down into 1 hour for the intervention (the video game itself); 1 hour for online one-on-one qualitative audio interviews via Zoom after completing the game, and after a 3-week follow-up; and 1 hour for the three-questionnaire assessments at baseline (pre-intervention), immediate post-intervention, and after a 3-week follow-up.

Who is carrying out the research?

The data are being collected by Prof. Andrew Kemp and research assistant Tom Gordon of the Psychology Department within the College of Human and Health Sciences at Swansea University, as well as Dr. Darren Edwards of The Department of Public Health, Policy, and Social Sciences. The research has been approved by the Department of Psychology's Research Ethics Committee.

What happens if I agree to take part?

If you are happy to take part in this research project, please click "accept" at the bottom of the consent section below, which will then allow you to complete a baseline questionnaire assessment. The questionnaire assessment includes a range of multiple-choice questions. These questions cover a range of factors including depression, anxiety, stress, and wellbeing. There are three questionnaire assessments to complete, the first of which is a baseline assessment prior to the intervention commencing, the second will be completed following the intervention, and a follow-up assessment 3 weeks following the baseline assessment. All of the questionnaires ask the same questions with the addition of demographic questions at baseline assessment, and the second also asking about adherence to the intervention.

The intervention will consist of completing a one-hour session of playing an ACT-based video game, followed by completion of a short reflection workbook. The game itself will be completed by the participant at home, using a mobile phone or any iOS device. This is an adventure game that will teach you about psychological resilience. In the game, you will control the character Steve as he embarks on a journey into his own mind, you will help as he confronts painful past memories and learns how to cope with his full potential.

Version 4 4 November 2022

For peer review only - http://bmjopen.bmj.com/site/about/guidelines.xhtml

Swansea University
Prifysgol Abertawe

You will also be interviewed, which will be conducted online via zoom, regarding your experiences of the game, focusing on what you felt did and did not work. The interviews will be conducted at two points during the study, post-intervention, and after a 3-week follow-up. Interview audio will be recorded and transcribed, following which the recordings and any personal information will be immediately deleted.

Are there any risks associated with taking part?

We are not aware of any significant risks associated with participation. While some of the questions will ask about existing states of mental health (e.g., levels of current anxiety and depression) – which some people may find uncomfortable – the video game has developed in accordance with recent developments in psychological science to help you manage these, and we expect that participation in this study will help to ameliorate these feelings.

If you feel affected by any issues raised by this research and would like to discuss any concerns, please contact the principal investigators of this study as indicated in the contact details at the bottom of this information sheet. If you feel that you would benefit from further psychological or psychiatric support, we advise you to contact your GP (family doctor) in the first instance. Further information on mental wellbeing can be found at https://www.mind.org.uk/. Additional mental health information can be found at https://www.nhs.uk/mental-health/.

This research has been approved by the Department of Psychology's Research Ethics Committee.

Data Protection and Confidentiality

Your data will be processed in accordance with the Data Protection Act 2018 and the General Data Protection Regulation 2016 (GDPR). All information collected about you will be kept strictly confidential. Your data will only be viewed by the researcher/research team.

Standard ethical procedures will involve you providing your consent to participate in this study by ticking the consent box in the consent section

All electronic data will be stored on password-protected computers.

Please note that the data to be collected for our study will be made anonymous once you have completed all stages of the research, and your response data will not hold any personally identifiable information. We will allocate you with an identifier code, and keep your email information for the duration of the study, so that we can communicate with you at the time points. We will delete contact emails at the end of the study.

All data deposited in Swansea University's OneDrive for Business service is stored within Microsoft's data centres located in the EU. Swansea University retains full ownership and control over the data and is satisfied that the data is properly secured and protected.

The contractual agreements between Microsoft and Swansea University have been negotiated by the JISC on behalf of the UK HE sector and abide by all relevant UK and European legislation. In addition, the UK Government has granted Microsoft Azure and Microsoft Office 365 "OFFICIAL" accreditation. This means that they are accredited to hold or transact public sector data for business conducted at the OFFICIAL level of Security Classification.

What will happen to the information I provide?

Version 4 4 November 2022

For peer review only

An analysis of the information will form part of our report at the end of the study and may be presented to interested parties and published in scientific journals and related media. *Note that all information presented in any reports or publications will be anonymous and unidentifiable.*

Is participation voluntary and what if I wish to later withdraw?

Your participation is entirely voluntary – you do not have to participate if you do not want to. If you decide to participate, but later wish to withdraw from the study, then you are free to withdraw at any time, without giving a reason and without penalty (simply close your web browser). All identifying information will be stripped from collected data once you have completed all phases of the study. If you wish to withdraw at any time during the study, all personal information will be immediately deleted.

Data Protection Privacy Notice

The data controller for this project will be Swansea University. The University Data Protection Officer provides oversight of university activities involving the processing of personal data, and can be contacted at the Vice Chancellors Office.

Your personal data will be processed for the purposes outlined in this information sheet. Standard ethical procedures will involve you providing your consent to participate in this study by ticking the consent box on the consent page.

The legal basis that we will rely on to process your personal data will be processing in line with public interest, scientific and statistical purposes.

How long will your information be held?

Anonymised data will be preserved and accessible online, as is encouraged by developments in open science.

What are your rights?

You have a right to access your personal information, to object to the processing of your personal information, to rectify, to erase, to restrict and to port your personal information. Please visit the University Data Protection webpages for further information in relation to your rights.

Any requests or objections should be made in writing to the University Data Protection Officer:-

University Compliance Officer (FOI/DP)
Vice-Chancellor's Office
Swansea University
Singleton Park
Swansea
SA2 8PP
Email: dataprotection@swansea.ac.uk

How to make a complaint

If you are unhappy with the way in which your personal data has been processed you may, in the first instance contact the University Data Protection Officer using the contact details above.

If you remain dissatisfied then you have the right to apply directly to the Information Commissioner for a decision. The Information Commissioner can be contacted at: -

Information Commissioner's Office,
Wycliffe House,

Version 4 4 November 2022

For peer review only

Swansea University
Prifysgol Abertawe

Water Lane,
Wilmslow,
Cheshire,
SK9 5AF
www.ico.org.uk

What if I have other questions?
If you have further questions about this study, please do not hesitate to contact us:

Tom Gordon
Research Assistant
Swansea University
tom.gordon@swansea.ac.uk

Dr. Darren Edwards
Department of Public Health, Policy, and Social Sciences.
Swansea University
d.j.edwards@swansea.ac.uk

Prof. Andrew Kemp
Department of Psychology
Swansea University
a.h.kemp@swansea.ac.uk

For peer review only

Version 4 4 November 2022

DEBRIEF FORM

Title of project: **A novel ACT-based video game to support mental health through embedded learning**

Thank you for taking part in our research. Now that your contribution has finished, let us explain the rationale behind this work.

We are interested in how a video game based on recent developments in clinical psychology might enable learning of psychological resilience skills through play. Typically, psychotherapeutic interventions are delivered through face to face sessions, but there is an increasing need for psychological support that is delivered through an online medium such as the video you have been playing in our study.

This work therefore builds on previous efforts to teach people important skills in psychological resilience through psychoeducation and embedded learning. Specifically, the game is designed to build psychological flexibility, which is a key outcome of an ACT intervention. Psychological flexibility within ACT refers to the promotion of positive mental health, contact with present emotions, wellbeing, and positive emotions.
We hope that information gained from this study will be useful for further developing our ACT-based video game and expanding further research in this area.

If you feel affected by any issues raised by this research and would like to discuss any concerns, please contact the principal investigators of this study as indicated in the details provided below. If you feel that you would benefit from further psychological or psychiatric support, we advise you to contact your GP (family doctor) in the first instance. Further information on mental wellbeing can be found at https://www.mind.org.uk/. Further information regarding mental health can be found at https://www.nhs.uk/mental-health/.

Tom Gordon
Research Assistant
Swansea University
tom.gordon@swansea.ac.uk

Dr. Darren Edwards
Department of Public Health, Policy, and Social Sciences.
Swansea University
d.j.edwards@swansea.ac.uk

Prof Andrew Kemp
Department of Psychology
Swansea University
a.h.kemp@swansea.ac.uk

For peer review only

CONSORT 2010 checklist of information to include when reporting a pilot or feasibility trial*

Section/Topic	Item No	Checklist item	Reported on page No
**Title and abstract**			
	1a	Identification as a pilot or feasibility randomised trial in the title	1
	1b	Structured summary of pilot trial design, methods, results, and conclusions (for specific guidance see CONSORT abstract extension for pilot trials)	2/3
**Introduction**			
Background and objectives	2a	Scientific background and explanation of rationale for future definitive trial, and reasons for randomised pilot trial	4-9
	2b	Specific objectives or research questions for pilot trial	8-9
**Methods**			
Trial design	3a	Description of pilot trial design (such as parallel, factorial) including allocation ratio	9
	3b	Important changes to methods after pilot trial commencement (such as eligibility criteria), with reasons	8
Participants	4a	Eligibility criteria for participants	10
	4b	Settings and locations where the data were collected	10
	4c	How participants were identified and consented	10
Interventions	5	The interventions for each group with sufficient details to allow replication, including how and when they were actually administered	12
Outcomes	6a	Completely defined prespecified assessments or measurements to address each pilot trial objective specified in 2b, including how and when they were assessed	10-12
	6b	Any changes to pilot trial assessments or measurements after the pilot trial commenced, with reasons	N/A
	6c	If applicable, prespecified criteria used to judge whether, or how, to proceed with future definitive trial	N/A
Sample size	7a	Rationale for numbers in the pilot trial	10
	7b	When applicable, explanation of any interim analyses and stopping guidelines	N/A
Randomisation:			
Sequence generation	8a	Method used to generate the random allocation sequence	N/A
	8b	Type of randomisation(s); details of any restriction (such as blocking and block size)	N/A
Allocation concealment mechanism	9	Mechanism used to implement the random allocation sequence (such as sequentially numbered containers), describing any steps taken to conceal the sequence until interventions were assigned	N/A

For peer review only - http://bmjopen.bmj.com/site/about/guidelines.xhtml

Implementation	10	Who generated the random allocation sequence, who enrolled participants, and who assigned participants to interventions	N/A
Blinding	11a	If done, who was blinded after assignment to interventions (for example, participants, care providers, those assessing outcomes) and how	N/A
	11b	If relevant, description of the similarity of interventions	N/A
Statistical methods	12	Methods used to address each pilot trial objective whether qualitative or quantitative	14-17
**Results**			
Participant flow (a diagram is strongly recommended)	13a	For each group, the numbers of participants who were approached and/or assessed for eligibility, randomly assigned, received intended treatment, and were assessed for each objective	17
	13b	For each group, losses and exclusions after randomisation, together with reasons	17
Recruitment	14a	Dates defining the periods of recruitment and follow-up	17
	14b	Why the pilot trial ended or was stopped	N/A
Baseline data	15	A table showing baseline demographic and clinical characteristics for each group	30
Numbers analysed	16	For each objective, number of participants (denominator) included in each analysis. If relevant, these numbers should be by randomised group	17
Outcomes and estimation	17	For each objective, results including expressions of uncertainty (such as 95% confidence interval) for any estimates. If relevant, these results should be by randomised group	30
Ancillary analyses	18	Results of any other analyses performed that could be used to inform the future definitive trial	17-29
Harms	19	All important harms or unintended effects in each group (for specific guidance see CONSORT for harms)	N/A
	19a	If relevant, other important unintended consequences	N/A
**Discussion**			
Limitations	20	Pilot trial limitations, addressing sources of potential bias and remaining uncertainty about feasibility	33-34
Generalisability	21	Generalisability (applicability) of pilot trial methods and findings to future definitive trial and other studies	31
Interpretation	22	Interpretation consistent with pilot trial objectives and findings, balancing potential benefits and harms, and considering other relevant evidence	31-35
	22a	Implications for progression from pilot to future definitive trial, including any proposed amendments	34-35
**Other information**			
Registration	23	Registration number for pilot trial and name of trial registry	3
Protocol	24	Where the pilot trial protocol can be accessed, if available	35
Funding	25	Sources of funding and other support (such as supply of drugs), role of funders	35
	26	Ethical approval or approval by research review committee, confirmed with reference number	36

For peer review only - http://bmjopen.bmj.com/site/about/guidelines.xhtml

Citation: Eldridge SM, Chan CL, Campbell MJ, Bond CM, Hopewell S, Thabane L, et al. CONSORT 2010 statement: extension to randomised pilot and feasibility trials. BMJ. 2016;355. This is an Open Access article distributed in accordance with the terms of the Creative Commons Attribution (CC BY 3.0) license (http://creativecommons.org/licenses/by/3.0/), which permits others to distribute, remix, adapt and build upon this work, for commercial use, provided the original work is properly cited.

*We strongly recommend reading this statement in conjunction with the CONSORT 2010, extension to randomised pilot and feasibility trials, Explanation and Elaboration for important clarifications on all the items. If relevant, we also recommend reading CONSORT extensions for cluster randomised trials, non-inferiority and equivalence trials, non-pharmacological treatments, herbal interventions, and pragmatic trials. Additional extensions are forthcoming: for those and for up-to-date references relevant to this checklist, see www.consort-statement.org.

For peer review only

For peer review only - http://bmjopen.bmj.com/site/about/guidelines.xhtml

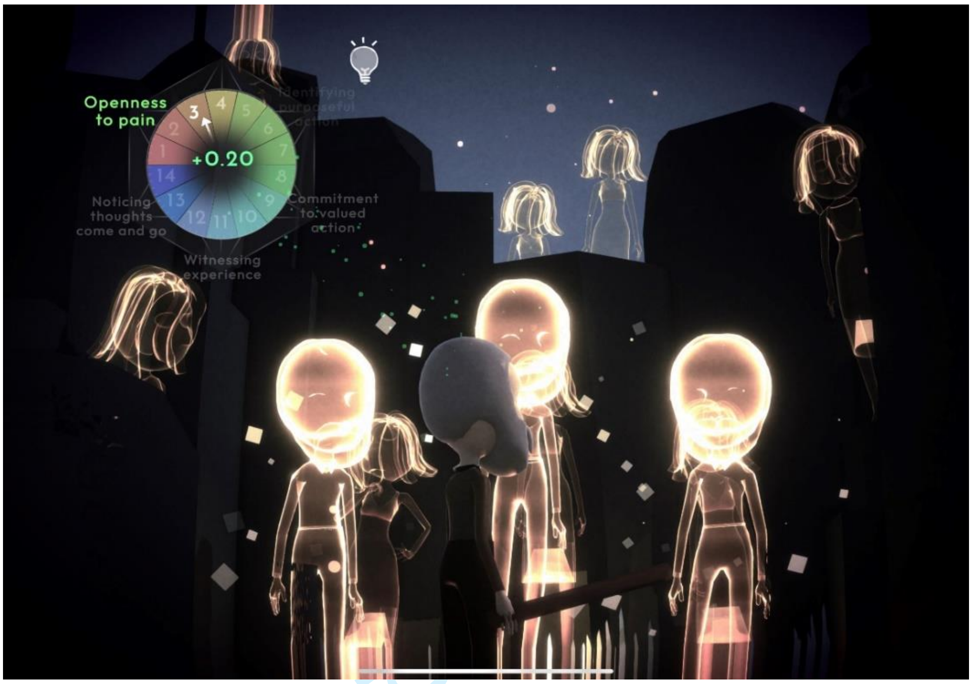
Openness to pain
+0.20
Identifying purposeful action
Commitment to valued action
Witnessing experience
Noticing thoughts come and go

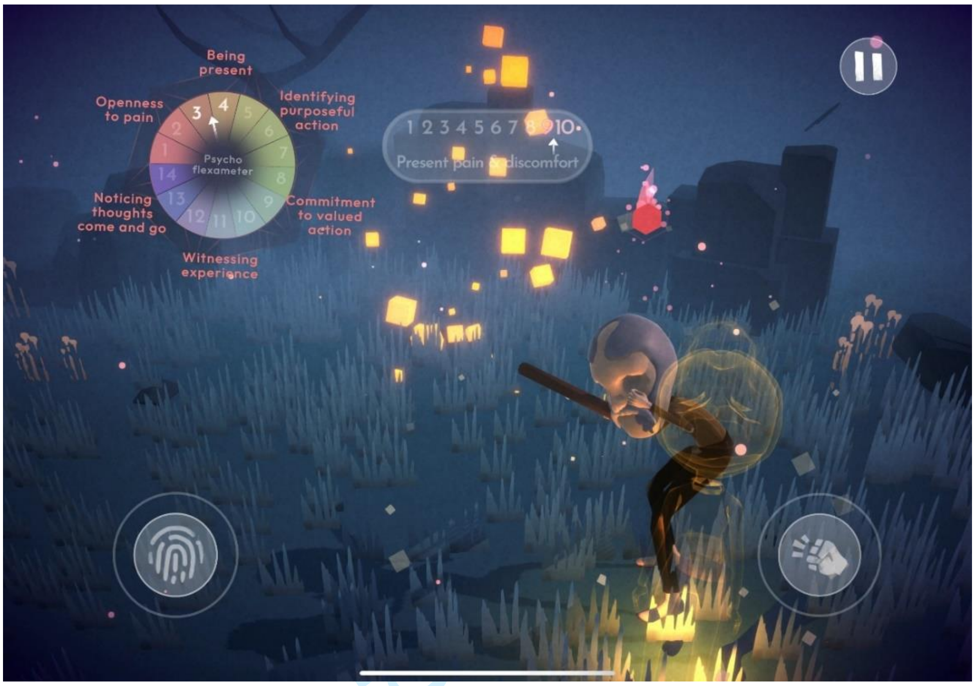

For peer review only

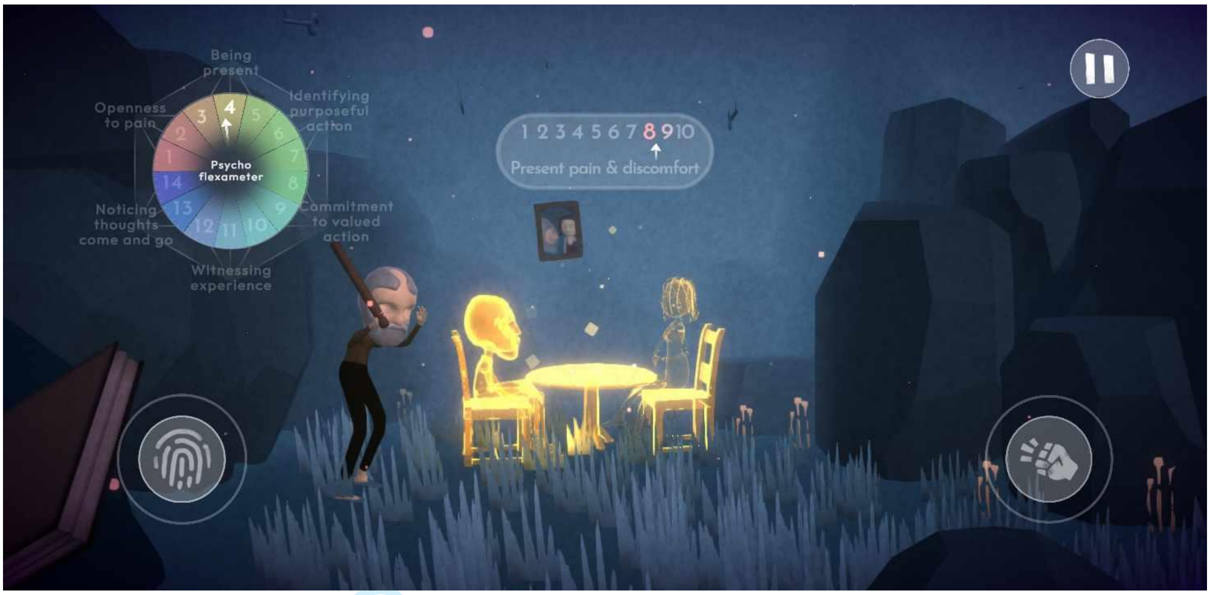
Being present
Openness to pain
Identifying purposeful action
Psycho flexameter
Noticing thoughts come and go
Commitment to valued action
Witnessing experience
1 2 3 4 5 6 7 8 9 10
Present pain & discomfort

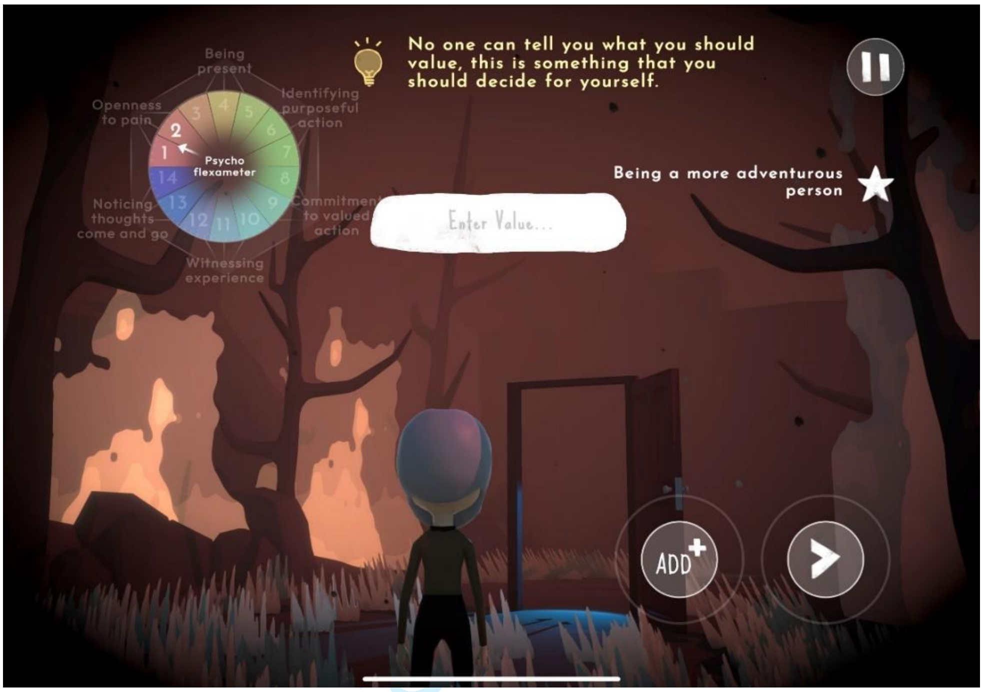
No one can tell you what you should value, this is something that you should decide for yourself.
Being present
Identifying purposeful action
Openness to pain
Psycho flexameter
Noticing thoughts come and go
Commitment to valued action
Witnessing experience
Enter Value...
Being a more adventurous person
ADD

BMJ Open

Mixed-methods feasibility outcomes for a novel ACT-based videogame 'ACTing Minds' to support mental health

Journal:	*BMJ Open*
Manuscript ID	bmjopen-2023-080972.R1
Article Type:	Original research
Date Submitted by the Author:	16-Feb-2024
Complete List of Authors:	Gordon, Tom; Swansea University, School of Psychology ; Swansea University<br>Kemp, Andrew; Swansea University, Psychology<br>Edwards, Darren; Swansea University, Department of Public Health
**Primary Subject Heading**:	Mental health
Secondary Subject Heading:	Public health
Keywords:	MENTAL HEALTH, Health Education, Depression & mood disorders < PSYCHIATRY

SCHOLARONE™
Manuscripts

I, the Submitting Author has the right to grant and does grant on behalf of all authors of the Work (as defined in the below author licence), an exclusive licence and/or a non-exclusive licence for contributions from authors who are: i) UK Crown employees; ii) where BMJ has agreed a CC-BY licence shall apply, and/or iii) in accordance with the terms applicable for US Federal Government officers or employees acting as part of their official duties; on a worldwide, perpetual, irrevocable, royalty-free basis to BMJ Publishing Group Ltd ("BMJ") its licensees and where the relevant Journal is co-owned by BMJ to the co-owners of the Journal, to publish the Work in this journal and any other BMJ products and to exploit all rights, as set out in our licence.

The Submitting Author accepts and understands that any supply made under these terms is made by BMJ to the Submitting Author unless you are acting as an employee on behalf of your employer or a postgraduate student of an affiliated institution which is paying any applicable article publishing charge ("APC") for Open Access articles. Where the Submitting Author wishes to make the Work available on an Open Access basis (and intends to pay the relevant APC), the terms of reuse of such Open Access shall be governed by a Creative Commons licence – details of these licences and which Creative Commons licence will apply to this Work are set out in our licence referred to above.

Other than as permitted in any relevant BMJ Author's Self Archiving Policies, I confirm this Work has not been accepted for publication elsewhere, is not being considered for publication elsewhere and does not duplicate material already published. I confirm all authors consent to publication of this Work and authorise the granting of this licence.

For peer review only

Mixed-methods feasibility outcomes for a novel ACT-based videogame 'ACTing Minds' to support mental health

Tom C. Gordon[1,2], Andrew H. Kemp[2], & Darren J. Edwards[1]

[1]Department of Public Health, Swansea University, UK

[2]School of Psychology, Swansea University, UK

Darren J. Edwards,

Department of Public Health,

Swansea University,

Swansea,

SA2 8PP,

UK

Email: d.j.edwards@swansea.ac.uk

Short title: Feasibility outcomes ACTing Minds

For peer review only

Abstract

Objectives: To determine the feasibility and acceptability of 'ACTing Minds', a novel single-player adventure videogame based on acceptance and commitment therapy (ACT).

Design: A single-arm, mixed-methods repeated measures feasibility study.

Setting: Intervention and questionnaires were completed at home by participants. Semi-structured interviews were also conducted at home via the Zoom platform.

Participants: Thirty-six participants were recruited into the study, 29 completed all phases of the feasibility design. Eligibility criteria required participants to be over the age of 18 and self-reporting experiencing ongoing depression, anxiety, or stress.

Intervention: Participants completed a single session of the 'ACTing Minds' videogame, lasting approximately 1-hour, designed to educate users on key principles from acceptance and commitment therapy (ACT).

Primary outcome measures: Participant recruitment and retention, questionnaire completion, long-term intervention adherence, and acceptability of the intervention. Reflexive thematic analysis was conducted on semi-structured interviews run immediately postintervention and 3-weeks later.

Secondary outcome measures: Measures of depression, anxiety, stress, psychological flexibility, social connectedness, and wellbeing were assessed at baseline, immediately following intervention completion, and after a 3-week follow-up period. We used a standardised battery of questionnaires.

Primary results: Twenty-nine participants completed the study. A reflexive thematic analysis indicated that participants responded positively to the intervention and the study at all stages. Themes reflect participants' desire for an engaging therapeutic experience, use of

For peer review only

game for exploring emotions, as well as their perspectives on how they had applied their learning to the real-world.

Secondary results: Quantitative results indicated small to large effect sizes associated with decreases in depression ($\eta p2 = .011$), anxiety ($\eta p2 = .096$), stress ($\eta p2 = .108$), and increases in psychological flexibility ($\eta p2 = .060$), social connectedness ($\eta p2 =.021$), well-being ($\eta p2 =.011$), and participation in usual activities ($\eta p2 = .307$).

Conclusions: Implementation of the 'ACTing Minds' intervention is warranted, based on both qualitative and quantitative outcomes.

Trial Registration number NCT04566042

For peer review only

Strengths and limitations of this study

- Mixed methods approach, combining thematic analysis of interviews and quantitative questionnaires.
- Collection of quantitative data at three time points and qualitative at two time points, allowing the process of change and identification of patterns to be examined.
- Remote data collection due to COVID restrictions meant that participants could not be directly observed while completing the intervention. We were also unable to record planned psychophysiological measurements of wellbeing such as heart rate variability.
- Reliance on self-report measures introduces the potential for bias.

Introduction

The global prevalence of common mental disorders and a lack of available resources for the identification and treatment of those with such conditions underpin an increasing burden on society (1). The Global Burden of Disease study conducted in 2017, reported a UK prevalence rate of 4.12% for depression, and 4.65% for anxiety disorders (2). Since this estimation, events such as the COVID-19 pandemic and the increasing threat of climate crises have had a substantial impact on societal wellbeing. A meta-analysis including 14 studies (n=46,158) found that 32% of adults in the UK experienced moderate to severe depressive symptoms in 2022, and 31% of adults reported high levels of anxiety (3), indicating a societal increase of 27.88% for depression, and 26.35% increase for anxiety between 2017 and 2022. These findings suggest the need for a transition towards population-wide strategies aimed at fostering psychological resilience on a broader scale.

To positively impact societal wellbeing, contemporary interventions must be affordable and widely accessible. Presently, the demand for mental health services far exceeds the available human resources required to meet this need. A study conducted for the Centre for Mental Health estimated that services cost the UK economy approximately £105 billion per year in 2020, 4.8% of the UK's annual GDP (4). Despite substantial funding of £34 billion to public mental health support and services, the prevalence of psychological disorders is high and only 33% of adults with depression and anxiety receive treatment in England (5), highlighting an urgent need for innovation.

There are numerous barriers to accessing psychological interventions, including a shortage of therapists, long waiting times, and societal stigma of accessing psychological treatment (6). A potential solution to these issues might be found in digital health interventions (DHIs). We live in an age of heavy digital media consumption, especially in the

For peer review only

West where at least 90% of UK adults use the internet regularly (7). We also know that during the COVID-19 pandemic, there were significant increases in online video consumption, social media usage, remote work, online news consumption, and video gaming (8). COVID-19 contributed to significant social isolation and further disconnection from nature, further contributing to increases in mental health conditions (9). We argue that there is an opportunity to leverage technological advancements and the growing use of technology to develop psycho-educational tools necessary to support mental health at scale.

DHIs have already been utilised in a variety of contexts for promoting wellbeing, from delivering healthcare and education to personalised diet and fitness plans. Mobile apps and online platforms offer guided meditation, breathing exercises, sleep tracking, and relaxation programs. Such applications might aim to enhance overall wellbeing, reduce stress, improve sleep quality, or cultivate mindfulness practices (10). We suggest that effectively addressing wellbeing at a population level should involve the development of DHIs that consider acceptability, feasibility, and widespread appeal.

Compared with alternative forms of media, videogame DHIs offer several advantages. By design they are interactive, applying behavioural principles for controlling and modifying behaviour (11), making them uniquely captivating. In the UK, the COVID-19 pandemic led to a substantial increase in the number of people playing video games, with males increasing their use from 46% to 63% and females increasing their use from 32% to 56% in 2022 (12). Innovations in the use of video games for treating mental health issues have wide potential applications, potentially offering a platform for individuals to explore their ongoing relationship with their emotions in a supportive environment.

In theory, by practicing skills derived from psychological therapies (such as ACT) within the game context, individuals can transfer these skills to real-life situations to improve their overall quality of life. Certain games specifically designed for therapeutic purposes,

For peer review only - http://bmjopen.bmj.com/site/about/guidelines.xhtml

such as ‘SPARX’ for depression (13) or ‘Elude’ for anxiety (14), guide players through interactive challenges and cognitive exercises for developing emotional regulation skills. Videogames are also being used in clinical settings to promote wellbeing outcomes. One game designed for this purpose, ‘Dojo’ (15), aims to treat anxiety by training users in breathing techniques, muscle relaxation, positive thinking, and guided imagery, utilising heart-rate variability (HRV) biofeedback. However, when compared against a standard commercial game ‘Rayman 2’ (control condition), a full pre-post RCT (n=138) found that playing either game significantly reduced participant anxiety at a 3-month follow-up, and there were no significant difference between these two games at reducing anxiety for this time period (15). Reasoning for this might be that ‘Dojo’ failed to develop the psychoeducational skills in the participants that it aimed to impart, or that both games only reduced anxiety by distracting (as a form of avoidance) participants from anxiety-provoking thoughts (16). The researchers concluded that ‘Dojo’ had crucial design issues that needed to be addressed including a lack of clear theoretical and therapeutic frameworks, and that research assessing the real-world effectiveness of video games in the treatment of mental health issues should require an appropriate methodology for understanding the underlying causes of improvement.

A study aiming to explore the wellbeing effects of playing video games on gamers during the COVID-19 pandemic (n=781) found that time spent playing had significantly increased in 71% of participants, and 58% of participants reported that playing games had positively impacted their wellbeing (17). The researchers conducted an online survey including both closed and open-ended questions, then conducted a thematic analysis to identify the causes of improvement. Themes of escape, cognitive stimulation, stress relief, agency, and socialisation were most associated with feelings that playing video games had increased wellbeing. The development of an effective DHI videogame should consider such

For peer review only

For peer review only - http://bmjopen.bmj.com/site/about/guidelines.xhtml

factors whilst also building on strong theoretical and therapeutic foundations that facilitate the uptake of such tools.

The 'ACTing Minds' videogame, developed in line with our intervention protocol (16), was designed to be a comprehensive transdiagnostic intervention that will integrate lessons from acceptance and commitment therapy (ACT) (18). In contrast to prior mentioned DHIs, commonly rooted in the medical model and second-wave approaches, ACT as a third-wave behavioural therapy focuses on promoting psychological flexibility rather than the elimination of disorder symptoms (19). More specifically, ACT aims to promote psychological flexibility through six core processes of change (20). These are: (1) present moment awareness: the practice of being in the here and now; (2) acceptance: the practice of being open to the range of human emotional experience, as opposed to experiential avoidance; (3) Cognitive defusion: the act of recognising the self as separate from thoughts, and not interpreting them literally; (4) values: identifying ones' personal values in contrast to perceived expectations, of which drive us toward self-direction and purpose; (5) action: a commitment to ones' values, facilitating the development of competence through the act of continual practice of alignment with values (21); and (6) self as context: developing an awareness of self that is more than a conceptualised sense of self, one that is flexible and facilitates a sense of connection with others.

Though ACT clinical practice does not focus primarily on reducing mental health symptoms, many studies have indicated that when the individual works towards greater psychological flexibility, many mental health symptoms, and destructive behaviours such as anxiety, depression, stress, pain, and substance misuse, tend to reduce with clinically acceptable small to high effect sizes. This was, for example, identified within a review of 20 meta-analyses, involving 133 studies (n=12,477) that found that ACT was efficacious for treating these disorders (22). The results also showed that ACT was generally superior to

For peer review only - http://bmjopen.bmj.com/site/about/guidelines.xhtml

most active intervention conditions (excluding CBT), treatment as usual, and inactive controls.

As such, an ACT-based DHI video game could allow for both greater psychological flexibility as well as a reduction in common mental health issues such as depression and anxiety. This is because by developing explicit DHI psychoeducational transdiagnostic skills that promote present moment awareness, values orientation, commitment to action, openness, and acceptance of painful emotions, cognitive defusion, and a transcendental self also have a positive impact on mental health. As a consequence, our videogame may have greater reach and impact than other videogame DHIs that are not based on third-wave psychotherapy, such as 'Dojo', which primarily aims to teach skills for limited emotional regulation and symptom reduction such as avoidance.

The ACT framework has already been adapted to a variety of accessible mediums, including self-help books and mobile phone applications (23). Resources for the education and practice of ACT are freely available through the Association of Contextual Behavioural Science website (see https://contectualscience.org/). ACT-based mobile phone applications are shown to be effective in promoting psychological flexibility (24) and reducing smoking intake (25). Considering this, we believe that choosing ACT as the basis for our game will allow us to harness the advantages of third-wave therapies as transdiagnostic therapeutic tools and integrate these with those of videogames, and if made well, will be engaging, educational, and capable of promoting wellbeing (psychological flexibility) at scale.

Based on our initial protocol (16) (see Supplementary 1), the ACT-based videogame called 'ACTing Mind', has been developed as a psychoeducational tool that teaches users the core processes of ACT through embedded learning. The goal of this research will be to determine the acceptability and feasibility of 'ACTing Minds' for promoting psychological flexibility as well as its clinical relevance for reducing mental distress as a secondary

For peer review only - http://bmjopen.bmj.com/site/about/guidelines.xhtml

outcome. The game teaches skills based on the ACT principles of acceptance, defusion, and values identification. This is a feasibility study, following the Medical Research Council (MRC) framework (26), laying the foundation for a full-scale RCT from which clinical effectiveness will be determined.

Several changes have been made to our originally published protocol (16), it was initially stated that participants would complete five weekly 1-hour sessions where they would play through six parts of the 'ACTing Minds' videogame, each one focusing on a different process of ACT. However, because of funding restrictions, 'ACTing Minds' has been compressed into a single game focusing on the ACT principles of acceptance, values identification, and defusion. Therefore, in this feasibility study, participants will be required to complete a single 1-hour session of 'ACTing Minds'. This meant a significant change to the overall time to complete the study protocol. Originally it was expected to take 3 months between baseline measurements and the final follow-up. Now, one-on-one semi-structured interviews will be conducted immediately postintervention, and after a 3-week follow-up. The research questions are as follows: Is the intervention acceptable and feasible for a full-scale RCT? Is there early evidence for effectiveness in reducing mental distress? Are there any changes in self-reported wellbeing measures following completion of the game? Are participants able to learn ACT principles and apply them in day-to-day life?

Methodology

Design:

This is a single-arm, mixed-methods repeated measures study, designed to determine the feasibility and acceptability of an ACT-based video game called 'ACTing Minds' that has been designed for individuals reporting mild to moderate anxiety, depression, and stress. Data was collected at baseline, immediately post-intervention, and 3-weeks post-intervention. Data collection was conducted between November 1st and December 31st, 2022.

For peer review only - http://bmjopen.bmj.com/site/about/guidelines.xhtml

Study Setting:

The study was conducted entirely online by participants, including the intervention (via a link to the mobile app; https://shorturl.at/iqFGI), quantitative assessment (via the Qualtrics platform), and qualitative interviews (via the Zoom platform). Strict recommendations were given to participants to ensure they were in a quiet room and without disruption for all study components.

Participants:

Thirty-six participants were recruited, 29 of which completed all phases of the study. Participants were recruited using purposive sampling methods, they were required to be at least 18 years of age, self-reporting ongoing experience of mild to moderate depression, anxiety, or stress within their day-to-day life, and able to read, write, and speak English. The sample size was justified on the basis of past research reporting the median numbers of participants recruited for similar feasibility studies incorporating both quantitative and qualitative elements (27). Advertisements were posted at Swansea University notice boards and on social media pages (Facebook mental health community groups). Participants were recruited between October 1st and December 1st, 2022, they completed a consent form (see Supplementary 2) after reading the study information sheet (see Supplementary 3) and were given a debrief sheet (see Supplementary 4) following completion of the study, each are included as supplementary materials.

Primary outcome measures:

Feasibility outcomes were determined using the MRC framework (26) and reported in line with CONSORT guidelines (28) (see Supplementary 5). Feasibility measures included data relating to participant recruitment and retention including the number of participants willing to take part, and completing each stage of the study (i.e., intervention, questionnaires,

For peer review only - http://bmjopen.bmj.com/site/about/guidelines.xhtml

interviews, and follow-up). Acceptability and efficacy of the intervention was assessed through thematic analysis of semi-structured interviews conducted immediately post-intervention and 3-weeks post-intervention, which focussed on participant experiences with 'ACTing Minds'. The first interview (see Open Science Framework for first interview protocol: https://osf.io/5fvjs) asked questions about: (1) the acceptability of the intervention; (2) what they learned from the intervention; (3) suggestions for further improvement; (4) whether there were any difficulties (barriers) in taking part; (5) aspects they liked and disliked; and (6) were there any adverse effects that they noticed whilst playing the game. This was followed by a second interview (see Open Science Framework for second interview protocol: https://osf.io/32epw) that was focused more on the real-world impact that ACTing Minds had on their lives and their experience over the three-week period. Specifically, the second interview asked: (1) about their retrospective experience in taking part in the ACTing Minds intervention; (2) how much they remember about the core ACT concepts; (3) were any aspects more memorable than others; (4) had they implemented any of the ACT concepts that they learned whilst playing the game into their day to day lives; (5) had they found that any particular ACT concepts were more applicable to their everyday lives than others; (6) would they reuse the intervention; and (7) had they noticed any adverse effects in the three weeks since playing ACTing Minds.

Secondary outcome measures:

Questionnaires were distributed at three points in time (baseline, immediate postintervention, and 3-month follow-up). A rule was created in Qualtrics requiring participants to complete every questionnaire item in order to finish the survey, which included the following questionnaires:

Depression Anxiety Stress Scales (DASS-21): a measure of general psychological distress with good construct validity (confirmatory factor analysis of 0.94), that can be

For peer review only - http://bmjopen.bmj.com/site/about/guidelines.xhtml

broken down into subscales relating to stress, anxiety, and depression. It has good internal reliability as measured through Cronbach's alpha coefficients, which are 0.88 for depression, 0.82 for anxiety, 0.90 for stress, and 0.93 for the total scale (29).

Acceptance and Action Questionnaire-second version (AAQ-II): a questionnaire of seven items, assessing psychological inflexibility by gauging one's capacity to embrace and remain receptive to challenging thoughts and emotions, while also actively participating in meaningful actions despite their presence. A higher score indicates higher psychological inflexibility. The measure has good construct validity with a Cronbach's alpha coefficient of 0.84 (30).

Social Connectedness Scale (adapted from Russell's (1996) UCLA Loneliness Scale (31): this measure involves two questions: (1) "During social interactions, I feel 'in tune' with the person/s around me", and (2) "During social interactions, I feel close to the person/s". The Cronbach's alpha coefficients for these two items ranged from 0.80 to 0.98 (M=0.94, SD=0.03) (32).

Warwick-Edinburgh Mental Well-Being Scale (WEMWBS): a metric that emphasises the positive facets of mental health, aiming to evaluate overall psychological well-being. This measure has good internal consistency with a Cronbach's alpha coefficient of 0.89 (student sample) and 0.91 (general population sample) (33).

EuroQol Five Dimensions (EQ5D) (34): a measure for health-related quality of life. There are five components within this measure which assess mobility, self-care, usual activities, pain, discomfort, anxiety, and a visual analogue scale for measuring current health status.

Intervention:

For peer review only

The intervention comprised of an ACT-based video game intervention called 'ACTing Minds', developed and designed by author DJE. Participants attended a single session lasting approximately 1-hour, during which they completed four in-game chapters.

The intervention teaches three core principles of ACT through embedded learning, meaning that the player should gain ACT-based skills while completing in-game objectives, without being directly taught those skills. These skills include 'Acceptance', 'Cognitive Defusion', and 'Values Identification'. Embedded learning refers to the incorporation of educational elements into the gameplay itself, in contrast to explicit lessons. In this context, it involves designing the game in a way that promotes psychological flexibility oriented behaviours derived from ACT, such that the adoption values orientation, present moment awareness, openness to pain and cognitive defusion. In 'ACTing Minds,' an example of embedded learning is the 'Psychoflexameter' (see Figure 1A), which serves as a gamified version of the Hexaflex (see Figure 1B), a model used in ACT to illustrate both the theory and goals for clinical change (35). Similar to the Hexaflex, the 'Psychoflexameter' showcases the 6 core processes of ACT. Initially introduced to players during the first ACT-oriented activity in the game, which emphasises acceptance, the 'Psychoflexameter' remains visible in the corner of the screen throughout the game while players engage in other ACT-related activities. As players exhibit ACT-consistent behaviours, they earn points and gradually increase the dial on the centre of the 'Psychoflexameter', and the text reflecting the ACT processes that the players use lights up green (see Supplementary 6). If players exhibit ACT-inconsistent behaviours, they lose points and the 'Psychoflexameter' lights up red (see Supplementary 7).

--**Figure 1 Here**--

The game starts with a text-based chapter, telling the story of a depressed individual, 'Steve', who has recently lost his wife in an accident, he is feeling depressed, isolated, and lonely.

For peer review only - http://bmjopen.bmj.com/site/about/guidelines.xhtml

The character has built a 'mind escape machine' intending to enter his own mind to destroy and suppress his unwanted painful thoughts and memories. The player takes control of 'Steve' in chapter two, where they see him in a state of mental distress at his home, surrounded by items that are reminders of his lost wife. At this stage, participants learn how to control the character using an onscreen directional stick and interact with the environment by touching key elements with their finger on their mobile phone or tablet.

Participants then engage with ACT content within chapters three and four, which begin with the character entering his mind (via the mind escape machine), walking around and viewing painful representations of his memories (of his lost wife). Chapter three focuses on 'Acceptance', introducing players to a bar in the centre of the screen indicating the characters' present level of pain and discomfort, as well as the 'Psychoflexameter' dial in the corner of the screen, indicating the character's psychological flexibility. While in the mind of the character, the player can approach memories of 'Steve' (himself) and his wife, which leads to an increase in present pain and allows the option to destroy the memories (this is intended as metaphorical representation of thought suppression). Destroying memories decreases short term pain and discomfort, but also removes points from the 'Psychoflexameter'. If the player chooses to destroy the memories (avoidance-based strategies), the world becomes increasingly distorted, and barriers form making the chapter impossible to complete. Alternatively, if the player chooses acceptance-based strategies they can continue the game and learn that acceptance is functionally better than avoidance (see Supplementary 8).

Chapter four focuses on rewarding 'Values identification' and 'Cognitive Defusion'. The player is still in the mind of the character, where they are then asked to reflect on their values, to type them out and make them explicit (see Supplementary 9). Following this, they complete a 'leaves on a stream' task, requiring them to type out any painful thoughts that

For peer review only

they might have and place them on a leaf, watching them as they float downstream (see Figure 1C). Both tasks reward the player by increasing their score on the 'Psychoflexameter'.

Qualitative analysis:

We used a critical realist ontological framework for our reflexive thematic analysis (RTA) as suggested by Braun and Clarke (36) which involves (after an initial familiarisation pre-coding phase) actively conducting both bottom-up (raw data driven and without a conceptual framework in mind) inductive, and top-down (ACT theory driven) deductive stages, to explore participant experience with the "ACTing Minds" intervention. We adopted this inductive (i.e., without framing the raw data through a theoretical model) first stage approach to ensure the themes developed were grounded in the raw data itself rather than potentially being imposed and biased by preconceived theories of the researcher. This is consistent with the critical realist approach which assumes that at least some of reality exists independently of our preconceived knowledge and theories, and the researcher should be actively aware of this. We then followed this with a deductive top-down ACT theory driven second stage that then allows for a more theory informed interpretation of the qualitative data based on ACT concepts and theory. This involved a re-examination of interview content with explicit consideration of how participant statements might relate to our research questions and ACT theory. The codes developed accordingly, transitioning from reflecting explicit semantic content to interpretations of underlying latent themes (via an ACT interpretation). This iteration of induction and deduction are important within RTA, as it allows for a more nuanced qualitative understanding of semi-structured interviews, that goes beyond a purely theory driven lens. This adopts a contextualist epistemological stance of our interpretation, recognising that both researchers' and participants' knowledge and perceptions are shaped by their subjective experiences and situational contexts (36). This combined (critical realist and contextualist epistemological) philosophical foundation guided the application of our

For peer review only - http://bmjopen.bmj.com/site/about/guidelines.xhtml

For peer review only

reflexive thematic analysis, serving as a contextualised lens for identifying and interpreting emergent themes from within the interview data.

The RTA was conducted on two sets of semi-structured interviews (post-intervention n=29, and 3-week follow-up n=29), following the guidelines outlined by Braun and Clarke (36). Both interview sets were analysed separately to gain an understanding of changes in participant perceptions of the intervention and relevant outcomes over time. For reporting on the acceptability of the 'ACTing Minds' intervention, findings and themes from both interviews are summarised in the primary outcomes section of the paper (see Table 1). Interview data was transcribed using Microsoft's automated audio-to-text software, which was then double checked and edited to correct for major spelling or grammatical errors. Throughout the initial data familiarisation phase, multiple points of potential analytical interest were identified. In the coding phase, several hundred codes were initially produced (both inductively and deductively), which were then clustered to make them more manageable and categorised into potential broad patterns of meaning. For the analysis of the first interview set these included: emotional experience; wellbeing needs, perceptions on mental-health education within the game; and participant engagement. For the second set of interviews, these were: application of the game's lessons, perceptions on what was learned, desire for growth, and sense of development.

Themes were then refined in the context of our research questions relating to how the participants experienced 'ACTing Minds', which involved a review of preliminary themes in relation to the codes, the coded data, and the full dataset. We became most interested in the latent ideas underpinning statements relating to how participants utilised the game as a psychoeducational tool. The preliminary theme 'emotional experience' was developed, as it was interpreted from the codes that participants were using the game as a 'base for exploring and accepting difficult emotions'. Further development of the remaining themes emphasised

For peer review only - http://bmjopen.bmj.com/site/about/guidelines.xhtml

the processes involved in participant engagement, personal therapeutic goals, and feelings regarding the games' embedded learning features. Themes derived from the codes in the second interview reflect the participant outcomes since playing 'ACTing Minds'. This allowed for an exploration of specific aspects of growth, how the participants implemented insights gained from the game in the weeks followed, and their reflections on what they had learned through the practical application of these insights in real-life scenarios.

For peer review only

Table 1.
Themes and sample codes taken from thematic analysis for Interviews 1 and 2

Interview 1 (Immediate postintervention)		
Themes	Raw-data (without preconceived theory) driven inductive codes	ACT-theory driven deductive codes
Theme 1: Desire for an engaging therapeutic experience	Need for wellbeing tools; Surprised by effectiveness; Interest in novelty; Wellbeing development as an enjoyable practice	Psychoflexameter aids engagement; Core ACT concepts useful
Theme 2: Personal process of immersion	Empathy with story; Interest in metaphor, Open mind needed; Personalisation aids relatability	Immersion through visual metaphors and agency; Engagement through ACT therapeutic intent
Theme 3: Game as a base for exploring and accepting difficult emotions	Anxiety while making decisions; Game helped to clear head; Desire for future use as a tool"; "Mood change with game;	ACT skills applicable across emotional scenarios; Long term acceptance benefit despite difficult emotional experience; learning to be open to difficult emotions
Theme 4: Embedded learning game dynamics pros and cons	Concepts made more sense as the game progressed; Lack of instruction, but quickly learned concepts; Conflicting choices	Interpreting ACT metaphors quickly; Priming of ACT-based wellbeing; ACT concepts clear despite confusion with game objectives"
Theme 5: Necessary learning for anyone	Desire to share with others; Growing societal appeal	ACT concepts made tangible; Game provided direction for growth towards ACT-values; ACT-concepts felt relevant
Interview 2 (3-weeks postintervention follow-up)		
Theme 6: Utility in the real world	Sharing lessons with others; Easier time letting go; Built desire to learn more	Applying ACT lessons actively; Potential real world subconscious influence of ACT lessons; Increased perspective-taking in real-life events
Theme 7: Practice facilitates psychological flexibility skills	New interest in wellbeing; Shift in thinking; Combined game and interview helpful	ACT-practice encourages optimism with new ACT knowledge despite present suffering; Trial and error of applying ACT-based lessons; ACT-practice encourages renewed focus on values
Theme 8: Closer alignment to an integrated self (as context), with acceptance, values, as part of who you are	Primed self-reflection; Seeing the bigger picture integration about self; Reduced self-judgment	Integrating present moment awareness, acceptance and values; Dealing with grief through acceptance of self as I am; Acceptance brings the self closer to reality; Self-assurance with values; Self as context identification

For peer review only - http://bmjopen.bmj.com/site/about/guidelines.xhtml

Quantitative analysis

A repeated measures ANOVA was performed using IBM SPSS Statistics 29 (the most up-to-date version at the time of analysis), to compare the effects of playing the 'ACTing Minds' videogame on scores taken from the questionnaires DASS-21, AAQ-II Psychological Flexibility Questionnaire, Warwick-Edinburgh Mental Well-Being Scale, Social Connectedness Score, and EuroQol Five Dimensions.

Descriptive statistics were used to summarise secondary outcome measures (see Table 2). Changes in scores from baseline are reported for each of the measurement time points. Partial eta squared (ηp2) effect sizes were calculated for each independent variable and interpretation was informed by prior literature on the topic (37). Values 0.14 or higher were interpreted to be a large effect, 0.06 – 0.14 were interpreted to be a moderate effect, and 0.01 – 0.06 were interpreted to be a small effect.

Procedure

After recruitment (see 'Participant' section for recruitment), and consenting to take part in the study, participants were given a link to Qualtrics where they completed the battery of questionnaires (see 'Secondary outcome measures' section) at baseline. They were then given a link to the ACTing Minds game (see 'Intervention' section) where they completed this within approximately one hour. Once completed, they then immediately completed the quantitative questionnaires (see 'Secondary outcome measures' section) again on Qualtrics as an immediate post-intervention. This was then followed by completing a 45-minute to 1-hour one-on-one interview which asked participants about their experiences with the game (see 'Primary outcome measures' section). Participants then, after a 3-week follow-up, completed the same questionnaires again, as well as a second interview (see 'Primary outcome measures' section) that focus on real world application of the ACTing Minds intervention.

For peer review only

For peer review only - http://bmjopen.bmj.com/site/about/guidelines.xhtml

Public and patient involvement

Key stakeholders were consulted and involved in the development of this protocol. The Patient Experience and Evaluation in Research (Patient Experience and Evaluation in Research (PEER): https://www.swansea.ac.uk/humanandhealthsciences/research-at-the-college-of-human-and-health/patientexperienceandevaluationinresearchpeergroup/) group in the College of Human and Health Sciences at Swansea University were consulted. This group represented members of the public, students and staff members, several of whom reported that they had experienced depression, anxiety or stress at some point in their lives and emphasised the need for innovative approaches of the delivery of mental health support. The feasibility design was explained to them, and they gave positive feedback about the nature of the design, intervention and outcome measures.

Results

Primary outcome measures

Participant recruitment and retention (feasibility)

Thirty-six participants were recruited through the initial study advertisement between October 1st and December 1st, all of which met eligibility criteria. Six participants did not show up for initial baseline measures, while one participant did not follow through with the intervention (see Figure 2). Only three participants reported why they were not able to attend, where one indicated they had a hospital appointment, another had forgotten about the study date and did not reschedule, while another said they needed to reschedule without giving a reason, but then failed to book in a new date for the study. The other three participants did not report why they could not attend.

---**Figure 2 Here**---

For peer review only

Participant feedback (acceptability)

Acceptability measures were assessed through thematic analysis of semi-structured interviews (all data is available on the Open Science Framework: https://osf.io/3wuh5/) (38), taken place immediately postintervention and at a 3-week follow-up. The interviews were analysed separately to understand participant perceptions before and after applying lessons learned from the game to their everyday lives. The results from both have been summarised and reported together as key themes.

Theme 1: Desire for an engaging therapeutic experience

Participants expressed interest in the novelty and potential utility of digital mental health applications, specifically in a gamified context. Overall, they felt that a videogame platform provided a uniquely stimulating means of engaging with mental health learning. Participants suggested that psychoeducational tools like 'ACTing minds' may be able to function as an engaging alternative, or an adjunct to therapy. For example:

"[…] playing a game that's based for your mental health is really good because you can actually do something therapeutical in the things that I like doing." -P06.

This quote comes from a participant who identifies as a gamer, with little experience or prior interest in mental-health learning. Their statement implies an underlying desire for an engaging, accessible therapeutic environment that he has an interest in. The intervention was also helpful for non-gamers, for example:

"I'm not one to sit down and just watch a video and then put that in practice. I'm someone who actually needs to be involved in something and interact with it, and course work just reminds me too much of school [...] with games it makes me sit, and actually interact because I'm enjoying the game at the same time. It's not just that I need to focus on my mental health,

For peer review only - http://bmjopen.bmj.com/site/about/guidelines.xhtml

I get to play a cool game while learning about my mental health. So yeah, I like I like video games with mental health stuff." -P08

In this situation the participant does not identify as a gamer and has substantial experience with their own mental health learning and therapy. They, like participant one, also express the same desire for a more engaging therapeutic experience and enjoyed the format of learning about mental health via a game. The participant expresses an aversion to learning about mental health in other more traditional ways.

Theme 2: Personal process of immersion

Responses from the semi-structured interviews highlighted the individual differences between participants in their ability to immerse themselves within the content of 'ACTing Minds' and engage directly with the personal decision-making aspects of the game. Responses indicated that the video game immersion and narrative helped them visualise the ACT metaphors and thus helped their understanding of the ACT concepts within the embedded learning environment. For example:

"It plays in a very good way of dealing with visual metaphor, but also the fact that you're given the agency to do it. When you were smashing the memories and I wish I didn't have to, but I have to carry on Steve's journey and I think it's a really good way of showing these memories do hurt, and sometimes you just have to accept the fact that it's going to hurt. Move on like it's explained through the visual and narrative storytelling, and I think it's a really good way to do it." -P18

This participant refers to the visual immersion and having agency within the game as facilitating their learning of ACT concepts. The requirement for participant agency in making emotionally difficult decisions within the game appears to have facilitated the learning of acceptance.

For peer review only - http://bmjopen.bmj.com/site/about/guidelines.xhtml

Learning was also facilitated by the personalised nature of the game, such as selecting difficult personal thoughts in the lily pad exercise, for example:

"[...] it's been like a learning experience, and so the sort of personalised bit at the end with the lily pads and the values. I think it was quite good because then you actually were able to think about like the purpose of the game within your own situation." -P10

Theme 3: Game as a base for exploring and accepting difficult emotions

Participants discussed the idea that the game provided a platform which allowed them to explore their own emotions in an immersive environment. Participants reported experiencing a variety of difficult thoughts and emotions throughout playing the game, and that the game encouraged them to observe and be open to sadness, anxiety, and grief with acceptance. Participants reported feeling that after playing the game they had learned to observe and accept those feelings rather than to actively avoid them, and the importance of values. For example:

"*At least the main bit that I got from it was to first just observe some of the negative feelings that you have, and not necessarily like reject and wrestle with them, just to sit and watch them and observe them, and accept them [...]. You can perhaps do something like remind yourself of values. I like that one too." -P05*

The participant clearly reflects that the game encouraged them to observe and accept their thoughts and feelings rather than suppress (or "wrestle") them. They also mention that the game reminded them of what really matters to them.

Participants appreciated the leaves of the stream exercise as promoting acceptance of difficult thoughts, for example:

For peer review only

"I'd like the Lily pad thing. I think it's really nice because I myself struggle with my emotions or bad thoughts or whatever, and I stay on them, and I make myself feel guilty about situations. So, it was really nice to accept letting go of yourself." -P06

Other participants reflected on the game encouraging acceptance and observation of difficult thoughts, for example:

"I think that section when it's like acceptance and realising that you know, even though you may not like these thoughts you're having, you still need to be aware of them and you can use them as a springboard so that was my favourite section." -P18

Theme 4: Embedded learning pros and cons

Data from the immediate post-intervention interviews suggest a mixed response to embedded learning in 'ACTing Minds'. Many participants reported learning a variety of lessons through playing the game, most being intended in the game's design. Participants were sometimes confused with the lack of explicit objectives but were still able to understand that there were consequences to avoidant behaviour, and that acceptance of difficult emotions was rewarded. This indicated the embedded learning within the game dynamics were successfully implemented. For example:

"So when you hit them [memories], the like walls go up right, yeah?[…] But I guess it was like kind of showing you that if you hit the memories, you kind of close yourself off and that's what the walls were." -P14

Another participant also suggested that they were able to learn relevant ACT concepts quickly such as not to avoid (suppress) thoughts, for example:

For peer review only - http://bmjopen.bmj.com/site/about/guidelines.xhtml

"*I think the fact that I think it was very quickly aware that you shouldn't just destroy memories and I think that was that was done really well. Maybe too well. As in like it went on a bit.*"

Theme 5: Necessary learning for anyone

Several participants expressed that they felt what they learned via ACTing Minds would be applicable and useful to many others in society, and not just for themselves. For example:

"I think it's a good thing that more people are learning about this kind of thing and. It kind of just leads into more research regarding and. More support out there and more help. It's generally like, I think. Maybe not my thing, but. It's not like a bad thing, it's. A good thing there's people doing it." P27.

"*I think like the impact did still stick with me like I still mentioned it quite a bit to like my parents afterwards and I mentioned it to my partner as well. They seemed interested and they like. I wish I could have done that too."-P18.*

Theme 6 (3-week follow-up interview theme): Utility in the real world

In the interview occurring 3-weeks postintervention, participants discussed how they were able to apply the lessons that they took from 'ACTing Minds' into the real-world. Whilst a few participants had not considered the game since playing or tried to actively apply lessons, many found that they made a conscious effort to apply ACT principles and techniques from the game into their lives. Some example quotes and descriptions are given below:

"I have noticed I've actively now, if I get like a negative thought in my brain. I try and register it and I don't hold on. But like, because sometimes before, even though I kind of subconsciously did it with some things, I just didn't [always], you know. But now if it's even something stupid like I've been lazy. I was like alright don't think about it all day. Actually go

For peer review only - http://bmjopen.bmj.com/site/about/guidelines.xhtml

out for a walk. Go to the gym you know. Don't just keep in your head, I'm so lazy and miserable and fat. Get out to do something about it." -P04

This statement was given by a participant who in the prior immediate-postintervention interview felt that they did not have a problem with holding on to difficult thoughts. Despite this, in the weeks since playing 'ACTing Minds', the participant found that they were more conscious of how their thoughts have impacted their real life, and as a result, have been able to apply the core ACT concepts in tandem. Their statement indicates that they were able to apply defusion lessons to difficult thoughts about their self ("I'm so lazy and miserable and fat"), acceptance of their behaviours ("I've been lazy"), and commitment to values by taking part in ACT-oriented activities regardless of their thoughts. Many participants noted that even if they had not explicitly tried to apply the ACT principles learned from the game to real life, that they felt the game had influenced them subconsciously:

"I wouldn't necessarily say I've sat there and primed those thoughts [regarding ACT concepts], but in unconscious thinking, if you know like passive thought and stuff like that in my day-to-day, I've definitely had hints of some of those topics. Do you know what I mean? Like even today, I was going about my day doing my thing and you know you'll have a thought that'll throw you back to the past, and then you learn like I came to accept it." -P11

This participant found that the ACT principles were more readily available to them when confronted with real-life situations that demanded them. In these cases, participants most commonly felt that they were more accepting of difficult emotions and situations.

Theme 7 (3-week follow-up interview theme): Practice facilitates psychological flexibility skills

A core theme present across follow-up interviews was that participants expressed how applying what they had learned into their day-to-day activities over three weeks led to an

For peer review only - http://bmjopen.bmj.com/site/about/guidelines.xhtml

even deeper understanding of the ACT concepts they learned. Participants reported greater engagement with values-orientated behaviours in their everyday real lives, and a greater ability to cognitively defuse (or let go) of difficult thoughts rather than engaging in avoidant behaviour. Some example quotes and descriptions are given below:

"Well, it's like actually testing on a real situation as to just generally learning it. But then when you come into a situation, you start to understand a bit better why you do those things [referring to ACT skills] and what benefit those things have, because the situation is actually impacting on your emotions or your feelings and stuff like that, and so then you're like oh, this is why this is a good technique." -P05

In practicing real-life application of ACT principles, participants were able to get a deeper sense of how and why the ACT techniques worked for them, especially during emotionally challenging situations. Through such practice, participants have noted that their personal values have become clearer to them:

"I've learned ways to engage with my thoughts, and like I've always tried to practice letting go of things that aren't like too meaningful, like things that won't matter in a day and all that sort of stuff. But I feel like the game has helped me also realise [...] a way to really put my values down in a more straightforward manner." -P13

The participant refers to the real life practicing of letting go of difficult thoughts (cognitive defusion), and values identification.

Theme 8 (3-week follow-up interview theme): Closer alignment to an integrated self (as context), with acceptance, values, as part of who you are

One of the most consistent patterns across interview responses was that participants felt they had learned more about themselves through acceptance, or that practicing the lessons taught in the game helped them align to their values. Some participants seem to have expressed that

For peer review only - http://bmjopen.bmj.com/site/about/guidelines.xhtml

they learned the ACT-concepts in a more integrated way, where they felt that acceptance of difficult thoughts and their values was part of who they were i.e., self as context. Some example quotes and descriptions are given below:

"I actually learned a lot from this game about like these inner emotions or bad memories are not sinful. They're a part of you, and they contribute what you're going to be or the current you [self]. So yeah, that's the core lessons I guess I learned from the game." -P23

The participant reflects here about accepting their personal experiences in the present moment for what they are (indicating broader integrated acknowledgements about themselves in context). For other participants, this acceptance of personal experiences in day-to-day life has facilitated further identification of personal values:

"Yeah, as I said, the values that's definitely helped me. Learning to just calm my thoughts for a little and think of the small but important things in life and what I appreciate that helps. It's also made me learn more about how to deal with grief [...]It's helped to learn that it's OK to feel grief, it's a part of who I am, and I must accept it -P08

Secondary outcome measures

Quantitative results (all data is available on the Open Science Framework: https://osf.io/3wuh5/) (38) revealed a large effect size for the EQ5D usual activities score ($\eta p2$ = .307), while medium effect sizes were found for DASS-21 Stress scores ($\eta p2$ = .108), DASS-21 Anxiety scores ($\eta p2$ = .096), and AAQ-II Psychological Flexibility scores ($\eta p2$ = .060). Small effect sizes were obtained for the DASS-21 Depression scores ($\eta p2$ = .011), the WEMWBS ($\eta p2$ =.011), UCLA Social Connectedness Scale ($\eta p2$ =.021), EQ5D Pain/Discomfort ($\eta p2$ =.010), and the EQ5D Anxiety/Depression ($\eta p2$ = .018). Given this is a feasibility study that is intentionally underpowered as it has a small sample size, *p* value significance are statistically meaningless for measuring efficacy of any given measure.

For peer review only - http://bmjopen.bmj.com/site/about/guidelines.xhtml

Instead, the effect sizes allow for a G-Power (39) a-priori analysis to be conducted that indicate the sample size required to detect meaningful statistical between group differences within a future full scale RCT. For this, the G-Power (version 3.1.9.7) indicated that when assuming a between factor (two group), repeated measures (three points in time) design with an alpha error probability of 0.05, and acceptable power of 0.8 (40), then 436 participants are required to detect a meaningful statistical difference for the smallest measure effect size (DASS-21 depression) in a future RCT. See Table 2 for full details including effect sizes, power, and estimated sample sizes for a future RCT given the observed effects sizes of this feasibility study.

Convergent outcomes

Integration of results are considered a defining feature of mixed-methods research (41). In the interest of transparency, we have included a visualisation of the research outcomes taken from our qualitative and quantitative approaches, illustrating how each outcome links back to feasibility, as well as the conclusions made following each outcome (see Figure 3).

For peer review only

For peer review only - http://bmjopen.bmj.com/site/about/guidelines.xhtml

Table 2.
Illustrating change in intervention outcomes over time (n=29)

	Pre-intervention Baseline, mean (SD)	Post-intervention, mean (SD)	Follow-up (3 weeks), mean (SD)	ηp2	Effect size	*F*	Power	Full RCT Sample size estimated assuming 0.8 power
DASS-21 Depression	14.34 (5.97)	14.21 (5.54)	13.90 (4.75)	.011	Small	.31	0.43	436
DASS-21 Stress	15.34 (4.55)	14.52 (4.40)	14.28 (4.65)	.108	Medium	3.39	0.999	46
DASS-21 Anxiety	12.79 (5.19)	12.10 (4.42)	11.66 (4.75)	.096	Medium	2.98	0.999	52
AAQ-II (Psychological Flexibility)	27.34 (10.47)	28.38 (9.86)	28.86 (8.65)	.060	Medium	1.76	0.999	86
WEMWBS	42.07 (7.31)	42.62 (7.91)	42.07 (7.00)	.011	Small	.31	0.33	436
UCLA Social-Connectedness	64.72 (7.52)	64.24 (8.85)	63.90 (8.33)	.021	Small	.60	0.77	236
EQ5D Mobility	4.83 (.38)	4.83 (.38)	4.83 (.38)	.000	N/A	.00	0.05	Negligible (no effect)
EQ5D Self-Care	4.78 (.58)	4.79 (.49)	4.76 (.51)	.000	N/A	.00	0.05	Negligible (no effect)
EQ5D Usual Activities	4.14 (.79)	4.38 (.68)	4.62 (.68)	.307	Large	12.42	1.00	16
EQ5D Pain/Discomfort	4.24 (.64)	4.45 (.63)	4.31 (.81)	.010	Small	.28	0.25	520
EQ5D Anxiety/ Depression	3.86 (.99)	4.03 (.87)	3.97 (.94)	.018	Small	.52	0.66	288
EQ5D Self-Rated Health Score	71.14 (19.01)	72.76 (19.57)	70.93 (20.71)	.000	N/A	.00	0.05	Negligible (no effect)

---Figure 3 Here---

Discussion

Statement of principal findings

The overarching aim of this research was to test the feasibility and acceptability of the 'ACTing Minds' video game as a DHI for treating depression, anxiety, and stress. Participant recruitment and retention, as well as quantitative and qualitative results, demonstrate that the study design and videogame intervention are feasible for testing in a full-scale RCT. Thematic analysis on qualitative data revealed several key findings. Firstly, participants were successfully able to learn about core ACT principles through embedded learning, specifically acceptance, defusion from thoughts, and commitment to personal values. There was also some indication that participants had learned something about themselves in an integrated way, in the form of self-as-context. Participants felt that the lessons taught within the game could be applied to their daily lives and that the game was effective in priming them to consider the core ACT principles throughout the weeks following their completion of the intervention. Participants also felt that they would recommend the game to someone that they care about and that they would be interested in downloading and completing future releases of 'ACTing Minds'.

Summary of secondary outcomes

Quantitative analysis of measures taken at baseline, immediately post-intervention, and after a 3-week follow-up, revealed promising small to large effect sizes in many of the quantitative measures (38). This included a large effect size for increasing EQ5D usual activities, medium effect sizes for reducing DASS-21 stress and anxiety, as well as increased AAQ-II psychological flexibility. Small effect sizes were observed for reducing DASS-21 depression, EQ5D pain and discomfort, EQ5D anxiety and depression, and increased UCLA social connectedness and WEMWBS general wellbeing. There were no observed effects for EQ5D mobility, self-care, and self-rated health score. Given this is a feasibility study that is intentionally underpowered as it has a small sample size, *p* value significance are statistically meaningless for measuring the efficacy of the interventions, and instead effect sizes are more

For peer review only - http://bmjopen.bmj.com/site/about/guidelines.xhtml

informative as they express the underlying effect and are not influenced by population size. Instead, the G-Power analysis provided an estimation of participants required (436 to account for the small effect sizes) to identify meaningful significance in a full scale future RCT.

Comparison to existing literature

This is the first study to utilise a videogame Digital Health Intervention (DHI) rooted in third-wave behavioural therapy to address mental distress. 'ACTing Minds' imparts psychological skills based on ACT to promote psychological flexibility. Prior research has primarily focused on videogame DHIs targeting ‘illbeing’ that aim to reduce symptoms of mental illness, two examples are games 'REThink' and 'Dojo'. 'REThink,' designed for a younger audience, developed the players’ ability to discern functional emotions from maladaptive ones, and was shown to effectively improve emotional symptoms and reduce depressive mood (42). 'Dojo,' utilising biofeedback and relaxation techniques to promote emotional regulation, was shown to significantly decrease participants’ anxiety and aggressive behaviour scores post-intervention but exhibited no long-term effects at a 4-month follow-up (43). Our DHI differs fundamentally from these other games due to strong theoretical underpinnings based on ACT for promoting psychological flexibility instead of a focus on reducing unwanted emotions or emotional regulation. Participants within the game were taught to observe and be open to emotional pain without judgement or any attempt to change them, and this is a key focus within the ‘ACTing Minds’ intervention.

Several meta-analyses have been conducted to explore the potential efficacy of DHIs, one found that the majority of DHIs were based on Cognitive Behavioural Therapy (CBT) and that the effect size for such interventions was small for reducing depressive symptoms compared to non-treatment controls (44). CBT-based DHIs usually do not take the form of full video games but may include elements of gamification such as rewards, badges, and progress tracking. They are typically structured programs including online education tools,

For peer review only

interactive exercises, and self-assessment tools, which focus on challenging and modifying negative thoughts and behaviours. A meta-analysis including 34 RCTs (17 of which were CBT-based) found that CBT-based DHIs yielded a medium effect size for reducing symptoms of depression and anxiety (45). However, a meta-analysis of 117 CBT-based applications revealed that only 12 of them provided support aligned with the evidence-based tenets of CBT (46). This finding suggests that the observed effectiveness in earlier studies could potentially stem from participants' interaction with a DHI (perhaps as a form of distraction) rather than their proficient implementation of CBT principles. CBT-DHI programs often require consistent use, and high attrition rates have limited their efficacy in research (47). One meta-analysis author suggested that DHIs may need to be complemented by existing mental health support (44). However, our study challenges this notion. In less than one hour of playing 'ACTing Minds', participants fully explored the game, retained ACT knowledge, and discussed its positive real-world application in interviews conducted 3 weeks post-intervention.

Strengths and limitations

Our study is the first to explore the feasibility and acceptability of a novel videogame DHI based on ACT. A core strength of this research was the utilisation of a mixed-methods approach. By incorporating thematic analysis of semi-structured interviews as well as quantitative analysis of questionnaire data, we were able to gain a comprehensive understanding of participant experience using 'ACTing Minds'. Collecting quantitative data at three separate time points, and interviews conducted at two separate time points meant that we were able to examine the gradual processes of change and identify patterns of improvement consistent with the ACT model. We gained valuable input from participants in terms of suggestions for improving the intervention which will aid in the further development of 'ACTing Minds' to optimise effectiveness and user engagement. The results from the

For peer review only - http://bmjopen.bmj.com/site/about/guidelines.xhtml

interviews also indicated that 'ACTing Minds' has broad appeal as a videogame even to those outside of clinical populations.

It is also important to acknowledge the limitations of our study. Firstly, there was no control group, though this is intentional as this is a feasibility study, it is only when we conduct a full RCT that we will have an adequate comparator group to determine whether the intervention is clinically useful at promoting psychological flexibility and reducing depression, anxiety, and stress. There was a lack of in-game data logging of specific ACT tasks completed. For example, we did not collect data on whether participants actually entered text about the difficult thoughts that they were experiencing such as in the leaves on a steam exercise, though all participants completed the game. The reliance on self-report measures potentially allowed for biased responses, including psychosociological measures such as HRV would strengthen the study outcomes. It is also possible that some of the questionnaires used in this study were insufficient for capturing the target measurements. One study used exploratory factor analysis to investigate the extent to which the AAQ-II Psychological Flexibility Questionnaire can discriminate between experiential avoidance and psychological flexibility. The researchers found that AAQ-II items were more strongly related to items measuring distress than items measuring acceptance (48). In line with this, the AAQ-II has been criticised as being too simple a measure for psychological flexibility (49). In a future RCT, we may adopt another measure for measuring psychological flexibility such as The Personalised Psychological Flexibility Index which may be a more valid measure of psychological flexibility (50). Finally, though thematic analysis can be highly useful for identifying shared meaning and variation amongst the themes, and bridging subjectivity and theoretical structure, it also has limitations. The contextualist epistemology used in this approach acknowledges the researcher has an active role in shaping the outcomes and can be biased by their own knowledge and experience. The subjective nature of the themes means

For peer review only

that there can be variation in the interpretation of the data between different thematic researchers. So, though this approach can be useful, these limitations also need to be acknowledged.

Clinical implications and directions for future research

The ACT-based videogame DHI used in this study is a low-cost, engaging, and easy-to-disseminate means of supporting those experiencing mental health difficulties. The present study highlights the clinical implications of 'ACTing Minds', including its potential therapeutic value, user engagement, and accessibility. However, further research is warranted to establish long-term effects, explore specific populations, conduct comparative studies, investigate underlying processes, and address any ethical considerations that may arise. Critically, a full RCT is now needed, in which participants are compared quantitatively with a control group, incorporating physiological wellbeing measures such as HRV, as well as research-validated questionnaires regarding mental health (i.e., depression, stress, anxiety, psychological flexibility, social connectedness, and wellbeing). By pursuing these future research directions, we can leverage the potential of ACT-based video games such as 'ACTing Minds' to enhance patient care, improve outcomes, and expand the reach of interventions in an increasingly digital era.

Conclusion

The results of this study demonstrate that 'ACTing Minds' is feasible to implement in a full-scale RCT. Both the intervention and study were well received by participants, thematic analysis of semi-structured interviews indicated that a single playthrough of the game was sufficient for teaching several core principles of ACT, namely 'Acceptance', 'Values Identification', and 'Cognitive Defusion' to participants, and priming them to implement the lessons in their day-to-day lives. There was some evidence that participants also integrated

For peer review only - http://bmjopen.bmj.com/site/about/guidelines.xhtml

their learning about themselves as self-as context, which is interesting. Quantitative results indicate that playing 'ACTing Minds' is associated with decreases in depression, anxiety, and stress, as well as increases in psychological flexibility, social connectedness, and wellbeing. However, these effects will need to be further explored in an adequately powered RCT to understand the potential clinical implications, therapeutic value, user engagement, and accessibility of an ACT-based video game DHI.

Data availability statement

The datasets supporting the conclusions of this article are available in the Open Science Framework (38): https://osf.io/3wuh5/?view_only=7998f1d0ae4b473ab7089a71be710270.

Original published protocol

https://bmjopen.bmj.com/content/10/11/e041667

Contribution statement

DJE and AHK designed the original protocol, whilst TCG updated and revised the protocol design. TCG wrote the first draft of the paper and conducted all of the quantitative and qualitative results. DJE and AHK provided substantial revisions on all drafts and advised TCG throughout the development of this manuscript. DJE designed and developed the game development.

Competing interests

The game ACTing Minds was developed using European development funds via the commercial entity of Swansea University (AgorIP) and awarded to DJE with the intention to develop this game for commercial purposes (as a game app for the Apple and Google Play stores). DJE was involved in the design of the protocol but did not recruit participants, collect any data, and did not conduct the analysis on the data. TCG and AHK have no involvement in any commercial aspects of the game.

Funding

For peer review only

Funding for the development of this game came from European development funds via the commercial entity of Swansea University called AgorIP (Reference: 229-0256-0046) awarded to DJE.

Acknowledgements

We would like to thank Miricle Tea Studios Ltd and Mikoshi Ltd for the production and release of the ACTing Minds videogame. We would also like to thank Prof. Louise McHugh (University College Dublin), Dr. Alison Stapleton (Dublin Business School), and Dr. Sarah Cassidy (Smithfield Clinic) for several helpful comments on various aspects of the ACTing Minds intervention and methodology.

Ethics statement

This study has received ethical approval from the Swansea University School of Psychology ethics sub-committee (2022-5630-4834).

Figure legends

Figure 1.

(a) Screenshot from 'ACTing Minds' showing the 'Psychoflexameter'. (b) The Acceptance and Commitment Therapy Hexaflex and core processes. (c) Screenshot from 'ACTing Minds', example of 'Cognitive defusion' task. The player is required to type in their own difficult thoughts, before dragging them on to a leaf that floats downstream.

Figure 2.
Flow of participants through the study.

Figure 3.
Diagram visualising the integrated outcomes of 'ACTing Minds' feasibility and acceptability study.

References

1. Fineberg NA, Haddad PM, Carpenter L, Gannon B, Sharpe R, Young AH, et al. The size, burden and cost of disorders of the brain in the UK. Journal of Psychopharmacology. 2013;27(9):761-70.

For peer review only

2. GBoDC N. Global Burden of Disease Study 2017 Results. Seattle, United States: Global Burden of Disease Collaborative Network. 2018.

3. Dettmann LM, Adams S, Taylor G. Investigating the prevalence of anxiety and depression during the first COVID-19 lockdown in the United Kingdom: Systematic review and meta-analyses. British Journal of Clinical Psychology. 2022;61(3):757-80.

4. Flynn D, Moloney E, Bhattarai N, Scott J, Breckons M, Avery L, et al. COVID-19 pandemic in the United Kingdom. Health Policy and Technology. 2020;9(4):673-91.

5. McManus S, Bebbington PE, Jenkins R, Brugha T. Mental health and wellbeing in England: the adult psychiatric morbidity survey 2014: NHS digital; 2016.

6. Salaheddin K, Mason B. Identifying barriers to mental health help-seeking among young adults in the UK: a cross-sectional survey. British Journal of General Practice. 2016;66(651):e686-e92.

7. Serafino P. Exploring the UK's digital divide. Office for National Statistics. 2019.

8. Lemenager T, Neissner M, Koopmann A, Reinhard I, Georgiadou E, Müller A, et al. COVID-19 lockdown restrictions and online media consumption in Germany. International journal of environmental research and public health. 2021;18(1):14.

9. Talevi D, Socci V, Carai M, Carnaghi G, Faleri S, Trebbi E, et al. Mental health outcomes of the CoViD-19 pandemic. Rivista di psichiatria. 2020;55(3):137-44.

10. Murray E, Hekler EB, Andersson G, Collins LM, Doherty A, Hollis C, et al. Evaluating digital health interventions: key questions and approaches. Elsevier; 2016. p. 843-51.

11. Linehan C, Kirman B, Roche B, editors. Gamification as behavioral psychology. The gameful world: Approaches, issues, applications; 2015: MIT Press.

For peer review only

12. Ofcom. Gaming penetration in the United Kingdom (UK) from 2013 to 2022, by age group and gender 2023 [cited 2023 June 19]. Available from: https://www.statista.com/statistics/300513/gaming-by-demographic-group-uk/.

13. Kuosmanen T, Fleming T, Newell J, Barry M. A pilot evaluation of the SPARX-R gaming intervention for preventing depression and improving wellbeing among adolescents in alternative education. Internet Interventions. 2017;8:40-7.

14. Rusch DC, editor " Elude" designing depression. Proceedings of the International Conference on the Foundations of Digital Games; 2012.

15. Scholten H, Malmberg M, Lobel A, Engels RC, Granic I. A randomized controlled trial to test the effectiveness of an immersive 3D video game for anxiety prevention among adolescents. PloS one. 2016;11(1):e0147763.

16. Edwards DJ, Kemp AH. A novel ACT-based video game to support mental health through embedded learning: a mixed-methods feasibility study protocol. BMJ open. 2020;10(11):e041667.

17. Barr M, Copeland-Stewart A. Playing video games during the COVID-19 pandemic and effects on players' well-being. Games and Culture. 2022;17(1):122-39.

18. Hayes S, Strosahl K, Wilson K. Acceptance and commitment therapy: American Psychological Association. Washington, DC[Google Scholar]. 2009.

19. Hayes SC, Hofmann SG. The third wave of cognitive behavioral therapy and the rise of process-based care. World psychiatry. 2017;16(3):245.

20. Hayes SC, Strosahl KD, Wilson KG. Acceptance and commitment therapy: The process and practice of mindful change: Guilford press; 2011.

21. Hayes S. A Liberated Mind: The essential guide to ACT: Random House; 2019.

For peer review only

For peer review only - http://bmjopen.bmj.com/site/about/guidelines.xhtml

22. Gloster AT, Walder N, Levin ME, Twohig MP, Karekla M. The empirical status of acceptance and commitment therapy: A review of meta-analyses. Journal of Contextual Behavioral Science. 2020;18:181-92.

23. Dindo L, Van Liew JR, Arch JJ. Acceptance and commitment therapy: a transdiagnostic behavioral intervention for mental health and medical conditions. Neurotherapeutics. 2017;14:546-53.

24. Mattila E, Lappalainen R, Välkkynen P, Sairanen E, Lappalainen P, Karhunen L, et al. Usage and dose response of a mobile acceptance and commitment therapy app: secondary analysis of the intervention arm of a randomized controlled trial. JMIR mHealth and uHealth. 2016;4(3):e5241.

25. Singh S, Starkey NJ, Sargisson RJ. Using SmartQuit®, an acceptance and commitment therapy smartphone application, to reduce smoking intake. Digital health. 2017;3:2055207617729535.

26. Craig P, Dieppe P, Macintyre S, Michie S, Nazareth I, Petticrew M. Developing and evaluating complex interventions: the new Medical Research Council guidance. Bmj. 2008;337.

27. Billingham SA, Whitehead AL, Julious SA. An audit of sample sizes for pilot and feasibility trials being undertaken in the United Kingdom registered in the United Kingdom Clinical Research Network database. BMC medical research methodology. 2013;13:1-6.

28. Eldridge SM, Chan CL, Campbell MJ, Bond CM, Hopewell S, Thabane L, et al. CONSORT 2010 statement: extension to randomised pilot and feasibility trials. bmj. 2016;355.

29. Henry JD, Crawford JR. The short-form version of the Depression Anxiety Stress Scales (DASS-21): Construct validity and normative data in a large non-clinical sample. British journal of clinical psychology. 2005;44(2):227-39.

For peer review only - http://bmjopen.bmj.com/site/about/guidelines.xhtml

30. Bond FW, Hayes SC, Baer RA, Carpenter KM, Guenole N, Orcutt HK, et al. Preliminary psychometric properties of the Acceptance and Action Questionnaire–II: A revised measure of psychological inflexibility and experiential avoidance. Behavior therapy. 2011;42(4):676-88.

31. Russell DW. UCLA Loneliness Scale (Version 3): Reliability, validity, and factor structure. Journal of personality assessment. 1996;66(1):20-40.

32. Kok BE, Coffey KA, Cohn MA, Catalino LI, Vacharkulksemsuk T, Algoe SB, et al. How positive emotions build physical health: Perceived positive social connections account for the upward spiral between positive emotions and vagal tone. Psychological science. 2013;24(7):1123-32.

33. Tennant R, Hiller L, Fishwick R, Platt S, Joseph S, Weich S, et al. The Warwick-Edinburgh mental well-being scale (WEMWBS): development and UK validation. Health and Quality of life Outcomes. 2007;5(1):1-13.

34. Balestroni G, Bertolotti G. EuroQol-5D (EQ-5D): an instrument for measuring quality of life. Monaldi Archives for Chest Disease. 2012;78(3).

35. McHugh L. A new approach in psychotherapy: ACT (acceptance and commitment therapy). The World Journal of Biological Psychiatry. 2011;12(sup1):76-9.

36. Clarke V, Braun V. Thematic analysis: a practical guide. Thematic Analysis. 2021:1-100.

37. Grissom RJ, Kim JJ. Effect sizes for research: Univariate and multivariate applications: Routledge; 2012.

[dataset] 38. Gordon TC, Kemp AH, Edwards DJ. Data from: Mixed-methods feasibility outcomes for a novel ACT-based videogame 'ACTing Minds' to support mental health. Open Science Framework, February 16, 2024. https://doi.org/10.17605/OSF.IO/3WUH5

For peer review only

39. Faul F, Erdfelder E, Lang A-G, Buchner A. G* Power 3: A flexible statistical power analysis program for the social, behavioral, and biomedical sciences. Behavior research methods. 2007;39(2):175-91.

40. Cohen J. Statistical power analysis for the behavioral sciences: Academic press; 2013.

41. McCrudden MT, Marchand G, Schutz PA. Joint displays for mixed methods research in psychology. Methods in Psychology. 2021;5:100067.

42. David OA, Cardoș RA, Matu S. Is RET hink therapeutic game effective in preventing emotional disorders in children and adolescents? Outcomes of a randomized clinical trial. European child & adolescent psychiatry. 2019;28:111-22.

43. Schuurmans AA, Nijhof KS, Engels RC, Granic I. Using a videogame intervention to reduce anxiety and externalizing problems among youths in residential care: an initial randomized controlled trial. Journal of psychopathology and behavioral assessment. 2018;40:344-54.

44. Garrido S, Millington C, Cheers D, Boydell K, Schubert E, Meade T, et al. What works and what doesn't work? A systematic review of digital mental health interventions for depression and anxiety in young people. Frontiers in psychiatry. 2019;10:759.

45. Grist R, Croker A, Denne M, Stallard P. Technology delivered interventions for depression and anxiety in children and adolescents: a systematic review and meta-analysis. Clinical Child and Family Psychology Review. 2019;22:147-71.

46. Huguet A, Rao S, McGrath PJ, Wozney L, Wheaton M, Conrod J, et al. A systematic review of cognitive behavioral therapy and behavioral activation apps for depression. PloS one. 2016;11(5):e0154248.

47. Venkatesan A, Rahimi L, Kaur M, Mosunic C. Digital cognitive behavior therapy intervention for depression and anxiety: retrospective study. JMIR Mental Health. 2020;7(8):e21304.

For peer review only

48. Wolgast M. What does the Acceptance and Action Questionnaire (AAQ-II) really measure? Behavior therapy. 2014;45(6):831-9.

49. Doorley JD, Goodman FR, Kelso KC, Kashdan TB. Psychological flexibility: What we know, what we do not know, and what we think we know. Social and Personality Psychology Compass. 2020;14(12):1-11.

50. Cherry KM, Vander Hoeven E, Patterson TS, Lumley MN. Defining and measuring "psychological flexibility": A narrative scoping review of diverse flexibility and rigidity constructs and perspectives. Clinical psychology review. 2021;84:101973.

For peer review only

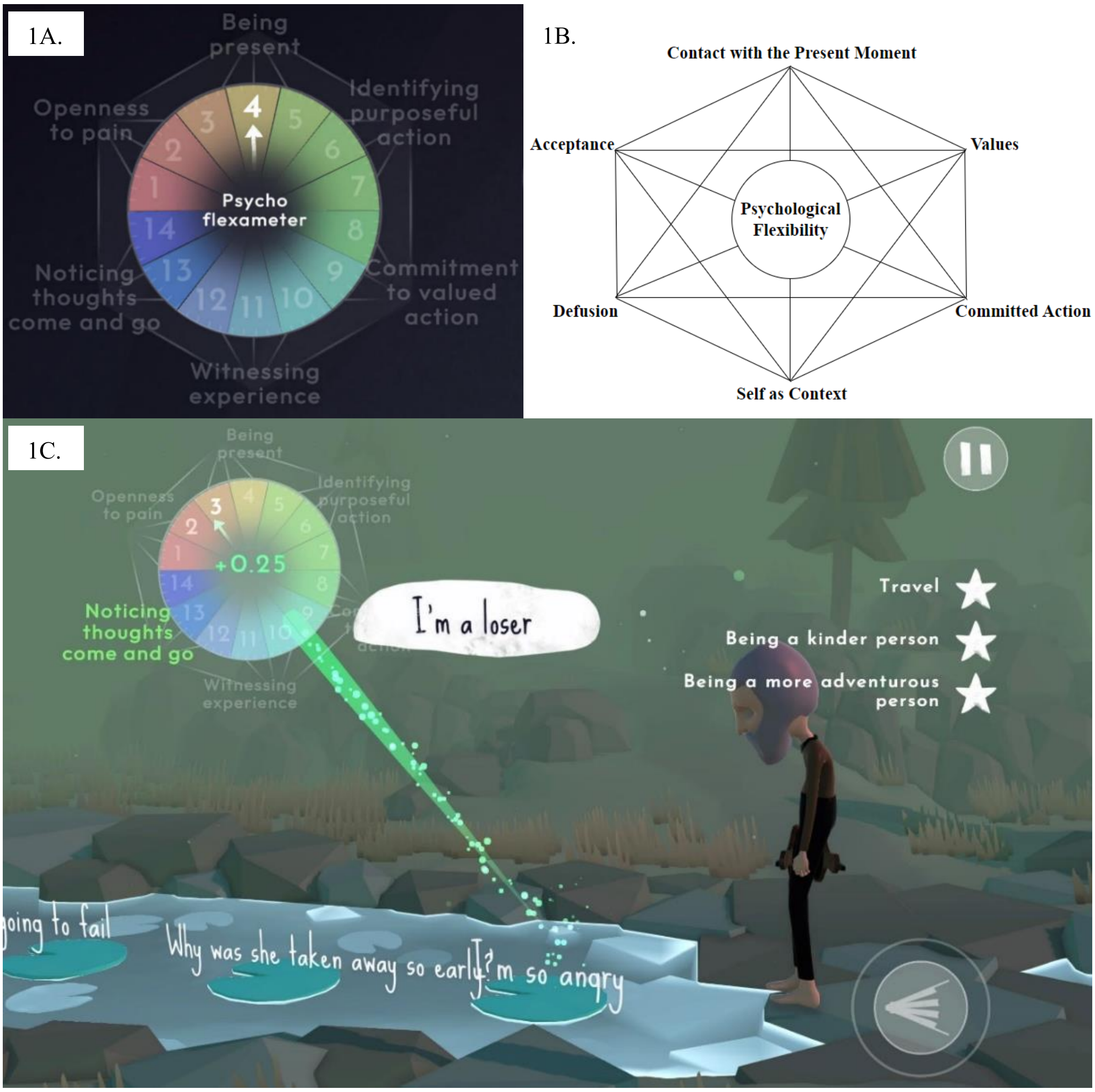
1A.
Being present
Identifying purposeful action
Openness to pain
Psycho flexameter
Noticing thoughts come and go
Commitment to valued action
Witnessing experience
1B.
Contact with the Present Moment
Acceptance
Values
Psychological Flexibility
Defusion
Committed Action
Self as Context
1C.
+0.25
I'm a loser
Travel
Being a kinder person
Being a more adventurous person
Why was she taken away so early
I'm so angry

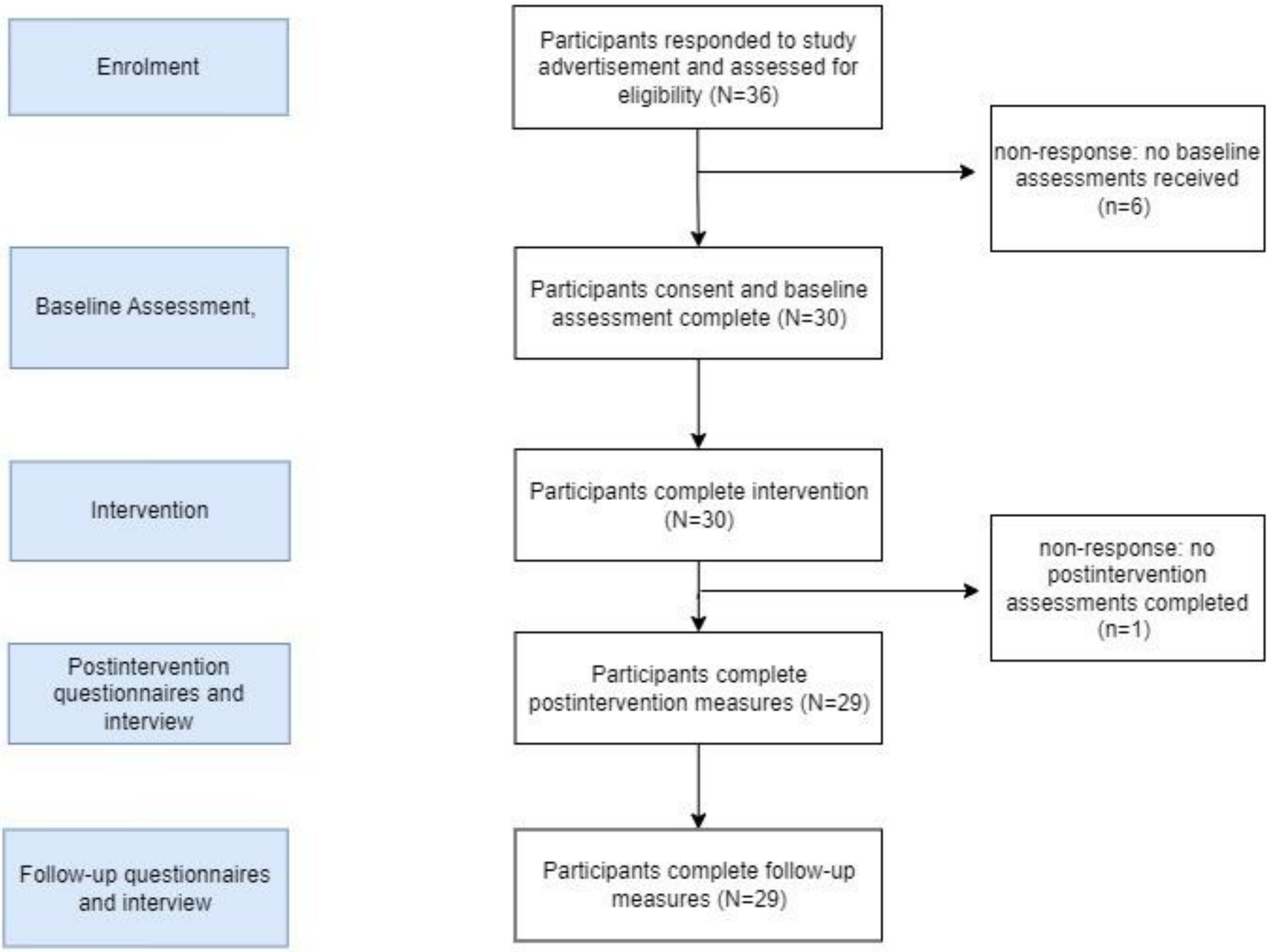
Enrolment
Participants responded to study advertisement and assessed for eligibility (N=36)
non-response: no baseline assessments received (n=6)
Baseline Assessment,
Participants consent and baseline assessment complete (N=30)
Intervention
Participants complete intervention (N=30)
non-response: no postintervention assessments completed (n=1)
Postintervention questionnaires and interview
Participants complete postintervention measures (N=29)
Follow-up questionnaires and interview
Participants complete follow-up measures (N=29)

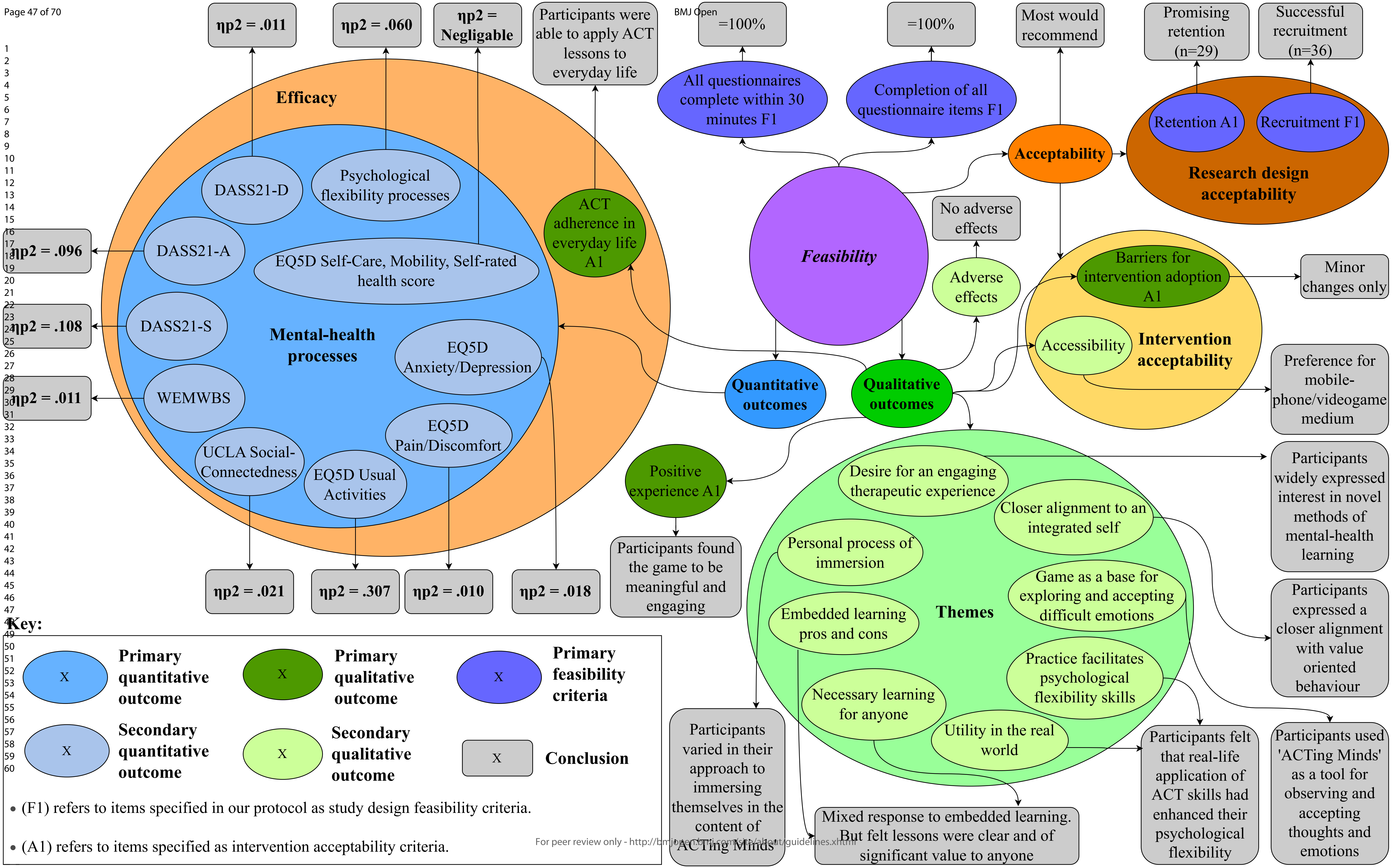

ηp2 = .011
ηp2 = .060
ηp2 = Negligable
Participants were able to apply ACT lessons to everyday life
=100%
=100%
Most would recommend
Promising retention (n=29)
Successful recruitment (n=36)
Efficacy
All questionnaires complete within 30 minutes F1
Completion of all questionnaire items F1
Retention A1
Recruitment F1
Acceptability
Research design acceptability
DASS21-D
Psychological flexibility processes
ACT adherence in everyday life A1
Feasibility
No adverse effects
ηp2 = .096
DASS21-A
EQ5D Self-Care, Mobility, Self-rated health score
Adverse effects
Barriers for intervention adoption A1
Minor changes only
ηp2 = .108
DASS21-S
Mental-health processes
EQ5D Anxiety/Depression
Accessibility
Intervention acceptability
Preference for mobile-phone/videogame medium
ηp2 = .011
WEMWBS
Quantitative outcomes
Qualitative outcomes
EQ5D Pain/Discomfort
UCLA Social-Connectedness
EQ5D Usual Activities
Positive experience A1
Desire for an engaging therapeutic experience
Participants widely expressed interest in novel methods of mental-health learning
Closer alignment to an integrated self
Participants found the game to be meaningful and engaging
Personal process of immersion
ηp2 = .021
ηp2 = .307
ηp2 = .010
ηp2 = .018
Game as a base for exploring and accepting difficult emotions
Participants expressed a closer alignment with value oriented behaviour
Embedded learning pros and cons
Themes
Key:
Primary quantitative outcome
Primary qualitative outcome
Primary feasibility criteria
Practice facilitates psychological flexibility skills
Necessary learning for anyone
Secondary quantitative outcome
Secondary qualitative outcome
Conclusion
Utility in the real world
Participants varied in their approach to immersing themselves in the content of ACTing Minds
Participants felt that real-life application of ACT skills had enhanced their psychological flexibility
Participants used 'ACTing Minds' as a tool for observing and accepting thoughts and emotions
• (F1) refers to items specified in our protocol as study design feasibility criteria.
• (A1) refers to items specified as intervention acceptability criteria.
Mixed response to embedded learning. But felt lessons were clear and of significant value to anyone

Open access
Protocol

BMJ Open

A novel ACT-based video game to support mental health through embedded learning: a mixed-methods feasibility study protocol

Darren J Edwards ,[1] Andrew H Kemp[2]

To cite: Edwards DJ, Kemp AH. A novel ACT-based video game to support mental health through embedded learning: a mixed-methods feasibility study protocol. *BMJ Open* 2020;**10**:e041667. doi:10.1136/bmjopen-2020-041667

► Prepublication history and additional material for this paper is available online. To view these files, please visit the journal online (http://dx.doi.org/10.1136/bmjopen-2020-041667).

Received 15 June 2020
Revised 06 October 2020
Accepted 30 October 2020

Check for updates

© Author(s) (or their employer(s)) 2020. Re-use permitted under CC BY-NC. No commercial re-use. See rights and permissions. Published by BMJ.

[1]Department of Public Health, Policy, and Social Sciences, Swansea University, Swansea, UK
[2]Department of Psychology, Swansea University, Swansea, UK

Correspondence to
Dr Darren J Edwards;
D.J.Edwards@swansea.ac.uk

ABSTRACT

Introduction In recent years, serious video games have been used to promote emotional regulation in individuals with mental health issues. Although these therapeutic strategies are innovative, they are limited with respect to scope of treatment, often focusing on specific cognitive skills, to help remediate a specific mental health disorder.
Objective Here, we propose a protocol for assessing the feasibility of a novel acceptance and commitment therapy (ACT)-based video game for young adults.
Methods and analysis The Medical Research Council (MRC) framework will be used for developing a complex intervention to design and test the feasibility of an ACT-based video game intervention using a mixed-methods approach involving qualitative and quantitative data. The primary outcomes will include feasibility testing of recruitment processes and the acceptability of the intervention through qualitative interviews, attendance and rates of attrition. Secondary outcomes will involve a series of quantitative questionnaires to obtain effect sizes for power analysis, allowing for the ideal sample size for an appropriately powered, randomised controlled trial to be determined.
Ethics and dissemination This study has been approved by the Psychology Department Research Ethics Committee (2020-4929-3923) at Swansea University in the UK. Dissemination activities will involve publications in peer-reviewed journals, presentations at local and national conferences and promotion through social media.
Trial registration number NCT04566042.

Strengths and limitations of this study

- Mixed-methods approach to build a rich dataset on which conclusions will be drawn.
- Protocol follows established Medical Research Council (MRC) guidelines.
- In line with MRC guidelines and stage of game development, randomisation is not a component of this study.
- Aims are to assess feasibility, an important step in the development of complex interventions, although limiting conclusions able to be drawn.

INTRODUCTION

Mental health issues such as anxiety and depression are a global problem of increasing concern, imposing considerable burden on society. The Global Burden of Disease project[1] has identified mental health disorders as a leading cause of disability globally, and suggest that there are 266 million cases of anxiety, and 253 million cases of major depressive disorder globally.

Unfortunately, the demand for mental health services far exceeds the available human resources able to meet this need in high-income and low-income andmiddle-income countries. This includes ever-growing treatment gaps[2] and lags.[3] These alarming increases have prompted the 2018 Lancet commission on global mental health to suggest that universal health coverage should include efforts to ensure the sustainable development of mental health.[4] Innovations to promote accessibility to mental health treatments include technology such as telephone, internet and smartphone devices, augmenting the psychotherapeutic toolkit.[5]

Innovations in video gaming for remediating mental health issues have wide potential application. In the USA, over 164 million adults play video games, and at least three-quarters of all American families have at least one person who video games regularly.[6] In the EU, 54% of the population play video games between the ages of 6 and 64, where the average age of video gamers is 31, and with a distribution of 46% female and 54% male. Of these, 77% play at least 1 hour per week, 16% play 1 hour per month, while only 7% play 1 hour per year.[7] Given that such a large proportion of the Western population play video games, developing mental health training in the form of psychoeducation may have great potential for building psychological resilience and

For peer review only - http://bmjopen.bmj.com/site/about/guidelines.xhtml

BMJ Open: first published as 10.1136/bmjopen-2020-041667 on 16 November 2020. Downloaded from http://bmjopen.bmj.com/ on October 15, 2023 by guest. Protected by copyright.

helping to better manage depression, anxiety and other forms of distress.

Technological developments for tackling such challenges include the exploitation of gamification.[8] This involves the application of behavioural principles for controlling and modifying human behaviour, in which game design elements are used to increase human interaction with or without technology.[9] Some examples of gamification include gamifying the development of cognitive skills and emotional regulation by rewarding the completion of relevant tasks within complex video games[10–13] in order to promote mental health.

When mental health-related video games are designed well, they have been shown to elevate self-esteem, self-efficacy, knowledge and awareness of illness, adherence to treatment and problem-solving skills, while lowering aggression.[14] One of the most successful in the facilitation of mental health improvement is a serious video game—a complex game with multiple levels and settings—called PlayMancer (PM), which targets emotional regulation and was specifically designed to help manage impulse control disorders.[11 13] The objective of the PM game is to develop emotional and cognitive skills, while reducing impulsivity. The game has been shown to help treat bulimia nervosa by improving emotional regulation.[15 16]

PM also uses biofeedback (heart rate and heart rate variability) to model physiological and emotional reactions, feeding this information back to the participant. Some research has shown that facilitating awareness of one's own physiology (such as brain activity or cardiac function) enhances the treatment effects of mental health disorders (such as anxiety disorder, depression, obsessive-compulsive disorder (OCD) and schizophrenia) via self-regulation.[17] Biofeedback has also been shown to improve impulse control difficulties, and attentional difficulties in bulimia nervosa and attention deficit hyperactivity disorder,[15 16 18] as well as symptoms of stress, anxiety and anger.[19] The focus on physiological data in the psychotherapeutic context is gaining traction[20–22] and has strong theoretical underpinnings.[23–25]

Within PM, there are three mini-games: 'The face of Cronos'; 'Treasures of the sea' and 'Sign of the Magupta'. Each of these mini-games were designed to train different skills, for example, 'The face of Cronos' and 'Treasures of the sea' develops planning skills, impulse control, coping skills, stress management and emotional self-regulation, while 'Sign of the Magupta' was designed to train relaxation, breathing techniques and improve physiological and emotional awareness. However, in the study[15] PM was combined with sessions of cognitive behavioural therapy (CBT) and without a control measure (eg, CBT only) so the game was developed as an adjunct to traditional mental health training, and there is no real way of knowing the direct benefits of the game as opposed to training in CBT. In another study—a case study of a single participant playing PM—anxiety and impulsivity decreased prior to CBT.[16] However, as this study was based on a single case, further studies using a randomised controlled trial (RCT) approach are needed to support and provide confidence to these findings.

Another game, Dojo,[26] develops emotional regulation in adolescents with anxiety. It uses biofeedback (heart rate variability) and trains breathing techniques, muscle relaxation, positive thinking and guided imagery to attempt to reduce anxiety in adolescence. It also uses instructional videos and then engages players through immersive and emotionally evocative puzzles that challenge players to use newly acquired emotion regulation skills. However, a pre-post RCT with 1347 participants, compared with a standard 'off the shelf' commercial game 'Rayman 2' (whereby Rayman 2 was the control), reported no difference between Dojo and the control condition at reducing anxiety. As both of these games significantly reduced anxiety, it is possible that the reduction in anxiety was due distraction from anxiety-provoking thoughts, rather than developing psychoeducational skills per se. The authors concluded that crucial design issues need to be carefully thought through, which include a clear theoretical and therapeutic foundation. This includes appropriate methodology that can assess the causes of improvement, before developing and testing a serious video game for the treatment of mental health issues such as anxiety.

Commercial games (such as Rayman 2) have been explored in their unmodified forms for their effectiveness in helping with social skills training for autism, and cognitive distraction for anxiety and nausea for patients undergoing chemotherapy,[27] with limited success. Evidence of generalisability of these games beyond game-playing is limited,[28] and this may be because they act as simple distractions rather than therapeutic psychoeducation applicable to participant's everyday lives. Another issue with many of these studies is that they often lack appropriate and rigorous methodology such as longitudinal follow-up,[29] and a mixed-methodological approach that can assess the feasibility and acceptability of such interventions.

Given these issues, it is important to emphasise that the underlying theoretical basis for PM and Dojo relates to the development of emotional regulation skills. While emotional regulation has transdiagnostic application[30] (ie, an intervention designed to treat multiple mental health conditions), these applications are not underpinned by theoretical frameworks that relate to formal psychotherapeutic interventions. Our proposed game is designed to be a comprehensive transdiagnostic intervention that will integrate a third wave behavioural therapy—as opposed to an adjunct to—acceptance and commitment therapy (ACT).[31 32] It will therefore be a comprehensive strategy for managing many common mental health issues such as depression and anxiety and focus on developing clear psychoeducational skills in the form of psychological flexibility, well-being and resilience more generally.[33]

Given this comprehensive transdiagnostic focus on psychological flexibility through ACT—a fundamental component of general health and well-being[34]—our online video game may have much greater reach and

For peer review only - http://bmjopen.bmj.com/site/about/guidelines.xhtml
BMJ Open: first published as 10.1136/bmjopen-2020-041667 on 16 November 2020. Downloaded from http://bmjopen.bmj.com/ on October 15, 2023 by guest. Protected by copyright.

impact than other serious video games such as PM, Dojo and many of the commercial games which are not based on third wave psychotherapy. Greater accessibility and impact have important implications for reducing treatment gaps and lags by making more mental health services available to those who need them.

One reason for choosing ACT in the game development process was pragmatism. For instance, researchers and clinicians may access freely available materials through the Association of Contextual Behavioural Science website (https://contextualscience.org/), and it does not require formal clinical training or accreditation to practice[35] which has important implications for translation to video game platforms. Another reason for choosing ACT as the basis for the game, is that it has a strong evidence base, and meta-analysis has found it to be efficacious for improving chronic pain, depression, psychotic symptoms, mixed anxiety, OCD, drug abuse and stress at work.[36] This means it is an ideal general purpose therapeutic tool as opposed to restricted focus on for example impulsivity control such as the PM application[11 13] or simple relaxation skills for adolescence with anxiety, as is the focus of the Dojo game.[26]

ACT principles are designed to undermine the trappings of language in the form of difficult thoughts and associated feelings, and promotion of psychological flexibility.[37] Language trappings can get individuals entangled and can prevent them from engaging with what is truly meaningful to them. The development of psychological flexibility through ACT is important because it is considered to be a fundamental component of well-being.[34]

The six ACT processes are: (1) the act of being in the here and now, present and mindful[32 38]; (2) acceptance, the act of being aware and open to painful thoughts; (3) cognitive fusion, the act of recognising that thoughts are just thoughts and not to buy into them (the process of cognitive defusion)[39]; (4) identifying values, values act as a life compass and direct us towards a life filled with purpose; (5) commitment to values orientation, which is the act of continually working towards a values orientation, even when an individual goes off track; (6) self as context (also called the transcendental self), is flexible and transcendent form of self. This involves the awareness of thoughts and feelings but the complete detachment from the literal meaning of thoughts.[34]

ACT has been usefully applied to many forms of mental health issues and has been applied in many different forms of delivery. This includes web-based interventions,[40–42] teleconference[43] and a downloadable app for smartphones.[44 45] So, given the fact that video games can have positive well-being benefits,[46 47] and are applicable for therapeutic purposes,[28 48] a transdiagnostic ACT serious video-game may have great potential for similar reasons.

As ACT is a comprehensive transdiagnostic model and formal third wave cognitive behavioural approach, then its reach and impact in the form of a video game may be greater than that of PM or Dojo which were focused on simpler emotional skills development and biofeedback. For these reasons, we are proposing an ACT-based video game called 'ACTing Mind' as an innovative and accessible intervention to help individuals who struggle with anxiety, depression, stress and other forms of distress.

Aims

The research goals of this proposal are to determine the feasibility and acceptability of a novel ACT-based video game intervention for individuals with mental distress, in line with methodology described in the Medical Research Council (MRC) framework.[49 50] This proposal lays the foundation for which a pilot and full-scale RCT will be conducted to determine clinical effectiveness, and ultimately the recommendations of the importance of such innovations in primary care mental health policies and practices.

METHODOLOGY

This protocol has been developed following the Template for Intervention Description and Replication of Studies[51] (see online supplemental appendix 1), as well as the MRC guidelines for the development of complex interventions.[49 50] This includes five stages of development for a complex intervention including: (1) preclinical, involving a theoretical review of the literature (provided here), justifying the need for such an intervention for the proposed population; (2) phase I, modelling, involving the use of evidence to determine the components for underlying mechanisms. For this, we propose a qualitative element involving thematic analysis to enable us to understand what would be most beneficial to a general population with anxiety and depression; (3) phase II, conducting an exploratory pilot study (outlined here) to determine the feasibility of the methodology and design where some initial data can be collected; (4) phase III, an RCT to test the efficacy of the proposed intervention (in subsequent work); (5) phase IV, long-term follow-up to assess replicability.

Public and patient involvement

Key stakeholders were consulted and involved in the development of this protocol. The Patient Experience and Evaluation in Research (Patient Experience and Evaluation in Research (PEER): https://www.swansea.ac.uk/humanandhealthsciences/research-at-the-college-of-human-and-health/patientexperienceandevaluationinresearchpeergroup/) group in the College of Human and Health Sciences at Swansea University were consulted. This group represented members of the public, students and staff members, several of whom reported that they had experienced depression, anxiety or stress at some point in their lives and emphasised the need for innovative approaches of the delivery of mental health support. The feasibility design was explained to them, and they gave positive feedback about the nature of the design, intervention and outcome measures.

For peer review only

For peer review only - http://bmjopen.bmj.com/site/about/guidelines.xhtml
BMJ Open: first published as 10.1136/bmjopen-2020-041667 on 16 November 2020. Downloaded from http://bmjopen.bmj.com/ on October 15, 2023 by guest. Protected by copyright.

Study design

This is a mixed-methods study which is designed to determine the feasibility and acceptability of an ACT-based video game for individuals with anxiety, depression and stress, and to increase psychological flexibility.

Study setting

The study will be conducted entirely online including the game and assessment (via the Qualtrics platform), and qualitative interviews (via the Zoom platform). Thus, potential participants will be able to access this study without restrictions, an important consideration for ongoing local lockdowns associated with the COVID-19 pandemic. Strict recommendations will require participants to ensure they are in a quiet room and without disruption for the duration of the study.

Recruitment and consent

We will recruit participants (n=36) using purposive sampling, focusing on—unlike an opportunity sample—the types of participants needed for a full-scale RCT (ie, individuals with depression, anxiety and stress). The sample size is justified on the basis of past research reporting the median numbers of participants recruited for similar types of feasibility studies,[52] incorporating both quantitative and qualitative elements.

Eligibility criteria

Participants will be recruited through general public mental health forums, social media and student populations. Thirty-six participants will take part in the study and they will be aged 18 years or older, be experiencing ongoing depression, anxiety and stress, and be able to read, write and speak English.

Intervention

This ACT-based video game intervention called 'ACTing Mind', developed and designed solely by DE, will involve students and members of the public attending five 1 hour sessions of an ACT-based video game. Each session will involve a different chapter of the video game, and each chapter will explore a different key component of ACT, with there being six in total (see table 1 for the different chapters and sessions involved).

These various components and principles of ACT[31 32] will be taught within the different chapters of the game and through embedded learning. For example, the player will gain ACT skills while completing objectives within the game and without directly being taught these skills, but rewarded indirectly through points and progress awards. For instance, in one scene (see table 1) the character is confronted by painful memories, and the player has two choices: (1) to destroy the painful memories or (2) to accept these memories. If the player chooses to destroy the memories (avoidant-based strategies), the world becomes distorted and barriers form making the chapter impossible to complete. Alternatively, if the player chooses acceptance-based strategies they will be able to continue the game (hence in this scene they learn that acceptance is functionally better than avoidance).

The game will start with a depressed individual who has recently lost his wife in an accident, and is feeling depressed, isolated and lonely (see figure 1 as an example of this scene). Each chapter will reward ACT consistent behaviour with points on a 'psychoflexameter'. This is a dial on the border of the screen which indicates increased psychological flexibility as the player completes ACT-based tasks such as acceptance (chapter 1), being present (chapter 2), values and commitment (chapter 3), defusion (chapter 4) and self as context (chapter 5). ACT uses metaphors to help clients visualise the key processes of ACT. In the game, these metaphors are real representations, such as the 'sinking sand' game, 'dropping the rope' game, the 'chessboard game', the 'unwanted monster' game, the 'leaves on a stream' game (see table 1).

Within the game, the character will have to enter his own mind through a 'mind escape machine' (see figure 2 of this as an example of the character in his own mind). At the start of the game, it is explained through a brief historical story that he develops this machine to destroy and supress his unwanted painful thoughts and memories about his wife and loss. Once in his mind, he will learn that destroying or supressing thoughts creates barriers in his mind which prevents him from continuing the game. So, learning acceptance is crucial throughout this game and the character is rewarded for this through points and progress awards. Also, within the game, psychoeducation components explain thoughts as trappings of language which can often get people stuck in life, and prevent them from value consistent living, as well as the various emotional regulation strategies such as avoidance and acceptance.

As part of the study, in addition to playing the video game, participants will be asked to record events on a weekly basis, aspects of application of the ACT principles learnt in an everyday life in a journal. It is anticipated that greater adherence to the intervention in everyday life, and engagement with the journal will lead to greater success of the intervention (greater psychological flexibility).

Data collection and management

MSc students will have the opportunity to be involved in this study and will collect and process the data under supervision by project leads, DJE and AK. Questionnaires will be completed online through Qualtrics which will store raw data copies, and also be held on an encrypted university server. Names and other personally identifiable information will not be stored, and consent form information will not be associated with the raw or processed data, instead each participant will be given a unique identifier code. Similarity recorded interviewer transcripts will use identifier codes as opposed to personal information (eg, names). The project leads (DJE, AHK) will frequently audit all processes in data collection and processing to ensure that the procedures stated in this protocol are adhered to.

For peer review only

BMJ Open: first published as 10.1136/bmjopen-2020-041667 on 16 November 2020. Downloaded from http://bmjopen.bmj.com/ on October 15, 2023 by guest. Protected by copyright.

Table 1 Overview of the 'ACTing Mind' intervention and everyday journal instructions

Session 1 (week 1): acceptance and openness to pain	► Chapter 1—Acceptance<br>► Introducing participants to the video game and ACT in everyday journal.<br>► A brief overview of the purpose of the programme and the content of each session.<br>► Explaining basic ACT tenets through introduction text of journal.<br>► Explaining the nature of painful thoughts and memories and getting caught up in the struggle explained through journal.<br>► Basic story context about the character being depressed and why, at start of video game.<br>► Explaining the objective of the video game, that is, to transcend form psychological inflexibility to psychological flexibility.<br>► Exercise, within the game there are choice, either to suppress, and break thoughts, or to accept and be open to them.<br>► Acceptance and openness are rewarded by psychological flexibility points on the 'psychoflexameter' and game progression, while suppression actions (breaking or suppressing painful memories) are punished with physical barriers, and sinking sand, which prevent the player from progressing in the game.<br>► A monster pulls against the player to prevent progress, but if the player fights with the monster, they get even more stuck (analogous to the drop the rope and sinking sand metaphor). Again, acceptance is important and must be learnt here.<br>► Reflecting in the journal about how this might be applied in life, and when this has occurred throughout the week daily.
Session 2 (week 1): being present (mindfulness)	► Chapter 2—Being present (mindfulness)<br>► Some instructions form the journal about being present and mindful is given, why it is useful and how to go about achieving with breathing exercises.<br>► The character is approached by monsters in the game in the past and future making him worry excessively about imaginary dangers, and reminding him of painful events.<br>► The game (in the form of the character's wife's ghost) instructs the player to be present, to focus on your breathing for 10 min.<br>► As the participant learns and completes relevant psychological flexibility tasks psychological flexibility on the 'psychoflexameter' will increase, which rewards the player for being present.<br>► Reflecting in the journal about how this might be applied in life, and when this has occurred throughout the week daily.
Session 3 (week 2): values identification and commitment	► Chapter 3—Values identification and commitment<br>► Instructions about what are values (a life compass) explained through the journal.<br>► Acceptance and commitment to values orientation as opposed to avoidance behaviour is rewarded.<br>► There are challenges to reach goals which are linked to the character's values, such as scary weather and monsters.<br>► Psychological flexibility on the 'psychoflexameter' and game progress, will increase with values consistent behaviour which rewards the player for committing to values.<br>► Reflecting in the journal about how this might be applied in life, and when this has occurred throughout the week daily.
Session 4 (week 2): defusion	► Chapter 4—Defusion<br>► Instructions about what is cognitive fusion and defusion (holding self-stories lightly) explained through journal.<br>► The character goes back into the 'Mind Escape' machine but this time there is a flowing river with leaves (analogous to leaves on a stream metaphor).<br>► Some of the character's painful memories will beg the player to help them, but if the player interacts, barriers and quicksand appear, punishing the player and preventing them from progressing in the game (analogous to the sinking sand metaphor).<br>► The ghost of the character's wife eventually instructs the player to put the memories and thoughts onto the leaves and watch them flow down the river, without interacting with them, and to simply observe.<br>► Psychological flexibility on the 'psychoflexameter', will increase when all of the memories and thoughts as left to go down the stream, hence the player is rewarded for defusing.<br>► Reflecting in the journal about how this might be applied in life, and when this has occurred throughout the week daily.

Continued

BMJ Open: first published as 10.1136/bmjopen-2020-041667 on 16 November 2020. Downloaded from http://bmjopen.bmj.com/ on October 15, 2023 by guest. Protected by copyright.

Table 1 Continued	
Session 5 (week 3): self as context	► Chapter 5—Self as context<br>► Instructions about what is self as context (being the observer of your thoughts and not your thought) are explained through journal.<br>► The world starts to fall apart and becomes abstract, like a chess board.<br>► The player realises that they are the white pieces on the chessboard (analogous to chess board metaphor).<br>► The player is compelled by the game to beat the black pieces in the chess game.<br>► But the more the players fights against the black pieces, the more they lose points on the 'psychoflexameter' and cannot progress in the game.<br>► The player must let the battle play out, once they do, they become aware that they are the chess board (they become it) and realise they do not need to be part of the never-ending battle between the opposing forces.<br>► Finally, a bus arrives, memories of the character's wife beg the player to stay, and the monsters pull on player.<br>► The player needs to get onto the bus with the monsters to move towards their values, a new beginning (analogues to bus metaphor).<br>► Finally, the player has a choice, go back and change the events that led to your wife's death, or stay on the bus with the monsters.<br>► Trying to change events leads to a loss in points and prevents game progression.<br>► Only staying on the bus, towards values, and accepting the monsters allows the player to complete the game successfully.<br>► Reflecting in the journal about how this might be applied in life, and when this has occurred throughout the week daily.

Outcome measures

Questionnaires will be collected at three points in time (baseline, immediate postintervention and 3-month follow-up). Interview data will be collected immediate postintervention only.

Demographic data

Demographic measures will include age, sex, medication use, which will all be recorded through Qualtrics and assessed by DJE and AHK.

Primary outcome measure

The primary outcomes for feasibility are determined using MRC framework measure for developing a complex intervention.[49 50] As this is a feasibility study, the primary outcomes (in accordance with the MRC framework) will include the acceptability of the ACT-based video game intervention, the feasibility of the recruitment, outcome measures and intervention adherence.

Acceptability

- Number of people dropping out.
- Barriers for adoption of intervention as assessed through interviews.
- Number of sessions attended.
- Time dedicated to home journal.
- ACT principles adherence in everyday life setting (as recorded in journal and expressed through interviews).
- Experience, identifying whether participants had positive experience with the intervention and whether they wanted to continue to be part of the intervention.

Feasibility

- Number of participants who are willing to take part.
- Time taken to complete questionnaires.
- Number of complete and incomplete questionnaires.

Secondary outcome measures

Warwick-Edinburgh Mental Well-Being Scale[53]: a measure of mental well-being with a focus on positive aspects of mental health. This measure has good internal

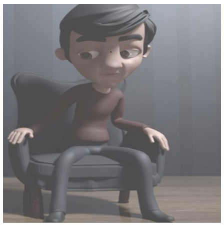

Figure 1 First scene in 'ACTing Mind', the character, Steve, is depressed and alone.

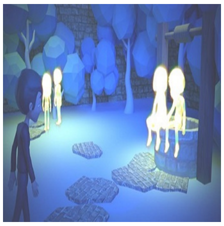

Figure 2 An example scene, where the character 'Steve' is in his own mind, and can see his own memories, through his Mindscape machine.

For peer review only - http://bmjopen.bmj.com/site/about/guidelines.xhtml

BMJ Open: first published as 10.1136/bmjopen-2020-041667 on 16 November 2020. Downloaded from http://bmjopen.bmj.com/ on October 15, 2023 by guest. Protected by copyright.

consistency with a Cronbach's alpha coefficient of 0.89 (student sample) and 0.91 (general population sample).

Depression Anxiety Stress Scales: a short version of this measure and a measure of general psychological distress with good construct validity (confirmatory factor analysis of 0.94). It has good internal reliability as measured through Cronbach's alpha coefficients, which are 0.88 for depression, 0.82 for anxiety, 0.90 for stress and 0.93 for the total scale.[54]

Social connectedness (adapted from Russell's (1996) UCLA Loneliness Scale[55]: this measure involves two questions: (1) "During social interactions, I feel 'in tune' with the person/s around me", and (2) "During social interactions, I feel close to the person/s". The Cronbach's alpha coefficients for these two items ranged from 0.80 to 0.98 (M=0.94, SD=0.03).[55]

EuroQol five dimensions (EQ5D): the EQ5D is a measure for health-related quality of life. There are five components within this measure which assess mobility, self-care, usual activities, pain, discomfort and anxiety. It also has a visual analogue scale (VAS) for measuring current health status. Scores for these will be calculated for each of these five subsections as well as including the VAS and total EQ5D score of all five subsections. The EQD5 correlates well with other health-related questionnaires such as the 36-Item Short Form Survey (r=0.61, p<0.0001) and Parkinson's Disease Questionnaire (PDQ)-39 (r=−0.75, p<0.0001).[56]

Acceptance and Action Questionnaire-second version: this is a seven-item scale developed by Bond *et al*[57] to measure psychological inflexibility, which involves the ability to accept and be open to difficult thoughts and feelings as well as to engage in valued behaviour in the presence of the difficult thoughts and feelings. A higher score indicates higher psychological inflexibility. The measure has good construct validity with a Cronbach's alpha coefficient of 0.84.[57]

Adherence to the intervention measure and trial

Adherence will be measured in a variety of ways such as intervention feedback, treatment adherence through attrition rates as well as meta-data of relating to game log-in and log-out, as well as how long the game was played for and what sessions of the game were completed for each participant. Similar information can be recorded in Qualtrics for ensuring questionnaires are completed carefully. This includes length of times completing the questionnaire, and paying attention to reverse-scored questions.

Sample size and statistical analysis

Sample size recruited will help us determine whether it is possible to recruit sufficient numbers of participants to manage a full-scale RCT at a later date.

Quantitative data analysis: analysis will focus on descriptive statistics and feasibility outcomes of the questionnaires. While clinical effectiveness will not be formally evaluated at this stage, effect sizes will be explored for early evidence that the intervention shows promising signs (including ACT-related process measures). It is predicted that outcomes will improve, and any improvement will be identified using a one-way analysis of covariance with a single within-subjects factor (time). The effect sizes will also allow for a power calculation to be made which will allow for an approximation for a sample size required in a future trial (if indicated).

Qualitative data analysis: transcripts of focus group interview data will be generated from digital audio-recordings of in-depth, face-to-face semi-structured interviews (all online and via a password-protected room in Zoom). In-depth semi-structured interviews will form the core topics to be discussed (see table 2), while leaving space and scope for the identification and exploration of unforeseen information that may emerge. Insights from this will allow for further development and improvement of the intervention, along with the quantitative data in line with the MRC guidelines.[49 50]

Thematic analysis will then be conducted which will explore key overarching themes that may emerge from the focus group interviews following standardised guidelines.[58] The interview questions are based on other novel ACT-based protocols.[40 59] The data will be analysed after the study has been completed. We will follow the inductive and deductive code development as outlined by Fereday and Muir-Cochrane[60] to ensure necessary rigour. Any key overarching themes identified which relate to feasibility of the study design of the acceptability of the intervention, as well as potential adverse effects, will be explored and reported.

The focus groups will comprise 4 to 6 groups with 6 to 10 individuals in each group as has been suggested as optimal in other studies.[61] The interview will take place at the end of the intervention (week 3). It will explore various aspects of the intervention such as perceived process of change, barriers to intervention adherence, trial process and any adverse effects, which help supplement the quantitative approach. Process of change questions indicate whether the participant learnt anything about ACT, and felt any positive change in their life due to participating in the intervention. The question relating to barriers explores any problems and difficulties they had with the intervention. Another question will be asked to elicit suggestions for improvement relating to game or study design. Acceptability questions and process of change in one's life relate to whether the participant accepted the intervention and used skills they learnt through the intervention in daily life. The question relating to the trial process will determine whether there were any difficulties or limitations of the trial itself such as whether the instructions were clear and how it could be improved. Finally, the question on adverse effects explores whether there were any potential unforeseen negative consequences of the intervention.

Limitations of the study

This study protocol has limitations. First, while physiological measures would ideally be collected to measure

For peer review only

For peer review only - http://bmjopen.bmj.com/site/about/guidelines.xhtml

BMJ Open: first published as 10.1136/bmjopen-2020-041667 on 16 November 2020. Downloaded from http://bmjopen.bmj.com/ on October 15, 2023 by guest. Protected by copyright.

Table 2 Qualitative interview protocol for the focus groups

Acceptability and feasibility	How would you describe your experience of taking part in 'ACTing Mind' video game programme?
Accessibility of intervention	If this intervention were rolled out as a video game app, do you think you would download it? Would you appreciate the accessibility?
Process of change	What did you learn from this programme?
Acceptability	What was the aspect of the programme that you liked the most? What was your favourite activity within the game (or applied to your everyday life)?
Suggestions for further improvement	What did you least like about the intervention? What do you think could be improved?
Barriers	Were there any difficulties to taking part?
Implementing change in everday life	Do you practice mindfulness, acceptance, defusion and values? How often? Could you apply what you have learnt through video game intervention to the real world in everyday events? Will you apply this new knowledge to everyday events?
Process of change	Have you noticed any differences in your life as a result of taking part in 'ACTing Mind'? If 'yes', what are these differences?
Acceptability	Would you recommend this intervention to someone you care about? Did you like the theoretical concepts central to the ACT intervention? How did you feel about its delivery? Was any of it too abstract or difficult to understand?
Processes of the trial	Was there anything you liked, or disliked about the study? How could we improve this study? Were all the instructions clear?
Adverse effects	Did you feel that any aspect of the intervention may have made worse any aspect of your anxiety, depression or stress? Were there any adverse effects that you can recognise due to the intervention?

variables such as heart rate variability, the COVID-19 pandemic limits our capacity to do this. However, the present study will provide important data on which such measures could be collected, analysed and interpreted in a future trial. Second, it could be questioned why there is no control condition in this study. Our response to this potential criticism is that the aim of the present study is to assess feasibility and—in line with the MRC guidelines[49 50]—has not been designed to be a full-scale RCT given the current research phase. Once the feasibility component is completed, a control condition will be introduced, which allow for the intervention condition to be compared with control, and as part of a full trial. Finally, although we would like to have ability to monitor the participant more directly, to ensure adherence to the intervention, we are sensitive to privacy issues associated with, for example, capturing participants' identity from the computers video camera. To mitigate this limitation, we have opted for less invasive procedures for measuring intervention adherence that will include logging metadata of the game such as log in and out times, as well as completion of game sections. Several questions in the questionnaire are also reverse scored to ensure participants are paying attention.

Protocol amendments

If the protocol is amended in any way, it will be communicated to relevant parties immediately, such as to participants, journal and ethics committee.

Ethics and dissemination

This study has received ethical approval from Swansea University Psychology Department ethics committee (2020-4920-3923). Participants will be informed of their rights to confidentiality and to leave the study at any time and without penalty. Both qualitative and quantitative data will be held on a password-protected computer accessible only to researchers DJE and AHK. The data will be anonymised with a unique identifier code, and any personally identifiable information will be removed.

Dissemination will involve peer-reviewed journals; leading national and international conferences, social media and public events and through general public health engagement such as talks at schools, the Welsh Government and engagement with annual science festivals including 'a pint of science'.

Impact of intervention

The potential impact of this study is far reaching as it will add to the growing set of online resources which support psychological resilience, flexibility and well-being. These resources are designed to be easy to access and are ideal for situations where travel is limited due to physical (disability) or situational (coronavirus) immobility. Such interventions can help alleviate widely reported mental health treatment gaps[2] and lags,[3] associated with the widely reported scarce human resources needed to provide mental health support for the many individuals who need it. The 2018 Lancet commission on global mental health argued that sustainable development

For peer review only - http://bmjopen.bmj.com/site/about/guidelines.xhtml

BMJ Open: first published as 10.1136/bmjopen-2020-041667 on 16 November 2020. Downloaded from http://bmjopen.bmj.com/ on October 15, 2023 by guest. Protected by copyright.

of mental health should be an essential component of universal health coverage.[4] Technological innovation of mental health support services, in the form of video games, may be one means to achieve this sustainability and a reduction in the treatment gap and lag.

Ancillary and poststudy care

Postintervention care has not been anticipated given this is a low-level (low-risk) intervention. Of course, all participants will be given a debrief form which will signpost individuals to the relevant free well-being services such as the Samaritans.

Acknowledgements The authors would like to thank Professor Louise McHugh (University College Dublin), who provided some very helpful feedback about the gamification of an ACT-based approach through the utilisation of behavioural principles.

Contributors DJE developed the intervention. DJE and AHK agreed on a set of outcomes. DJE wrote the first draft of the protocol and DJE and AHK then revised the subsequent drafts of the protocol. Both authors helped to revise the manuscript for intellectual content and agreed on the final version prior to submission for peer review.

Funding The authors have not declared a specific grant for this research from any funding agency in the public, commercial or not-for-profit sectors.

Competing interests At the time of writing this, DJE is discussing with Agor IP at Swansea University the potential to commercialise the described video game as a mobile application; however, at this time no agreements have been made or signed. AHK has no competing interests.

Patient consent for publication Not required.

Provenance and peer review Not commissioned; externally peer reviewed.

Supplemental material This content has been supplied by the author(s). It has not been vetted by BMJ Publishing Group Limited (BMJ) and may not have been peer-reviewed. Any opinions or recommendations discussed are solely those of the author(s) and are not endorsed by BMJ. BMJ disclaims all liability and responsibility arising from any reliance placed on the content. Where the content includes any translated material, BMJ does not warrant the accuracy and reliability of the translations (including but not limited to local regulations, clinical guidelines, terminology, drug names and drug dosages), and is not responsible for any error and/or omissions arising from translation and adaptation or otherwise.

Open access This is an open access article distributed in accordance with the Creative Commons Attribution Non Commercial (CC BY-NC 4.0) license, which permits others to distribute, remix, adapt, build upon this work non-commercially, and license their derivative works on different terms, provided the original work is properly cited, appropriate credit is given, any changes made indicated, and the use is non-commercial. See: http://creativecommons.org/licenses/by-nc/4.0/.

ORCID iD

Darren J Edwards http://orcid.org/0000-0002-2143-1198

REFERENCES

1 Whiteford H, Ferrari A, Degenhardt L. Global burden of disease studies: implications for mental and substance use disorders. *Health Aff* 2016;35:1114–20.
2 Patel V, Maj M, Flisher AJ, *et al*. Reducing the treatment gap for mental disorders: a WPA survey. *World Psychiatry* 2010;9:169–76.
3 Wang PS, Berglund PA, Olfson M, *et al*. Delays in initial treatment contact after first onset of a mental disorder. *Health Serv Res* 2004;39:393–416.
4 Patel V, Saxena S, Lund C, *et al*. The Lancet commission on global mental health and sustainable development. *The Lancet* 2018;392:1553–98.
5 Kazdin AE, Blase SL. Rebooting psychotherapy research and practice to reduce the burden of mental illness. *Perspect Psychol Sci* 2011;6:21–37.
6 ESA. 2019 essential facts about the computer and video game industry, 2020. Available: https://www.theesa.com/esa-research/2019-essential-facts-about-the-computer-and-video-game-industry/
7 ISFE. ISFE key facts 2019, 2020. Available: https://www.isfe.eu/isfe-key-facts/
8 Linehan C, Kirman B, Roche B. *Gamification as behavioral psychology. In the gameful world: approaches, issues, applications*. Cambridge, Massachusetts: MIT Press, 2015.
9 Deterding S, Dixon D, Khaled R, *et al*. From game design elements to gamefulness: defining "gamification". *Proceedings of the 15th international academic MindTrek conference: Envisioning future media environments*, 2011:9–15.
10 Jon Hobbs L, Yan Z. Cracking the walnut: using a computer game to impact cognition, emotion, and behavior of highly aggressive fifth grade students. *Comput Human Behav* 2008;24:421–38.
11 Jiménez-Murcia S, Fernández-Aranda F, Kalapanidas E, *et al*. Playmancer project: a serious videogame as an additional therapy tool for eating and impulse control disorders. *Stud Health Technol Inform* 2009;144:163–6.
12 Ducharme P, Wharff E, Hutchinson E, *et al*. Videogame assisted emotional regulation training: an act with RAGE-Control case illustration. *Clin Soc Work J* 2012;40:75–84.
13 Fernández-Aranda F, Jiménez-Murcia S, Santamaría JJ, *et al*. Video games as a complementary therapy tool in mental disorders: PlayMancer, a European multicentre study. *J Ment Health* 2012;21:364–74.
14 Santamaria JJ, Antonio S, Fernando F-A, *et al*. Serious games as additional psychological support: a review of the literature. *J Cyber Ther Reh* 2011;4:469–76.
15 Fagundo AB, Santamaría JJ, Forcano L, *et al*. Video game therapy for emotional regulation and impulsivity control in a series of treated cases with Bulimia nervosa. *Eur Eat Disord Rev* 2013;21:493–9.
16 Giner-Bartolomé C, Fagundo AB, Sánchez I, *et al*. Can an intervention based on a serious videogame prior to cognitive behavioral therapy be helpful in Bulimia nervosa? a clinical case study. *Front Psychol* 2015;6:982.
17 Schoenberg PLA, David AS. Biofeedback for psychiatric disorders: a systematic review. *Appl Psychophysiol Biofeedback* 2014;39:109–35.
18 Howard R, Schellhorn K, Lumsden J. A biofeedback intervention to control impulsiveness in a severely personality disordered forensic patient. *Personal Ment Health* 2013;7:168–73.
19 Pawlow LA, O'Neil PM, Malcolm RJ. Night eating syndrome: effects of brief relaxation training on stress, mood, hunger, and eating patterns. *Int J Obes Relat Metab Disord* 2003;27:970–8.
20 Dana DA. *The Polyvagal theory in therapy: engaging the rhythm of regulation (Norton series on interpersonal neurobiology)*. New York: WW Norton & Company, 2018.
21 Lehrer PM. Heart rate variability biofeedback and other psychophysiological procedures as important elements in psychotherapy. *Int J Psychophysiol* 2018;131:89–95.
22 Tulip C, Fisher Z, Bankhead H, *et al*. Building wellbeing in people with chronic conditions: a qualitative evaluation of an 8-week positive psychotherapy intervention for people living with an acquired brain injury. *Front Psychol* 2020;11:66.
23 Kemp AH, Koenig J, Thayer JF. From psychological moments to mortality: a multidisciplinary synthesis on heart rate variability spanning the continuum of time. *Neurosci Biobehav Rev* 2017;83:547–67.
24 Mead J, Wilkie L, Gibbs K, *et al*. Rethinking wellbeing: toward a more ethical science of wellbeing that considers current and future generations, 2019. Available: https://www.authorea.com/doi/full/10.22541/au.156649190.08734276
25 Kemp A. *Fisher, social ties, health and wellbeing: a literature review and model, in neuroscience and social science*. Berlin: Springer, 2017: 397–427.
26 Scholten H, Malmberg M, Lobel A, *et al*. A randomized controlled trial to test the effectiveness of an immersive 3D video game for anxiety prevention among adolescents. *PLoS One* 2016;11:e0147763.
27 Colder Carras M, Van Rooij AJ, Spruijt-Metz D, *et al*. Commercial video games as therapy: a new research agenda to unlock the potential of a global pastime. *Front Psychiatry* 2017;8:300.
28 Griffiths MD, Kuss DJ, de Gortari ABO. Videogames as therapy: an updated selective review of the medical and psychological literature. *JPHIM* 2017;5:71–96.
29 Zayeni D, Raynaud J-P, Revet A. Therapeutic and preventive use of video games in child and adolescent psychiatry: a systematic review. *Front Psychiatry* 2020;11:36.
30 Sloan E, Hall K, Moulding R, *et al*. Emotion regulation as a transdiagnostic treatment construct across anxiety, depression, substance, eating and borderline personality disorders: a systematic review. *Clin Psychol Rev* 2017;57:141–63.
31 Hayes S. *Acceptance and commitment therapy*. Washington, DC: American Psychological Association, 2009.

BMJ Open: first published as 10.1136/bmjopen-2020-041667 on 16 November 2020. Downloaded from http://bmjopen.bmj.com/ on October 15, 2023 by guest. Protected by copyright.

For peer review only - http://bmjopen.bmj.com/site/about/guidelines.xhtml

32 Hayes Steven C, Kirk D. *Acceptance and commitment therapy: the process and practice of mindful change*. New York: Guilford Press, 2011.
33 Dindo L, Van Liew JR, Arch JJ. Acceptance and commitment therapy: a transdiagnostic behavioral intervention for mental health and medical conditions. *Neurotherapeutics* 2017;14:546–53.
34 Kashdan TB, Rottenberg J. Psychological flexibility as a fundamental aspect of health. *Clin Psychol Rev* 2010;30:865–78.
35 Harris R. *Act made simple: an easy-to-read primer on acceptance and commitment therapy*. Oakland, California: New Harbinger Publications, 2009.
36 Öst L-G. The efficacy of acceptance and commitment therapy: an updated systematic review and meta-analysis. *Behav Res Ther* 2014;61:105–21.
37 Hayes SC, Strosahl KD, Bunting K, *et al*. *What is acceptance and commitment therapy?, in a practical guide to acceptance and commitment therapy*. Berlin: Springer, 2004: 3–29.
38 Strosahl K, Wilson K. *Acceptance and commitment therapy: an experiential approach to behavior change*. New York: Guilford Press, 1999.
39 Hayes SC. *Get out of your mind and into your life: the new acceptance and commitment therapy*. Oakland, California: New Harbinger Publications, 2005.
40 Edwards DJ, Rainey E, Boukouvala V, *et al*. Novel ACT-based eHealth psychoeducational intervention for students with mental distress: a study protocol for a mixed-methodology pilot trial. *BMJ Open* 2019;9:e029411.
41 Viskovich S, Pakenham KI. Pilot evaluation of a web-based acceptance and commitment therapy program to promote mental health skills in university students. *J Clin Psychol* 2018;74:2047–69.
42 Levin ME, Haeger JA, Pierce BG, *et al*. Web-based acceptance and commitment therapy for mental health problems in college students: a randomized controlled trial. *Behav Modif* 2017;41:141–62.
43 Herbert MS, Afari N, Liu L, *et al*. Telehealth versus in-person acceptance and commitment therapy for chronic pain: a randomized noninferiority trial. *J Pain* 2017;18:200–11.
44 Bricker JB, Mull KE, Kientz JA, *et al*. Randomized, controlled pilot trial of a smartphone APP for smoking cessation using acceptance and commitment therapy. *Drug Alcohol Depend* 2014;143: :87–94.
45 Levin ME, Haeger J, Pierce B, *et al*. Evaluating an adjunctive mobile APP to enhance psychological flexibility in acceptance and commitment therapy. *Behav Modif* 2017;41:846–67.
46 Johnson D, Jones C, Scholes L, *et al*. Videogames and wellbeing: a comprehensive review. young and well cooperative research centre, Melbourne, 2013. Available: https://www.youngandwellcrc.org.au/wp-content/uploads/2014/03/Videogames_and_Wellbeing.pdf
47 Vella K, Johnson D, Hides L. Positively playful: when videogames lead to player wellbeing. *Proceedings of the First International Conference on Gameful Design, Research, and Applications*, 2013:99–102.
48 Villani D, Carissoli C, Triberti S, *et al*. Videogames for emotion regulation: a systematic review. *Games Health J* 2018;7:85–99.
49 Craig P. Developing and evaluating complex interventions: following considerable development in the field since 2006, MRC and NIHR have jointly commissioned an update of this guidance to be published in 2019, 2019. Available: https://mrc.ukri.org/documents/pdf/complex-interventions-guidance/
50 Craig P, Dieppe P, Macintyre S, *et al*. Developing and evaluating complex interventions: the new medical Research Council guidance. *BMJ* 2008;337:a1655.
51 Hoffmann TC, Glasziou PP, Boutron I, *et al*. Better reporting of interventions: template for intervention description and replication (TIDieR) checklist and guide. *BMJ* 2014;348:g1687.
52 Billingham SAM, Whitehead AL, Julious SA. An audit of sample sizes for pilot and feasibility trials being undertaken in the United Kingdom registered in the United Kingdom clinical research network database. *BMC Med Res Methodol* 2013;13:104.
53 Tennant R, Hiller L, Fishwick R, *et al*. The Warwick-Edinburgh mental well-being scale (WEMWBS): development and UK validation. *Health Qual Life Outcomes* 2007;5:63.
54 Henry JD, Crawford JR. The short-form version of the depression anxiety stress scales (DASS-21): construct validity and normative data in a large non-clinical sample. *Br J Clin Psychol* 2005;44:227–39.
55 Kok BE, Coffey KA, Cohn MA, *et al*. How positive emotions build physical health: perceived positive social connections account for the upward spiral between positive emotions and vagal tone. *Psychol Sci* 2013;24:1123–32.
56 Schrag A, Selai C, Jahanshahi M, *et al*. The EQ-5D--a generic quality of life measure-is a useful instrument to measure quality of life in patients with Parkinson's disease. *J Neurol Neurosurg Psychiatry* 2000;69:67–73.
57 Bond FW, Hayes SC, Baer RA, *et al*. Preliminary psychometric properties of the acceptance and action Questionnaire-II: a revised measure of psychological inflexibility and experiential avoidance. *Behav Ther* 2011;42:676–88.
58 Braun V, Clarke V. Using thematic analysis in psychology. *Qual Res Psychol* 2006;3:77–101.
59 Saracutu M, Edwards DJ, Davies H, *et al*. Protocol for a feasibility and acceptability study using a brief ACT-based intervention for people from Southwest Wales who live with persistent pain. *BMJ Open* 2018;8:e021866.
60 Fereday J, Muir-Cochrane E. Demonstrating rigor using thematic analysis: a hybrid approach of inductive and deductive coding and theme development. *Int J Qual Methods* 2006;5:80–92.
61 Morgan DL. Focus groups. *Annu Rev Sociol* 1996;22:129–52.

For peer review only

For peer review only - http://bmjopen.bmj.com/site/about/guidelines.xhtml

BMJ Open: first published as 10.1136/bmjopen-2020-041667 on 16 November 2020. Downloaded from http://bmjopen.bmj.com/ on October 15, 2023 by guest. Protected by copyright.

Swansea University
Prifysgol Abertawe

PARTICIPANT INFORMATION SHEET

A novel ACT-based video game to support mental health through embedded learning

You are being invited to take part in some research. Before you decide whether or not to participate, it is important for you to understand why the research is being conducted and what it will involve. Please read the following information carefully.

What is the purpose of the research?

The aim of the research project is to gain insight into whether exposure to a novel-acceptance and commitment therapy (ACT) video game can improve various outcome measures of depression, anxiety, stress, and other measures relating to wellbeing.

If you are above the age of 18 and are currently experiencing ongoing mild to moderate levels of depression, anxiety, or stress you are welcome to participate in this study. For those individuals who present with high levels of depression and anxiety, we will advise the participant to consult either with a GP or with a therapist if they have one, in relation to participating in this experiment.

Participation in this study will take approximately 3 hours over three weeks. Which is broken down into 1 hour for the intervention (the video game itself); 1 hour for online one-on-one qualitative audio interviews via Zoom after completing the game, and after a 3-week follow-up; and 1 hour for the three-questionnaire assessments at baseline (pre-intervention), immediate post-intervention, and after a 3-week follow-up.

Who is carrying out the research?

The data are being collected by Prof. Andrew Kemp and research assistant Tom Gordon of the Psychology Department within the College of Human and Health Sciences at Swansea University, as well as Dr. Darren Edwards of The Department of Public Health, Policy, and Social Sciences. The research has been approved by the Department of Psychology's Research Ethics Committee.

What happens if I agree to take part?

If you are happy to take part in this research project, please click "accept" at the bottom of the consent section below, which will then allow you to complete a baseline questionnaire assessment. The questionnaire assessment includes a range of multiple-choice questions. These questions cover a range of factors including depression, anxiety, stress, and wellbeing. There are three questionnaire assessments to complete, the first of which is a baseline assessment prior to the intervention commencing, the second will be completed following the intervention, and a follow-up assessment 3 weeks following the baseline assessment. All of the questionnaires ask the same questions with the addition of demographic questions at baseline assessment, and the second also asking about adherence to the intervention.

The intervention will consist of completing a one-hour session of playing an ACT-based video game, followed by completion of a short reflection workbook. The game itself will be completed by the participant at home, using a mobile phone or any iOS device. This is an adventure game that will teach you about psychological resilience. In the game, you will control the character Steve as he embarks on a journey into his own mind, you will help as he confronts painful past memories and learns how to cope with his full potential.

For peer review only - http://bmjopen.bmj.com/site/about/guidelines.xhtml

You will also be interviewed, which will be conducted online via zoom, regarding your experiences of the game, focusing on what you felt did and did not work. The interviews will be conducted at two points during the study, post-intervention, and after a 3-week follow-up. Interview audio will be recorded and transcribed, following which the recordings and any personal information will be immediately deleted.

Are there any risks associated with taking part?

We are not aware of any significant risks associated with participation. While some of the questions will ask about existing states of mental health (e.g., levels of current anxiety and depression) – which some people may find uncomfortable – the video game has developed in accordance with recent developments in psychological science to help you manage these, and we expect that participation in this study will help to ameliorate these feelings.

If you feel affected by any issues raised by this research and would like to discuss any concerns, please contact the principal investigators of this study as indicated in the contact details at the bottom of this information sheet. If you feel that you would benefit from further psychological or psychiatric support, we advise you to contact your GP (family doctor) in the first instance. Further information on mental wellbeing can be found at https://www.mind.org.uk/. Additional mental health information can be found at https://www.nhs.uk/mental-health/.

This research has been approved by the Department of Psychology's Research Ethics Committee.

Data Protection and Confidentiality

Your data will be processed in accordance with the Data Protection Act 2018 and the General Data Protection Regulation 2016 (GDPR). All information collected about you will be kept strictly confidential. Your data will only be viewed by the researcher/research team.

Standard ethical procedures will involve you providing your consent to participate in this study by ticking the consent box in the consent section

All electronic data will be stored on password-protected computers.

Please note that the data to be collected for our study will be made anonymous once you have completed all stages of the research, and your response data will not hold any personally identifiable information. We will allocate you with an identifier code, and keep your email information for the duration of the study, so that we can communicate with you at the time points. We will delete contact emails at the end of the study.

All data deposited in Swansea University's OneDrive for Business service is stored within Microsoft's data centres located in the EU. Swansea University retains full ownership and control over the data and is satisfied that the data is properly secured and protected.

The contractual agreements between Microsoft and Swansea University have been negotiated by the JISC on behalf of the UK HE sector and abide by all relevant UK and European legislation. In addition, the UK Government has granted Microsoft Azure and Microsoft Office 365 "OFFICIAL" accreditation. This means that they are accredited to hold or transact public sector data for business conducted at the OFFICIAL level of Security Classification.

What will happen to the information I provide?

Version 4 4 November 2022

For peer review only

An analysis of the information will form part of our report at the end of the study and may be presented to interested parties and published in scientific journals and related media. *Note that all information presented in any reports or publications will be anonymous and unidentifiable.*

Is participation voluntary and what if I wish to later withdraw?
Your participation is entirely voluntary – you do not have to participate if you do not want to. If you decide to participate, but later wish to withdraw from the study, then you are free to withdraw at any time, without giving a reason and without penalty (simply close your web browser). All identifying information will be stripped from collected data once you have completed all phases of the study. If you wish to withdraw at any time during the study, all personal information will be immediately deleted.

Data Protection Privacy Notice
The data controller for this project will be Swansea University. The University Data Protection Officer provides oversight of university activities involving the processing of personal data, and can be contacted at the Vice Chancellors Office.

Your personal data will be processed for the purposes outlined in this information sheet. Standard ethical procedures will involve you providing your consent to participate in this study by ticking the consent box on the consent page.

The legal basis that we will rely on to process your personal data will be processing in line with public interest, scientific and statistical purposes.

How long will your information be held?
Anonymised data will be preserved and accessible online, as is encouraged by developments in open science.

What are your rights?
You have a right to access your personal information, to object to the processing of your personal information, to rectify, to erase, to restrict and to port your personal information. Please visit the University Data Protection webpages for further information in relation to your rights.

Any requests or objections should be made in writing to the University Data Protection Officer:-

University Compliance Officer (FOI/DP)
Vice-Chancellor's Office
Swansea University
Singleton Park
Swansea
SA2 8PP
Email: dataprotection@swansea.ac.uk

How to make a complaint
If you are unhappy with the way in which your personal data has been processed you may, in the first instance contact the University Data Protection Officer using the contact details above.

If you remain dissatisfied then you have the right to apply directly to the Information Commissioner for a decision. The Information Commissioner can be contacted at: -

Information Commissioner's Office,
Wycliffe House,

For peer review only - http://bmjopen.bmj.com/site/about/guidelines.xhtml

Water Lane,
Wilmslow,
Cheshire,
SK9 5AF
www.ico.org.uk

What if I have other questions?
If you have further questions about this study, please do not hesitate to contact us:

Tom Gordon
Research Assistant
Swansea University
tom.gordon@swansea.ac.uk

Dr. Darren Edwards
Department of Public Health, Policy, and Social Sciences.
Swansea University
d.j.edwards@swansea.ac.uk

Prof. Andrew Kemp
Department of Psychology
Swansea University
a.h.kemp@swansea.ac.uk

For peer review only - http://bmjopen.bmj.com/site/about/guidelines.xhtml

Participant Consent Form

Swansea University
Prifysgol Abertawe

Project title: **A novel ACT-based video game to support mental health through embedded learning**

You must be age 18 or over to complete this online survey.

Name and Contact details of the principal researchers: Tom Gordon tom.gordon@swansea.ac.uk, Prof. Andrew Kemp a.h.kemp@swansea.ac.uk, Dr. Darren Edwards d.j.edwards@swansea.ac.uk.

This study is being conducted by Swansea University, Faculty and life sciences.

- I (the participant) consent to participate in the study
- I confirm that I have read and understand the information provided in relation to this study.
- I understand that this study will involve three phases, taking place over a period of three weeks. Within which I will complete a 1-hour therapy-based mobile phone videogame at home, that includes potentially upsetting themes relating to mental health.
- I understand that partaking in this study involves one-on-one online interviews using Zoom audio, and that any identifiable personal information will be immediately deleted following transcription.
- I understand that I have the option of undergoing electrocardiograph recording at three stages during the study, which will take place at Swansea University.
- I understand that my participation is voluntary. I understand that I am free to withdraw at any time during the study but once I have completed all phases of the study, withdrawal will not be possible because data will be completely anonymised.
- I understand what my role will be in this research, and all my questions have been answered to my satisfaction.
- I have been informed that the information I provide will be safeguarded.
- I am happy for the information I provide to be used (anonymously) in academic papers and other formal research outputs, however my name will not be published so anonymity is ensured.

For peer review only

- I agree to the researchers processing my personal data in accordance with the aims of the study described in the participant information.

- I am age 18 years or above.

If you agree with all statements listed above, click **YES** (I consent).

If you disagree with any of the statements above, click **NO** (I do not consent).

For peer review only

DEBRIEF FORM

Title of project: **A novel ACT-based video game to support mental health through embedded learning**

Thank you for taking part in our research. Now that your contribution has finished, let us explain the rationale behind this work.

We are interested in how a video game based on recent developments in clinical psychology might enable learning of psychological resilience skills through play. Typically, psychotherapeutic interventions are delivered through face to face sessions, but there is an increasing need for psychological support that is delivered through an online medium such as the video you have been playing in our study.

This work therefore builds on previous efforts to teach people important skills in psychological resilience through psychoeducation and embedded learning. Specifically, the game is designed to build psychological flexibility, which is a key outcome of an ACT intervention. Psychological flexibility within ACT refers to the promotion of positive mental health, contact with present emotions, wellbeing, and positive emotions.
We hope that information gained from this study will be useful for further developing our ACT-based video game and expanding further research in this area.

If you feel affected by any issues raised by this research and would like to discuss any concerns, please contact the principal investigators of this study as indicated in the details provided below. If you feel that you would benefit from further psychological or psychiatric support, we advise you to contact your GP (family doctor) in the first instance. Further information on mental wellbeing can be found at https://www.mind.org.uk/. Further information regarding mental health can be found at https://www.nhs.uk/mental-health/.

Tom Gordon<br>Research Assistant<br>Swansea University<br>tom.gordon@swansea.ac.uk<br><br>Dr. Darren Edwards<br>Department of Public Health, Policy, and Social Sciences.<br>Swansea University<br>d.j.edwards@swansea.ac.uk	Prof Andrew Kemp<br>Department of Psychology<br>Swansea University<br>a.h.kemp@swansea.ac.uk

For peer review only

CONSORT 2010 checklist of information to include when reporting a pilot or feasibility trial*

Section/Topic	Item No	Checklist item	Reported on page No
**Title and abstract**			
	1a	Identification as a pilot or feasibility randomised trial in the title	1
	1b	Structured summary of pilot trial design, methods, results, and conclusions (for specific guidance see CONSORT abstract extension for pilot trials)	2/3
**Introduction**			
Background and objectives	2a	Scientific background and explanation of rationale for future definitive trial, and reasons for randomised pilot trial	4-9
	2b	Specific objectives or research questions for pilot trial	8-9
**Methods**			
Trial design	3a	Description of pilot trial design (such as parallel, factorial) including allocation ratio	9
	3b	Important changes to methods after pilot trial commencement (such as eligibility criteria), with reasons	8
Participants	4a	Eligibility criteria for participants	10
	4b	Settings and locations where the data were collected	10
	4c	How participants were identified and consented	10
Interventions	5	The interventions for each group with sufficient details to allow replication, including how and when they were actually administered	12
Outcomes	6a	Completely defined prespecified assessments or measurements to address each pilot trial objective specified in 2b, including how and when they were assessed	10-12
	6b	Any changes to pilot trial assessments or measurements after the pilot trial commenced, with reasons	N/A
	6c	If applicable, prespecified criteria used to judge whether, or how, to proceed with future definitive trial	N/A
Sample size	7a	Rationale for numbers in the pilot trial	10
	7b	When applicable, explanation of any interim analyses and stopping guidelines	N/A
Randomisation:			
Sequence generation	8a	Method used to generate the random allocation sequence	N/A
	8b	Type of randomisation(s); details of any restriction (such as blocking and block size)	N/A
Allocation concealment mechanism	9	Mechanism used to implement the random allocation sequence (such as sequentially numbered containers), describing any steps taken to conceal the sequence until interventions were assigned	N/A

For peer review only - http://bmjopen.bmj.com/site/about/guidelines.xhtml

Implementation	10	Who generated the random allocation sequence, who enrolled participants, and who assigned participants to interventions	N/A
Blinding	11a	If done, who was blinded after assignment to interventions (for example, participants, care providers, those assessing outcomes) and how	N/A
	11b	If relevant, description of the similarity of interventions	N/A
Statistical methods	12	Methods used to address each pilot trial objective whether qualitative or quantitative	14-17
**Results**			
Participant flow (a diagram is strongly recommended)	13a	For each group, the numbers of participants who were approached and/or assessed for eligibility, randomly assigned, received intended treatment, and were assessed for each objective	17
	13b	For each group, losses and exclusions after randomisation, together with reasons	17
Recruitment	14a	Dates defining the periods of recruitment and follow-up	17
	14b	Why the pilot trial ended or was stopped	N/A
Baseline data	15	A table showing baseline demographic and clinical characteristics for each group	30
Numbers analysed	16	For each objective, number of participants (denominator) included in each analysis. If relevant, these numbers should be by randomised group	17
Outcomes and estimation	17	For each objective, results including expressions of uncertainty (such as 95% confidence interval) for any estimates. If relevant, these results should be by randomised group	30
Ancillary analyses	18	Results of any other analyses performed that could be used to inform the future definitive trial	17-29
Harms	19	All important harms or unintended effects in each group (for specific guidance see CONSORT for harms)	N/A
	19a	If relevant, other important unintended consequences	N/A
**Discussion**			
Limitations	20	Pilot trial limitations, addressing sources of potential bias and remaining uncertainty about feasibility	33-34
Generalisability	21	Generalisability (applicability) of pilot trial methods and findings to future definitive trial and other studies	31
Interpretation	22	Interpretation consistent with pilot trial objectives and findings, balancing potential benefits and harms, and considering other relevant evidence	31-35
	22a	Implications for progression from pilot to future definitive trial, including any proposed amendments	34-35
**Other information**			
Registration	23	Registration number for pilot trial and name of trial registry	3
Protocol	24	Where the pilot trial protocol can be accessed, if available	35
Funding	25	Sources of funding and other support (such as supply of drugs), role of funders	35
	26	Ethical approval or approval by research review committee, confirmed with reference number	36

For peer review only - http://bmjopen.bmj.com/site/about/guidelines.xhtml

Citation: Eldridge SM, Chan CL, Campbell MJ, Bond CM, Hopewell S, Thabane L, et al. CONSORT 2010 statement: extension to randomised pilot and feasibility trials. BMJ. 2016;355. This is an Open Access article distributed in accordance with the terms of the Creative Commons Attribution (CC BY 3.0) license (http://creativecommons.org/licenses/by/3.0/), which permits others to distribute, remix, adapt and build upon this work, for commercial use, provided the original work is properly cited.

*We strongly recommend reading this statement in conjunction with the CONSORT 2010, extension to randomised pilot and feasibility trials, Explanation and Elaboration for important clarifications on all the items. If relevant, we also recommend reading CONSORT extensions for cluster randomised trials, non-inferiority and equivalence trials, non-pharmacological treatments, herbal interventions, and pragmatic trials. Additional extensions are forthcoming: for those and for up-to-date references relevant to this checklist, see www.consort-statement.org.

For peer review only

For peer review only - http://bmjopen.bmj.com/site/about/guidelines.xhtml

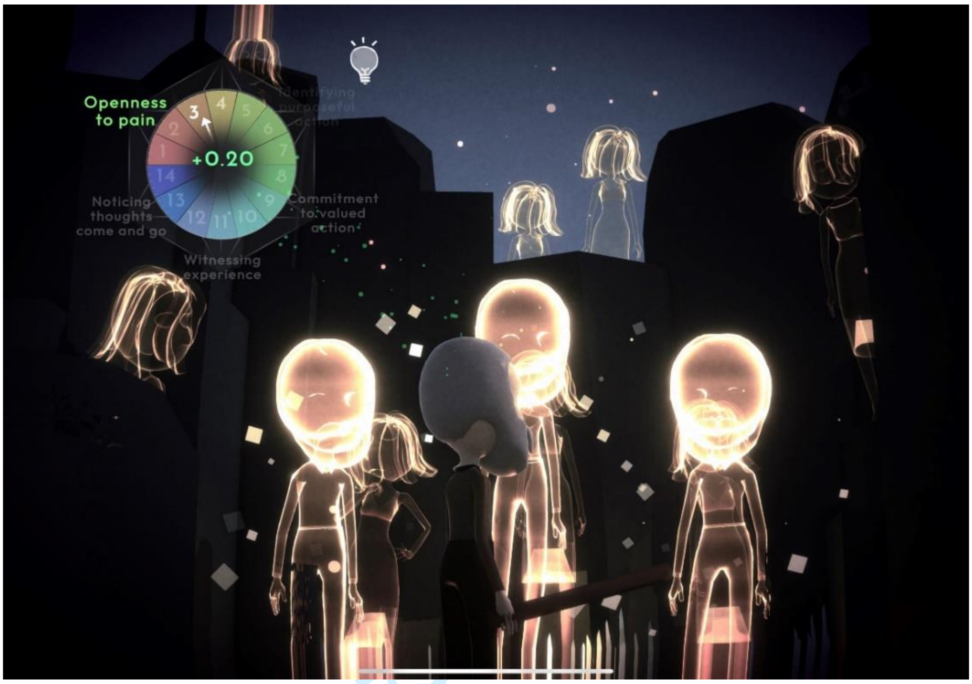

For peer review only

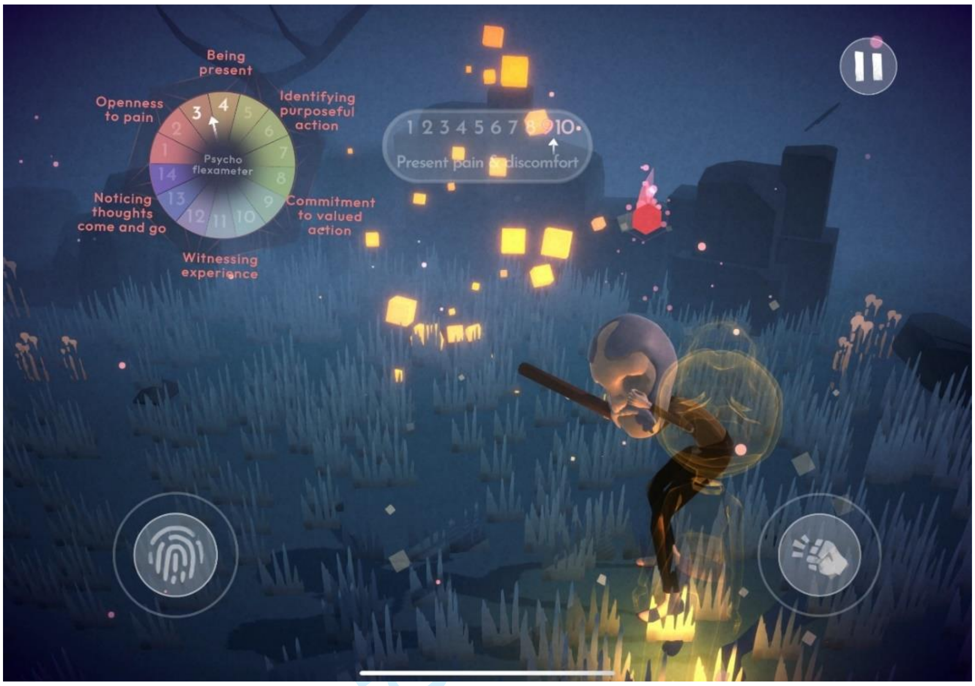

For peer review only

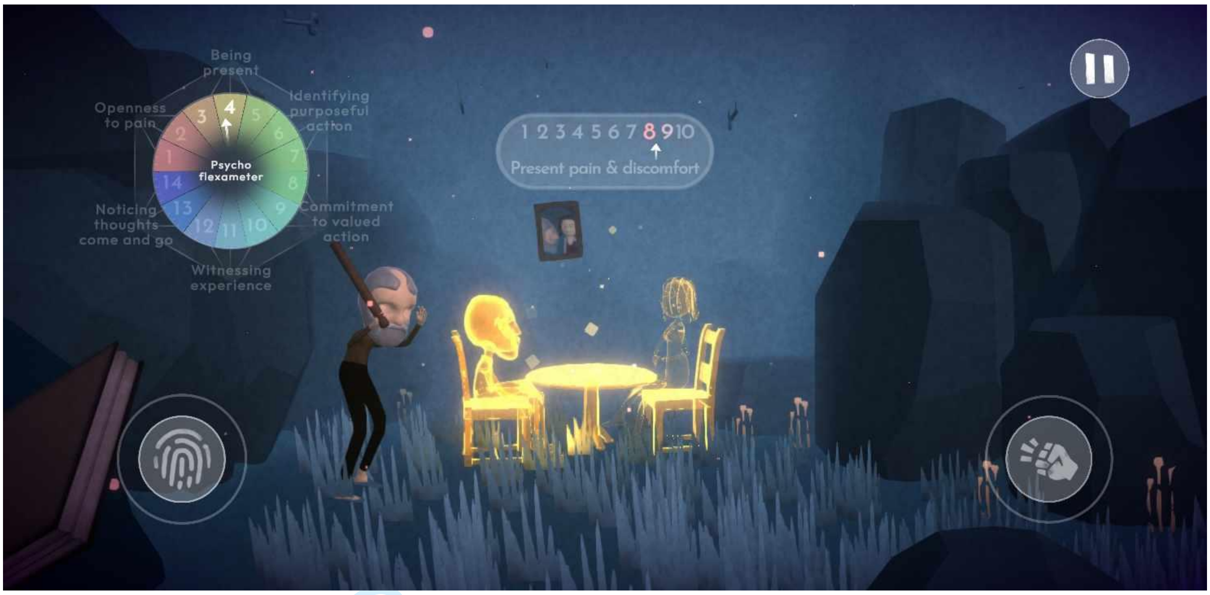

For peer review only

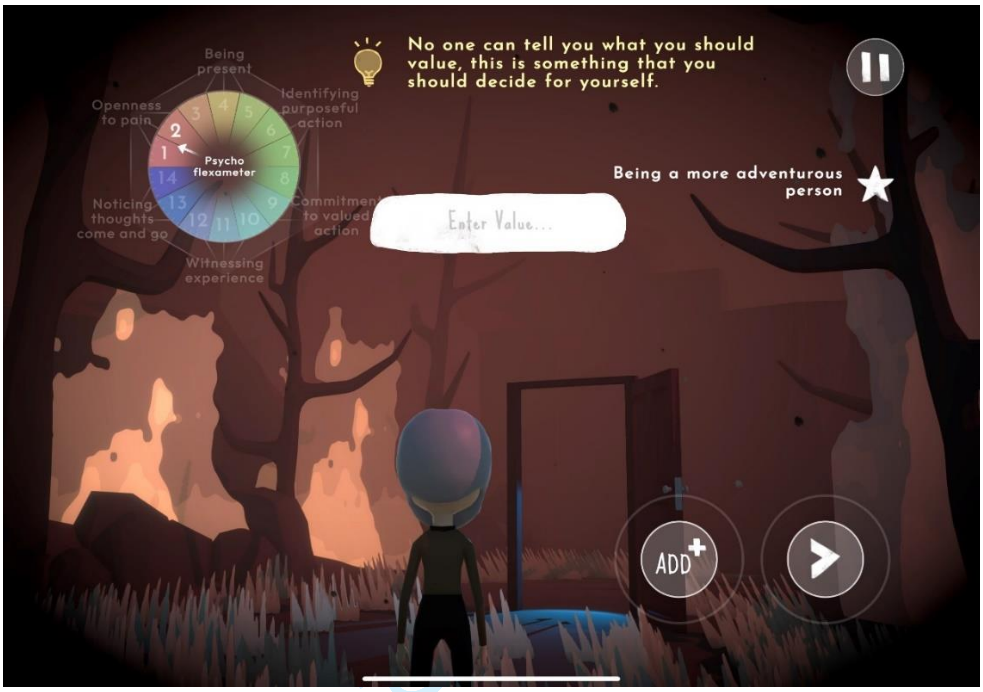

For peer review only

Mixed-methods feasibility outcomes for a novel ACT-based videogame ‘ACTing Minds’ to support mental health

Journal:	*BMJ Open*
Manuscript ID	bmjopen-2023-080972.R2
Article Type:	Original research
Date Submitted by the Author:	28-Feb-2024
Complete List of Authors:	Gordon, Tom; Swansea University, School of Psychology ; Swansea University<br>Kemp, Andrew; Swansea University, Psychology<br>Edwards, Darren; Swansea University, Department of Public Health
**Primary Subject Heading**:	Mental health
Secondary Subject Heading:	Public health
Keywords:	MENTAL HEALTH, Health Education, Depression & mood disorders < PSYCHIATRY

SCHOLARONE™ Manuscripts

I, the Submitting Author has the right to grant and does grant on behalf of all authors of the Work (as defined in the below author licence), an exclusive licence and/or a non-exclusive licence for contributions from authors who are: i) UK Crown employees; ii) where BMJ has agreed a CC-BY licence shall apply, and/or iii) in accordance with the terms applicable for US Federal Government officers or employees acting as part of their official duties; on a worldwide, perpetual, irrevocable, royalty-free basis to BMJ Publishing Group Ltd ("BMJ") its licensees and where the relevant Journal is co-owned by BMJ to the co-owners of the Journal, to publish the Work in this journal and any other BMJ products and to exploit all rights, as set out in our licence.

The Submitting Author accepts and understands that any supply made under these terms is made by BMJ to the Submitting Author unless you are acting as an employee on behalf of your employer or a postgraduate student of an affiliated institution which is paying any applicable article publishing charge ("APC") for Open Access articles. Where the Submitting Author wishes to make the Work available on an Open Access basis (and intends to pay the relevant APC), the terms of reuse of such Open Access shall be governed by a Creative Commons licence – details of these licences and which Creative Commons licence will apply to this Work are set out in our licence referred to above.

Other than as permitted in any relevant BMJ Author's Self Archiving Policies, I confirm this Work has not been accepted for publication elsewhere, is not being considered for publication elsewhere and does not duplicate material already published. I confirm all authors consent to publication of this Work and authorise the granting of this licence.

For peer review only

Mixed-methods feasibility outcomes for a novel ACT-based videogame 'ACTing Minds' to support mental health

Tom C. Gordon[1,2], Andrew H. Kemp[2], & Darren J. Edwards[1]

[1]Department of Public Health, Swansea University, UK

[2]School of Psychology, Swansea University, UK

For peer review only

Darren J. Edwards,

Department of Public Health,

Swansea University,

Swansea,

SA2 8PP,

UK

Email: d.j.edwards@swansea.ac.uk

Short title: Feasibility outcomes ACTing Minds

For peer review only - http://bmjopen.bmj.com/site/about/guidelines.xhtml

Abstract

Objectives: To determine the feasibility and acceptability of 'ACTing Minds', a novel single-player adventure videogame based on acceptance and commitment therapy (ACT).

Design: A single-arm, mixed-methods repeated measures feasibility study.

Setting: Intervention and questionnaires were completed at home by participants. Semi-structured interviews were also conducted at home via the Zoom platform.

Participants: Thirty-six participants were recruited into the study, 29 completed all phases of the feasibility design. Eligibility criteria required participants to be over the age of 18 and self-reporting experiencing ongoing depression, anxiety, or stress.

Intervention: Participants completed a single session of the 'ACTing Minds' videogame, lasting approximately 1-hour, designed to educate users on key principles from acceptance and commitment therapy (ACT).

Primary outcome measures: Participant recruitment and retention, questionnaire completion, long-term intervention adherence, and acceptability of the intervention. Reflexive thematic analysis was conducted on semi-structured interviews run immediately postintervention and 3-weeks later.

Secondary outcome measures: Measures of depression, anxiety, stress, psychological flexibility, social connectedness, and wellbeing were assessed at baseline, immediately following intervention completion, and after a 3-week follow-up period. We used a standardised battery of questionnaires.

Primary results: Twenty-nine participants completed the study. A reflexive thematic analysis indicated that participants responded positively to the intervention and the study at all stages. Themes reflect participants' desire for an engaging therapeutic experience, use of

For peer review only

game for exploring emotions, as well as their perspectives on how they had applied their learning to the real-world.

Secondary results: Quantitative results indicated small to large effect sizes associated with decreases in depression ($\eta p2$ = .011), anxiety ($\eta p2$ = .096), stress ($\eta p2$ = .108), and increases in psychological flexibility ($\eta p2$ = .060), social connectedness ($\eta p2$ =.021), well-being ($\eta p2$ =.011), and participation in usual activities ($\eta p2$ = .307).

Conclusions: Implementation of the 'ACTing Minds' intervention is warranted, based on both qualitative and quantitative outcomes.

Trial Registration number NCT04566042

Strengths and limitations of this study

- Mixed methods approach, combining thematic analysis of interviews and quantitative questionnaires.
- Collection of quantitative data at three time points and qualitative at two time points, allowing the process of change and identification of patterns to be examined.
- Remote data collection due to COVID restrictions meant that participants could not be directly observed while completing the intervention. We were also unable to record planned psychophysiological measurements of wellbeing such as heart rate variability.
- Reliance on self-report measures introduces the potential for bias.

For peer review only

Introduction

The global prevalence of common mental disorders and a lack of available resources for the identification and treatment of those with such conditions underpin an increasing burden on society (1). The Global Burden of Disease study conducted in 2017, reported a UK prevalence rate of 4.12% for depression, and 4.65% for anxiety disorders (2). Since this estimation, events such as the COVID-19 pandemic and the increasing threat of climate crises have had a substantial impact on societal wellbeing. A meta-analysis including 14 studies (n=46,158) found that 32% of adults in the UK experienced moderate to severe depressive symptoms in 2022, and 31% of adults reported high levels of anxiety (3), indicating a societal increase of 27.88% for depression, and 26.35% increase for anxiety between 2017 and 2022. These findings suggest the need for a transition towards population-wide strategies aimed at fostering psychological resilience on a broader scale.

To positively impact societal wellbeing, contemporary interventions must be affordable and widely accessible. Presently, the demand for mental health services far exceeds the available human resources required to meet this need. A study conducted for the Centre for Mental Health estimated that services cost the UK economy approximately £105 billion per year in 2020, 4.8% of the UK's annual GDP (4). Despite substantial funding of £34 billion to public mental health support and services, the prevalence of psychological disorders is high and only 33% of adults with depression and anxiety receive treatment in England (5), highlighting an urgent need for innovation.

There are numerous barriers to accessing psychological interventions, including a shortage of therapists, long waiting times, and societal stigma of accessing psychological treatment (6). A potential solution to these issues might be found in digital health interventions (DHIs). We live in an age of heavy digital media consumption, especially in the

For peer review only - http://bmjopen.bmj.com/site/about/guidelines.xhtml

West where at least 90% of UK adults use the internet regularly (7). We also know that during the COVID-19 pandemic, there were significant increases in online video consumption, social media usage, remote work, online news consumption, and video gaming (8). COVID-19 contributed to significant social isolation and further disconnection from nature, further contributing to increases in mental health conditions (9). We argue that there is an opportunity to leverage technological advancements and the growing use of technology to develop psycho-educational tools necessary to support mental health at scale.

DHIs have already been utilised in a variety of contexts for promoting wellbeing, from delivering healthcare and education to personalised diet and fitness plans. Mobile apps and online platforms offer guided meditation, breathing exercises, sleep tracking, and relaxation programs. Such applications might aim to enhance overall wellbeing, reduce stress, improve sleep quality, or cultivate mindfulness practices (10). We suggest that effectively addressing wellbeing at a population level should involve the development of DHIs that consider acceptability, feasibility, and widespread appeal.

Compared with alternative forms of media, videogame DHIs offer several advantages. By design they are interactive, applying behavioural principles for controlling and modifying behaviour (11), making them uniquely captivating. In the UK, the COVID-19 pandemic led to a substantial increase in the number of people playing video games, with males increasing their use from 46% to 63% and females increasing their use from 32% to 56% in 2022 (12). Innovations in the use of video games for treating mental health issues have wide potential applications, potentially offering a platform for individuals to explore their ongoing relationship with their emotions in a supportive environment.

In theory, by practising skills derived from psychological therapies (such as ACT) within the game context, individuals can transfer these skills to real-life situations to improve their overall quality of life. Certain games specifically designed for therapeutic purposes,

For peer review only

such as ‘SPARX’ for depression (13) or ‘Elude’ for anxiety (14), guide players through interactive challenges and cognitive exercises for developing emotional regulation skills. Videogames are also being used in clinical settings to promote wellbeing outcomes. One game designed for this purpose, ‘Dojo’ (15), aims to treat anxiety by training users in breathing techniques, muscle relaxation, positive thinking, and guided imagery, utilising heart-rate variability (HRV) biofeedback. However, when compared against a standard commercial game ‘Rayman 2’ (control condition), a full pre-post RCT (n=138) found that playing either game significantly reduced participant anxiety at a 3-month follow-up, and there were no significant differences between these two games at reducing anxiety for this time period (15). Reasoning for this might be that ‘Dojo’ failed to develop the psychoeducational skills in the participants that it aimed to impart, or that both games only reduced anxiety by distracting (as a form of avoidance) participants from anxiety-provoking thoughts (16). The researchers concluded that ‘Dojo’ had crucial design issues that needed to be addressed including a lack of clear theoretical and therapeutic frameworks, and that research assessing the real-world effectiveness of video games in the treatment of mental health issues should require an appropriate methodology for understanding the underlying causes of improvement.

A study aiming to explore the wellbeing effects of playing video games on gamers during the COVID-19 pandemic (n=781) found that time spent playing had significantly increased in 71% of participants, and 58% of participants reported that playing games had positively impacted their wellbeing (17). The researchers conducted an online survey including both closed and open-ended questions, then conducted a thematic analysis to identify the causes of improvement. Themes of escape, cognitive stimulation, stress relief, agency, and socialisation were most associated with feelings that playing video games had increased wellbeing. The development of an effective DHI videogame should consider such

For peer review only - http://bmjopen.bmj.com/site/about/guidelines.xhtml

factors whilst also building on strong theoretical and therapeutic foundations that facilitate the uptake of such tools.

The 'ACTing Minds' videogame, developed in line with our intervention protocol (16), was designed to be a comprehensive transdiagnostic intervention that will integrate lessons from acceptance and commitment therapy (ACT) (18). In contrast to prior mentioned DHIs, commonly rooted in the medical model and second-wave approaches, ACT as a third-wave behavioural therapy focuses on promoting psychological flexibility rather than the elimination of disorder symptoms (19). More specifically, ACT aims to promote psychological flexibility through six core processes of change (20). These are: (1) present moment awareness: the practice of being in the here and now; (2) acceptance: the practice of being open to the range of human emotional experience, as opposed to experiential avoidance; (3) Cognitive defusion: the act of recognising the self as separate from thoughts, and not interpreting them literally; (4) values: identifying ones' personal values in contrast to perceived expectations, of which drive us toward self-direction and purpose; (5) action: a commitment to ones' values, facilitating the development of competence through the act of continual practice of alignment with values (21); and (6) self as context: developing an awareness of self that is more than a conceptualised sense of self, one that is flexible and facilitates a sense of connection with others.

Though ACT clinical practice does not focus primarily on reducing mental health symptoms, many studies have indicated that when the individual works towards greater psychological flexibility, many mental health symptoms, and destructive behaviours such as anxiety, depression, stress, pain, and substance misuse, tend to reduce with clinically acceptable small to high effect sizes. This was, for example, identified within a review of 20 meta-analyses, involving 133 studies (n=12,477) that found that ACT was efficacious for treating these disorders (22). The results also showed that ACT was generally superior to

For peer review only

most active intervention conditions (excluding CBT), treatment as usual, and inactive controls.

As such, an ACT-based DHI video game could allow for both greater psychological flexibility as well as a reduction in common mental health issues such as depression and anxiety. This is because developing explicit DHI psychoeducational transdiagnostic skills that promote present moment awareness, values orientation, commitment to action, openness, and acceptance of painful emotions, cognitive defusion, and a transcendental self also have a positive impact on mental health. As a consequence, our videogame may have greater reach and impact than other videogame DHIs that are not based on third-wave psychotherapy, such as 'Dojo', which primarily aims to teach skills for limited emotional regulation and symptom reduction such as avoidance.

The ACT framework has already been adapted to a variety of accessible mediums, including self-help books and mobile phone applications (23). Resources for the education and practice of ACT are freely available through the Association of Contextual Behavioural Science website (see https://contectualscience.org/). ACT-based mobile phone applications are shown to be effective in promoting psychological flexibility (24) and reducing smoking intake (25). Considering this, we believe that choosing ACT as the basis for our game will allow us to harness the advantages of third-wave therapies as transdiagnostic therapeutic tools and integrate these with those of videogames, and if made well, will be engaging, educational, and capable of promoting wellbeing (psychological flexibility) at scale.

Based on our initial protocol (16) (see Supplementary 1), the ACT-based videogame called 'ACTing Mind', has been developed as a psychoeducational tool that teaches users the core processes of ACT through embedded learning. The goal of this research will be to determine the acceptability and feasibility of 'ACTing Minds' for promoting psychological flexibility as well as its clinical relevance for reducing mental distress as a secondary

For peer review only - http://bmjopen.bmj.com/site/about/guidelines.xhtml

outcome. The game teaches skills based on the ACT principles of acceptance, defusion, and values identification. This is a feasibility study, following the Medical Research Council (MRC) framework (26), laying the foundation for a full-scale RCT from which clinical effectiveness will be determined.

Several changes have been made to our originally published protocol (16), it was initially stated that participants would complete five weekly 1-hour sessions where they would play through six parts of the 'ACTing Minds' videogame, each one focusing on a different process of ACT. However, because of funding restrictions, 'ACTing Minds' has been compressed into a single game focusing on the ACT principles of acceptance, values identification, and defusion. Therefore, in this feasibility study, participants will be required to complete a single 1-hour session of 'ACTing Minds'. This meant a significant change to the overall time to complete the study protocol. Originally it was expected to take 3 months between baseline measurements and the final follow-up. Now, one-on-one semi-structured interviews will be conducted immediately postintervention, and after a 3-week follow-up. The research questions are as follows: Is the intervention acceptable and feasible for a full-scale RCT? Is there early evidence for effectiveness in reducing mental distress? Are there any changes in self-reported wellbeing measures following completion of the game? Are participants able to learn ACT principles and apply them in day-to-day life?

Methodology

Design:

This is a single-arm, mixed-methods repeated measures study, designed to determine the feasibility and acceptability of an ACT-based video game called 'ACTing Minds' that has been designed for individuals reporting mild to moderate anxiety, depression, and stress. Data was collected at baseline, immediately post-intervention, and 3-weeks post-intervention. Data collection was conducted between November 1st and December 31st, 2022.

For peer review only - http://bmjopen.bmj.com/site/about/guidelines.xhtml

Study Setting:

The study was conducted entirely online by participants, including the intervention (via a link to the mobile app; https://shorturl.at/iqFGI), quantitative assessment (via the Qualtrics platform), and qualitative interviews (via the Zoom platform). Strict recommendations were given to participants to ensure they were in a quiet room and without disruption for all study components.

Participants:

Thirty-six participants were recruited, 29 of which completed all phases of the study. Participants were recruited using purposive sampling methods, they were required to be at least 18 years of age, self-reporting ongoing experience of mild to moderate depression, anxiety, or stress within their day-to-day life, and able to read, write, and speak English. The sample size was justified on the basis of past research reporting the median numbers of participants recruited for similar feasibility studies incorporating both quantitative and qualitative elements (27). Advertisements were posted at Swansea University notice boards and on social media pages (Facebook mental health community groups). Participants were recruited between October 1st and December 1st, 2022, they completed a consent form (see Supplementary 2) after reading the study information sheet (see Supplementary 3) and were given a debrief sheet (see Supplementary 4) following completion of the study, each are included as supplementary materials.

Primary outcome measures:

Feasibility outcomes were determined using the MRC framework (26) and reported in line with CONSORT guidelines (28) (see Supplementary 5). Feasibility measures included data relating to participant recruitment and retention including the number of participants willing to take part, and completing each stage of the study (i.e., intervention, questionnaires,

For peer review only - http://bmjopen.bmj.com/site/about/guidelines.xhtml

interviews, and follow-up). Acceptability and efficacy of the intervention were assessed through thematic analysis of semi-structured interviews conducted immediately post-intervention and 3-weeks post-intervention, which focussed on participant experiences with ‘ACTing Minds’. The first interview (see Open Science Framework for first interview protocol: https://osf.io/5fvjs) asked questions about: (1) the acceptability of the intervention; (2) what they learned from the intervention; (3) suggestions for further improvement; (4) whether there were any difficulties (barriers) in taking part; (5) aspects they liked and disliked; and (6) were there any adverse effects that they noticed whilst playing the game. This was followed by a second interview (see Open Science Framework for second interview protocol: https://osf.io/32epw) that was focused more on the real-world impact that ACTing Minds had on their lives and their experience over the 3-week period. Specifically, the second interview asked: (1) about their retrospective experience in taking part in the ACTing Minds intervention; (2) how much they remember about the core ACT concepts; (3) were any aspects more memorable than others; (4) had they implemented any of the ACT concepts that they learned whilst playing the game into their day to day lives; (5) had they found that any particular ACT concepts were more applicable to their everyday lives than others; (6) would they reuse the intervention; and (7) had they noticed any adverse effects in the three weeks since playing ACTing Minds.

Secondary outcome measures:

Questionnaires were distributed at three points in time (baseline, immediate postintervention, and 3-month follow-up). A rule was created in Qualtrics requiring participants to complete every questionnaire item in order to finish the survey, which included the following questionnaires:

Depression Anxiety Stress Scales (DASS-21): a measure of general psychological distress with good construct validity (confirmatory factor analysis of 0.94), that can be

For peer review only - http://bmjopen.bmj.com/site/about/guidelines.xhtml

broken down into subscales relating to stress, anxiety, and depression. It has good internal reliability as measured through Cronbach's alpha coefficients, which are 0.88 for depression, 0.82 for anxiety, 0.90 for stress, and 0.93 for the total scale (29).

Acceptance and Action Questionnaire-second version (AAQ-II): a questionnaire of seven items, assessing psychological inflexibility by gauging one's capacity to embrace and remain receptive to challenging thoughts and emotions, while also actively participating in meaningful actions despite their presence. A higher score indicates higher psychological inflexibility. The measure has good construct validity with a Cronbach's alpha coefficient of 0.84 (30).

Social Connectedness Scale (adapted from Russell's (1996) UCLA Loneliness Scale (31): this measure involves two questions: (1) "During social interactions, I feel 'in tune' with the person/s around me", and (2) "During social interactions, I feel close to the person/s". The Cronbach's alpha coefficients for these two items ranged from 0.80 to 0.98 (M=0.94, SD=0.03) (32).

Warwick-Edinburgh Mental Well-Being Scale (WEMWBS): a metric that emphasises the positive facets of mental health, aiming to evaluate overall psychological well-being. This measure has good internal consistency with a Cronbach's alpha coefficient of 0.89 (student sample) and 0.91 (general population sample) (33).

EuroQol Five Dimensions (EQ5D) (34): a measure for health-related quality of life. There are five components within this measure which assess mobility, self-care, usual activities, pain, discomfort, anxiety, and a visual analogue scale for measuring current health status.

Intervention:

For peer review only

The intervention comprised of an ACT-based video game intervention called 'ACTing Minds', developed and designed by author DJE. Participants attended a single session lasting approximately 1-hour, during which they completed four in-game chapters.

The intervention teaches three core principles of ACT through embedded learning, meaning that the player should gain ACT-based skills while completing in-game objectives, without being directly taught those skills. These skills include 'Acceptance', 'Cognitive Defusion', and 'Values Identification'. Embedded learning refers to the incorporation of educational elements into the gameplay itself, in contrast to explicit lessons. In this context, it involves designing the game in a way that promotes psychological flexibility oriented behaviours derived from ACT, such that the adoption values orientation, present moment awareness, openness to pain and cognitive defusion. In 'ACTing Minds,' an example of embedded learning is the 'Psychoflexameter' (see Figure 1A), which serves as a gamified version of the Hexaflex (see Figure 1B), a model used in ACT to illustrate both the theory and goals for clinical change (35). Similar to the Hexaflex, the 'Psychoflexameter' showcases the 6 core processes of ACT. Initially introduced to players during the first ACT-oriented activity in the game, which emphasises acceptance, the 'Psychoflexameter' remains visible in the corner of the screen throughout the game while players engage in other ACT-related activities. As players exhibit ACT-consistent behaviours, they earn points and gradually increase the dial on the centre of the 'Psychoflexameter', and the text reflecting the ACT processes that the players use lights up green (see Supplementary 6). If players exhibit ACT-inconsistent behaviours, they lose points and the 'Psychoflexameter' lights up red (see Supplementary 7).

---**Figure 1 Here**---

The game starts with a text-based chapter, telling the story of a depressed individual, 'Steve', who has recently lost his wife in an accident, he is feeling depressed, isolated, and lonely.

For peer review only

The character has built a 'mind escape machine' intending to enter his own mind to destroy and suppress his unwanted painful thoughts and memories. The player takes control of 'Steve' in chapter two, where they see him in a state of mental distress at his home, surrounded by items that are reminders of his lost wife. At this stage, participants learn how to control the character using an onscreen directional stick and interact with the environment by touching key elements with their finger on their mobile phone or tablet.

Participants then engage with ACT content within chapters three and four, which begin with the character entering his mind (via the mind escape machine), walking around and viewing painful representations of his memories (of his lost wife). Chapter three focuses on 'Acceptance', introducing players to a bar in the centre of the screen indicating the characters' present level of pain and discomfort, as well as the 'Psychoflexameter' dial in the corner of the screen, indicating the character's psychological flexibility. While in the mind of the character, the player can approach memories of 'Steve' (himself) and his wife, which leads to an increase in present pain and allows the option to destroy the memories (this is intended as a metaphorical representation of thought suppression). Destroying memories decreases short-term pain and discomfort, but also removes points from the 'Psychoflexameter'. If the player chooses to destroy the memories (avoidance-based strategies), the world becomes increasingly distorted, and barriers form making the chapter impossible to complete. Alternatively, if the player chooses acceptance-based strategies they can continue the game and learn that acceptance is functionally better than avoidance (see Supplementary 8).

Chapter four focuses on rewarding 'Values identification' and 'Cognitive Defusion'. The player is still in the mind of the character, where they are then asked to reflect on their values, to type them out and make them explicit (see Supplementary 9). Following this, they complete a 'leaves on a stream' task, requiring them to type out any painful thoughts that

For peer review only

they might have and place them on a leaf, watching them as they float downstream (see Figure 1C). Both tasks reward the player by increasing their score on the 'Psychoflexameter'.

Qualitative analysis:

We used a critical realist ontological framework for our reflexive thematic analysis (RTA) as suggested by Braun and Clarke (36) which involves (after an initial familiarisation pre-coding phase) actively conducting both bottom-up (raw data driven and without a conceptual framework in mind) inductive, and top-down (ACT theory driven) deductive stages, to explore participant experience with the "ACTing Minds" intervention. We adopted this inductive (i.e., without framing the raw data through a theoretical model) first stage approach to ensure the themes developed were grounded in the raw data itself rather than potentially being imposed and biased by preconceived theories of the researcher. This is consistent with the critical realist approach which assumes that at least some of reality exists independently of our preconceived knowledge and theories, and the researcher should be actively aware of this. We then followed this with a deductive top-down ACT theory driven second stage that then allows for a more theory informed interpretation of the qualitative data based on ACT concepts and theory. This involved a re-examination of interview content with explicit consideration of how participant statements might relate to our research questions and ACT theory. The codes developed accordingly, transitioning from reflecting explicit semantic content to interpretations of underlying latent themes (via an ACT interpretation). This iteration of induction and deduction is important within RTA, as it allows for a more nuanced qualitative understanding of semi-structured interviews, that goes beyond a purely theory driven lens. This adopts a contextualist epistemological stance of our interpretation, recognising that both researchers' and participants' knowledge and perceptions are shaped by their subjective experiences and situational contexts (36). This combined (critical realist and contextualist epistemological) philosophical foundation guided the application of our

For peer review only

reflexive thematic analysis, serving as a contextualised lens for identifying and generating themes from within the interview data.

The RTA was conducted on two sets of semi-structured interviews (post-intervention n=29, and 3-week follow-up n=29), following the guidelines outlined by Braun and Clarke (36). Both interview sets were analysed separately to gain an understanding of changes in participant perceptions of the intervention and relevant outcomes over time. For reporting on the acceptability of the 'ACTing Minds' intervention, findings and themes from both interviews are summarised in the primary outcomes section of the paper (see Table 1). Interview data was transcribed using Microsoft's automated audio-to-text software, which was then double checked and edited to correct for major spelling or grammatical errors. Throughout the initial data familiarisation phase, multiple points of potential analytical interest were identified. In the coding phase, several hundred codes were initially produced (both inductively and deductively), which were then clustered to make them more manageable and categorised into potential broad patterns of meaning. For the analysis of the first interview set these included: emotional experience; wellbeing needs, perceptions on mental-health education within the game; and participant engagement. For the second set of interviews, these were: application of the game's lessons, perceptions on what was learned, desire for growth, and sense of development.

Themes were then refined in the context of our research questions relating to how the participants experienced 'ACTing Minds', which involved a review of preliminary themes in relation to the codes, the coded data, and the full dataset. We became most interested in the latent ideas underpinning statements relating to how participants utilised the game as a psychoeducational tool. The preliminary theme 'emotional experience' was developed, as it was interpreted from the codes that participants were using the game as a 'base for exploring and accepting difficult emotions'. Further development of the remaining themes emphasised

For peer review only

the processes involved in participant engagement, personal therapeutic goals, and feelings regarding the games' embedded learning features. Themes derived from the codes in the second interview reflect the participant outcomes since playing 'ACTing Minds'. This allowed for an exploration of specific aspects of growth, how the participants implemented insights gained from the game in the weeks that followed, and their reflections on what they had learned through the practical application of these insights in real-life scenarios.

For peer review only

For peer review only - http://bmjopen.bmj.com/site/about/guidelines.xhtml

Table 1.
Themes and sample codes taken from thematic analysis for Interviews 1 and 2

Interview 1 (Immediate postintervention)		
Themes	Raw-data (without preconceived theory) driven inductive codes	ACT-theory driven deductive codes
Theme 1: Desire for an engaging therapeutic experience	Need for wellbeing tools; Surprised by effectiveness; Interest in novelty; Wellbeing development as an enjoyable practice	Psychoflexameter aids engagement; Core ACT concepts useful
Theme 2: Personal process of immersion	Empathy with story; Interest in metaphor, Open mind needed; Personalisation aids relatability	Immersion through visual metaphors and agency; Engagement through ACT therapeutic intent
Theme 3: Game as a base for exploring and accepting difficult emotions	Anxiety while making decisions; Game helped to clear head; Desire for future use as a tool"; "Mood change with game;	ACT skills applicable across emotional scenarios; Long term acceptance benefit despite difficult emotional experience; learning to be open to difficult emotions
Theme 4: Embedded learning game dynamics pros and cons	Concepts made more sense as the game progressed; Lack of instruction, but quickly learned concepts; Conflicting choices	Interpreting ACT metaphors quickly; Priming of ACT-based wellbeing; ACT concepts clear despite confusion with game objectives"
Theme 5: Necessary learning for anyone	Desire to share with others; Growing societal appeal	ACT concepts made tangible; Game provided direction for growth towards ACT-values; ACT-concepts felt relevant
Interview 2 (3-weeks postintervention follow-up)		
Theme 6: Utility in the real world	Sharing lessons with others; Easier time letting go; Built desire to learn more	Applying ACT lessons actively; Potential real world subconscious influence of ACT lessons; Increased perspective-taking in real-life events
Theme 7: Practice facilitates psychological flexibility skills	New interest in wellbeing; Shift in thinking; Combined game and interview helpful	ACT-practice encourages optimism with new ACT knowledge despite present suffering; Trial and error of applying ACT-based lessons; ACT-practice encourages renewed focus on values
Theme 8: Closer alignment to an integrated self (as context), with acceptance, values, as part of who you are	Primed self-reflection; Seeing the bigger picture integration about self; Reduced self-judgment	Integrating present moment awareness, acceptance and values; Dealing with grief through acceptance of self as I am; Acceptance brings the self closer to reality; Self-assurance with values; Self as context identification

For peer review only - http://bmjopen.bmj.com/site/about/guidelines.xhtml

Quantitative analysis

A repeated measures ANOVA was performed using IBM SPSS Statistics 29 (the most up-to-date version at the time of analysis), to compare the effects of playing the 'ACTing Minds' videogame on scores taken from the questionnaires DASS-21, AAQ-II Psychological Flexibility Questionnaire, Warwick-Edinburgh Mental Well-Being Scale, Social Connectedness Score, and EuroQol Five Dimensions.

Descriptive statistics were used to summarise secondary outcome measures (see Table 2). Changes in scores from baseline are reported for each of the measurement time points. Partial eta squared (ηp2) effect sizes were calculated for each independent variable and interpretation was informed by prior literature on the topic (37). Values 0.14 or higher were interpreted to be a large effect, 0.06 – 0.14 were interpreted to be a moderate effect, and 0.01 – 0.06 were interpreted to be a small effect.

Procedure

After recruitment (see 'Participant' section for recruitment), and consenting to take part in the study, participants were given a link to Qualtrics where they completed the battery of questionnaires (see 'Secondary outcome measures' section) at baseline. They were then given a link to the ACTing Minds game (see 'Intervention' section) where they completed this within approximately one hour. Once completed, they then immediately completed the quantitative questionnaires (see 'Secondary outcome measures' section) again on Qualtrics as an immediate post-intervention. This was then followed by completing a 45-minute to 1-hour one-on-one interview which asked participants about their experiences with the game (see 'Primary outcome measures' section). Participants then, after a 3-week follow-up, completed the same questionnaires again, as well as a second interview (see 'Primary outcome measures' section) that focused on real world application of the ACTing Minds intervention.

For peer review only

For peer review only - http://bmjopen.bmj.com/site/about/guidelines.xhtml

Public and patient involvement

Key stakeholders were consulted and involved in the development of this protocol. The Patient Experience and Evaluation in Research (Patient Experience and Evaluation in Research (PEER): https://www.swansea.ac.uk/humanandhealthsciences/research-at-the-college-of-human-and-health/patientexperienceandevaluationinresearchpeergroup/) group in the College of Human and Health Sciences at Swansea University were consulted. This group represented members of the public, students and staff members, several of whom reported that they had experienced depression, anxiety or stress at some point in their lives and emphasised the need for innovative approaches to the delivery of mental health support. The feasibility design was explained to them, and they gave positive feedback about the nature of the design, intervention and outcome measures.

Results

Primary outcome measures

Participant recruitment and retention (feasibility)

Thirty-six participants were recruited through the initial study advertisement between October 1st and December 1st, all of which met eligibility criteria. Six participants did not show up for initial baseline measures, while one participant did not follow through with the intervention (see Figure 2). Only three participants reported why they were not able to attend, where one indicated they had a hospital appointment, another had forgotten about the study date and did not reschedule, while another said they needed to reschedule without giving a reason, but then failed to book in a new date for the study. The other three participants did not report why they could not attend.

---**Figure 2 Here**---

For peer review only - http://bmjopen.bmj.com/site/about/guidelines.xhtml

Participant feedback (acceptability)

Acceptability measures were assessed through thematic analysis of semi-structured interviews (all data is available on the Open Science Framework: https://osf.io/3wuh5/) (38), taken place immediately postintervention and at a 3-week follow-up. The interviews were analysed separately to understand participant perceptions before and after applying lessons learned from the game to their everyday lives. The results from both have been summarised and reported together as key themes.

Theme 1: Desire for an engaging therapeutic experience

Participants expressed interest in the novelty and potential utility of digital mental health applications, specifically in a gamified context. Overall, they felt that a videogame platform provided a uniquely stimulating means of engaging with mental health learning. Participants suggested that psychoeducational tools like 'ACTing minds' may be able to function as an engaging alternative or an adjunct to therapy. For example:

"[…] playing a game that's based for your mental health is really good because you can actually do something therapeutical in the things that I like doing." -P06.

This quote comes from a participant who identifies as a gamer, with little experience or prior interest in mental-health learning. Their statement implies an underlying desire for an engaging, accessible therapeutic environment that he has an interest in. The intervention was also helpful for non-gamers, for example:

"I'm not one to sit down and just watch a video and then put that in practice. I'm someone who actually needs to be involved in something and interact with it, and coursework just reminds me too much of school […] with games it makes me sit, and actually interact because I'm enjoying the game at the same time. It's not just that I need to focus on my mental health,

For peer review only - http://bmjopen.bmj.com/site/about/guidelines.xhtml

I get to play a cool game while learning about my mental health. So yeah, I like I like video games with mental health stuff." -P08

In this situation, the participant does not identify as a gamer and has substantial experience with their own mental health learning and therapy. They, like participant one, also expressed the same desire for a more engaging therapeutic experience and enjoyed the format of learning about mental health via a game. The participant expresses an aversion to learning about mental health in other more traditional ways.

Theme 2: Personal process of immersion

Responses from the semi-structured interviews highlighted the individual differences between participants in their ability to immerse themselves within the content of 'ACTing Minds' and engage directly with the personal decision-making aspects of the game. Responses indicated that the video game immersion and narrative helped them visualise the ACT metaphors and thus helped their understanding of the ACT concepts within the embedded learning environment. For example:

"It plays in a very good way of dealing with visual metaphor, but also the fact that you're given the agency to do it. When you were smashing the memories and I wish I didn't have to, but I have to carry on Steve's journey and I think it's a really good way of showing these memories do hurt, and sometimes you just have to accept the fact that it's going to hurt. Move on like it's explained through the visual and narrative storytelling, and I think it's a really good way to do it." -P18

This participant refers to the visual immersion and having agency within the game as facilitating their learning of ACT concepts. The requirement for participant agency in making emotionally difficult decisions within the game appears to have facilitated the learning of acceptance.

For peer review only - http://bmjopen.bmj.com/site/about/guidelines.xhtml

Learning was also facilitated by the personalised nature of the game, such as selecting difficult personal thoughts in the lily pad exercise, for example:

"[...] it's been like a learning experience, and so the sort of personalised bit at the end with the lily pads and the values. I think it was quite good because then you actually were able to think about like the purpose of the game within your own situation." -P10

Theme 3: Game as a base for exploring and accepting difficult emotions

Participants discussed the idea that the game provided a platform that allowed them to explore their own emotions in an immersive environment. Participants reported experiencing a variety of difficult thoughts and emotions throughout playing the game, and that the game encouraged them to observe and be open to sadness, anxiety, and grief with acceptance. Participants reported feeling that after playing the game they had learned to observe and accept those feelings rather than to actively avoid them, and the importance of values. For example:

"*At least the main bit that I got from it was to first just observe some of the negative feelings that you have, and not necessarily like reject and wrestle with them, just to sit and watch them and observe them, and accept them [...]. You can perhaps do something like remind yourself of values. I like that one too." -P05*

The participant clearly reflects that the game encouraged them to observe and accept their thoughts and feelings rather than suppress (or "wrestle") them. They also mention that the game reminded them of what really matters to them.

Participants appreciated the leaves of the stream exercise as promoting acceptance of difficult thoughts, for example:

For peer review only - http://bmjopen.bmj.com/site/about/guidelines.xhtml

"I'd like the Lily pad thing. I think it's really nice because I myself struggle with my emotions or bad thoughts or whatever, and I stay on them, and I make myself feel guilty about situations. So, it was really nice to accept letting go of yourself." -P06

Other participants reflected on the game encouraging acceptance and observation of difficult thoughts, for example:

"I think that section when it's like acceptance and realising that you know, even though you may not like these thoughts you're having, you still need to be aware of them and you can use them as a springboard so that was my favourite section." -P18

Theme 4: Embedded learning pros and cons

Data from the immediate post-intervention interviews suggest a mixed response to embedded learning in 'ACTing Minds'. Many participants reported learning a variety of lessons through playing the game, most being intended in the game's design. Participants were sometimes confused with the lack of explicit objectives but were still able to understand that there were consequences to avoidant behaviour, and that acceptance of difficult emotions was rewarded. This indicated the embedded learning within the game dynamics were successfully implemented. For example:

"So when you hit them [memories], the like walls go up right, yeah? […] But I guess it was like kind of showing you that if you hit the memories, you kind of close yourself off and that's what the walls were." -P14

Another participant also suggested that they were able to learn relevant ACT concepts quickly such as not to avoid (suppress) thoughts, for example:

For peer review only - http://bmjopen.bmj.com/site/about/guidelines.xhtml

"I think the fact that I think it was very quickly aware that you shouldn't just destroy memories and I think that was that was done really well. Maybe too well. As in like it went on a bit."

Theme 5: Necessary learning for anyone

Several participants expressed that they felt what they learned via ACTing Minds would be applicable and useful to many others in society, and not just for themselves. For example:

"I think it's a good thing that more people are learning about this kind of thing and. It kind of just leads into more research regarding and. More support out there and more help. It's generally like, I think. Maybe not my thing, but. It's not like a bad thing, it's. A good thing there's people doing it." P27.

"I think like the impact did still stick with me, like I still mentioned it quite a bit to like my parents afterwards and I mentioned it to my partner as well. They seemed interested and they like wished they could have done that too."-P18.

Theme 6 (3-week follow-up interview theme): Utility in the real world

In the interview occurring 3-weeks postintervention, participants discussed how they were able to apply the lessons that they took from 'ACTing Minds' into the real-world. Whilst a few participants had not considered the game since playing or tried to actively apply lessons, many found that they made a conscious effort to apply ACT principles and techniques from the game into their lives. Some example quotes and descriptions are given below:

"I have noticed I've actively now, if I get like a negative thought in my brain. I try and register it and I don't hold on. But like, because sometimes before, even though I kind of subconsciously did it with some things, I just didn't [always], you know. But now if it's even something stupid like I've been lazy. I was like alright don't think about it all day. Actually go

For peer review only - http://bmjopen.bmj.com/site/about/guidelines.xhtml

out for a walk. Go to the gym you know. Don't just keep in your head, I'm so lazy and miserable and fat. Get out to do something about it." -P04

This statement was given by a participant who in the prior immediate-postintervention interview felt that they did not have a problem with holding on to difficult thoughts. Despite this, in the weeks since playing 'ACTing Minds', the participant found that they were more conscious of how their thoughts have impacted their real life, and as a result, have been able to apply the core ACT concepts in tandem. Their statement indicates that they were able to apply defusion lessons to difficult thoughts about their self ("I'm so lazy and miserable and fat"), acceptance of their behaviours ("I've been lazy"), and commitment to values by taking part in ACT-oriented activities regardless of their thoughts. Many participants noted that even if they had not explicitly tried to apply the ACT principles learned from the game to real life, that they felt the game had influenced them subconsciously:

"I wouldn't necessarily say I've sat there and primed those thoughts [regarding ACT concepts], but in unconscious thinking, if you know like passive thought and stuff like that in my day-to-day, I've definitely had hints of some of those topics. Do you know what I mean? Like even today, I was going about my day doing my thing and you know you'll have a thought that'll throw you back to the past, and then you learn like I came to accept it." -P11

This participant found that the ACT principles were more readily available to them when confronted with real-life situations that demanded them. In these cases, participants most commonly felt that they were more accepting of difficult emotions and situations.

Theme 7 (3-week follow-up interview theme): Practice facilitates psychological flexibility skills

A core theme present across follow-up interviews was that participants expressed how applying what they had learned into their day-to-day activities over three weeks led to an

For peer review only - http://bmjopen.bmj.com/site/about/guidelines.xhtml

even deeper understanding of the ACT concepts they learned. Participants reported greater engagement with values-orientated behaviours in their everyday real lives, and a greater ability to cognitively defuse (or let go) of difficult thoughts rather than engaging in avoidant behaviour. Some example quotes and descriptions are given below:

“Well, it's like actually testing on a real situation as to just generally learning it. But then when you come into a situation, you start to understand a bit better why you do those things [referring to ACT skills] and what benefit those things have, because the situation is actually impacting on your emotions or your feelings and stuff like that, and so then you're like oh, this is why this is a good technique.” -P05

In practising real-life application of ACT principles, participants were able to get a deeper sense of how and why the ACT techniques worked for them, especially during emotionally challenging situations. Through such practice, participants have noted that their personal values have become clearer to them:

“I've learned ways to engage with my thoughts, and like I've always tried to practice letting go of things that aren't like too meaningful, like things that won't matter in a day and all that sort of stuff. But I feel like the game has helped me also realise […] a way to really put my values down in a more straightforward manner.” -P13

The participant refers to the real life practising of letting go of difficult thoughts (cognitive defusion), and values identification.

Theme 8 (3-week follow-up interview theme): Closer alignment to an integrated self (as context), with acceptance, values, as part of who you are

One of the most consistent patterns across interview responses was that participants felt they had learned more about themselves through acceptance, or that practicing the lessons taught in the game helped them align to their values. Some participants seem to have expressed that

For peer review only - http://bmjopen.bmj.com/site/about/guidelines.xhtml

they learned the ACT-concepts in a more integrated way, where they felt that acceptance of difficult thoughts and their values was part of who they were i.e., self as context. Some example quotes and descriptions are given below:

"I actually learned a lot from this game about like these inner emotions or bad memories are not sinful. They're a part of you, and they contribute what you're going to be or the current you [self]. So yeah, that's the core lessons I guess I learned from the game." -P23

The participant reflects here about accepting their personal experiences in the present moment for what they are (indicating broader integrated acknowledgements about themselves in context). For other participants, this acceptance of personal experiences in day-to-day life has facilitated further identification of personal values:

"Yeah, as I said, the values that's definitely helped me. Learning to just calm my thoughts for a little and think of the small but important things in life and what I appreciate that helps. It's also made me learn more about how to deal with grief [...] It's helped to learn that it's OK to feel grief, it's a part of who I am, and I must accept it -P08

Secondary outcome measures

Quantitative results (all data is available on the Open Science Framework: https://osf.io/3wuh5/) (38) revealed a large effect size for the EQ5D usual activities score ($\eta p2 = .307$), while medium effect sizes were found for DASS-21 Stress scores ($\eta p2 = .108$), DASS-21 Anxiety scores ($\eta p2 = .096$), and AAQ-II Psychological Flexibility scores ($\eta p2 = .060$). Small effect sizes were obtained for the DASS-21 Depression scores ($\eta p2 = .011$), the WEMWBS ($\eta p2 =.011$), UCLA Social Connectedness Scale ($\eta p2 =.021$), EQ5D Pain/Discomfort ($\eta p2 =.010$), and the EQ5D Anxiety/Depression ($\eta p2 = .018$). Given this is a feasibility study that is intentionally underpowered as it has a small sample size, *p* value significance is statistically meaningless for measuring the efficacy of any given measure.

For peer review only - http://bmjopen.bmj.com/site/about/guidelines.xhtml

Instead, the effect sizes allow for a G-Power (39) a-priori analysis to be conducted that indicates the sample size required to detect meaningful statistical between-group differences within a future full-scale RCT. For this, the G-Power (version 3.1.9.7) indicated that when assuming a between factor (two group), repeated measures (three points in time) design with an alpha error probability of 0.05, and acceptable power of 0.8 (40), then 436 participants are required to detect a meaningful statistical difference for the smallest measure effect size (DASS-21 depression) in a future RCT. See Table 2 for full details including effect sizes, power, and estimated sample sizes for a future RCT given the observed effects sizes of this feasibility study.

Convergent outcomes

Integration of results are considered a defining feature of mixed-methods research (41). In the interest of transparency, we have included a visualisation of the research outcomes taken from our qualitative and quantitative approaches, illustrating how each outcome links back to feasibility, as well as the conclusions made following each outcome (see Figure 3).

For peer review only

For peer review only - http://bmjopen.bmj.com/site/about/guidelines.xhtml

Table 2.
Illustrating change in intervention outcomes over time (n=29)

	**Pre-intervention Baseline, mean (SD)**	**Post-intervention, mean (SD)**	**Follow-up (3 weeks), mean (SD)**	**ηp2**	**Effect size**	***F***	**Power**	**Full RCT Sample size estimated assuming 0.8 power**
DASS-21 Depression	14.34 (5.97)	14.21 (5.54)	13.90 (4.75)	.011	Small	.31	0.43	436
DASS-21 Stress	15.34 (4.55)	14.52 (4.40)	14.28 (4.65)	.108	Medium	3.39	0.999	46
DASS-21 Anxiety	12.79 (5.19)	12.10 (4.42)	11.66 (4.75)	.096	Medium	2.98	0.999	52
AAQ-II (Psychological Flexibility)	27.34 (10.47)	28.38 (9.86)	28.86 (8.65)	.060	Medium	1.76	0.999	86
WEMWBS	42.07 (7.31)	42.62 (7.91)	42.07 (7.00)	.011	Small	.31	0.33	436
UCLA Social-Connectedness	64.72 (7.52)	64.24 (8.85)	63.90 (8.33)	.021	Small	.60	0.77	236
EQ5D Mobility	4.83 (.38)	4.83 (.38)	4.83 (.38)	.000	N/A	.00	0.05	Negligible (no effect)
EQ5D Self-Care	4.78 (.58)	4.79 (.49)	4.76 (.51)	.000	N/A	.00	0.05	Negligible (no effect)
EQ5D Usual Activities	4.14 (.79)	4.38 (.68)	4.62 (.68)	.307	Large	12.42	1.00	16
EQ5D Pain/Discomfort	4.24 (.64)	4.45 (.63)	4.31 (.81)	.010	Small	.28	0.25	520
EQ5D Anxiety/ Depression	3.86 (.99)	4.03 (.87)	3.97 (.94)	.018	Small	.52	0.66	288
EQ5D Self-Rated Health Score	71.14 (19.01)	72.76 (19.57)	70.93 (20.71)	.000	N/A	.00	0.05	Negligible (no effect)

---Figure 3 Here---

Discussion

Statement of principal findings

For peer review only - http://bmjopen.bmj.com/site/about/guidelines.xhtml

The overarching aim of this research was to test the feasibility and acceptability of the 'ACTing Minds' video game as a DHI for treating depression, anxiety, and stress. Participant recruitment and retention, as well as quantitative and qualitative results, demonstrate that the study design and videogame intervention are feasible for testing in a full-scale RCT. Thematic analysis on qualitative data revealed several key findings. Firstly, participants were successfully able to learn about core ACT principles through embedded learning, specifically acceptance, defusion from thoughts, and commitment to personal values. There was also some indication that participants had learned something about themselves in an integrated way, in the form of self-as-context. Participants felt that the lessons taught within the game could be applied to their daily lives and that the game was effective in priming them to consider the core ACT principles throughout the weeks following their completion of the intervention. Participants also felt that they would recommend the game to someone that they care about and that they would be interested in downloading and completing future releases of 'ACTing Minds'.

Summary of secondary outcomes

Quantitative analysis of measures taken at baseline, immediately post-intervention, and after a 3-week follow-up, revealed promising small to large effect sizes in many of the quantitative measures (38). This included a large effect size for increasing EQ5D usual activities, medium effect sizes for reducing DASS-21 stress and anxiety, as well as increased AAQ-II psychological flexibility. Small effect sizes were observed for reducing DASS-21 depression, EQ5D pain and discomfort, EQ5D anxiety and depression, and increased UCLA social connectedness and WEMWBS general wellbeing. There were no observed effects for EQ5D mobility, self-care, and self-rated health score. Given this is a feasibility study that is intentionally underpowered as it has a small sample size, *p* value significance is statistically meaningless for measuring the efficacy of the interventions, instead effect sizes are more

For peer review only - http://bmjopen.bmj.com/site/about/guidelines.xhtml

informative as they express the underlying effect and are not influenced by population size. Instead, the G-Power analysis provided an estimation of participants required (436 to account for the small effect sizes) to identify meaningful significance in a full scale future RCT.

Comparison to existing literature

This is the first study to utilise a videogame Digital Health Intervention (DHI) rooted in third-wave behavioural therapy to address mental distress. 'ACTing Minds' imparts psychological skills based on ACT to promote psychological flexibility. Prior research has primarily focused on videogame DHIs targeting ‘illbeing’ that aim to reduce symptoms of mental illness, two examples are games 'REThink' and 'Dojo'. 'REThink,' designed for a younger audience, developed the players’ ability to discern functional emotions from maladaptive ones, and was shown to effectively improve emotional symptoms and reduce depressive mood (42). 'Dojo,' utilising biofeedback and relaxation techniques to promote emotional regulation, was shown to significantly decrease participants’ anxiety and aggressive behaviour scores post-intervention but exhibited no long-term effects at a 4-month follow-up (43). Our DHI differs fundamentally from these other games due to strong theoretical underpinnings based on ACT for promoting psychological flexibility instead of a focus on reducing unwanted emotions or emotional regulation. Participants within the game were taught to observe and be open to emotional pain without judgement or any attempt to change them, and this is a key focus within the ‘ACTing Minds’ intervention.

Several meta-analyses have been conducted to explore the potential efficacy of DHIs, one found that the majority of DHIs were based on Cognitive Behavioural Therapy (CBT) and that the effect size for such interventions was small for reducing depressive symptoms compared to non-treatment controls (44). CBT-based DHIs usually do not take the form of full video games but may include elements of gamification such as rewards, badges, and progress tracking. They are typically structured programs including online education tools,

For peer review only - http://bmjopen.bmj.com/site/about/guidelines.xhtml

interactive exercises, and self-assessment tools, which focus on challenging and modifying negative thoughts and behaviours. A meta-analysis including 34 RCTs (17 of which were CBT-based) found that CBT-based DHIs yielded a medium effect size for reducing symptoms of depression and anxiety (45). However, a meta-analysis of 117 CBT-based applications revealed that only 12 of them provided support aligned with the evidence-based tenets of CBT (46). This finding suggests that the observed effectiveness in earlier studies could potentially stem from participants' interaction with a DHI (perhaps as a form of distraction) rather than their proficient implementation of CBT principles. CBT-DHI programs often require consistent use, and high attrition rates have limited their efficacy in research (47). One meta-analysis author suggested that DHIs may need to be complemented by existing mental health support (44). However, our study challenges this notion. In less than one hour of playing 'ACTing Minds', participants fully explored the game, retained ACT knowledge, and discussed its positive real-world application in interviews conducted 3 weeks post-intervention.

Strengths and limitations

Our study is the first to explore the feasibility and acceptability of a novel videogame DHI based on ACT. A core strength of this research was the utilisation of a mixed-methods approach. By incorporating thematic analysis of semi-structured interviews as well as quantitative analysis of questionnaire data, we were able to gain a comprehensive understanding of participant experience using 'ACTing Minds'. Collecting quantitative data at three separate time points, and interviews conducted at two separate time points meant that we were able to examine the gradual processes of change and identify patterns of improvement consistent with the ACT model. We gained valuable input from participants in terms of suggestions for improving the intervention which will aid in the further development of 'ACTing Minds' to optimise effectiveness and user engagement. The results from the

For peer review only - http://bmjopen.bmj.com/site/about/guidelines.xhtml

interviews also indicated that ‘ACTing Minds’ has broad appeal as a videogame even to those outside of clinical populations.

It is also important to acknowledge the limitations of our study. Firstly, there was no control group, though this is intentional as this is a feasibility study, it is only when we conduct a full RCT that we will have an adequate comparator group to determine whether the intervention is clinically useful at promoting psychological flexibility and reducing depression, anxiety, and stress. There was a lack of in-game data logging of specific ACT tasks completed. For example, we did not collect data on whether participants actually entered text about the difficult thoughts that they were experiencing such as in the leaves on a steam exercise, though all participants completed the game. The reliance on self-report measures potentially allowed for biased responses, including psychosociological measures such as HRV would strengthen the study outcomes. It is also possible that some of the questionnaires used in this study were insufficient for capturing the target measurements. One study used exploratory factor analysis to investigate the extent to which the AAQ-II Psychological Flexibility Questionnaire can discriminate between experiential avoidance and psychological flexibility. The researchers found that AAQ-II items were more strongly related to items measuring distress than items measuring acceptance (48). In line with this, the AAQ-II has been criticised as being too simple a measure for psychological flexibility (49). In a future RCT, we may adopt another measure for measuring psychological flexibility such as The Personalised Psychological Flexibility Index which may be a more valid measure of psychological flexibility (50). Finally, though thematic analysis can be highly useful for identifying shared meaning and variation amongst the themes, and bridging subjectivity and theoretical structure, it also has limitations. The contextualist epistemology used in this approach acknowledges the researcher has an active role in shaping the outcomes and can be biased by their own knowledge and experience. The subjective nature of the themes means

For peer review only

For peer review only - http://bmjopen.bmj.com/site/about/guidelines.xhtml

that there can be variation in the interpretation of the data between different thematic researchers. So, though this approach can be useful, these limitations also need to be acknowledged.

Clinical implications and directions for future research

The ACT-based videogame DHI used in this study is a low-cost, engaging, and easy-to-disseminate means of supporting those experiencing mental health difficulties. The present study highlights the clinical implications of 'ACTing Minds', including its potential therapeutic value, user engagement, and accessibility. However, further research is warranted to establish long-term effects, explore specific populations, conduct comparative studies, investigate underlying processes, and address any ethical considerations that may arise. Critically, a full RCT is now needed, in which participants are compared quantitatively with a control group, incorporating physiological wellbeing measures such as HRV, as well as research-validated questionnaires regarding mental health (i.e., depression, stress, anxiety, psychological flexibility, social connectedness, and wellbeing). By pursuing these future research directions, we can leverage the potential of ACT-based video games such as 'ACTing Minds' to enhance patient care, improve outcomes, and expand the reach of interventions in an increasingly digital era.

Conclusion

The results of this study demonstrate that 'ACTing Minds' is feasible to implement in a full-scale RCT. Both the intervention and study were well received by participants, thematic analysis of semi-structured interviews indicated that a single playthrough of the game was sufficient for teaching several core principles of ACT, namely 'Acceptance', 'Values Identification', and 'Cognitive Defusion' to participants, and priming them to implement the lessons in their day-to-day lives. There was some evidence that participants also integrated

For peer review only - http://bmjopen.bmj.com/site/about/guidelines.xhtml

their learning about themselves as self-as context, which is interesting. Quantitative results indicate that playing 'ACTing Minds' is associated with decreases in depression, anxiety, and stress, as well as increases in psychological flexibility, social connectedness, and wellbeing. However, these effects will need to be further explored in an adequately powered RCT to understand the potential clinical implications, therapeutic value, user engagement, and accessibility of an ACT-based video game DHI.

Data availability statement

The datasets supporting the conclusions of this article are available in the Open Science Framework (38): https://osf.io/3wuh5/?view_only=7998f1d0ae4b473ab7089a71be710270.

Original published protocol

https://bmjopen.bmj.com/content/10/11/e041667

Contribution statement

DJE and AHK designed the original protocol, whilst TCG updated and revised the protocol design. TCG wrote the first draft of the paper and conducted all of the quantitative and qualitative results. DJE and AHK provided substantial revisions on all drafts and advised TCG throughout the development of this manuscript. DJE designed and developed the game development.

Competing interests

The game ACTing Minds was developed using European development funds via the commercial entity of Swansea University (AgorIP) and awarded to DJE with the intention to develop this game for commercial purposes (as a game app for the Apple and Google Play stores). DJE was involved in the design of the protocol but did not recruit participants, collect any data, and did not conduct the analysis on the data. TCG and AHK have no involvement in any commercial aspects of the game.

Funding

For peer review only - http://bmjopen.bmj.com/site/about/guidelines.xhtml

Funding for the development of this game came from European development funds via the commercial entity of Swansea University called AgorIP (Reference: 229-0256-0046) awarded to DJE.

Acknowledgements

We would like to thank Miricle Tea Studios Ltd and Mikoshi Ltd for the production and release of the ACTing Minds videogame. We would also like to thank Prof. Louise McHugh (University College Dublin), Dr. Alison Stapleton (Dublin Business School), and Dr. Sarah Cassidy (Smithfield Clinic) for several helpful comments on various aspects of the ACTing Minds intervention and methodology.

Ethics statement

This study has received ethical approval from the Swansea University School of Psychology ethics sub-committee (2022-5630-4834).

Figure legends

Figure 1.

(a) Screenshot from 'ACTing Minds' showing the 'Psychoflexameter'. (b) The Acceptance and Commitment Therapy Hexaflex and core processes. (c) Screenshot from 'ACTing Minds', example of 'Cognitive defusion' task. The player is required to type in their own difficult thoughts, before dragging them on to a leaf that floats downstream.

Figure 2.
Flow of participants through the study.

Figure 3.
Diagram visualising the integrated outcomes of 'ACTing Minds' feasibility and acceptability study.

References

1. Fineberg NA, Haddad PM, Carpenter L, Gannon B, Sharpe R, Young AH, et al. The size, burden and cost of disorders of the brain in the UK. Journal of Psychopharmacology. 2013;27(9):761-70.

For peer review only

2. GBoDC N. Global Burden of Disease Study 2017 Results. Seattle, United States: Global Burden of Disease Collaborative Network. 2018.

3. Dettmann LM, Adams S, Taylor G. Investigating the prevalence of anxiety and depression during the first COVID-19 lockdown in the United Kingdom: Systematic review and meta-analyses. British Journal of Clinical Psychology. 2022;61(3):757-80.

4. Flynn D, Moloney E, Bhattarai N, Scott J, Breckons M, Avery L, et al. COVID-19 pandemic in the United Kingdom. Health Policy and Technology. 2020;9(4):673-91.

5. McManus S, Bebbington PE, Jenkins R, Brugha T. Mental health and wellbeing in England: the adult psychiatric morbidity survey 2014: NHS digital; 2016.

6. Salaheddin K, Mason B. Identifying barriers to mental health help-seeking among young adults in the UK: a cross-sectional survey. British Journal of General Practice. 2016;66(651):e686-e92.

7. Serafino P. Exploring the UK's digital divide. Office for National Statistics. 2019.

8. Lemenager T, Neissner M, Koopmann A, Reinhard I, Georgiadou E, Müller A, et al. COVID-19 lockdown restrictions and online media consumption in Germany. International journal of environmental research and public health. 2021;18(1):14.

9. Talevi D, Socci V, Carai M, Carnaghi G, Faleri S, Trebbi E, et al. Mental health outcomes of the CoViD-19 pandemic. Rivista di psichiatria. 2020;55(3):137-44.

10. Murray E, Hekler EB, Andersson G, Collins LM, Doherty A, Hollis C, et al. Evaluating digital health interventions: key questions and approaches. Elsevier; 2016. p. 843-51.

11. Linehan C, Kirman B, Roche B, editors. Gamification as behavioral psychology. The gameful world: Approaches, issues, applications; 2015: MIT Press.

For peer review only

12. Ofcom. Gaming penetration in the United Kingdom (UK) from 2013 to 2022, by age group and gender 2023 [cited 2023 June 19]. Available from: https://www.statista.com/statistics/300513/gaming-by-demographic-group-uk/.

13. Kuosmanen T, Fleming T, Newell J, Barry M. A pilot evaluation of the SPARX-R gaming intervention for preventing depression and improving wellbeing among adolescents in alternative education. Internet Interventions. 2017;8:40-7.

14. Rusch DC, editor " Elude" designing depression. Proceedings of the International Conference on the Foundations of Digital Games; 2012.

15. Scholten H, Malmberg M, Lobel A, Engels RC, Granic I. A randomized controlled trial to test the effectiveness of an immersive 3D video game for anxiety prevention among adolescents. PloS one. 2016;11(1):e0147763.

16. Edwards DJ, Kemp AH. A novel ACT-based video game to support mental health through embedded learning: a mixed-methods feasibility study protocol. BMJ open. 2020;10(11):e041667.

17. Barr M, Copeland-Stewart A. Playing video games during the COVID-19 pandemic and effects on players' well-being. Games and Culture. 2022;17(1):122-39.

18. Hayes S, Strosahl K, Wilson K. Acceptance and commitment therapy: American Psychological Association. Washington, DC[Google Scholar]. 2009.

19. Hayes SC, Hofmann SG. The third wave of cognitive behavioral therapy and the rise of process-based care. World psychiatry. 2017;16(3):245.

20. Hayes SC, Strosahl KD, Wilson KG. Acceptance and commitment therapy: The process and practice of mindful change: Guilford press; 2011.

21. Hayes S. A Liberated Mind: The essential guide to ACT: Random House; 2019.

For peer review only

22. Gloster AT, Walder N, Levin ME, Twohig MP, Karekla M. The empirical status of acceptance and commitment therapy: A review of meta-analyses. Journal of Contextual Behavioral Science. 2020;18:181-92.

23. Dindo L, Van Liew JR, Arch JJ. Acceptance and commitment therapy: a transdiagnostic behavioral intervention for mental health and medical conditions. Neurotherapeutics. 2017;14:546-53.

24. Mattila E, Lappalainen R, Välkkynen P, Sairanen E, Lappalainen P, Karhunen L, et al. Usage and dose response of a mobile acceptance and commitment therapy app: secondary analysis of the intervention arm of a randomized controlled trial. JMIR mHealth and uHealth. 2016;4(3):e5241.

25. Singh S, Starkey NJ, Sargisson RJ. Using SmartQuit®, an acceptance and commitment therapy smartphone application, to reduce smoking intake. Digital health. 2017;3:2055207617729535.

26. Craig P, Dieppe P, Macintyre S, Michie S, Nazareth I, Petticrew M. Developing and evaluating complex interventions: the new Medical Research Council guidance. Bmj. 2008;337.

27. Billingham SA, Whitehead AL, Julious SA. An audit of sample sizes for pilot and feasibility trials being undertaken in the United Kingdom registered in the United Kingdom Clinical Research Network database. BMC medical research methodology. 2013;13:1-6.

28. Eldridge SM, Chan CL, Campbell MJ, Bond CM, Hopewell S, Thabane L, et al. CONSORT 2010 statement: extension to randomised pilot and feasibility trials. bmj. 2016;355.

29. Henry JD, Crawford JR. The short-form version of the Depression Anxiety Stress Scales (DASS-21): Construct validity and normative data in a large non-clinical sample. British journal of clinical psychology. 2005;44(2):227-39.

For peer review only

For peer review only - http://bmjopen.bmj.com/site/about/guidelines.xhtml

30. Bond FW, Hayes SC, Baer RA, Carpenter KM, Guenole N, Orcutt HK, et al. Preliminary psychometric properties of the Acceptance and Action Questionnaire–II: A revised measure of psychological inflexibility and experiential avoidance. Behavior therapy. 2011;42(4):676-88.

31. Russell DW. UCLA Loneliness Scale (Version 3): Reliability, validity, and factor structure. Journal of personality assessment. 1996;66(1):20-40.

32. Kok BE, Coffey KA, Cohn MA, Catalino LI, Vacharkulksemsuk T, Algoe SB, et al. How positive emotions build physical health: Perceived positive social connections account for the upward spiral between positive emotions and vagal tone. Psychological science. 2013;24(7):1123-32.

33. Tennant R, Hiller L, Fishwick R, Platt S, Joseph S, Weich S, et al. The Warwick-Edinburgh mental well-being scale (WEMWBS): development and UK validation. Health and Quality of life Outcomes. 2007;5(1):1-13.

34. Balestroni G, Bertolotti G. EuroQol-5D (EQ-5D): an instrument for measuring quality of life. Monaldi Archives for Chest Disease. 2012;78(3).

35. McHugh L. A new approach in psychotherapy: ACT (acceptance and commitment therapy). The World Journal of Biological Psychiatry. 2011;12(sup1):76-9.

36. Clarke V, Braun V. Thematic analysis: a practical guide. Thematic Analysis. 2021:1-100.

37. Grissom RJ, Kim JJ. Effect sizes for research: Univariate and multivariate applications: Routledge; 2012.

[dataset] 38. Gordon TC, Kemp AH, Edwards DJ. Data from: Mixed-methods feasibility outcomes for a novel ACT-based videogame 'ACTing Minds' to support mental health. Open Science Framework, February 16, 2024. https://doi.org/10.17605/OSF.IO/3WUH5

For peer review only

39. Faul F, Erdfelder E, Lang A-G, Buchner A. G* Power 3: A flexible statistical power analysis program for the social, behavioral, and biomedical sciences. Behavior research methods. 2007;39(2):175-91.

40. Cohen J. Statistical power analysis for the behavioral sciences: Academic press; 2013.

41. McCrudden MT, Marchand G, Schutz PA. Joint displays for mixed methods research in psychology. Methods in Psychology. 2021;5:100067.

42. David OA, Cardoș RA, Matu S. Is RET hink therapeutic game effective in preventing emotional disorders in children and adolescents? Outcomes of a randomized clinical trial. European child & adolescent psychiatry. 2019;28:111-22.

43. Schuurmans AA, Nijhof KS, Engels RC, Granic I. Using a videogame intervention to reduce anxiety and externalizing problems among youths in residential care: an initial randomized controlled trial. Journal of psychopathology and behavioral assessment. 2018;40:344-54.

44. Garrido S, Millington C, Cheers D, Boydell K, Schubert E, Meade T, et al. What works and what doesn't work? A systematic review of digital mental health interventions for depression and anxiety in young people. Frontiers in psychiatry. 2019;10:759.

45. Grist R, Croker A, Denne M, Stallard P. Technology delivered interventions for depression and anxiety in children and adolescents: a systematic review and meta-analysis. Clinical Child and Family Psychology Review. 2019;22:147-71.

46. Huguet A, Rao S, McGrath PJ, Wozney L, Wheaton M, Conrod J, et al. A systematic review of cognitive behavioral therapy and behavioral activation apps for depression. PloS one. 2016;11(5):e0154248.

47. Venkatesan A, Rahimi L, Kaur M, Mosunic C. Digital cognitive behavior therapy intervention for depression and anxiety: retrospective study. JMIR Mental Health. 2020;7(8):e21304.

For peer review only

48. Wolgast M. What does the Acceptance and Action Questionnaire (AAQ-II) really measure? Behavior therapy. 2014;45(6):831-9.

49. Doorley JD, Goodman FR, Kelso KC, Kashdan TB. Psychological flexibility: What we know, what we do not know, and what we think we know. Social and Personality Psychology Compass. 2020;14(12):1-11.

50. Cherry KM, Vander Hoeven E, Patterson TS, Lumley MN. Defining and measuring "psychological flexibility": A narrative scoping review of diverse flexibility and rigidity constructs and perspectives. Clinical psychology review. 2021;84:101973.

For peer review only

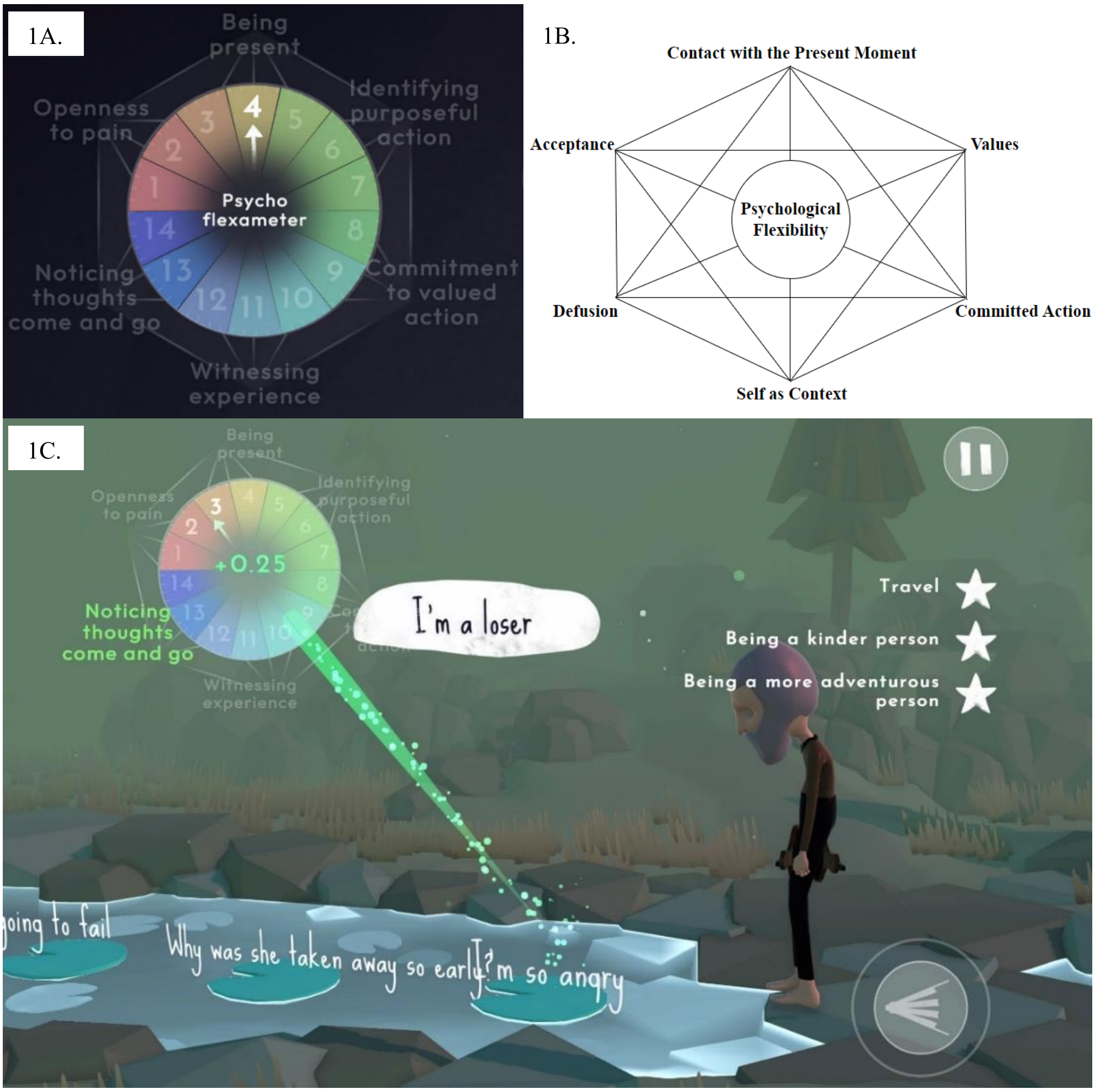
1A.
Being present
Identifying purposeful action
Openness to pain
Psycho flexameter
Noticing thoughts come and go
Commitment to valued action
Witnessing experience
1B.
Contact with the Present Moment
Acceptance
Values
Psychological Flexibility
Defusion
Committed Action
Self as Context
1C.
Being present
Openness to pain
Identifying purposeful action
+0.25
Noticing thoughts come and go
Witnessing experience
I'm a loser
Travel
Being a kinder person
Being a more adventurous person
Why was she taken away so early
I'm so angry

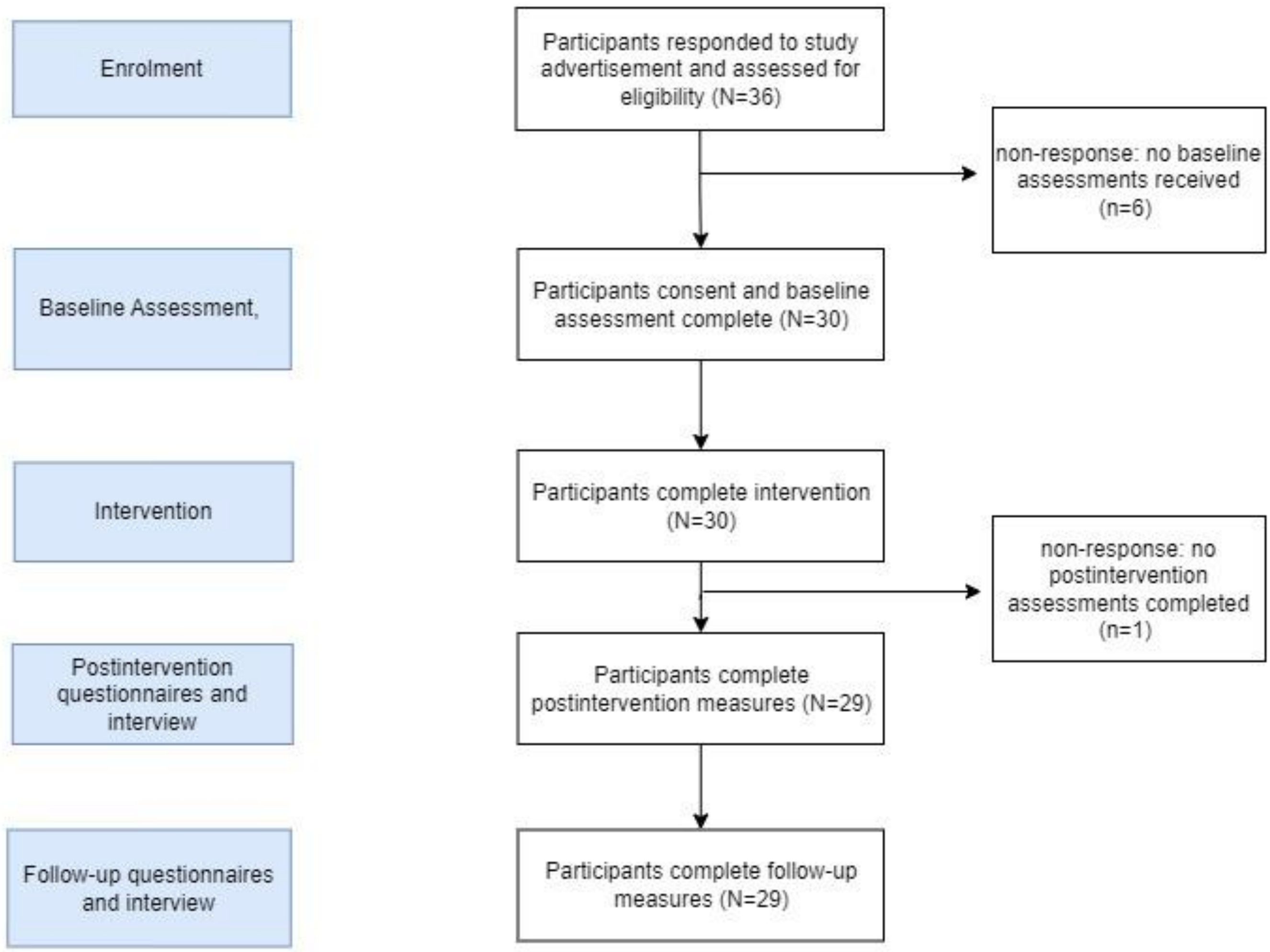
Enrolment
Participants responded to study advertisement and assessed for eligibility (N=36)
non-response: no baseline assessments received (n=6)
Baseline Assessment,
Participants consent and baseline assessment complete (N=30)
Intervention
Participants complete intervention (N=30)
non-response: no postintervention assessments completed (n=1)
Postintervention questionnaires and interview
Participants complete postintervention measures (N=29)
Follow-up questionnaires and interview
Participants complete follow-up measures (N=29)

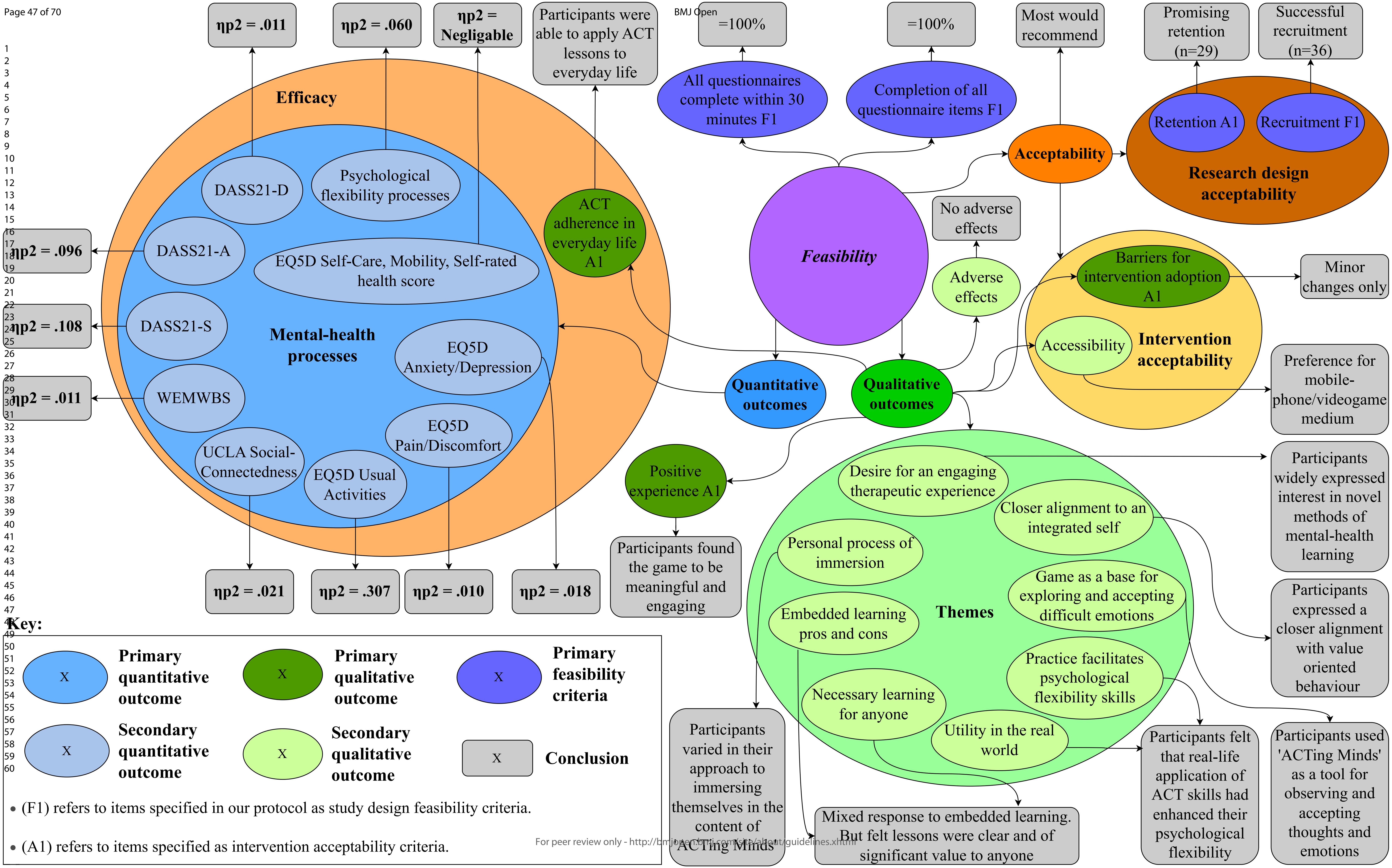

ηp2 = .011
ηp2 = .060
ηp2 = Negligable
Participants were able to apply ACT lessons to everyday life
=100%
=100%
Most would recommend
Promising retention (n=29)
Successful recruitment (n=36)
Efficacy
DASS21-D
Psychological flexibility processes
ACT adherence in everyday life A1
All questionnaires complete within 30 minutes F1
Completion of all questionnaire items F1
Acceptability
Retention A1
Recruitment F1
Research design acceptability
ηp2 = .096
DASS21-A
EQ5D Self-Care, Mobility, Self-rated health score
Feasibility
No adverse effects
Adverse effects
Barriers for intervention adoption A1
Minor changes only
ηp2 = .108
DASS21-S
Mental-health processes
EQ5D Anxiety/Depression
Quantitative outcomes
Qualitative outcomes
Accessibility
Intervention acceptability
Preference for mobile-phone/videogame medium
ηp2 = .011
WEMWBS
EQ5D Pain/Discomfort
UCLA Social-Connectedness
EQ5D Usual Activities
Positive experience A1
Participants widely expressed interest in novel methods of mental-health learning
Desire for an engaging therapeutic experience
Closer alignment to an integrated self
Personal process of immersion
Participants found the game to be meaningful and engaging
ηp2 = .021
ηp2 = .307
ηp2 = .010
ηp2 = .018
Game as a base for exploring and accepting difficult emotions
Embedded learning pros and cons
Themes
Participants expressed a closer alignment with value oriented behaviour
Key:
X
Primary quantitative outcome
X
Primary qualitative outcome
X
Primary feasibility criteria
Practice facilitates psychological flexibility skills
Necessary learning for anyone
Utility in the real world
X
Secondary quantitative outcome
X
Secondary qualitative outcome
X
Conclusion
Participants varied in their approach to immersing themselves in the content of ACTing Minds
Participants felt that real-life application of ACT skills had enhanced their psychological flexibility
Participants used 'ACTing Minds' as a tool for observing and accepting thoughts and emotions
• (F1) refers to items specified in our protocol as study design feasibility criteria.
• (A1) refers to items specified as intervention acceptability criteria.
Mixed response to embedded learning. But felt lessons were clear and of significant value to anyone

BMJ Open

A novel ACT-based video game to support mental health through embedded learning: a mixed-methods feasibility study protocol

Darren J Edwards ,[1] Andrew H Kemp[2]

To cite: Edwards DJ, Kemp AH. A novel ACT-based video game to support mental health through embedded learning: a mixed-methods feasibility study protocol. *BMJ Open* 2020;**10**:e041667. doi:10.1136/bmjopen-2020-041667

► Prepublication history and additional material for this paper is available online. To view these files, please visit the journal online (http://dx.doi.org/10.1136/bmjopen-2020-041667).

Received 15 June 2020
Revised 06 October 2020
Accepted 30 October 2020

Check for updates

© Author(s) (or their employer(s)) 2020. Re-use permitted under CC BY-NC. No commercial re-use. See rights and permissions. Published by BMJ.

[1]Department of Public Health, Policy, and Social Sciences, Swansea University, Swansea, UK
[2]Department of Psychology, Swansea University, Swansea, UK

Correspondence to
Dr Darren J Edwards; D.J.Edwards@swansea.ac.uk

ABSTRACT

Introduction In recent years, serious video games have been used to promote emotional regulation in individuals with mental health issues. Although these therapeutic strategies are innovative, they are limited with respect to scope of treatment, often focusing on specific cognitive skills, to help remediate a specific mental health disorder.
Objective Here, we propose a protocol for assessing the feasibility of a novel acceptance and commitment therapy (ACT)-based video game for young adults.
Methods and analysis The Medical Research Council (MRC) framework will be used for developing a complex intervention to design and test the feasibility of an ACT-based video game intervention using a mixed-methods approach involving qualitative and quantitative data. The primary outcomes will include feasibility testing of recruitment processes and the acceptability of the intervention through qualitative interviews, attendance and rates of attrition. Secondary outcomes will involve a series of quantitative questionnaires to obtain effect sizes for power analysis, allowing for the ideal sample size for an appropriately powered, randomised controlled trial to be determined.
Ethics and dissemination This study has been approved by the Psychology Department Research Ethics Committee (2020-4929-3923) at Swansea University in the UK. Dissemination activities will involve publications in peer-reviewed journals, presentations at local and national conferences and promotion through social media.
Trial registration number NCT04566042.

Strengths and limitations of this study

- ► Mixed-methods approach to build a rich dataset on which conclusions will be drawn.
- ► Protocol follows established Medical Research Council (MRC) guidelines.
- ► In line with MRC guidelines and stage of game development, randomisation is not a component of this study.
- ► Aims are to assess feasibility, an important step in the development of complex interventions, although limiting conclusions able to be drawn.

INTRODUCTION

Mental health issues such as anxiety and depression are a global problem of increasing concern, imposing considerable burden on society. The Global Burden of Disease project[1] has identified mental health disorders as a leading cause of disability globally, and suggest that there are 266 million cases of anxiety, and 253 million cases of major depressive disorder globally.

Unfortunately, the demand for mental health services far exceeds the available human resources able to meet this need in high-income and low-income andmiddle-income countries. This includes ever-growing treatment gaps[2] and lags.[3] These alarming increases have prompted the 2018 Lancet commission on global mental health to suggest that universal health coverage should include efforts to ensure the sustainable development of mental health.[4] Innovations to promote accessibility to mental health treatments include technology such as telephone, internet and smartphone devices, augmenting the psychotherapeutic toolkit.[5]

Innovations in video gaming for remediating mental health issues have wide potential application. In the USA, over 164 million adults play video games, and at least three-quarters of all American families have at least one person who video games regularly.[6] In the EU, 54% of the population play video games between the ages of 6 and 64, where the average age of video gamers is 31, and with a distribution of 46% female and 54% male. Of these, 77% play at least 1 hour per week, 16% play 1 hour per month, while only 7% play 1 hour per year.[7] Given that such a large proportion of the Western population play video games, developing mental health training in the form of psychoeducation may have great potential for building psychological resilience and

For peer review only - http://bmjopen.bmj.com/site/about/guidelines.xhtml

BMJ Open: first published as 10.1136/bmjopen-2020-041667 on 16 November 2020. Downloaded from http://bmjopen.bmj.com/ on October 15, 2023 by guest. Protected by copyright.

helping to better manage depression, anxiety and other forms of distress.

Technological developments for tackling such challenges include the exploitation of gamification.[8] This involves the application of behavioural principles for controlling and modifying human behaviour, in which game design elements are used to increase human interaction with or without technology.[9] Some examples of gamification include gamifying the development of cognitive skills and emotional regulation by rewarding the completion of relevant tasks within complex video games[10–13] in order to promote mental health.

When mental health-related video games are designed well, they have been shown to elevate self-esteem, self-efficacy, knowledge and awareness of illness, adherence to treatment and problem-solving skills, while lowering aggression.[14] One of the most successful in the facilitation of mental health improvement is a serious video game—a complex game with multiple levels and settings—called PlayMancer (PM), which targets emotional regulation and was specifically designed to help manage impulse control disorders.[11 13] The objective of the PM game is to develop emotional and cognitive skills, while reducing impulsivity. The game has been shown to help treat bulimia nervosa by improving emotional regulation.[15 16]

PM also uses biofeedback (heart rate and heart rate variability) to model physiological and emotional reactions, feeding this information back to the participant. Some research has shown that facilitating awareness of one's own physiology (such as brain activity or cardiac function) enhances the treatment effects of mental health disorders (such as anxiety disorder, depression, obsessive-compulsive disorder (OCD) and schizophrenia) via self-regulation.[17] Biofeedback has also been shown to improve impulse control difficulties, and attentional difficulties in bulimia nervosa and attention deficit hyperactivity disorder,[15 16 18] as well as symptoms of stress, anxiety and anger.[19] The focus on physiological data in the psychotherapeutic context is gaining traction[20–22] and has strong theoretical underpinnings.[23–25]

Within PM, there are three mini-games: 'The face of Cronos'; 'Treasures of the sea' and 'Sign of the Magupta'. Each of these mini-games were designed to train different skills, for example, 'The face of Cronos' and 'Treasures of the sea' develops planning skills, impulse control, coping skills, stress management and emotional self-regulation, while 'Sign of the Magupta' was designed to train relaxation, breathing techniques and improve physiological and emotional awareness. However, in the study[15] PM was combined with sessions of cognitive behavioural therapy (CBT) and without a control measure (eg, CBT only) so the game was developed as an adjunct to traditional mental health training, and there is no real way of knowing the direct benefits of the game as opposed to training in CBT. In another study—a case study of a single participant playing PM—anxiety and impulsivity decreased prior to CBT.[16] However, as this study was based on a single case, further studies using a randomised controlled trial (RCT) approach are needed to support and provide confidence to these findings.

Another game, Dojo,[26] develops emotional regulation in adolescents with anxiety. It uses biofeedback (heart rate variability) and trains breathing techniques, muscle relaxation, positive thinking and guided imagery to attempt to reduce anxiety in adolescence. It also uses instructional videos and then engages players through immersive and emotionally evocative puzzles that challenge players to use newly acquired emotion regulation skills. However, a pre-post RCT with 1347 participants, compared with a standard 'off the shelf' commercial game 'Rayman 2' (whereby Rayman 2 was the control), reported no difference between Dojo and the control condition at reducing anxiety. As both of these games significantly reduced anxiety, it is possible that the reduction in anxiety was due distraction from anxiety-provoking thoughts, rather than developing psychoeducational skills per se. The authors concluded that crucial design issues need to be carefully thought through, which include a clear theoretical and therapeutic foundation. This includes appropriate methodology that can assess the causes of improvement, before developing and testing a serious video game for the treatment of mental health issues such as anxiety.

Commercial games (such as Rayman 2) have been explored in their unmodified forms for their effectiveness in helping with social skills training for autism, and cognitive distraction for anxiety and nausea for patients undergoing chemotherapy,[27] with limited success. Evidence of generalisability of these games beyond game-playing is limited,[28] and this may be because they act as simple distractions rather than therapeutic psychoeducation applicable to participant's everyday lives. Another issue with many of these studies is that they often lack appropriate and rigorous methodology such as longitudinal follow-up,[29] and a mixed-methodological approach that can assess the feasibility and acceptability of such interventions.

Given these issues, it is important to emphasise that the underlying theoretical basis for PM and Dojo relates to the development of emotional regulation skills. While emotional regulation has transdiagnostic application[30] (ie, an intervention designed to treat multiple mental health conditions), these applications are not underpinned by theoretical frameworks that relate to formal psychotherapeutic interventions. Our proposed game is designed to be a comprehensive transdiagnostic intervention that will integrate a third wave behavioural therapy—as opposed to an adjunct to—acceptance and commitment therapy (ACT).[31 32] It will therefore be a comprehensive strategy for managing many common mental health issues such as depression and anxiety and focus on developing clear psychoeducational skills in the form of psychological flexibility, well-being and resilience more generally.[33]

Given this comprehensive transdiagnostic focus on psychological flexibility through ACT—a fundamental component of general health and well-being[34]—our online video game may have much greater reach and

For peer review only - http://bmjopen.bmj.com/site/about/guidelines.xhtml

BMJ Open: first published as 10.1136/bmjopen-2020-041667 on 16 November 2020. Downloaded from http://bmjopen.bmj.com/ on October 15, 2023 by guest. Protected by copyright.

impact than other serious video games such as PM, Dojo and many of the commercial games which are not based on third wave psychotherapy. Greater accessibility and impact have important implications for reducing treatment gaps and lags by making more mental health services available to those who need them.

One reason for choosing ACT in the game development process was pragmatism. For instance, researchers and clinicians may access freely available materials through the Association of Contextual Behavioural Science website (https://contextualscience.org/), and it does not require formal clinical training or accreditation to practice[35] which has important implications for translation to video game platforms. Another reason for choosing ACT as the basis for the game, is that it has a strong evidence base, and meta-analysis has found it to be efficacious for improving chronic pain, depression, psychotic symptoms, mixed anxiety, OCD, drug abuse and stress at work.[36] This means it is an ideal general purpose therapeutic tool as opposed to restricted focus on for example impulsivity control such as the PM application[11 13] or simple relaxation skills for adolescence with anxiety, as is the focus of the Dojo game.[26]

ACT principles are designed to undermine the trappings of language in the form of difficult thoughts and associated feelings, and promotion of psychological flexibility.[37] Language trappings can get individuals entangled and can prevent them from engaging with what is truly meaningful to them. The development of psychological flexibility through ACT is important because it is considered to be a fundamental component of well-being.[34]

The six ACT processes are: (1) the act of being in the here and now, present and mindful[32 38]; (2) acceptance, the act of being aware and open to painful thoughts; (3) cognitive fusion, the act of recognising that thoughts are just thoughts and not to buy into them (the process of cognitive defusion)[39]; (4) identifying values, values act as a life compass and direct us towards a life filled with purpose; (5) commitment to values orientation, which is the act of continually working towards a values orientation, even when an individual goes off track; (6) self as context (also called the transcendental self), is flexible and transcendent form of self. This involves the awareness of thoughts and feelings but the complete detachment from the literal meaning of thoughts.[34]

ACT has been usefully applied to many forms of mental health issues and has been applied in many different forms of delivery. This includes web-based interventions,[40–42] teleconference[43] and a downloadable app for smartphones.[44 45] So, given the fact that video games can have positive well-being benefits,[46 47] and are applicable for therapeutic purposes,[28 48] a transdiagnostic ACT serious video-game may have great potential for similar reasons.

As ACT is a comprehensive transdiagnostic model and formal third wave cognitive behavioural approach, then its reach and impact in the form of a video game may be greater than that of PM or Dojo which were focused on simpler emotional skills development and biofeedback. For these reasons, we are proposing an ACT-based video game called 'ACTing Mind' as an innovative and accessible intervention to help individuals who struggle with anxiety, depression, stress and other forms of distress.

Aims

The research goals of this proposal are to determine the feasibility and acceptability of a novel ACT-based video game intervention for individuals with mental distress, in line with methodology described in the Medical Research Council (MRC) framework.[49 50] This proposal lays the foundation for which a pilot and full-scale RCT will be conducted to determine clinical effectiveness, and ultimately the recommendations of the importance of such innovations in primary care mental health policies and practices.

METHODOLOGY

This protocol has been developed following the Template for Intervention Description and Replication of Studies[51] (see online supplemental appendix 1), as well as the MRC guidelines for the development of complex interventions.[49 50] This includes five stages of development for a complex intervention including: (1) preclinical, involving a theoretical review of the literature (provided here), justifying the need for such an intervention for the proposed population; (2) phase I, modelling, involving the use of evidence to determine the components for underlying mechanisms. For this, we propose a qualitative element involving thematic analysis to enable us to understand what would be most beneficial to a general population with anxiety and depression; (3) phase II, conducting an exploratory pilot study (outlined here) to determine the feasibility of the methodology and design where some initial data can be collected; (4) phase III, an RCT to test the efficacy of the proposed intervention (in subsequent work); (5) phase IV, long-term follow-up to assess replicability.

Public and patient involvement

Key stakeholders were consulted and involved in the development of this protocol. The Patient Experience and Evaluation in Research (Patient Experience and Evaluation in Research (PEER): https://www.swansea.ac.uk/humanandhealthsciences/research-at-the-college-of-human-and-health/patientexperienceandevaluationinresearchpeergroup/) group in the College of Human and Health Sciences at Swansea University were consulted. This group represented members of the public, students and staff members, several of whom reported that they had experienced depression, anxiety or stress at some point in their lives and emphasised the need for innovative approaches of the delivery of mental health support. The feasibility design was explained to them, and they gave positive feedback about the nature of the design, intervention and outcome measures.

For peer review only

BMJ Open: first published as 10.1136/bmjopen-2020-041667 on 16 November 2020. Downloaded from http://bmjopen.bmj.com/ on October 15, 2023 by guest. Protected by copyright.

Study design

This is a mixed-methods study which is designed to determine the feasibility and acceptability of an ACT-based video game for individuals with anxiety, depression and stress, and to increase psychological flexibility.

Study setting

The study will be conducted entirely online including the game and assessment (via the Qualtrics platform), and qualitative interviews (via the Zoom platform). Thus, potential participants will be able to access this study without restrictions, an important consideration for ongoing local lockdowns associated with the COVID-19 pandemic. Strict recommendations will require participants to ensure they are in a quiet room and without disruption for the duration of the study.

Recruitment and consent

We will recruit participants (n=36) using purposive sampling, focusing on—unlike an opportunity sample—the types of participants needed for a full-scale RCT (ie, individuals with depression, anxiety and stress). The sample size is justified on the basis of past research reporting the median numbers of participants recruited for similar types of feasibility studies,[52] incorporating both quantitative and qualitative elements.

Eligibility criteria

Participants will be recruited through general public mental health forums, social media and student populations. Thirty-six participants will take part in the study and they will be aged 18 years or older, be experiencing ongoing depression, anxiety and stress, and be able to read, write and speak English.

Intervention

This ACT-based video game intervention called 'ACTing Mind', developed and designed solely by DE, will involve students and members of the public attending five 1 hour sessions of an ACT-based video game. Each session will involve a different chapter of the video game, and each chapter will explore a different key component of ACT, with there being six in total (see table 1 for the different chapters and sessions involved).

These various components and principles of ACT[31 32] will be taught within the different chapters of the game and through embedded learning. For example, the player will gain ACT skills while completing objectives within the game and without directly being taught these skills, but rewarded indirectly through points and progress awards. For instance, in one scene (see table 1) the character is confronted by painful memories, and the player has two choices: (1) to destroy the painful memories or (2) to accept these memories. If the player chooses to destroy the memories (avoidant-based strategies), the world becomes distorted and barriers form making the chapter impossible to complete. Alternatively, if the player chooses acceptance-based strategies they will be able to continue the game (hence in this scene they learn that acceptance is functionally better than avoidance).

The game will start with a depressed individual who has recently lost his wife in an accident, and is feeling depressed, isolated and lonely (see figure 1 as an example of this scene). Each chapter will reward ACT consistent behaviour with points on a 'psychoflexameter'. This is a dial on the border of the screen which indicates increased psychological flexibility as the player completes ACT-based tasks such as acceptance (chapter 1), being present (chapter 2), values and commitment (chapter 3), defusion (chapter 4) and self as context (chapter 5). ACT uses metaphors to help clients visualise the key processes of ACT. In the game, these metaphors are real representations, such as the 'sinking sand' game, 'dropping the rope' game, the 'chessboard game', the 'unwanted monster' game, the 'leaves on a stream' game (see table 1).

Within the game, the character will have to enter his own mind through a 'mind escape machine' (see figure 2 of this as an example of the character in his own mind). At the start of the game, it is explained through a brief historical story that he develops this machine to destroy and supress his unwanted painful thoughts and memories about his wife and loss. Once in his mind, he will learn that destroying or supressing thoughts creates barriers in his mind which prevents him from continuing the game. So, learning acceptance is crucial throughout this game and the character is rewarded for this through points and progress awards. Also, within the game, psychoeducation components explain thoughts as trappings of language which can often get people stuck in life, and prevent them from value consistent living, as well as the various emotional regulation strategies such as avoidance and acceptance.

As part of the study, in addition to playing the video game, participants will be asked to record events on a weekly basis, aspects of application of the ACT principles learnt in an everyday life in a journal. It is anticipated that greater adherence to the intervention in everyday life, and engagement with the journal will lead to greater success of the intervention (greater psychological flexibility).

Data collection and management

MSc students will have the opportunity to be involved in this study and will collect and process the data under supervision by project leads, DJE and AK. Questionnaires will be completed online through Qualtrics which will store raw data copies, and also be held on an encrypted university server. Names and other personally identifiable information will not be stored, and consent form information will not be associated with the raw or processed data, instead each participant will be given a unique identifier code. Similarity recorded interviewer transcripts will use identifier codes as opposed to personal information (eg, names). The project leads (DJE, AHK) will frequently audit all processes in data collection and processing to ensure that the procedures stated in this protocol are adhered to.

For peer review only

BMJ Open: first published as 10.1136/bmjopen-2020-041667 on 16 November 2020. Downloaded from http://bmjopen.bmj.com/ on October 15, 2023 by guest. Protected by copyright.

Table 1 Overview of the 'ACTing Mind' intervention and everyday journal instructions

Session 1 (week 1): acceptance and openness to pain	► Chapter 1—Acceptance<br>► Introducing participants to the video game and ACT in everyday journal.<br>► A brief overview of the purpose of the programme and the content of each session.<br>► Explaining basic ACT tenets through introduction text of journal.<br>► Explaining the nature of painful thoughts and memories and getting caught up in the struggle explained through journal.<br>► Basic story context about the character being depressed and why, at start of video game.<br>► Explaining the objective of the video game, that is, to transcend form psychological inflexibility to psychological flexibility.<br>► Exercise, within the game there are choice, either to suppress, and break thoughts, or to accept and be open to them.<br>► Acceptance and openness are rewarded by psychological flexibility points on the 'psychoflexameter' and game progression, while suppression actions (breaking or suppressing painful memories) are punished with physical barriers, and sinking sand, which prevent the player from progressing in the game.<br>► A monster pulls against the player to prevent progress, but if the player fights with the monster, they get even more stuck (analogous to the drop the rope and sinking sand metaphor). Again, acceptance is important and must be learnt here.<br>► Reflecting in the journal about how this might be applied in life, and when this has occurred throughout the week daily.
Session 2 (week 1): being present (mindfulness)	► Chapter 2—Being present (mindfulness)<br>► Some instructions form the journal about being present and mindful is given, why it is useful and how to go about achieving with breathing exercises.<br>► The character is approached by monsters in the game in the past and future making him worry excessively about imaginary dangers, and reminding him of painful events.<br>► The game (in the form of the character's wife's ghost) instructs the player to be present, to focus on your breathing for 10 min.<br>► As the participant learns and completes relevant psychological flexibility tasks psychological flexibility on the 'psychoflexameter' will increase, which rewards the player for being present.<br>► Reflecting in the journal about how this might be applied in life, and when this has occurred throughout the week daily.
Session 3 (week 2): values identification and commitment	► Chapter 3—Values identification and commitment<br>► Instructions about what are values (a life compass) explained through the journal.<br>► Acceptance and commitment to values orientation as opposed to avoidance behaviour is rewarded.<br>► There are challenges to reach goals which are linked to the character's values, such as scary weather and monsters.<br>► Psychological flexibility on the 'psychoflexameter' and game progress, will increase with values consistent behaviour which rewards the player for committing to values.<br>► Reflecting in the journal about how this might be applied in life, and when this has occurred throughout the week daily.
Session 4 (week 2): defusion	► Chapter 4—Defusion<br>► Instructions about what is cognitive fusion and defusion (holding self-stories lightly) explained through journal.<br>► The character goes back into the 'Mind Escape' machine but this time there is a flowing river with leaves (analogous to leaves on a stream metaphor).<br>► Some of the character's painful memories will beg the player to help them, but if the player interacts, barriers and quicksand appear, punishing the player and preventing them from progressing in the game (analogous to the sinking sand metaphor).<br>► The ghost of the character's wife eventually instructs the player to put the memories and thoughts onto the leaves and watch them flow down the river, without interacting with them, and to simply observe.<br>► Psychological flexibility on the 'psychoflexameter', will increase when all of the memories and thoughts as left to go down the stream, hence the player is rewarded for defusing.<br>► Reflecting in the journal about how this might be applied in life, and when this has occurred throughout the week daily.

Continued

BMJ Open: first published as 10.1136/bmjopen-2020-041667 on 16 November 2020. Downloaded from http://bmjopen.bmj.com/ on October 15, 2023 by guest. Protected by copyright.

For peer review only - http://bmjopen.bmj.com/site/about/guidelines.xhtml

Table 1 Continued

Session 5 (week 3): self as context	► Chapter 5—Self as context<br>► Instructions about what is self as context (being the observer of your thoughts and not your thought) are explained through journal.<br>► The world starts to fall apart and becomes abstract, like a chess board.<br>► The player realises that they are the white pieces on the chessboard (analogous to chess board metaphor).<br>► The player is compelled by the game to beat the black pieces in the chess game.<br>► But the more the players fights against the black pieces, the more they lose points on the 'psychoflexameter' and cannot progress in the game.<br>► The player must let the battle play out, once they do, they become aware that they are the chess board (they become it) and realise they do not need to be part of the never-ending battle between the opposing forces.<br>► Finally, a bus arrives, memories of the character's wife beg the player to stay, and the monsters pull on player.<br>► The player needs to get onto the bus with the monsters to move towards their values, a new beginning (analogues to bus metaphor).<br>► Finally, the player has a choice, go back and change the events that led to your wife's death, or stay on the bus with the monsters.<br>► Trying to change events leads to a loss in points and prevents game progression.<br>► Only staying on the bus, towards values, and accepting the monsters allows the player to complete the game successfully.<br>► Reflecting in the journal about how this might be applied in life, and when this has occurred throughout the week daily.

Outcome measures

Questionnaires will be collected at three points in time (baseline, immediate postintervention and 3-month follow-up). Interview data will be collected immediate postintervention only.

Demographic data

Demographic measures will include age, sex, medication use, which will all be recorded through Qualtrics and assessed by DJE and AHK.

Primary outcome measure

The primary outcomes for feasibility are determined using MRC framework measure for developing a complex intervention.[49 50] As this is a feasibility study, the primary outcomes (in accordance with the MRC framework) will include the acceptability of the ACT-based video game intervention, the feasibility of the recruitment, outcome measures and intervention adherence.

Acceptability

- Number of people dropping out.
- Barriers for adoption of intervention as assessed through interviews.
- Number of sessions attended.
- Time dedicated to home journal.
- ACT principles adherence in everyday life setting (as recorded in journal and expressed through interviews).
- Experience, identifying whether participants had positive experience with the intervention and whether they wanted to continue to be part of the intervention.

Feasibility

- Number of participants who are willing to take part.
- Time taken to complete questionnaires.
- Number of complete and incomplete questionnaires.

Secondary outcome measures

Warwick-Edinburgh Mental Well-Being Scale[53]: a measure of mental well-being with a focus on positive aspects of mental health. This measure has good internal

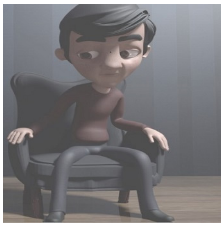

Figure 1 First scene in 'ACTing Mind', the character, Steve, is depressed and alone.

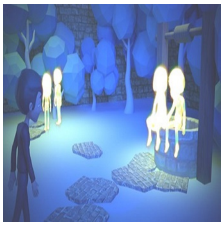

Figure 2 An example scene, where the character 'Steve' is in his own mind, and can see his own memories, through his Mindscape machine.

For peer review only - http://bmjopen.bmj.com/site/about/guidelines.xhtml

Edwards DJ, Kemp AH. *BMJ Open* 2020;**10**:e041667. doi:10.1136/bmjopen-2020-041667

BMJ Open: first published as 10.1136/bmjopen-2020-041667 on 16 November 2020. Downloaded from http://bmjopen.bmj.com/ on October 15, 2023 by guest. Protected by copyright.

consistency with a Cronbach's alpha coefficient of 0.89 (student sample) and 0.91 (general population sample).

Depression Anxiety Stress Scales: a short version of this measure and a measure of general psychological distress with good construct validity (confirmatory factor analysis of 0.94). It has good internal reliability as measured through Cronbach's alpha coefficients, which are 0.88 for depression, 0.82 for anxiety, 0.90 for stress and 0.93 for the total scale.[54]

Social connectedness (adapted from Russell's (1996) UCLA Loneliness Scale[55]: this measure involves two questions: (1) "During social interactions, I feel 'in tune' with the person/s around me", and (2) "During social interactions, I feel close to the person/s". The Cronbach's alpha coefficients for these two items ranged from 0.80 to 0.98 (M=0.94, SD=0.03).[55]

EuroQol five dimensions (EQ5D): the EQ5D is a measure for health-related quality of life. There are five components within this measure which assess mobility, self-care, usual activities, pain, discomfort and anxiety. It also has a visual analogue scale (VAS) for measuring current health status. Scores for these will be calculated for each of these five subsections as well as including the VAS and total EQ5D score of all five subsections. The EQD5 correlates well with other health-related questionnaires such as the 36-Item Short Form Survey (r=0.61, p<0.0001) and Parkinson's Disease Questionnaire (PDQ)-39 (r=−0.75, p<0.0001).[56]

Acceptance and Action Questionnaire-second version: this is a seven-item scale developed by Bond *et al*[57] to measure psychological inflexibility, which involves the ability to accept and be open to difficult thoughts and feelings as well as to engage in valued behaviour in the presence of the difficult thoughts and feelings. A higher score indicates higher psychological inflexibility. The measure has good construct validity with a Cronbach's alpha coefficient of 0.84.[57]

Adherence to the intervention measure and trial

Adherence will be measured in a variety of ways such as intervention feedback, treatment adherence through attrition rates as well as meta-data of relating to game log-in and log-out, as well as how long the game was played for and what sessions of the game were completed for each participant. Similar information can be recorded in Qualtrics for ensuring questionnaires are completed carefully. This includes length of times completing the questionnaire, and paying attention to reverse-scored questions.

Sample size and statistical analysis

Sample size recruited will help us determine whether it is possible to recruit sufficient numbers of participants to manage a full-scale RCT at a later date.

Quantitative data analysis: analysis will focus on descriptive statistics and feasibility outcomes of the questionnaires. While clinical effectiveness will not be formally evaluated at this stage, effect sizes will be explored for early evidence that the intervention shows promising signs (including ACT-related process measures). It is predicted that outcomes will improve, and any improvement will be identified using a one-way analysis of covariance with a single within-subjects factor (time). The effect sizes will also allow for a power calculation to be made which will allow for an approximation for a sample size required in a future trial (if indicated).

Qualitative data analysis: transcripts of focus group interview data will be generated from digital audio-recordings of in-depth, face-to-face semi-structured interviews (all online and via a password-protected room in Zoom). In-depth semi-structured interviews will form the core topics to be discussed (see table 2), while leaving space and scope for the identification and exploration of unforeseen information that may emerge. Insights from this will allow for further development and improvement of the intervention, along with the quantitative data in line with the MRC guidelines.[49 50]

Thematic analysis will then be conducted which will explore key overarching themes that may emerge from the focus group interviews following standardised guidelines.[58] The interview questions are based on other novel ACT-based protocols.[40 59] The data will be analysed after the study has been completed. We will follow the inductive and deductive code development as outlined by Fereday and Muir-Cochrane[60] to ensure necessary rigour. Any key overarching themes identified which relate to feasibility of the study design of the acceptability of the intervention, as well as potential adverse effects, will be explored and reported.

The focus groups will comprise 4 to 6 groups with 6 to 10 individuals in each group as has been suggested as optimal in other studies.[61] The interview will take place at the end of the intervention (week 3). It will explore various aspects of the intervention such as perceived process of change, barriers to intervention adherence, trial process and any adverse effects, which help supplement the quantitative approach. Process of change questions indicate whether the participant learnt anything about ACT, and felt any positive change in their life due to participating in the intervention. The question relating to barriers explores any problems and difficulties they had with the intervention. Another question will be asked to elicit suggestions for improvement relating to game or study design. Acceptability questions and process of change in one's life relate to whether the participant accepted the intervention and used skills they learnt through the intervention in daily life. The question relating to the trial process will determine whether there were any difficulties or limitations of the trial itself such as whether the instructions were clear and how it could be improved. Finally, the question on adverse effects explores whether there were any potential unforeseen negative consequences of the intervention.

Limitations of the study

This study protocol has limitations. First, while physiological measures would ideally be collected to measure

For peer review only - http://bmjopen.bmj.com/site/about/guidelines.xhtml
BMJ Open: first published as 10.1136/bmjopen-2020-041667 on 16 November 2020. Downloaded from http://bmjopen.bmj.com/ on October 15, 2023 by guest. Protected by copyright.
For peer review only

Table 2 Qualitative interview protocol for the focus groups

Acceptability and feasibility	How would you describe your experience of taking part in 'ACTing Mind' video game programme?
Accessibility of intervention	If this intervention were rolled out as a video game app, do you think you would download it? Would you appreciate the accessibility?
Process of change	What did you learn from this programme?
Acceptability	What was the aspect of the programme that you liked the most? What was your favourite activity within the game (or applied to your everyday life)?
Suggestions for further improvement	What did you least like about the intervention? What do you think could be improved?
Barriers	Were there any difficulties to taking part?
Implementing change in everday life	Do you practice mindfulness, acceptance, defusion and values? How often? Could you apply what you have learnt through video game intervention to the real world in everyday events? Will you apply this new knowledge to everyday events?
Process of change	Have you noticed any differences in your life as a result of taking part in 'ACTing Mind'? If 'yes', what are these differences?
Acceptability	Would you recommend this intervention to someone you care about? Did you like the theoretical concepts central to the ACT intervention? How did you feel about its delivery? Was any of it too abstract or difficult to understand?
Processes of the trial	Was there anything you liked, or disliked about the study? How could we improve this study? Were all the instructions clear?
Adverse effects	Did you feel that any aspect of the intervention may have made worse any aspect of your anxiety, depression or stress? Were there any adverse effects that you can recognise due to the intervention?

variables such as heart rate variability, the COVID-19 pandemic limits our capacity to do this. However, the present study will provide important data on which such measures could be collected, analysed and interpreted in a future trial. Second, it could be questioned why there is no control condition in this study. Our response to this potential criticism is that the aim of the present study is to assess feasibility and—in line with the MRC guidelines[49 50]—has not been designed to be a full-scale RCT given the current research phase. Once the feasibility component is completed, a control condition will be introduced, which allow for the intervention condition to be compared with control, and as part of a full trial. Finally, although we would like to have ability to monitor the participant more directly, to ensure adherence to the intervention, we are sensitive to privacy issues associated with, for example, capturing participants' identity from the computers video camera. To mitigate this limitation, we have opted for less invasive procedures for measuring intervention adherence that will include logging metadata of the game such as log in and out times, as well as completion of game sections. Several questions in the questionnaire are also reverse scored to ensure participants are paying attention.

Protocol amendments

If the protocol is amended in any way, it will be communicated to relevant parties immediately, such as to participants, journal and ethics committee.

Ethics and dissemination

This study has received ethical approval from Swansea University Psychology Department ethics committee (2020-4920-3923). Participants will be informed of their rights to confidentiality and to leave the study at any time and without penalty. Both qualitative and quantitative data will be held on a password-protected computer accessible only to researchers DJE and AHK. The data will be anonymised with a unique identifier code, and any personally identifiable information will be removed.

Dissemination will involve peer-reviewed journals; leading national and international conferences, social media and public events and through general public health engagement such as talks at schools, the Welsh Government and engagement with annual science festivals including 'a pint of science'.

Impact of intervention

The potential impact of this study is far reaching as it will add to the growing set of online resources which support psychological resilience, flexibility and well-being. These resources are designed to be easy to access and are ideal for situations where travel is limited due to physical (disability) or situational (coronavirus) immobility. Such interventions can help alleviate widely reported mental health treatment gaps[2] and lags,[3] associated with the widely reported scarce human resources needed to provide mental health support for the many individuals who need it. The 2018 Lancet commission on global mental health argued that sustainable development

For peer review only - http://bmjopen.bmj.com/site/about/guidelines.xhtml

BMJ Open: first published as 10.1136/bmjopen-2020-041667 on 16 November 2020. Downloaded from http://bmjopen.bmj.com/ on October 15, 2023 by guest. Protected by copyright.

of mental health should be an essential component of universal health coverage.[4] Technological innovation of mental health support services, in the form of video games, may be one means to achieve this sustainability and a reduction in the treatment gap and lag.

Ancillary and poststudy care

Postintervention care has not been anticipated given this is a low-level (low-risk) intervention. Of course, all participants will be given a debrief form which will signpost individuals to the relevant free well-being services such as the Samaritans.

Acknowledgements The authors would like to thank Professor Louise McHugh (University College Dublin), who provided some very helpful feedback about the gamification of an ACT-based approach through the utilisation of behavioural principles.

Contributors DJE developed the intervention. DJE and AHK agreed on a set of outcomes. DJE wrote the first draft of the protocol and DJE and AHK then revised the subsequent drafts of the protocol. Both authors helped to revise the manuscript for intellectual content and agreed on the final version prior to submission for peer review.

Funding The authors have not declared a specific grant for this research from any funding agency in the public, commercial or not-for-profit sectors.

Competing interests At the time of writing this, DJE is discussing with Agor IP at Swansea University the potential to commercialise the described video game as a mobile application; however, at this time no agreements have been made or signed. AHK has no competing interests.

Patient consent for publication Not required.

Provenance and peer review Not commissioned; externally peer reviewed.

Supplemental material This content has been supplied by the author(s). It has not been vetted by BMJ Publishing Group Limited (BMJ) and may not have been peer-reviewed. Any opinions or recommendations discussed are solely those of the author(s) and are not endorsed by BMJ. BMJ disclaims all liability and responsibility arising from any reliance placed on the content. Where the content includes any translated material, BMJ does not warrant the accuracy and reliability of the translations (including but not limited to local regulations, clinical guidelines, terminology, drug names and drug dosages), and is not responsible for any error and/or omissions arising from translation and adaptation or otherwise.

Open access This is an open access article distributed in accordance with the Creative Commons Attribution Non Commercial (CC BY-NC 4.0) license, which permits others to distribute, remix, adapt, build upon this work non-commercially, and license their derivative works on different terms, provided the original work is properly cited, appropriate credit is given, any changes made indicated, and the use is non-commercial. See: http://creativecommons.org/licenses/by-nc/4.0/.

ORCID iD
Darren J Edwards http://orcid.org/0000-0002-2143-1198

REFERENCES

1 Whiteford H, Ferrari A, Degenhardt L. Global burden of disease studies: implications for mental and substance use disorders. *Health Aff* 2016;35:1114–20.
2 Patel V, Maj M, Flisher AJ, *et al*. Reducing the treatment gap for mental disorders: a WPA survey. *World Psychiatry* 2010;9:169–76.
3 Wang PS, Berglund PA, Olfson M, *et al*. Delays in initial treatment contact after first onset of a mental disorder. *Health Serv Res* 2004;39:393–416.
4 Patel V, Saxena S, Lund C, *et al*. The Lancet commission on global mental health and sustainable development. *The Lancet* 2018;392:1553–98.
5 Kazdin AE, Blase SL. Rebooting psychotherapy research and practice to reduce the burden of mental illness. *Perspect Psychol Sci* 2011;6:21–37.
6 ESA. 2019 essential facts about the computer and video game industry, 2020. Available: https://www.theesa.com/esa-research/2019-essential-facts-about-the-computer-and-video-game-industry/
7 ISFE. ISFE key facts 2019, 2020. Available: https://www.isfe.eu/isfe-key-facts/
8 Linehan C, Kirman B, Roche B. *Gamification as behavioral psychology. In the gameful world: approaches, issues, applications*. Cambridge, Massachusetts: MIT Press, 2015.
9 Deterding S, Dixon D, Khaled R, *et al*. From game design elements to gamefulness: defining "gamification". *Proceedings of the 15th international academic MindTrek conference: Envisioning future media environments*, 2011:9–15.
10 Jon Hobbs L, Yan Z. Cracking the walnut: using a computer game to impact cognition, emotion, and behavior of highly aggressive fifth grade students. *Comput Human Behav* 2008;24:421–38.
11 Jiménez-Murcia S, Fernández-Aranda F, Kalapanidas E, *et al*. Playmancer project: a serious videogame as an additional therapy tool for eating and impulse control disorders. *Stud Health Technol Inform* 2009;144:163–6.
12 Ducharme P, Wharff E, Hutchinson E, *et al*. Videogame assisted emotional regulation training: an act with RAGE-Control case illustration. *Clin Soc Work J* 2012;40:75–84.
13 Fernández-Aranda F, Jiménez-Murcia S, Santamaría JJ, *et al*. Video games as a complementary therapy tool in mental disorders: PlayMancer, a European multicentre study. *J Ment Health* 2012;21:364–74.
14 Santamaria JJ, Antonio S, Fernando F-A, *et al*. Serious games as additional psychological support: a review of the literature. *J Cyber Ther Reh* 2011;4:469–76.
15 Fagundo AB, Santamaría JJ, Forcano L, *et al*. Video game therapy for emotional regulation and impulsivity control in a series of treated cases with Bulimia nervosa. *Eur Eat Disord Rev* 2013;21:493–9.
16 Giner-Bartolomé C, Fagundo AB, Sánchez I, *et al*. Can an intervention based on a serious videogame prior to cognitive behavioral therapy be helpful in Bulimia nervosa? a clinical case study. *Front Psychol* 2015;6:982.
17 Schoenberg PLA, David AS. Biofeedback for psychiatric disorders: a systematic review. *Appl Psychophysiol Biofeedback* 2014;39:109–35.
18 Howard R, Schellhorn K, Lumsden J. A biofeedback intervention to control impulsiveness in a severely personality disordered forensic patient. *Personal Ment Health* 2013;7:168–73.
19 Pawlow LA, O'Neil PM, Malcolm RJ. Night eating syndrome: effects of brief relaxation training on stress, mood, hunger, and eating patterns. *Int J Obes Relat Metab Disord* 2003;27:970–8.
20 Dana DA. *The Polyvagal theory in therapy: engaging the rhythm of regulation (Norton series on interpersonal neurobiology)*. New York: WW Norton & Company, 2018.
21 Lehrer PM. Heart rate variability biofeedback and other psychophysiological procedures as important elements in psychotherapy. *Int J Psychophysiol* 2018;131:89–95.
22 Tulip C, Fisher Z, Bankhead H, *et al*. Building wellbeing in people with chronic conditions: a qualitative evaluation of an 8-week positive psychotherapy intervention for people living with an acquired brain injury. *Front Psychol* 2020;11:66.
23 Kemp AH, Koenig J, Thayer JF. From psychological moments to mortality: a multidisciplinary synthesis on heart rate variability spanning the continuum of time. *Neurosci Biobehav Rev* 2017;83:547–67.
24 Mead J, Wilkie L, Gibbs K, *et al*. Rethinking wellbeing: toward a more ethical science of wellbeing that considers current and future generations, 2019. Available: https://www.authorea.com/doi/full/10.22541/au.156649190.08734276
25 Kemp A. *Fisher, social ties, health and wellbeing: a literature review and model, in neuroscience and social science*. Berlin: Springer, 2017: 397–427.
26 Scholten H, Malmberg M, Lobel A, *et al*. A randomized controlled trial to test the effectiveness of an immersive 3D video game for anxiety prevention among adolescents. *PLoS One* 2016;11:e0147763.
27 Colder Carras M, Van Rooij AJ, Spruijt-Metz D, *et al*. Commercial video games as therapy: a new research agenda to unlock the potential of a global pastime. *Front Psychiatry* 2017;8:300.
28 Griffiths MD, Kuss DJ, de Gortari ABO. Videogames as therapy: an updated selective review of the medical and psychological literature. *JPHIM* 2017;5:71–96.
29 Zayeni D, Raynaud J-P, Revet A. Therapeutic and preventive use of video games in child and adolescent psychiatry: a systematic review. *Front Psychiatry* 2020;11:36.
30 Sloan E, Hall K, Moulding R, *et al*. Emotion regulation as a transdiagnostic treatment construct across anxiety, depression, substance, eating and borderline personality disorders: a systematic review. *Clin Psychol Rev* 2017;57:141–63.
31 Hayes S. *Acceptance and commitment therapy*. Washington, DC: American Psychological Association, 2009.

For peer review only

BMJ Open: first published as 10.1136/bmjopen-2020-041667 on 16 November 2020. Downloaded from http://bmjopen.bmj.com/ on October 15, 2023 by guest. Protected by copyright.

32 Hayes Steven C, Kirk D. *Acceptance and commitment therapy: the process and practice of mindful change*. New York: Guilford Press, 2011.
33 Dindo L, Van Liew JR, Arch JJ. Acceptance and commitment therapy: a transdiagnostic behavioral intervention for mental health and medical conditions. *Neurotherapeutics* 2017;14:546–53.
34 Kashdan TB, Rottenberg J. Psychological flexibility as a fundamental aspect of health. *Clin Psychol Rev* 2010;30:865–78.
35 Harris R. *Act made simple: an easy-to-read primer on acceptance and commitment therapy*. Oakland, California: New Harbinger Publications, 2009.
36 Öst L-G. The efficacy of acceptance and commitment therapy: an updated systematic review and meta-analysis. *Behav Res Ther* 2014;61:105–21.
37 Hayes SC, Strosahl KD, Bunting K, *et al*. *What is acceptance and commitment therapy?, in a practical guide to acceptance and commitment therapy*. Berlin: Springer, 2004: 3–29.
38 Strosahl K, Wilson K. *Acceptance and commitment therapy: an experiential approach to behavior change*. New York: Guilford Press, 1999.
39 Hayes SC. *Get out of your mind and into your life: the new acceptance and commitment therapy*. Oakland, California: New Harbinger Publications, 2005.
40 Edwards DJ, Rainey E, Boukouvala V, *et al*. Novel ACT-based eHealth psychoeducational intervention for students with mental distress: a study protocol for a mixed-methodology pilot trial. *BMJ Open* 2019;9:e029411.
41 Viskovich S, Pakenham KI. Pilot evaluation of a web-based acceptance and commitment therapy program to promote mental health skills in university students. *J Clin Psychol* 2018;74:2047–69.
42 Levin ME, Haeger JA, Pierce BG, *et al*. Web-based acceptance and commitment therapy for mental health problems in college students: a randomized controlled trial. *Behav Modif* 2017;41:141–62.
43 Herbert MS, Afari N, Liu L, *et al*. Telehealth versus in-person acceptance and commitment therapy for chronic pain: a randomized noninferiority trial. *J Pain* 2017;18:200–11.
44 Bricker JB, Mull KE, Kientz JA, *et al*. Randomized, controlled pilot trial of a smartphone APP for smoking cessation using acceptance and commitment therapy. *Drug Alcohol Depend* 2014;143: :87–94.
45 Levin ME, Haeger J, Pierce B, *et al*. Evaluating an adjunctive mobile APP to enhance psychological flexibility in acceptance and commitment therapy. *Behav Modif* 2017;41:846–67.
46 Johnson D, Jones C, Scholes L, *et al*. Videogames and wellbeing: a comprehensive review. young and well cooperative research centre, Melbourne, 2013. Available: https://www.youngandwellcrc.org.au/wp-content/uploads/2014/03/Videogames_and_Wellbeing.pdf
47 Vella K, Johnson D, Hides L. Positively playful: when videogames lead to player wellbeing. *Proceedings of the First International Conference on Gameful Design, Research, and Applications*, 2013:99–102.
48 Villani D, Carissoli C, Triberti S, *et al*. Videogames for emotion regulation: a systematic review. *Games Health J* 2018;7:85–99.
49 Craig P. Developing and evaluating complex interventions: following considerable development in the field since 2006, MRC and NIHR have jointly commissioned an update of this guidance to be published in 2019, 2019. Available: https://mrc.ukri.org/documents/pdf/complex-interventions-guidance/
50 Craig P, Dieppe P, Macintyre S, *et al*. Developing and evaluating complex interventions: the new medical Research Council guidance. *BMJ* 2008;337:a1655.
51 Hoffmann TC, Glasziou PP, Boutron I, *et al*. Better reporting of interventions: template for intervention description and replication (TIDieR) checklist and guide. *BMJ* 2014;348:g1687.
52 Billingham SAM, Whitehead AL, Julious SA. An audit of sample sizes for pilot and feasibility trials being undertaken in the United Kingdom registered in the United Kingdom clinical research network database. *BMC Med Res Methodol* 2013;13:104.
53 Tennant R, Hiller L, Fishwick R, *et al*. The Warwick-Edinburgh mental well-being scale (WEMWBS): development and UK validation. *Health Qual Life Outcomes* 2007;5:63.
54 Henry JD, Crawford JR. The short-form version of the depression anxiety stress scales (DASS-21): construct validity and normative data in a large non-clinical sample. *Br J Clin Psychol* 2005;44:227–39.
55 Kok BE, Coffey KA, Cohn MA, *et al*. How positive emotions build physical health: perceived positive social connections account for the upward spiral between positive emotions and vagal tone. *Psychol Sci* 2013;24:1123–32.
56 Schrag A, Selai C, Jahanshahi M, *et al*. The EQ-5D--a generic quality of life measure-is a useful instrument to measure quality of life in patients with Parkinson's disease. *J Neurol Neurosurg Psychiatry* 2000;69:67–73.
57 Bond FW, Hayes SC, Baer RA, *et al*. Preliminary psychometric properties of the acceptance and action Questionnaire-II: a revised measure of psychological inflexibility and experiential avoidance. *Behav Ther* 2011;42:676–88.
58 Braun V, Clarke V. Using thematic analysis in psychology. *Qual Res Psychol* 2006;3:77–101.
59 Saracutu M, Edwards DJ, Davies H, *et al*. Protocol for a feasibility and acceptability study using a brief ACT-based intervention for people from Southwest Wales who live with persistent pain. *BMJ Open* 2018;8:e021866.
60 Fereday J, Muir-Cochrane E. Demonstrating rigor using thematic analysis: a hybrid approach of inductive and deductive coding and theme development. *Int J Qual Methods* 2006;5:80–92.
61 Morgan DL. Focus groups. *Annu Rev Sociol* 1996;22:129–52.

For peer review only

BMJ Open: first published as 10.1136/bmjopen-2020-041667 on 16 November 2020. Downloaded from http://bmjopen.bmj.com/ on October 15, 2023 by guest. Protected by copyright.

For peer review only - http://bmjopen.bmj.com/site/about/guidelines.xhtml

Swansea University
Prifysgol Abertawe

PARTICIPANT INFORMATION SHEET

A novel ACT-based video game to support mental health through embedded learning

You are being invited to take part in some research. Before you decide whether or not to participate, it is important for you to understand why the research is being conducted and what it will involve. Please read the following information carefully.

What is the purpose of the research?

The aim of the research project is to gain insight into whether exposure to a novel-acceptance and commitment therapy (ACT) video game can improve various outcome measures of depression, anxiety, stress, and other measures relating to wellbeing.

If you are above the age of 18 and are currently experiencing ongoing mild to moderate levels of depression, anxiety, or stress you are welcome to participate in this study. For those individuals who present with high levels of depression and anxiety, we will advise the participant to consult either with a GP or with a therapist if they have one, in relation to participating in this experiment.

Participation in this study will take approximately 3 hours over three weeks. Which is broken down into 1 hour for the intervention (the video game itself); 1 hour for online one-on-one qualitative audio interviews via Zoom after completing the game, and after a 3-week follow-up; and 1 hour for the three-questionnaire assessments at baseline (pre-intervention), immediate post-intervention, and after a 3-week follow-up.

Who is carrying out the research?

The data are being collected by Prof. Andrew Kemp and research assistant Tom Gordon of the Psychology Department within the College of Human and Health Sciences at Swansea University, as well as Dr. Darren Edwards of The Department of Public Health, Policy, and Social Sciences. The research has been approved by the Department of Psychology's Research Ethics Committee.

What happens if I agree to take part?

If you are happy to take part in this research project, please click "accept" at the bottom of the consent section below, which will then allow you to complete a baseline questionnaire assessment. The questionnaire assessment includes a range of multiple-choice questions. These questions cover a range of factors including depression, anxiety, stress, and wellbeing. There are three questionnaire assessments to complete, the first of which is a baseline assessment prior to the intervention commencing, the second will be completed following the intervention, and a follow-up assessment 3 weeks following the baseline assessment. All of the questionnaires ask the same questions with the addition of demographic questions at baseline assessment, and the second also asking about adherence to the intervention.

The intervention will consist of completing a one-hour session of playing an ACT-based video game, followed by completion of a short reflection workbook. The game itself will be completed by the participant at home, using a mobile phone or any iOS device. This is an adventure game that will teach you about psychological resilience. In the game, you will control the character Steve as he embarks on a journey into his own mind, you will help as he confronts painful past memories and learns how to cope with his full potential.

Version 4 4 November 2022

For peer review only

You will also be interviewed, which will be conducted online via zoom, regarding your experiences of the game, focusing on what you felt did and did not work. The interviews will be conducted at two points during the study, post-intervention, and after a 3-week follow-up. Interview audio will be recorded and transcribed, following which the recordings and any personal information will be immediately deleted.

Are there any risks associated with taking part?

We are not aware of any significant risks associated with participation. While some of the questions will ask about existing states of mental health (e.g., levels of current anxiety and depression) – which some people may find uncomfortable – the video game has developed in accordance with recent developments in psychological science to help you manage these, and we expect that participation in this study will help to ameliorate these feelings.

If you feel affected by any issues raised by this research and would like to discuss any concerns, please contact the principal investigators of this study as indicated in the contact details at the bottom of this information sheet. If you feel that you would benefit from further psychological or psychiatric support, we advise you to contact your GP (family doctor) in the first instance. Further information on mental wellbeing can be found at https://www.mind.org.uk/. Additional mental health information can be found at https://www.nhs.uk/mental-health/.

This research has been approved by the Department of Psychology's Research Ethics Committee.

Data Protection and Confidentiality

Your data will be processed in accordance with the Data Protection Act 2018 and the General Data Protection Regulation 2016 (GDPR). All information collected about you will be kept strictly confidential. Your data will only be viewed by the researcher/research team.

Standard ethical procedures will involve you providing your consent to participate in this study by ticking the consent box in the consent section

All electronic data will be stored on password-protected computers.

Please note that the data to be collected for our study will be made anonymous once you have completed all stages of the research, and your response data will not hold any personally identifiable information. We will allocate you with an identifier code, and keep your email information for the duration of the study, so that we can communicate with you at the time points. We will delete contact emails at the end of the study.

All data deposited in Swansea University's OneDrive for Business service is stored within Microsoft's data centres located in the EU. Swansea University retains full ownership and control over the data and is satisfied that the data is properly secured and protected.

The contractual agreements between Microsoft and Swansea University have been negotiated by the JISC on behalf of the UK HE sector and abide by all relevant UK and European legislation. In addition, the UK Government has granted Microsoft Azure and Microsoft Office 365 "OFFICIAL" accreditation. This means that they are accredited to hold or transact public sector data for business conducted at the OFFICIAL level of Security Classification.

What will happen to the information I provide?

Version 4 4 November 2022

For peer review only

An analysis of the information will form part of our report at the end of the study and may be presented to interested parties and published in scientific journals and related media. *Note that all information presented in any reports or publications will be anonymous and unidentifiable.*

Is participation voluntary and what if I wish to later withdraw?

Your participation is entirely voluntary – you do not have to participate if you do not want to. If you decide to participate, but later wish to withdraw from the study, then you are free to withdraw at any time, without giving a reason and without penalty (simply close your web browser). All identifying information will be stripped from collected data once you have completed all phases of the study. If you wish to withdraw at any time during the study, all personal information will be immediately deleted.

Data Protection Privacy Notice

The data controller for this project will be Swansea University. The University Data Protection Officer provides oversight of university activities involving the processing of personal data, and can be contacted at the Vice Chancellors Office.

Your personal data will be processed for the purposes outlined in this information sheet. Standard ethical procedures will involve you providing your consent to participate in this study by ticking the consent box on the consent page.

The legal basis that we will rely on to process your personal data will be processing in line with public interest, scientific and statistical purposes.

How long will your information be held?

Anonymised data will be preserved and accessible online, as is encouraged by developments in open science.

What are your rights?

You have a right to access your personal information, to object to the processing of your personal information, to rectify, to erase, to restrict and to port your personal information. Please visit the University Data Protection webpages for further information in relation to your rights.

Any requests or objections should be made in writing to the University Data Protection Officer:-

University Compliance Officer (FOI/DP)
Vice-Chancellor's Office
Swansea University
Singleton Park
Swansea
SA2 8PP
Email: dataprotection@swansea.ac.uk

How to make a complaint

If you are unhappy with the way in which your personal data has been processed you may, in the first instance contact the University Data Protection Officer using the contact details above.

If you remain dissatisfied then you have the right to apply directly to the Information Commissioner for a decision. The Information Commissioner can be contacted at: -

Information Commissioner's Office,
Wycliffe House,

Version 4 4 November 2022

For peer review only

Water Lane,
Wilmslow,
Cheshire,
SK9 5AF
www.ico.org.uk

What if I have other questions?
If you have further questions about this study, please do not hesitate to contact us:

Tom Gordon
Research Assistant
Swansea University
tom.gordon@swansea.ac.uk

Dr. Darren Edwards
Department of Public Health, Policy, and Social Sciences.
Swansea University
d.j.edwards@swansea.ac.uk

Prof. Andrew Kemp
Department of Psychology
Swansea University
a.h.kemp@swansea.ac.uk

For peer review only - http://bmjopen.bmj.com/site/about/guidelines.xhtml

Participant Consent Form

Swansea University
Prifysgol Abertawe

Project title: **A novel ACT-based video game to support mental health through embedded learning**

You must be age 18 or over to complete this online survey.

Name and Contact details of the principal researchers: Tom Gordon tom.gordon@swansea.ac.uk, Prof. Andrew Kemp a.h.kemp@swansea.ac.uk, Dr. Darren Edwards d.j.edwards@swansea.ac.uk.

This study is being conducted by Swansea University, Faculty and life sciences.

- I (the participant) consent to participate in the study
- I confirm that I have read and understand the information provided in relation to this study.
- I understand that this study will involve three phases, taking place over a period of three weeks. Within which I will complete a 1-hour therapy-based mobile phone videogame at home, that includes potentially upsetting themes relating to mental health.
- I understand that partaking in this study involves one-on-one online interviews using Zoom audio, and that any identifiable personal information will be immediately deleted following transcription.
- I understand that I have the option of undergoing electrocardiograph recording at three stages during the study, which will take place at Swansea University.
- I understand that my participation is voluntary. I understand that I am free to withdraw at any time during the study but once I have completed all phases of the study, withdrawal will not be possible because data will be completely anonymised.
- I understand what my role will be in this research, and all my questions have been answered to my satisfaction.
- I have been informed that the information I provide will be safeguarded.
- I am happy for the information I provide to be used (anonymously) in academic papers and other formal research outputs, however my name will not be published so anonymity is ensured.

For peer review only

- I agree to the researchers processing my personal data in accordance with the aims of the study described in the participant information.
- I am age 18 years or above.

If you agree with all statements listed above, click **YES** (I consent).

If you disagree with any of the statements above, click **NO** (I do not consent).

For peer review only

DEBRIEF FORM

Title of project: **A novel ACT-based video game to support mental health through embedded learning**

Thank you for taking part in our research. Now that your contribution has finished, let us explain the rationale behind this work.

We are interested in how a video game based on recent developments in clinical psychology might enable learning of psychological resilience skills through play. Typically, psychotherapeutic interventions are delivered through face to face sessions, but there is an increasing need for psychological support that is delivered through an online medium such as the video you have been playing in our study.

This work therefore builds on previous efforts to teach people important skills in psychological resilience through psychoeducation and embedded learning. Specifically, the game is designed to build psychological flexibility, which is a key outcome of an ACT intervention. Psychological flexibility within ACT refers to the promotion of positive mental health, contact with present emotions, wellbeing, and positive emotions.
We hope that information gained from this study will be useful for further developing our ACT-based video game and expanding further research in this area.

If you feel affected by any issues raised by this research and would like to discuss any concerns, please contact the principal investigators of this study as indicated in the details provided below. If you feel that you would benefit from further psychological or psychiatric support, we advise you to contact your GP (family doctor) in the first instance. Further information on mental wellbeing can be found at https://www.mind.org.uk/. Further information regarding mental health can be found at https://www.nhs.uk/mental-health/.

Tom Gordon
Research Assistant
Swansea University
tom.gordon@swansea.ac.uk

Dr. Darren Edwards
Department of Public Health, Policy, and Social Sciences.
Swansea University
d.j.edwards@swansea.ac.uk

Prof Andrew Kemp
Department of Psychology
Swansea University
a.h.kemp@swansea.ac.uk

For peer review only

CONSORT 2010 checklist of information to include when reporting a pilot or feasibility trial*

Section/Topic	Item No	Checklist item	Reported on page No
**Title and abstract**			
	1a	Identification as a pilot or feasibility randomised trial in the title	1
	1b	Structured summary of pilot trial design, methods, results, and conclusions (for specific guidance see CONSORT abstract extension for pilot trials)	2/3
**Introduction**			
Background and objectives	2a	Scientific background and explanation of rationale for future definitive trial, and reasons for randomised pilot trial	4-9
	2b	Specific objectives or research questions for pilot trial	8-9
**Methods**			
Trial design	3a	Description of pilot trial design (such as parallel, factorial) including allocation ratio	9
	3b	Important changes to methods after pilot trial commencement (such as eligibility criteria), with reasons	8
Participants	4a	Eligibility criteria for participants	10
	4b	Settings and locations where the data were collected	10
	4c	How participants were identified and consented	10
Interventions	5	The interventions for each group with sufficient details to allow replication, including how and when they were actually administered	12
Outcomes	6a	Completely defined prespecified assessments or measurements to address each pilot trial objective specified in 2b, including how and when they were assessed	10-12
	6b	Any changes to pilot trial assessments or measurements after the pilot trial commenced, with reasons	N/A
	6c	If applicable, prespecified criteria used to judge whether, or how, to proceed with future definitive trial	N/A
Sample size	7a	Rationale for numbers in the pilot trial	10
	7b	When applicable, explanation of any interim analyses and stopping guidelines	N/A
Randomisation:			
Sequence generation	8a	Method used to generate the random allocation sequence	N/A
	8b	Type of randomisation(s); details of any restriction (such as blocking and block size)	N/A
Allocation concealment mechanism	9	Mechanism used to implement the random allocation sequence (such as sequentially numbered containers), describing any steps taken to conceal the sequence until interventions were assigned	N/A

For peer review only - http://bmjopen.bmj.com/site/about/guidelines.xhtml

Implementation	10	Who generated the random allocation sequence, who enrolled participants, and who assigned participants to interventions	N/A
Blinding	11a	If done, who was blinded after assignment to interventions (for example, participants, care providers, those assessing outcomes) and how	N/A
	11b	If relevant, description of the similarity of interventions	N/A
Statistical methods	12	Methods used to address each pilot trial objective whether qualitative or quantitative	14-17
**Results**			
Participant flow (a diagram is strongly recommended)	13a	For each group, the numbers of participants who were approached and/or assessed for eligibility, randomly assigned, received intended treatment, and were assessed for each objective	17
	13b	For each group, losses and exclusions after randomisation, together with reasons	17
Recruitment	14a	Dates defining the periods of recruitment and follow-up	17
	14b	Why the pilot trial ended or was stopped	N/A
Baseline data	15	A table showing baseline demographic and clinical characteristics for each group	30
Numbers analysed	16	For each objective, number of participants (denominator) included in each analysis. If relevant, these numbers should be by randomised group	17
Outcomes and estimation	17	For each objective, results including expressions of uncertainty (such as 95% confidence interval) for any estimates. If relevant, these results should be by randomised group	30
Ancillary analyses	18	Results of any other analyses performed that could be used to inform the future definitive trial	17-29
Harms	19	All important harms or unintended effects in each group (for specific guidance see CONSORT for harms)	N/A
	19a	If relevant, other important unintended consequences	N/A
**Discussion**			
Limitations	20	Pilot trial limitations, addressing sources of potential bias and remaining uncertainty about feasibility	33-34
Generalisability	21	Generalisability (applicability) of pilot trial methods and findings to future definitive trial and other studies	31
Interpretation	22	Interpretation consistent with pilot trial objectives and findings, balancing potential benefits and harms, and considering other relevant evidence	31-35
	22a	Implications for progression from pilot to future definitive trial, including any proposed amendments	34-35
**Other information**			
Registration	23	Registration number for pilot trial and name of trial registry	3
Protocol	24	Where the pilot trial protocol can be accessed, if available	35
Funding	25	Sources of funding and other support (such as supply of drugs), role of funders	35
	26	Ethical approval or approval by research review committee, confirmed with reference number	36

For peer review only

Citation: Eldridge SM, Chan CL, Campbell MJ, Bond CM, Hopewell S, Thabane L, et al. CONSORT 2010 statement: extension to randomised pilot and feasibility trials. BMJ. 2016;355. This is an Open Access article distributed in accordance with the terms of the Creative Commons Attribution (CC BY 3.0) license (http://creativecommons.org/licenses/by/3.0/), which permits others to distribute, remix, adapt and build upon this work, for commercial use, provided the original work is properly cited.

*We strongly recommend reading this statement in conjunction with the CONSORT 2010, extension to randomised pilot and feasibility trials, Explanation and Elaboration for important clarifications on all the items. If relevant, we also recommend reading CONSORT extensions for cluster randomised trials, non-inferiority and equivalence trials, non-pharmacological treatments, herbal interventions, and pragmatic trials. Additional extensions are forthcoming: for those and for up-to-date references relevant to this checklist, see www.consort-statement.org.

For peer review only

For peer review only - http://bmjopen.bmj.com/site/about/guidelines.xhtml

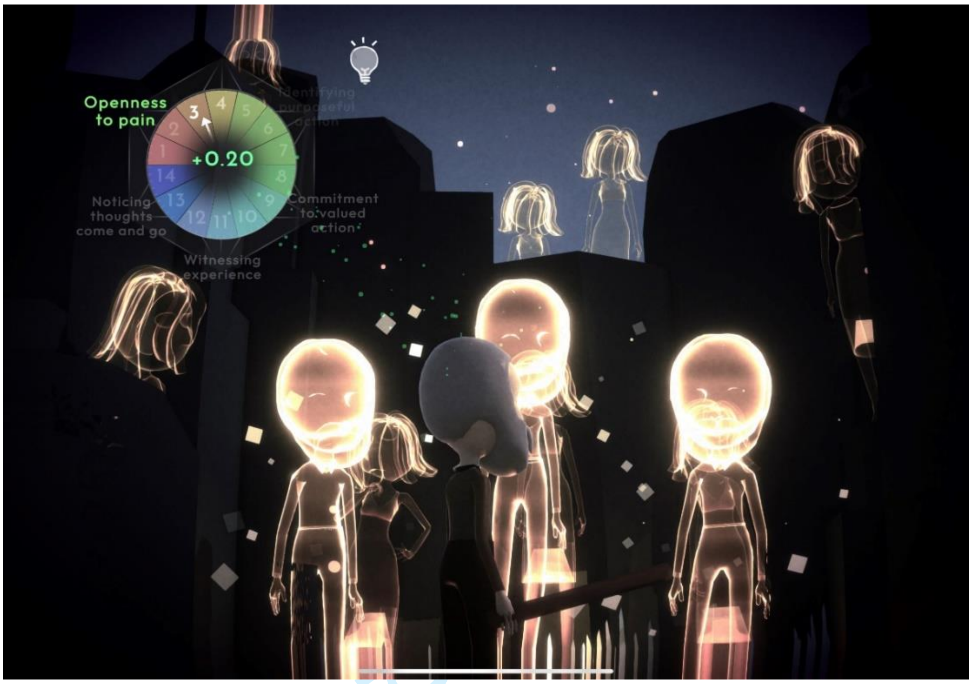
Openness to pain
+0.20
Identifying purposeful action
Commitment to valued action
Witnessing experience
Noticing thoughts come and go

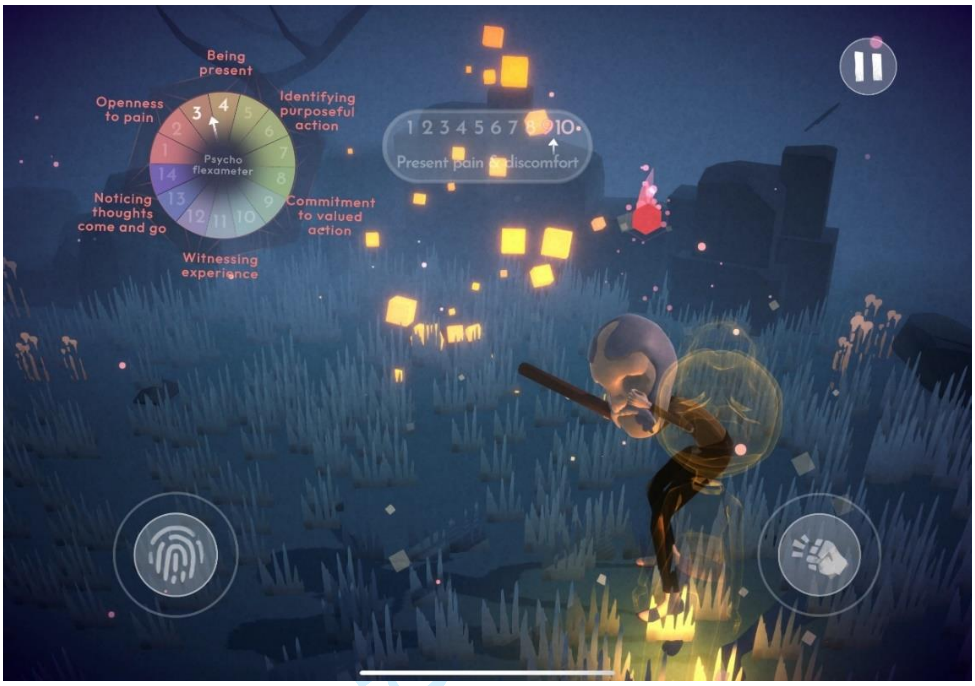

For peer review only

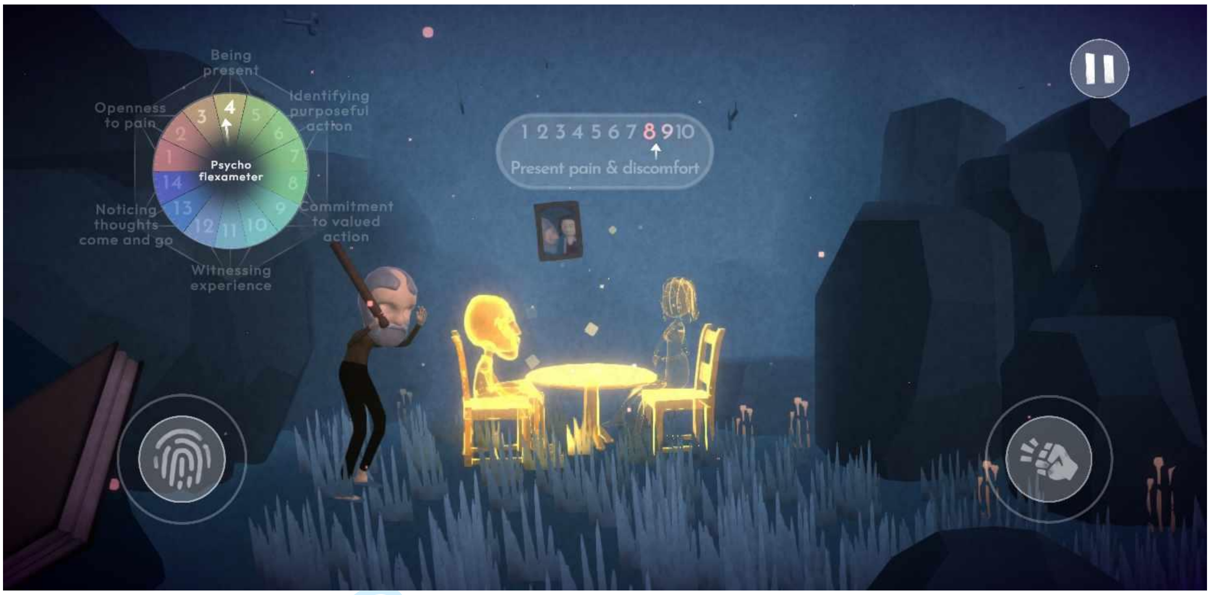
Being present
Identifying purposeful action
Openness to pain
Psycho flexameter
Commitment to valued action
Noticing thoughts come and go
Witnessing experience
1 2 3 4 5 6 7 8 9 10
Present pain & discomfort

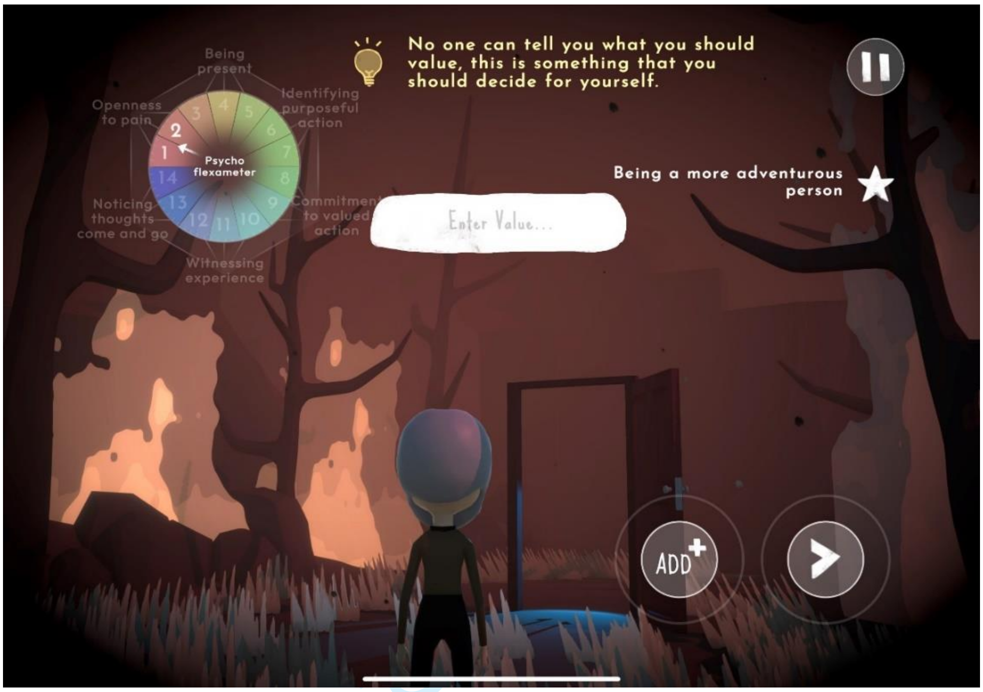
No one can tell you what you should value, this is something that you should decide for yourself.
Being present
Identifying purposeful action
Openness to pain
Psycho flexameter
Noticing thoughts come and go
Commitment to valued action
Witnessing experience
Being a more adventurous person
Enter Value...
ADD
